# A QM/MM–Based Computational Investigation on the Catalytic Mechanism of Saccharopine Reductase

**DOI:** 10.3390/molecules16108569

**Published:** 2011-10-12

**Authors:** Almasi Joel N., Bushnell Eric A.C., James W. Gauld

**Affiliations:** Department of Chemistry and Biochemistry, University of Windsor, Windsor, Ontario N9B 3P4, Canada

**Keywords:** Schiff base, saccharopine reductase, α-aminoadipate-δ-semialdehyde, saccharopine, imine formation, carbinolamine, QM/MM, theoretical, computational

## Abstract

Saccharopine reductase from *Magnaporthe grisea*, an NADPH-containing enzyme in the α-aminoadipate pathway, catalyses the formation of saccharopine, a precursor to L-lysine, from the substrates glutamate and α-aminoadipate-δ-semialdehyde. Its catalytic mechanism has been investigated using quantum mechanics/molecular mechanics (QM/MM) ONIOM-based approaches. In particular, the overall catalytic pathway has been elucidated and the effects of electron correlation and the anisotropic polar protein environment have been examined via the use of the ONIOM(HF/6-31G(d):AMBER94) and ONIOM(MP2/6-31G(d)//HF/6-31G(d):AMBER94) methods within the mechanical embedding formulism and ONIOM(MP2/6-31G(d)//HF/6-31G(d):AMBER94) and ONIOM(MP2/6-311G(d,p)//HF/6-31G(d):AMBER94) within the electronic embedding formulism. The results of the present study suggest that saccharopine reductase utilises a substrate-assisted catalytic pathway in which acid/base groups within the cosubstrates themselves facilitate the mechanistically required proton transfers. Thus, the enzyme appears to act most likely by binding the three required reactant molecules glutamate, α-aminoadipate-δ-semialdehyde and NADPH in a manner and polar environment conducive to reaction.

## 1. Introduction

The genetic material of organisms contains the codons for twenty “standard” α-amino acids. Despite their central importance for the construction of cellular proteins and enzymes, however, not all cells are able to synthesize all twenty *de novo*. For instance, the ability to biosynthesize the essential amino acid L-lysine is limited to some green plants, bacteria, fungi, and cyanobacteria [1,2]. In addition, it has been observed to occur via just two distinct routes: (i) the diaminopimelate (green plants, bacteria, and lower fungi), and (ii) the α-aminoadipate (cyanobacteria and higher fungi) pathways [3]. For example, the fungal species, *Candida albicans*, *Cryptococcus neoformans*, *Aspergillus fumigatus*, *Saccharomyces cerevisiae* and *Magnaporthe grisea* have all been shown to utilise the α-aminoadipate pathway [1]. The former three are all human fungal agents and pose a risk to those with compromised immune systems such as AIDS, cancer, and transplant patients [4]. *Magnaporthe grisea*, on the other hand, affects many grass and crop species’ and is perhaps best known for causing rice blast disease [5]. Thus, this pathway represents an attractive target for the development of new fungicides [6].

**Scheme 1:**
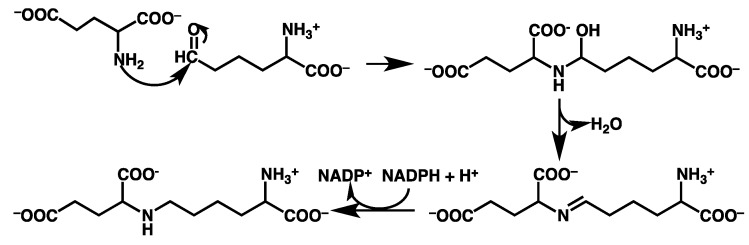
The catalytic mechanism of saccharopine reductase as proposed by Johansson *et al.* [4].

Saccharopine reductase is a key enzyme in the α-aminoadipate pathway. Specifically, it catalyses the condensation of α-aminoadipate-δ-semialdehyde (AASA) with glutamic acid and subsequent reduction by NADPH of the resulting Schiff base to give the L-lysine precursor saccharopine [1]. It has been found that the substrates of saccharopine reductase bind in the order of NAPDH, AASA and lastly glutamate [1]. However, after the binding of the substrates, two possible catalytic mechanisms have been proposed for saccharopine reductase [1,4]. Johansson *et al.* [4] obtained X-ray crystal structures of the apo-enzyme and an enzyme…saccharopine/NADPH complex. Based on these structures they suggested that there are no suitable active-site acid/base residues able to facilitate the mechanistically required proton transfers. Hence, they concluded that the observed catalytic rate enhancement of saccharopine reductase is instead due to favourable positioning of the substrates with respect to each other within the active-site [4]. Consequently, they proposed the catalytic mechanism outlined in Scheme 1. Notably, the α-amino of the glutamic acid is initially neutral, while the α-carboxylate groups are both ionised. Thus, the first step in the overall mechanism is nucleophilic attack of the Glu-NH_2_ nitrogen (^Glu^N) at the R-group carbonyl carbon (^AASA^C) of AASA. This occurs with concomitant transfer of a proton to AASA’s R-group carbonyl oxygen (^AASA^O) and loss of a proton from the bridging amine to give a carbinolamine intermediate. The latter then formally undergoes a 1,3-intramolecular proton transfer from its -^Glu^NH- moiety to the newly formed hydroxyl (^AASA^OH) group, resulting in loss of water and formation of an unprotonated-Schiff base intermediate. In the third and final step the Schiff base is reduced via hydride transfer from the NADPH cofactor with a concomitant protonation by an unknown moiety to give saccharopine.

**Scheme 2:**
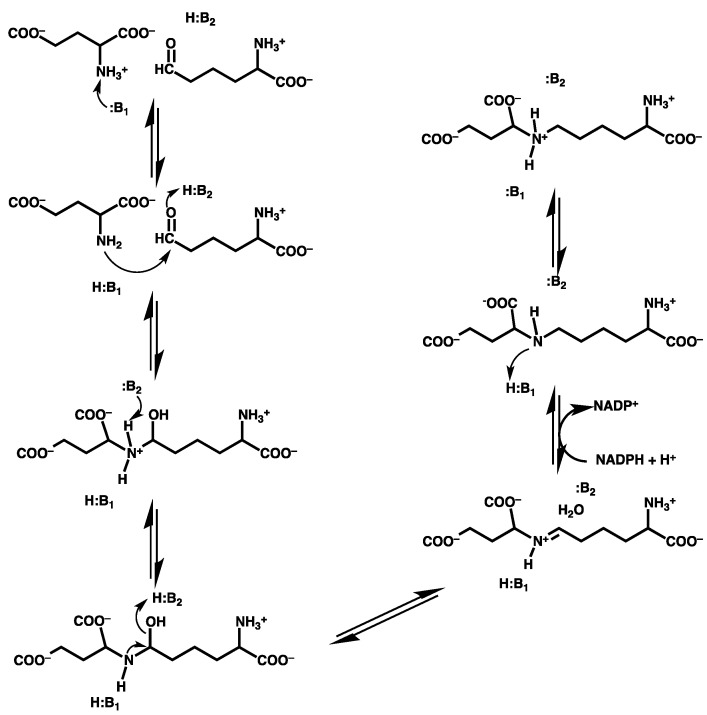
The general acid/base catalytic mechanism as proposed by Vashishtha *et al.* [1].

More recently, Vashishtha *et al.* [1] experimentally examined pH-rate profiles and solvent deuterium kinetic isotope effects of saccharopine reductase from *S. cerevisiae*. Based on their observations, they concluded that it utilises an acid/base mechanism involving two active-site residues and proposed the catalytic mechanism outlined in Scheme 2. Specifically, an active-site base (**B1**), estimated [1] to have a p*K*_a_ in the range of 5.6–5.7, initially deprotonates the glutamates protonated α-amino group. However, as noted by Johannson *et al.* [4] it is not clear what group may act as this general base. As a result, the ^Glu^N centre is then able to nucleophilically attack at the ^AASA^C centre. However, in contrast to that proposed by Johansson *et al.* [4] this occurs concomitant protonation of the ^AASA^O centre by an acidic active site residue (**H:B2**) with an estimated p*K*_a_ of 7.8–8. Based on their X-ray crystal structure of the enzyme-product complex, Johannson *et al.* [4] concluded that no obvious active site residue exists to facilitate this protonation. However, Vashishtha *et al.* [1] have alternatively suggested that an aspartyl (Asp126) may be able to protonate the oxyanion formed during nucleophilic attack of the glutamates amine. This results in formation of a ^Glu^N-protonated carbinolamine intermediate. Subsequently, **B2** abstracts a proton from the intermediate’s -^Glu^NH_2_^+^- moiety before transferring it onto the newly formed nearby ^AASA^OH group, resulting in formation of a protonated-Schiff base intermediate with loss of water (see Scheme 2). The Schiff-base is then reduced by hydride transfer from the NADPH cofactor onto the imines carbon centre, thus giving saccharopine. In the last two steps the initial active-site is regenerated with assistance of the product itself; **H:B1** transfers its proton to **B2** via the saccharopine’s -^Glu^NH- group to reform **B1** and **H:B2**, *i.e.*, their initial states. Unfortunately, they were unable to identify the exact active-site acid/base residues involved.

At present, there have been no computational investigations on the catalytic mechanism of saccharopine reductase. However, Schiff base formation has been extensively studied both experimentally and computationally due in part to their common occurrence as reaction intermediates in biochemistry and chemistry [4,7,8,9]. From these studies it has been shown that Schiff base formation depends on several factors including the solvent, pH, and the chemical nature of the reactants [7,10,11,12,13,14,15]. Mechanistically, it can be thought of occurring in two stages: (i) initial formation of a carbinolamine-type intermediate via nucleophilic attack of an amino group at a carbonyl carbon, followed by (ii) loss of its carbinolamine hydroxyl as water to give the corresponding imine. Overall, Schiff base formation is favoured at neutral pH. However, markedly lower reaction barriers are obtained if a water or some other suitable moiety facilitates the required proton transfers [7]. In contrast, under acidic conditions, *i.e.*, those in which the attacking amino group is initially protonated, the first stage is slow as it requires deprotonation of the amino group while the subsequent stage, loss of water, is quite rapid.

Elucidation of an enzymes catalytic mechanism is central to a complete understanding of its biochemical role and the development of effective inhibitors. In this present study we have used ONIOM quantum mechanics/molecular mechanics (QM/MM) computational methods to investigate the overall mechanism of saccharopine reductase. In particular, we have examined the initial protonation states of key substrate functional groups and possible mechanistic roles of the substrates own acid/base groups.

## 2. Computational Methods

For all calculations the combined quantum mechanical and molecular mechanical (QM/MM) method in the ONIOM [16,17,18,19,20,21,22,23,24] formalism was applied as implemented within the Gaussian 09 program suite [25].

Density functional theory (DFT) is a common tool for investigating biochemical reactions [26]. However, it has a tendency to underestimate barriers, in particular, those corresponding to proton transfers [27]. In contrast, Hartree-Fock (HF) tends to overestimate barriers for proton transfer [27]. However, in a related computational investigation Williams [28] studied the condensation reaction between ammonia and formaldehyde at the HF/3-21G level of theory. They found that for nucleophilic attack of the amino at the carbonyl carbon, the lowest barrier to formation of the carbinolamine intermediate was obtained when two water molecules were involved in the reaction, in agreement with experimental predictions [28]. Later, as part of a computational study on Schiff base formation in the same chemical system, Hall and Smith [29] re-examined the reaction steps leading to formation of the carbinolamine intermediate at the considerably higher G2(MP2,SVP) level of theory. Importantly, they obtained the same series of reaction steps leading to formation of the carbinolamine intermediate as previously found by Williams [28]. although the relative energies differed [28].

Thus, all geometry optimizations were performed at the ONIOM(HF/6-31G(d):AMBER94) level of theory within the mechanical embedding (ME) formalism [30]. Harmonic vibrational frequency calculations of stationary points along the potential energy surface (PES) were performed at the same level of theory in order to characterize them as minima or transition structures and to calculate Gibbs free energy corrections at standard ambient temperature and pressure (SATP).

Relative energies were then obtained by performing single point (SP) calculations at higher levels of theory based on the above-optimized geometries, with inclusion of the appropriate free energy correction. Specifically, SP calculations were performed at the; (i) ONIOM(MP2/6-31G(d)//HF/6-31G(d):AMBER94)-ME; (ii) ONIOM(MP2/6-31G(d)//HF/6-31G(d):AMBER94) within the electronic embedding (EE) formalism; and (iii) ONIOM(MP2/6-311G(d,p)//HF/6-31G(d):AMBER94)-EE levels of theory. These were chosen in order to enable systematic consideration of the effects of (i) incorporation of electron correlation, (ii) the anisotropic protein environment on the reactive core (*i.e.*, QM layer) and (iii) increasing the basis set size, respectively. It is noted that all energies reported herein have been corrected by adding the necessary Gibbs corrections discussed above.

**Figure 1:**
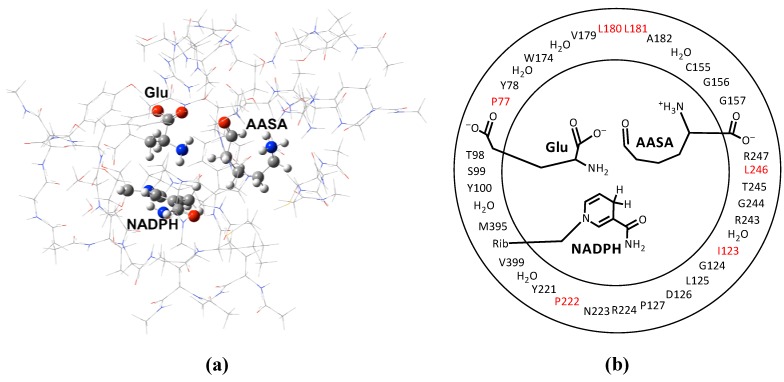
**(a)** The X-ray crystal structure (PDB ID: 1E5Q)-derived QM/MM model used to investigate the catalytic mechanism of saccharopine reductase. **(b)** Schematic representation of the QM/MM model: groups in the inner and outer circles have been modelled at the HF/6-31G(d) and AMBER94 levels of theory, respectively. Note, residues, waters and functional groups in black have been included in the model in their entirety, while residues in red have only had their peptide backbone included.

A large active-site model for saccharopine reductase from *M. grisea* was obtained from an X-ray crystal structure [PDB: 1E5Q] of the enzyme co-crystallized with the saccharopine product and is shown in Figure 1. Specifically, both the glutamate and α-aminoadipate-δ-semialdehyde (AASA) substrates and reactive moiety of the NADPH cofactor were included in the QM layer. All residues and waters within 15 Å of the cosubstrates were included in the MM layer either in their entirety or only including their peptide backbone component (*i.e.*, the residue was modelled as -NHCH_2_CO-). The saccharopine moiety in the X-ray crystal structure was replaced by the two cosubstrates accordingly. Specifically, the C-N bond was cleaved and hydrogens added to the nitrogen to regenerate the initial glutamate substrate while an oxygen was added to the carbon centre thus reforming the initial α-aminoadipate-δ-semialdehyde cosubstrate. Hydrogens were added to the active-site model with all ionisable functional groups being modelled in their most likely protonation state at pH = 7. To ensure the integrity of the model during calculations the α-carbon of each residue was held fixed at its crystal structure position. It should be noted that residue Tyr100 was included in the QM layer for the examination of the initial protonation state of the Glu-NH_2_ moiety and in the MM layer for the mechanism studies (see below).

## 3. Results and Discussion

### 3.1. The pK_a_ of the Substrate Glutamate’s α-Amine

It has been suggested [1] that for favourable binding, the amines of both the glutamate and AASA substrates must initially be protonated. Then, once bound, the glutamate’s α-NH_3_^+^ group deprotonates thus enabling it to act as a nucleophile [1]. Hence, prior to an investigation of the catalytic mechanism the likely initial protonation state of the glutamate’s α-amine (Glu-NH_2_) was examined.

In particular we have considered the proton affinities (PAs) of the α-amine of glutamate and AASA in aqueous solution and when bound in the active site, and that of H_2_O_(aq)_, *i.e.*, a water in the bulk aqueous environment. It is noted that the PA of an acidic group is simply the difference in electronic energy between a base (X^−^) and its conjugate acid (HX) as shown in Equation 1:

PA = E(X^−^) − E(HX) (1)

**Figure 2:**
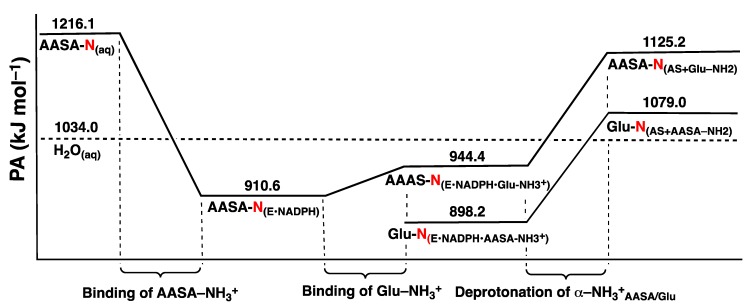
The PAs for the α-amines of AASA and Glu with respect to the local environment. The horizontal dashed line represents the PA of H_2_O in solution.

More specifically, the PAs of AASA-NH_2(aq)_, Glu-NH_2(aq)_ and H_2_O_(aq)_ were obtained at the IEF-PCM(ε = 78.3553)/MP2/6-311 + G(2df,p)//HF/6-31G(d) level of theory. The PAs of AASA-NH_2_ and Glu-NH_2_ when bound within the active site were obtained at the ONIOM(MP2/6-311+G(2df,p)//HF/6-31G(d):AMBER94) level of theory within the electronic embedding formulism. It is noted that in each of these “bound systems” the substrates carboxylates were modelled in their ionised forms (*i.e.*, -COO^−^). The PAs obtained are illustrated in Figure 2.

In aqueous solution the PA of AASA-NH_2_ is calculated to be 1,216.1 kJ mol^−1^, which is significantly greater than that calculated for H_2_O_(aq)_ (1,034.0 kJ mol^−1^). Given the similarities in the α-amines of Glu and AASA, their PAs are expected to be in close agreement. Thus, in aqueous solution both AASA-NH_2_ and Glu-NH_2_ are likely protonated.

As noted by Vashishtha *et al.* [1] the NADPH binds within the active site first to give an E•NADPH complex, followed by AASA and then Glu. From Figure 2 it can be seen that upon binding of AASA to E•NADPH the PA of its α-amine (**AASA-N_(E•NADPH)_**) drops significantly to 910.6 kJ mol^−1^. In fact, it is now lower than that of H_2_O_(aq)_, thus suggesting that once bound the AASA-NH_3_^+^ group could readily donate a proton to the bulk solution.

The next step is binding of Glu to the E•NADPH•AASA complex. As noted above, it has been suggested [1] that for binding the α-amine of Glu must be protonated. From Figure 2 it can be seen that upon binding of Glu-NH_3_^+^ the PA of AASA-NH_2_ (**AASA-N_(E•NADPH•Glu-NH3+)_**) increases to 944.4 kJ mol^−1^. In order for the α-amine of Glu to act as a nucleophile it must be neutral, *i.e.*, Glu-NH_3_^+^ must lose a proton. As noted by Johannson *et al.* [4] there appears to be no suitable base within the active site to deprotonate Glu-NH_3_^+^. However, from Figure 2 it can be seen that deprotonation of the glutamates α-amine results in a significant increase inthe PA of AASA-NH_2_ to 1,125.2 kJ mol^−1^. In contrast, the PA of the resulting Glu-NH_2_ moiety is lower at 1,079.0 kJ mol^−1^. Importantly, both of these PAs are now larger than that of H_2_O_(aq)_. This suggests that it is unlikely for the α-amine of both substrates (AASA and Glu) to be at least simultaneously neutral; in such a case they both can potentially accept a proton from the bulk aqueous solution. However, if the more basic AASA-NH_2_ group does take up a proton, *i.e.*, becomes AASA-NH_3_^+^, the PA of the Glu-NH_2_ decreases considerably to 898.2 kJ mol^−1^, and is now in fact lower than that of H_2_O_(aq)_. Furthermore, it has the lowest PA of all possible both-substrates-bound configurations considered herein. That is, the preferred configuration of the fully bound active site has a neutral Glu-NH_2_ and protonated AASA-NH_3_^+^.

### 3.2. Mechanism for Formation of Saccharopine

**Figure 3:**
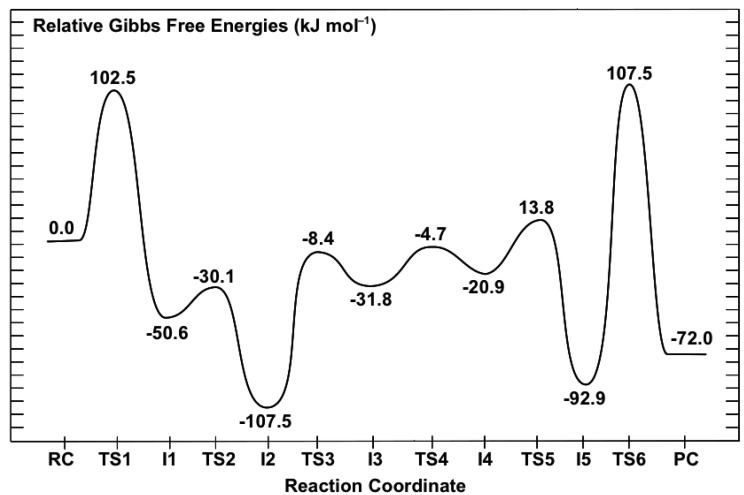
Overall PES for the catalytic mechanism of saccharopine reductase obtained at the ONIOM(HF/6-31G(d):AMBER94)-ME level of theory with inclusion of Gibbs corrections.

**Figure 4:**
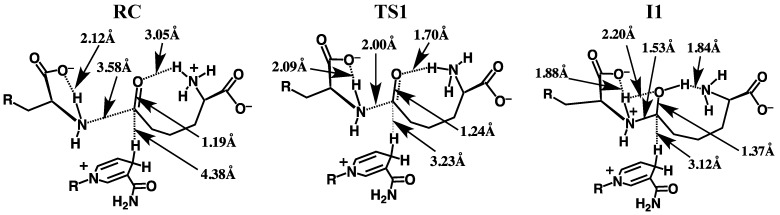
Optimized structures obtained at the ONIOM(HF/6-31G(d):AMBER94)-ME level of theory of the reactant complex (**RC**), transition structure (**TS1**) and the carbinolamine intermediate **I1** with selected distances shown (in Angstroms).

**Figure 5:**
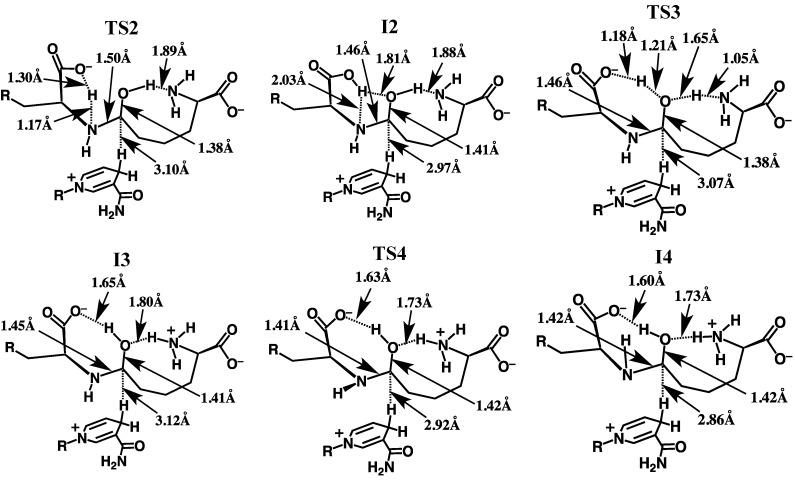
Optimized structures of **TS2**, **I2**, **TS3**, **I3**, **TS4** and **I4** obtained at the ONIOM(HF/6-31G(d):AMBER94)-ME level of theory with selected distances shown (in Angstroms).

**Figure 6:**
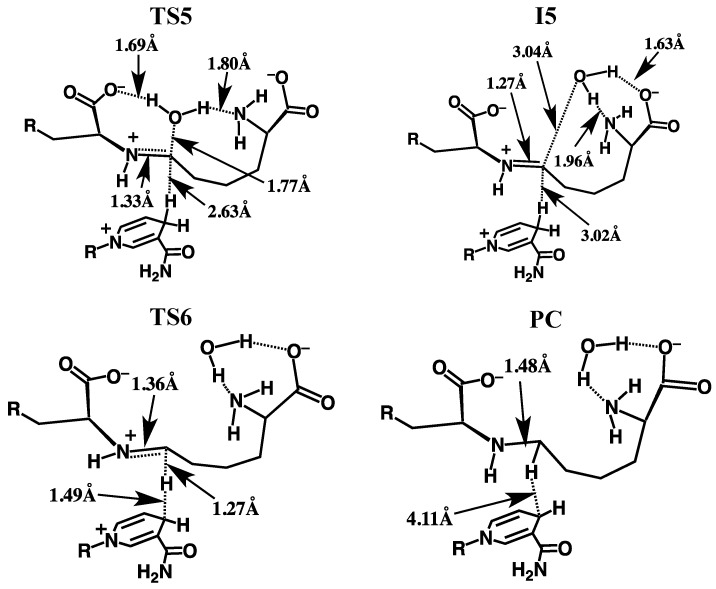
Optimized structures of **TS5**, **I5** , **TS6** and **PC** obtained at the ONIOM(HF/6-31G(d):AMBER94)-ME level of theory with selected distances shown (in Angstroms).

The overall potential energy surface (PES) obtained for the catalytic mechanism of saccharopine reductase at the ONIOM(HF/6-31G(d):AMBER94)-ME level of theory with inclusion of Gibbs free energy corrections is presented in Figure 3. The optimized geometries of the corresponding reactant, product, intermediate complexes and transition structures, with selected distances, are presented in Figure 4, Figure 5 and Figure 6.

In the optimized structure of the reactant complex (**RC**) both cosubstrates form intramolecular hydrogen bonds. Specifically, in the glutamate moiety its α-amino and -carboxylate groups form a reasonably strong hydrogen bond with an NH…O distance of 2.12 Å (Figure 4). Meanwhile, in the α-aminoadipate-δ-semialdehyde (AASA) cosubstrate its R-group carbonyl oxygen weakly hydrogen bonds with its protonated α-amino group with an ^AASA^CO…H_3_N^+^-AASA distance of 3.05 Å. More importantly, however, the distance between the nitrogen centre of Glu-NH_2_ and the R-group carbonyl carbon of AASA (^AASA^C), *i.e.*, *r*(Glu-N(H_2_)…(O)C^AASA^), is 3.58 Å. While this distance is quite long, it is shorter than that observed between these same two centres in gas-phase calculations on the complexed isolated substrates (*i.e.* in the absence of active-site and NADPH); 3.83 Å (not shown). It is also noted that the distance from the hydrogen on NADPH to be transferred later in the mechanism as a hydride to ^AASA^C is quite long at 4.38 Å (see Figure 4). Thus, at least initially the two co-substrates and cofactor appear to interact only weakly within the active-site. However, they all appear to be suitably positioned to react further.

#### 3.2.1. Formation of a Carbinolamine Intermediate

The first step in the overall pathway is nucleophilic attack of the glutamates α-amino nitrogen at the R-group carbonyl carbon centre of the cosubstrate AASA. This occurs via transition structure **TS1** at a cost of 102.5 kJ mol^−1^ at the ONIOM(HF/6-31G(d):AMBER94)-ME + Gibbs corrections level of theory (Figure 3). In **TS1** the ^Glu^N…C^AASA^ distance has shortened markedly to 2.00 Å while concomitantly the O-C^AASA^ bond has lengthened to 1.24 Å, *i.e.*, has reduced double-bond character (Figure 4). The nucleophilicity of the attacking amine nitrogen is likely slightly enhanced by the modest decrease in the length of the glutamates intramolecular α-NH…–OOC-Glu hydrogen bond to 2.09 Å. In addition, however, the electrophilicity of the ^AASA^C centre is enhanced by the significant decrease in the intramolecular ^AASA^CO…H_3_N^+^-AASA hydrogen bond to just 1.70 Å.

In the resulting carbinolamine intermediate **I1**, lying 50.6 kJ mol^–1^ lower in energy than **RC** , the newly formed ^AASA^C-N^Glu^ bond has a length of 1.53 Å, slightly longer than a typical C-N single bond (HF/6-31G(d): *r*(CH_3_-NH_2_) = 1.46 Å). Concomitantly, the ^AASA^C-O bond has lengthened to 1.37 Å and a proton has now been transferred onto its oxygen centre from the ^+^H_3_N-AASA group. While this is a substantial increase from that observed in **RC** , it is still shorter than for a typical C-O single bond (HF/6-31G(d): *r*(CH_3_-OH) = 1.40 Å). This is likely due to the fact that the newly formed ^AASA^COH group maintains a short, strong hydrogen bond with the AASA-NH_2_ nitrogen centre (Figure 4). It should be noted that the ^Glu^NH…–OOC-Glu hydrogen bond has also shortened to 1.88 Å. Furthermore, the distance between the mechanistically important NADPH hydrogen and the intermediates ^AASA^C centre has decreased markedly by 1.26 Å to 3.12 Å in **I1**.

Vashishtha *et al.* [1] have suggested that an acidic residue within the active-site with a p*K*_a_ of 7.8–8.0, possibly an aspartate (Asp126), protonates the oxyanion formed during nucleophilic attack of the glutamates amine. In the optimized structure of **RC** , the side chain of Asp126 hydrogen bonds with that of Arg243 and thus, it would seem unlikely to be able to act as a general acid. Furthermore, the p*K*_a_ of an aspartate R-group carboxylate in aqueous solution is 3.8. Hence, the protein environment would have to significantly perturb its p*K*_a_ upwards by approximately 4 or more units. In addition, in the optimized structure of **RC** the side chains of Asp126 and Arg243 form a hydrogen bonded ion pair and thus, Asp126 is unlikely to be able to act as a general acid. In contrast, in aqueous solution primary amines such as that of the cosubstrate AASA (AASA-NH_3_^+^) typically have p*K*_a_’s in the range of 9–10 and these values can be lowered when placed within the less polar environment of a protein’s active site. Indeed, the p*K*_a_ measured by Vashishtha *et al.* [1] is only slightly lower than what one would anticipate for AASA-NH_3_^+^, the acidic group that protonates the ^AASA^O centre in our present mechanism, in aqueous solution. 

#### 3.2.2. Rearrangement of the Carbinolamine Intermediate **I1**

Before Schiff base formation the carbinolamine intermediate **I1** must undergo a rearrangement to allow for loss of H_2_O; specifically, deprotonation of the bridging -NH_2_^+^- moiety and inversion of the resulting -NH- group.[7]

In saccharopine reductase this proceeds in a stepwise manner with the first being transfer of a proton from the bridging -^Glu^NH_2_^+^- group onto what was initially the glutamate’s carboxylate group. This occurs via **TS2** with a quite low barrier of only 20.5 kJ mol^−1^ relative to **I1** (Figure 3) The resulting “neutral” carbinolamine intermediate **I2** lies significantly lower in energy than **I1** by 56.9 kJ mol^−1^, most likely due to the neutralisation of charges. As can be seen in Figure 5, in **I2** the ^Glu^N-C^AASA^ bond has shortened considerably by 0.07 Å to 1.46 Å; a typical C-N single bond length (see above). Concomitantly, the C-OH bond has lengthened by 0.04 Å to 1.41 Å which similarly, is a length more typical of a C-O single bond (see above). The mechanistically important NADPH hydrogen is also now significantly closer by 0.15 Å to the intermediates ^AASA^C centre. It should be noted, that the proton transferred onto the glutamate’s carboxylate now forms a bifurcated hydrogen bond with both the bridging -^Glu^NH- nitrogen *and* the oxygen of the ^AASA^C-OH moiety (Figure 5). Furthermore, the Glu-COOH…N^Glu^ interaction inhibits the bridging -^Glu^NH- group from inverting. As noted in the Introduction, Vashishtha *et al.* [1] have suggested that a general base with a p*K*_a_ in the range of 5.6–5.7 deprotonates the α-amine of glutamate prior to nucleophilic attack. However, as detailed above, upon binding the Glu-NH_3_^+^ group appears able to readily lose a proton to the bulk aqueous solution. The above results suggest that the carboxylate originating from the substrate glutamate may be the acid/base group measured. Indeed, while the p*K*_a_ of the α-COO^−^ of glutamate in aqueous solution is 2.1, that of acetic acid is markedly higher at 4.8. Furthermore, as noted above, the low polarity of the protein environment can also induce a shift in measured p*K*_a_’s.

Thus, the next step is transfer of the Glu-COOH proton onto the carbinolamine’s ^AASA^C-OH hydroxyl group, which itself simultaneously donates its proton to the nearby H_2_N-AASA amino moiety. This double proton transfer proceeds via **TS3** at a cost of 99.1 kJ mol^−1^ with respect to **I2**. It is noted that in **TS3** the -OH proton is essentially almost wholly transferred onto the H_2_N-AASA moiety while that of the Glu-COOH lies almost equidistant between both the carboxylate and carbinolamine oxygen’s (see Figure 5). The resulting “charged” carbinolamine intermediate **I3** lies 75.7 kJ mol^−1^ higher in energy than **I2**, which is still 31.8 kJ mol^–1^ lower than that of the initial reactant complex **RC** (Figure 3). Importantly, this reaction has now removed the Glu-COOH…N^Glu^ interaction. It is noted that we were unable to obtain any carbinolamine intermediate that contained a neutral Glu-COOH group that did not have a Glu-COOH…N^Glu^ hydrogen bond.

The bridging -^Glu^NH- moiety is then able to undergo an inversion as is required to enable loss of the carbinolamine -OH group as H_2_O to form the Schiff base.[7] This inversion allows for the overlap in the non-bonding MO containing the nitrogen’s lone pair and the anti-bonding MO of the C-O bond. Such an overlap weakens the C-O leading to a more facile bond cleavage process. The process of inversion occurs via **TS4** with a barrier of 12.6 kJ mol^−^^1^ with respect to **I3**, to give the alternate carbinolamine intermediate **I4** lying 10.9 kJ mol^−^^1^ higher in energy than **I3**. As can be seen in Figure 5, in **I4** a marginal shortening and lengthening of the ^AASA^C-N and ^AASA^C-O bonds, respectively is observed. In addition, the distance between the mechanistically important NADPH hydrogen and ^AASA^C centre has decreased further to 2.86 Å (Figure 5).

#### 3.2.3. Formation of the Schiff Base and its Reduction

Once the bridging -^Glu^NH- has inverted, formation of the corresponding “N-protonated” Schiff base **I5** can then occur via loss of the carbinolamine -OH as water. This is achieved in one step by transfer of a proton from the AASA-NH_3_^+^ group onto the ^AASA^C-OH oxygen centre and occurs via **TS5** with a barrier of only 34.7 kJ mol^−1^ with respect to **I4** (Figure 3). The resulting imine intermediate **I5** lies markedly lower in energy than **I4** by 72.0 kJ mol^−1^. It is noted that in **I5** the bridging ^Glu^N-^AASA^C bond has shortened significantly to 1.27 Å while the water that was released remains hydrogen bonded to both the α-amino and -carboxylate of the initial AASA cosubstrate. In addition, while the distance between the NADPH hydrogen and the ^AASA^C centre has increased by 0.16 Å to 3.02 Å, it is still significantly closer than observed in **RC** (*cf.* Figure 4).

The final step in the overall catalytic pathway is formation of the saccharopine product via reduction of the Schiff base by a hydride transfer from NADPH onto the ^AASA^C centre of **I5** . It is noted that experimentally it has been found that nucleophilic attack of the N=C double bond only occurs when the Schiff base is protonated (*i.e.*, when in its iminium form) as is the case for **I5** [29,31]. This H^−^ transfer step proceeds via **TS6** with a barrier of 107.5 kJ mol^−1^ with respect to **RC** at the ONIOM(HF/6-31G(d):AMBER94)-ME + Gibbs corrections level of theory. This barrier is lower than the generally accepted upper thermodynamic limit for enzymatic reactions of approximately 120 kJ mol^−1^ [32]. However, it corresponds to a reaction barrier of 200.4 kJ mol^–1^ relative to **I5** and thus, at the level of theory above is predicted to at least be kinetically unfavourable (Figure 3).

The final active-site bound-saccharopine complex (PC) is −72.0 kJ mol^−1^ lower in energy than the initial active-site bound-reactant complex RC. Thus, overall, the pathway is calculated to be exothermic and thus thermodynamically favoured at the ONIOM(HF/6-31G(d):AMBER94)-ME + Gibbs corrections level of theory (Figure 3).

### 3.3. Obtaining More Accurate and Reliable Energies for the Mechanism of Saccharopine Reductase

**Figure 7:**
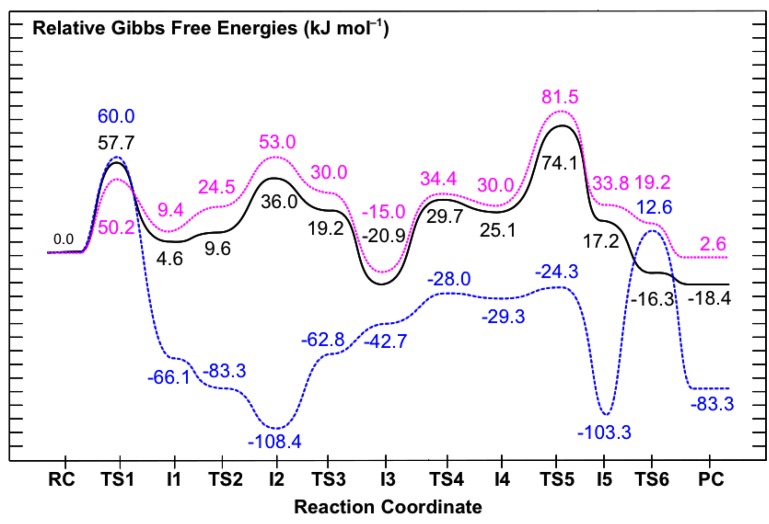
Overall PES’s obtained for the catalytic mechanism of saccharopine reductase at the (i) ONIOM(MP2/6-31G(d)//HF/6-31G(d):AMBER94)-ME + Gibb’s Corrections (dashed blue line), (ii) ONIOM(MP2/6-31G(d)//HF/6-31G(d):AMBER94)-EE + Gibb’s Corrections (dotted pink line), and (iii) ONIOM(MP2/6-311G(d,p)//HF/6-31G(d):AMBER94)-EE + Gibb’s Corrections (solid black line) levels of theory.

As noted in the Computational Methods, previous studies have shown that the Hartree-Fock level of theory can provide a reliable mechanistic pathway for Schiff base formation, although the associated relative energies may be less accurate. However, by careful choice of higher levels of theory one can systematically consider the effects of, for example, electron correlation and the polarity of the protein environment surrounding the reactants and enzyme active-site. This is usually done by performing single-point (SP) calculations at higher levels of theory that are based on the geometries optimized at a lower level of theory, in this case ONIOM(HF/6-31G(d):AMBER94)-ME. These provide more accurate relative energies and hence, potential energy surfaces. Thus PES’s were then obtained at several systematically higher levels of theory and which are presented in Figure 7. In order to facilitate comparison with the PES in Figure 3, the relative energy of **RC** at all levels of theory have been set to zero.

#### 3.3.1. The Inclusion of Electron Correlation Effects

In the ONIOM(HF/6-31G(d):AMBER94)-ME approach the key reactive region, the QM-region, is described by the Hartree-Fock method. This method, however, lacks inclusion of electron correlation effects which can be important in describing bond making and breaking processes. Thus, relative energies were obtained at the higher ONIOM(MP2/6-31G(d)//HF/6-31G(d):AMBER94)-ME level of theory with inclusion of Gibbs corrections. That is, single-points were performed in which the key QM-region is now described using the conventional electron correlation approach MP2/6-31G(d). The resulting PES obtained is shown in Figure 7; dashed blue line. 

It can be seen clearly that a number of significant changes in relative energies occur along the catalytic pathway. In particular, those of the intermediates and product complex all decrease by 0.9–15.5 kJ mol^−1^ with respect to **RC** . However, the largest effects are observed for the transition structures (TS’s) which all decrease considerably by 23.3–94.9 kJ mol^−1^ with respect to **RC** . For example, the reaction barrier for the initial nucleophilic attack of the Glu-NH_2_ nitrogen at the R-group carbonyl carbon of AASA is significantly reduced by 42.5 kJ mol^−1^ to 60.0 kJ mol^−1^; namely is, the relative energy of **TS1** for formation of the ^Glu^N-C^AASA^ bond decreases. In fact, at this level of theory this reaction step now represents the rate-limiting step of the overall catalytic mechanism. 

The resulting “N-protonated” carbinolamine **I1** lies lower in energy than **RC** by –66.1 kJ mol^–1^, a modest lowering of 15.5 kJ mol^−1^ (cf. Figure 3). The subsequent proton transfer from the bridging -^Glu^NH_2_^+^- moiety to the Glu-COO^−^ group now occurs essentially without a barrier via **TS2** to give the “neutralized” carbinolamine intermediate **I2**. Therefore, **I1** has become kinetically and thermodynamically unstable with respect to rearrangement to **I2**. Indeed, re-optimization of **I1** at the ONIOM(MP2/6-31G(d)//HF/6-31G(d):AMBER94)-ME level of theory gave **I2** directly (not shown). Notably, **I2** is again the lowest energy intermediate along the catalytic pathway with respect to **RC** and now has a relative energy of −108.4 kJ mol^−1^. This corresponds to a marginal decrease of just 0.9 kJ mol^−1^, the smallest observed of any intermediate upon inclusion of electron correlation effects.

A large reduction in the calculated barrier for the subsequent double-proton transfer via **TS2** to give the alternate carbinolamine intermediate **I3** is also observed. Specifically, it has been reduced by 54.4 kJ mol^−1^. Consequently, similar to that observed for **I1** at the same level of theory, **I3** which now has a relative energy of −47.2 kJ mol^−1^ with regards to **RC** , is kinetically and thermodynamically unstable with respect to rearrangement back to **I2**. Indeed, as for **I1**, re-optimization of **I3** at the ONIOM(MP2/6-31G(d)//HF/6-31G(d):AMBER94)-ME level of theory directly gave **I2** (not shown).

The reaction barrier for inversion of the bridging -^Glu^NH-; namely, rearrangement of **I3** to give the alternate carbinolamine intermediate **I4** via **TS4**, is calculated to be just 14.7 kJ mol^−^^1^ with respect to **I3** (Figure 7). Notably, the resulting “inverted carbinolamine” intermediate **I4** is calculated to be only slightly stable with respect to rearrangement back to **I3** by 1.3 kJ mol^−^^1^. The subsequent loss of water via **TS5** is found to occur at a very low cost of 5.0 kJ mol^−^^1^ with respect to **I4** (Figure 7). Thus, while the energy of **TS4** relative to **RC** has decreased by 38.1 kJ mol^−^^1^ upon inclusion of electron correlation effects, this corresponds to a decrease in the actual reaction barrier height of just 2.1 kJ mol^−^^1^ (*cf.* Figure 3). Similar to that obtained at the lower ONIOM(HF/6-31G(d):AMBER94)-ME + Gibbs corrections level of theory, the resulting Schiff base intermediate **I5** is calculated to lie very low in energy relative to **RC** . Indeed, it has only modestly decreased by 10.4 kJ mol^−1^ to −103.3 kJ mol^−1^.

The largest impact of including the effects of electron correlation, however, are observed in the final step of the overall pathway; reduction of the Schiff base **I5** via hydride transfer from NADPH onto the intermediates ^AASA^C centre to give the desired saccharopine product. Specifically, as can be seen in Figure 7, the relative energy of **TS6** with respect to **RC** decreases by 94.9 kJ mol^−1^. As a result, the barrier for this final step is markedly reduced to 115.9 kJ mol^−1^ with respect to **I5** and in fact, is now predicted to be enzymatically and kinetically feasible [32]. The overall mechanism is again predicted to be exothermic with the final saccharopine-bound active-site complex **PC** lying lower in relative energy by 83.3 kJ mol^−1^ than the initial reactant-bound active-site complex **RC** (Figure 7).

#### 3.3.2. The Effects of the Protein’s Anisotropic Polar Environment

In the ONIOM(MP2/6-31G(d)//HF/6-31G(d):AMBER94)-ME approach above the surrounding protein environment and its effects on the reactive QM region are only treated at the molecular mechanics (MM) level of theory. In contrast, in an electronic embedding (EE) formalism the point charges of the MM protein environment are included in the self-consistent optimization of the wave function. Consequently, it enables one to examine the effects of polarization on the reactive region (QM layer) by the anisotropic protein environment. Thus, relative energies were then also obtained at the ONIOM(MP2/6-31G(d)//HF/6-31G(d):AMBER94)-EE + Gibbs corrections level of theory. The resulting PES obtained is shown in Figure 7; dotted pink line. Comparison with the PES obtained at the ONIOM(MP2/6-31G(d)//HF/6-31G(d):AMBER94)-ME + Gibbs corrections level of theory (Figure 7, dashed blue line) provides insight into the protein environment’s influence on the catalytic mechanism.

As can be seen in Figure 7, inclusion of the polarizing effects of the protein environment has a tremendous influence on the overall pathway. More specifically, the relative energy of almost all intermediates, transition structures and the product complex are now significantly raised with respect to **RC** by 27.7–161.4 kJ mol^−1^. The only exception occurs for the initial nucleophilic attack of Glu-NH_2_ at the ^AASA^C centre via **TS1** for which the barrier is instead reduced by 9.8 kJ mol^−1^ to 50.2 kJ mol^−1^. Importantly, as a result, this step is no longer rate-limiting in the overall pathway (see below).

Significant changes are also observed for the carbinolamine intermediates **I1**, **I2** and **I3** and the proton transfer reactions via **TS2** and **TS3** through which they interconvert. In particular, the initial “N-protonated” carbinolamine intermediate **I1** formed is now in fact slightly higher in energy than **RC** by 9.4 kJ mol^−1^. Furthermore, it is stable with respect to rearrangement to the subsequent “neutralised” carbinolamine **I2** (see below). In contrast, **I2** now has the highest relative energy with respect to **RC** , 53.0 kJ mol^−1^, of the three carbinolamine intermediates **I1**, **I2** and **I3**. In addition, it is thermodynamically and kinetically unstable with respect to rearrangement back to **I1** or to the subsequent “charged” carbinolamine **I3**. This is indicated by the fact that both **TS2** and **TS3** now have lower relative energies than **I2** of 24.5 and 30.0 kJ mol^−1^, respectively (Figure 7). The complex **I3** continues to lie lower in energy than **RC** , but by a lesser margin of 15.0 kJ mol^−1^. However, as a result it is now the lowest energy carbinolamine intermediate of all three and in fact, is the lowest energy intermediate obtained along the entire catalytic pathway. 

A possible explanation for these observed changes may be found by considering the substrate glutamate’s carboxylate and the active-site residues with which it interacts. In particular, in **I2** the Glu-COO^−^ group hydrogen bonds to the guanidinium of an arginyl (Arg224) and phenolic R-group of a tyrosyl (Tyr78). At the previous level of theory considered, ONIOM(MP2/6-31G(d)//HF/6-31G(d):AMBER94)-ME, the Glu-COO^−^…Arg224/Tyr78 interactions were modelled at the MM level and thus, in effect, were modelled as a steric interaction. However, by now considering the polarizing effects of the protein environment these interactions are preferred when the Glu–COO^-^ group is anionic as in **I2** and **I3**. Furthermore, the lower relative energy for **I3** may reflect that there is also preference for having the positive charge on the intermediate further removed from the carboxylate and the positively charged Arg224 residue; in **I1** the Glu-COO^−^ hydrogen bonds directly with the bridging -^Glu^NH_2_^+^- group while in **I3** it indirectly hydrogen bonds with the AASA-NH_3_^+^ group via the carbinolamine -OH moiety. In addition, the predicted instability of **I2** suggests that it may resemble a transition structure for Glu-COO^−^-assisted proton transfer from -^Glu^NH_2_^+^- to the carbinolamine hydroxyl oxygen, which would otherwise require an inherently high energy four-membered ring transition structure [7,29]. This is analogous to previous studies that have found lower barriers in related systems for a water-assisted proton transfer from the bridging -NH_2_^+^- to the carbinolamine oxygen [28,29,33].

Inversion of the bridging -^Glu^NH- moiety via **TS4** is calculated to have now a decidedly higher barrier of 49.4 kJ mol^−^^1^ with respect to **I3**. This corresponds to an increase of 34.7 kJ mol^−^^1^ compared to that obtained within the mechanical embedding formulism at the same level of theory (see above). Thus, the protein environment has a greater effect on this reaction step than does the inclusion of electron correlation which resulted in a comparatively slight increase of 2.1 kJ mol^−^^1^.

Considerable changes upon inclusion of the polarizing effects of the protein environment are also observed for the subsequent loss of water via **TS5** to give the Schiff base intermediate **I5** . In particular, the barrier for this step is now 51.5 kJ mol^−^^1^ with respect to **I4**, a ten-fold increase compared to that obtained using the ONIOM(MP2/6-31G(d)//HF/6-31G(d):AMBER94)-ME approach. In fact, this process now represents the overall rate-limiting step along the catalytic pathway. Furthermore, **I5** lies higher in energy than **RC** by 33.8 kJ mol^−^^1^ and importantly, is thermodynamically and kinetically unstable with respect to further reaction via **TS6** to give the final product complex **PC** (Figure 7). Therefore, hydride transfer from the NADPH cofactor to the ^AASA^C centre of the imine now essentially occurs without a barrier. The complex **PC** is calculated to be marginally endothermic compared to **RC** by 2.6 kJ mol^−^^1^.

#### 3.3.3. The Effects of Increasing the Basis Set Size

In any computational study it is important to use a basis set that adequately describes the chemical system being studied. This is particularly true when examining bond making and breaking process or those systems that involve weak, long-range or charged interactions. Thus, we also chose to examine the effects of increasing the basis set size for the reactive region, the QM layer. Specifically, the PES for the overall catalytic mechanism was obtained at the ONIOM(MP2/6-311G(d,p)//HF/6-31G(d): AMBER94)-EE + Gibbs corrections level of theory and is shown in Figure 7 (solid black line). This approach also represents the best, or benchmark, level of theory used in this present study.

One can clearly see that improving the basis set used for the QM layer from 6-31G(d) to 6-311G(d,p) does not change the overall reaction pathway. Indeed, for almost all of the intermediates, transition structures and product complex, their relative energy with respect to **RC** decreases by just −4.7–−37.5 kJ mol^−1^. The only exception is again observed for **TS1**, *i.e.*, nucleophilic attack of Glu-NH_2_ at the ^AASA^C centre, which instead increases by 7.5 kJ mol^−1^ to 57.7 kJ mol^−1^.

For example, the carbinolamine **I1** is again predicted to lie just slightly higher in energy than **RC** by 4.6 kJ mol^−1^. Similarly, **I3** is again calculated to lie lower in energy than **RC** , though now by 20.9 kJ mol^−1^, a 5.9 kJ mol^−1^ decrease (Figure 7). Furthermore, it is still the lowest energy intermediate along the overall pathway. In addition, despite a decrease in its relative energy of 17.0 kJ mol^−1^ to 36.0 kJ mol^−1^, **I2** is still predicted to be thermodynamically and kinetically unstable with respect to rearrangement back to **I1** or on to **I3**.

A marginal increase in the barrier of just 1.2 kJ mol^−1^ to 50.6 kJ mol^−1^ is observed for inversion of the bridging -^Glu^NH- moiety via **TS4**, *i.e.*, interconversion of **I3** and **I4**. In contrast, there is a modest reduction in the subsequent barrier height for loss of the carbinolamine -OH group as water via **TS5**. Specifically, the barrier is reduced by 7.4 kJ mol^−1^ to 74.1 kJ mol^−1^ with respect to **RC** . However, this reaction process again remains the overall rate-limiting step in the catalytic mechanism of saccharopine reductase. It is noted that despite this relative decrease in the barrier height of **TS5**, the energy difference between **I3** and **TS5**, the lowest energy intermediate and highest barrier respectively, remains fairly constant upon increasing the basis sets size. Indeed, this difference decreases by just 1.5 kJ mol^−1^ to 95.0 kJ mol^−1^ (Figure 7).

The resulting Schiff base intermediate **I5** now lies 17.2 kJ mol^−1^ higher in energy than **RC** , but again can essentially undergo a barrierless hydride transfer from the NADPH cofactor to the its ^AASA^C centre to give the final saccharopine-bound active-site product complex (**PC**). It is noted that upon increasing the basis set size the overall mechanism has once again become slightly exothermic with PC lying 18.4 kJ mol^−1^ lower in energy than the initial reactant-bound active-site complex **RC** .

It is interesting to note that we also used the first principles quantum and statistical mechanics approach outlined by Llano and Eriksson [34], in combination with small chemical models consisting of only the Schiff base itself in **I5** , *i.e.*, no active-site, and the NADPH ring from which the hydride is donated. It was predicted that the inherent free energy change for hydride transfer favoured the reduced imine product by 33.3 kJ mol^−1^. Within the active-site, at our presently used highest level of theory ONIOM(MP2/6-311G(d,p)//HF/6-31G(d):AMBER94)-EE + Gibbs corrections, **PC** lies 35.6 kJ mol^−1^ lower in free energy than **I5** . This suggests that for this final mechanistic step the enzyme does not aim to target distinctly or favour binding of the product over the preceding Schiff base. Rather, it simply utilises the inherent favourable free energy change for the hydride transfer.

## 4. Conclusions

A series of systematically higher level ONIOM-based computational methods have been used in order to examine the overall catalytic mechanism of saccharopine reductase and the effects of electron correlation and the anisotropic polar protein environment on the mechanism.

The enzymes overall mechanism was elucidated using the quantum mechanics/molecular mechanics (QM/MM) ONIOM(HF/6-31G(d):AMBER94) method within the mechanical embedding (ME) formulism. The present results suggest that the catalytic mechanism does not require the direct involvement of active-site residues in the required proton transfer processes. For example, the protonated α-amine (AASA-NH_3_^+^) of the cosubstrate α-aminoadipate-δ-semialdehyde (AASA) is able to act as an acid. Specifically, during nucleophilic attack of the Glu-NH_2_ group at the R-group aldehyde carbon of AASA it protonates the forming oxyanion centre. In addition, the glutamate’s carboxylate (Glu-COO^−^) is able to assist the proton transfer from the bridging -^Glu^NH_2_^+^- moiety in the formed initial carbinolamine intermediate (**I1**) to the nearby carbinolamine hydroxyl oxygen. Notably, at the ONIOM(HF/6-31G(d):AMBER94)-ME + Gibb’s corrections level of theory the lowest energy intermediate along the overall pathway is calculated to be the “neutralised” carbinolamine intermediate **I2** in which both the α-carboxylate and α-amino of the initial glutamate and AASA cosubstrates respectively are neutral. The overall rate-limiting step was calculated to be hydride transfer via **TS6** from the NADPH cofactor onto the bridging -NH^+^=C- carbon centre of the Schiff base intermediate **I5** to give the final product bound active-site complex **PC**. In particular, it lies 107.5 kJ mol^−1^ higher in energy with regards to the initial reactant complex **RC**, but is 200.4 kJ mol^−1^ higher in energy than the preceding Schiff base intermediate **I5** (Figure 3).

The inclusion of electron correlation effects, on the key reactive region (QM layer) by increasing the level of theory to ONIOM(MP2/6-31G(d)//HF/6-31G(d):AMBER94)-ME + Gibb’s corrections, leads to considerable changes along the catalytic pathway. In particular, with respect to the initial reactant complex **RC** the relative energies of the mechanistic intermediates and product complex all decreased by 0.9–15.5 kJ mol^−1^ while those of the transition structures (TS’s) all decreased by 23.3–94.9 kJ mol^−1^. As a result, while the “neutralised” carbinolamine **I2** remains the lowest energy intermediate along the pathway (−108.4 kJ mol^−1^), the thermodynamic rate-limiting step is now nucleophilic attack of GluNH_2_ at the R-group aldehyde carbon (^AASA^C) of the AASA cosubstrate via **TS1** at a cost of 60.0 kJ mol^−1^ (Figure 7). The largest single reaction step barrier again occurs for reduction of the Schiff base intermediate via **TS6**, though now greatly reduced at 115.9 kJ mol^−1^.

Re-examination of the PES at the ONIOM(MP2/6-31G(d)//HF/6-31G(d):AMBER94) within the electronic embedding (EE) formulism with inclusion of Gibb’s corrections enabled the effects of the polarizing protein environment on the reactive region (QM layer) to be investigated. Importantly, it was found that relative to the initial reactant complex **RC** almost all intermediates, transition structures and product complex were destabilized by 27.7–161.4 kJ mol^−1^; namely, their relative energy was raised. The only exception occurred for the initial nucleophilic attack of Glu-NH_2_ on the AASA cosubstrate via **TS1** for which the barrier decreased by 9.8 kJ mol^−1^. Consequently, the carbinolamine **I3** lying 15.0 kJ mol^−1^ lower in energy than **RC** was now found to be the lowest energy intermediate along the overall pathway. Furthermore, the rate limiting step is now loss of water from the “inverted” carbinolamine **I4** via **TS5**, at a cost of 81.5 kJ mol^−1^ with respect to RC, to give the Schiff base **I5**. In addition, the subsequent and final step in the overall pathway, reduction of the Schiff base, is found to occur now essentially without a barrier.

Increasing the size of the basis set used to describe the key QM layer from 6-31G(d) to 6-311G(d,p) was also considered by the use of the ONIOM(MP2/6-311G(d,p)//HF/6-31G(d):AMBER94)-EE method with inclusion of Gibb’s corrections. In general, only comparatively modest decreases of 4.7–37.5 kJ mol^−1^ in the relative energy of all intermediates, transition structures and product complex with respect to **RC** were observed. The only exception being for **TS1** whose relative energy increased by 7.5 kJ mol^−1^. This was also the highest level of theory used in this present study. The carbinoalamine **I3** is again the the lowest energy intermediate along the catalytic pathway being 20.9 kJ mol^−1^ lower in energy than RC. The overall rate-limiting step is the loss of water to give the Schiff base intermediate **I5** which occurs via **TS6** at a cost of 74.1 kJ mol^−1^ with respect to RC. A subsequent barrierless hydride transfer reduces **I5** to the final saccharopine product.

Experimentally, it has been suggested that two active site acid/base residues with p*K*_a_’s of 5.6–5.7 and 7.8–8.0 facilitate the mechanistically required proton transfers [1]. However, it has also been experimentally suggested that there are no active site acid/base residues available and thus, the enzyme functions by orientating and positioning the substrates for reaction [4]. The present results suggest that acid/base functional groups within the substrates themselves, specifically the α-amine of α-aminoadipate-δ-semialdehyde and α-carboxylate of glutamate, are able to catalyse the mechanistically required proton transfer reactions, in support of previous suggestions by Johansson *et al.* [4] In addition, it is also suggested that the two p*K*_a_ values experimentally measured by Vashishtha *et al.* [1] may in fact correspond to these two substrate functional groups. That is, based on the extensive and high-level computational models used herein, the present results suggest that saccharopine reductase catalyses the overall reaction by binding the three required reactant molecules glutamate, α-aminoadipate-δ-semialdehyde and NADPH in an orientation and polar environment conducive to reaction.

## Supplementary Materials

Supplementary File 1
