# Supplementary material for: A QM/MM–Based Computational Investigation on the Catalytic Mechanism of Saccharopine Reductase
Source: Molecules. 2011 Oct 12;16(10):8569–89. doi: 10.3390/molecules16108569 (PMC6264447; doi:10.3390/molecules16108569)
Supplement: Supplementary File 1 [file molecules-16-08569-s001.pdf]

Supporting Information

Table S1. Cartesian x,y,z coordinates of the optimized structures noted in the manuscript for the small model DFT study.

System: Neutral

RC_N

N	-0.91794200	1.60595200	0.40626900
H	-0.17496400	1.16994700	-0.22329400
H	-0.87441600	1.04864500	1.24629300
H	-0.56821400	2.52066200	0.63606200
C	-2.27189400	1.59975800	-0.18969200
H	-2.35439300	2.49062200	-0.79797200
C	-2.53553700	0.37644200	-1.06938400
H	-1.79576000	0.35009900	-1.86310600
H	-3.48967900	0.57622200	-1.54852000
C	-2.62959800	-1.00711900	-0.39139300
H	-3.31037600	-1.59826100	-0.99456200
H	-3.09207000	-0.91840000	0.58640700
C	-1.32539800	-1.80889000	-0.27494800
H	-0.68379700	-1.66555800	-1.13720800
H	-1.55702200	-2.87363500	-0.24576200
C	-0.49268300	-1.54994800	0.95133700
H	0.46352000	-2.06892100	0.98510300
O	-0.83838600	-0.85387400	1.86210800
N	3.03054000	-1.38411800	-0.53908700
C	3.24602600	0.00670600	-0.17313700
H	3.72250200	0.06211300	0.79679400
C	1.93302700	0.77639800	-0.09848300
O	1.86912800	1.76214400	0.62163200
O	0.98509400	0.34426600	-0.81669400
H	3.88528300	0.55015100	-0.87362400
H	-2.99829200	1.67266000	0.60966300
H	2.46658100	-1.41665800	-1.36521000
H	3.90256900	-1.83288700	-0.74351200

$TS1_N$

N	0.61137800	-1.74024400	0.48990700
H	0.03489700	-1.75547000	-0.35548000
H	0.34011200	-0.82854700	0.95130000
H	0.32486500	-2.50578700	1.07517100
C	2.05629600	-1.72958700	0.19380900
H	2.30466500	-2.65899000	-0.30294800
C	2.44882300	-0.54629600	-0.69236100
H	1.82778200	-0.55521700	-1.58303600
H	3.45851000	-0.76261900	-1.02827900
C	2.44847100	0.86204300	-0.04637400
H	3.32893100	1.37416700	-0.42093000
H	2.57475100	0.78392400	1.02731100
C	1.24565400	1.77221400	-0.35651600
H	0.95152000	1.61579900	-1.38976800
H	1.58382300	2.80360000	-0.30110300
C	0.04226100	1.62523900	0.61856800
H	-0.15715400	2.58464200	1.10253100

O	0.02623100	0.62407100	1.41229100
N	-1.31477500	1.59713100	-0.38938900
C	-2.38205000	0.69439700	0.09356500
H	-2.45665800	0.75451500	1.16549300
C	-1.99758600	-0.73586300	-0.33118400
O	-2.44590700	-1.65358100	0.32385400
O	-1.19668100	-0.78430900	-1.29665700
H	-3.32366200	0.97914400	-0.36224000
H	2.58619700	-1.69838100	1.13725400
H	-1.04734800	1.19518800	-1.27568200
H	-1.64780400	2.53349300	-0.53617300

$IM1_N$

N	-1.02748800	1.62861800	0.39014400
H	-0.57693700	1.72748100	-0.50283200
H	-0.26990800	0.20143100	1.15778100
H	-0.76070300	2.43097500	0.92909400
C	-2.47916300	1.51740200	0.29927700
H	-2.93554500	2.41529800	-0.11526200
C	-2.94308500	0.31937000	-0.53348100
H	-2.70426000	0.49077100	-1.58100000
H	-4.02800700	0.29886100	-0.47390700
C	-2.40119500	-1.05957200	-0.11273500
H	-3.07698400	-1.81412900	-0.50058900
H	-2.43435700	-1.15086200	0.96795900
C	-0.97758600	-1.35641900	-0.64010700
H	-0.65085200	-0.54695100	-1.27717400
H	-0.98474800	-2.25261600	-1.25559400
C	0.08102300	-1.55374600	0.44992300
H	0.02540800	-2.54581400	0.87681300
O	0.04420000	-0.66824500	1.46894100
N	1.46166400	-1.51479300	-0.25164700
C	2.47655800	-0.56689900	0.31417800
H	2.41029200	-0.54425400	1.38603200
C	2.18347500	0.82515500	-0.32417900
O	2.69550000	1.77100800	0.22691600
O	1.45462500	0.74706900	-1.33344200
H	3.45333400	-0.91633500	0.00557700
H	-2.85856800	1.41048000	1.31096200
H	1.33107100	-1.10480400	-1.17694900
H	1.82677300	-2.44671800	-0.33697400

$TS2_N$

N	-1.59707600	1.82335400	-0.03114300
H	-1.51404200	2.00427000	-1.01428700
H	-0.41491400	0.60657100	0.77288100
H	-1.36954800	2.68292800	0.43289000
C	-2.94823800	1.38988100	0.32052100
H	-3.69292000	2.14598100	0.07548200
C	-3.35276200	0.08170000	-0.36153200
H	-3.32017800	0.21204900	-1.44236800
H	-4.39745400	-0.08770600	-0.11487900
C	-2.56418900	-1.18153500	0.02479700
H	-3.15943300	-2.03684900	-0.27687400
H	-2.48399200	-1.23608600	1.10591400

C	-1.16339700	-1.31108500	-0.61558600
H	-1.01821400	-0.53727500	-1.36139500
H	-1.08465700	-2.25839600	-1.14295700
C	-0.01300000	-1.23552600	0.38940900
H	0.01788900	-2.12895800	1.00131900
O	-0.07180700	-0.16694900	1.23702800
N	1.26339900	-1.17340800	-0.37899800
C	2.50384900	-0.85598800	0.37549900
H	2.31625600	-0.82544600	1.43578600
C	2.90697000	0.55204000	-0.15491000
O	3.87799100	1.08518600	0.30574200
O	2.09691400	0.92870700	-1.05177600
H	3.27512800	-1.58099400	0.15916900
H	-2.97579900	1.25597400	1.39674700
H	1.34785200	-0.19569500	-0.99012700
H	1.36957300	-1.98247700	-0.96345100

$IM2_N$

N	1.75181300	1.82582500	0.37340700
H	1.94931800	1.90772100	1.35348500
H	0.35147000	0.72623500	-0.41901600
H	1.48765900	2.74115200	0.05935300
C	2.93547700	1.36311100	-0.35259000
H	3.76686500	2.05938300	-0.24692600
C	3.40901700	-0.01969200	0.09962400
H	3.57607200	-0.00790200	1.17631700
H	4.38653800	-0.17596800	-0.34951900
C	2.52775000	-1.22416600	-0.27052700
H	3.13428800	-2.11319600	-0.12843100
H	2.30154000	-1.18223800	-1.33174900
C	1.22048700	-1.38633800	0.53598000
H	1.20341900	-0.70917900	1.38287000
H	1.16952400	-2.39043300	0.95014500
C	-0.04626800	-1.15653400	-0.28696400
H	-0.11992300	-1.92269800	-1.05760100
O	-0.05107100	0.04840200	-0.96485300
N	-1.20878300	-1.21140600	0.59334500
C	-2.47644500	-0.98291100	-0.07942300
H	-2.47656800	-1.28174800	-1.12134700
C	-2.91113400	0.47571400	-0.02299800
O	-3.76690400	0.90894000	-0.71804100
O	-2.31301300	1.20064800	0.90788800
H	-3.25556200	-1.54687100	0.42279200
H	2.68030900	1.33130900	-1.40604100
H	-1.22368100	-2.07413000	1.09921900
H	-1.63787000	0.65862400	1.31062100

$TS3_N$

N	-2.15326300	1.77783500	-0.37410500
H	-2.25651600	1.80691300	-1.37166200
H	-0.65117900	0.84334100	0.42528000
H	-2.04421800	2.72826000	-0.07246400
C	-3.33773400	1.18442700	0.24786700
H	-4.23919800	1.75146900	0.01838100
C	-3.56153500	-0.26986100	-0.16753700

H	-3.67520100	-0.32520200	-1.24949300
H	-4.52136000	-0.56669500	0.24710800
C	-2.50527200	-1.28672000	0.29561900
H	-2.96284300	-2.26965000	0.25065100
H	-2.28239900	-1.10932900	1.34336300
C	-1.19211800	-1.31954100	-0.51991100
H	-1.24447900	-0.64570100	-1.36855900
H	-1.04595000	-2.31483200	-0.93097100
C	0.04964800	-0.96175800	0.29453800
H	0.17311300	-1.68616200	1.09841800
O	-0.05662900	0.27657100	0.92235000
N	1.21170900	-0.95386400	-0.57210300
C	2.50819000	-0.94543400	0.08166100
H	2.42405500	-1.17592700	1.14077700
C	3.24640500	0.38520400	-0.01578900
O	4.41920500	0.45507300	0.14981000
O	2.50591700	1.44582200	-0.27047300
H	3.16726300	-1.68696200	-0.34971000
H	-3.19837400	1.23266900	1.32254100
H	1.16249300	-1.67152500	-1.26379500
H	1.59222300	1.18390700	-0.32860400

$IM3_N$

N	2.12458500	1.72510200	0.57795100
H	2.23085100	1.63756100	1.57167300
H	0.62656800	0.88168400	-0.27388100
H	2.00983200	2.70371700	0.39014200
C	3.30615100	1.21165900	-0.11608800
H	4.20590000	1.75891500	0.16266700
C	3.54693000	-0.27633000	0.14027200
H	3.67924200	-0.44351500	1.20852700
H	4.50191800	-0.51918300	-0.31830300
C	2.49179200	-1.25096900	-0.40875700
H	2.95798300	-2.22913100	-0.46711800
H	2.25245700	-0.97293000	-1.43054700
C	1.19201800	-1.37706700	0.41861900
H	1.25906900	-0.80325400	1.33677500
H	1.05122300	-2.41244900	0.71667600
C	-0.06748700	-0.93434300	-0.32375500
H	-0.21999200	-1.57375200	-1.19211000
O	0.05490300	0.35921200	-0.84637600
N	-1.20128000	-0.99149000	0.56918300
C	-2.52448500	-0.97408500	-0.03255500
H	-2.49572200	-1.28528300	-1.07750300
C	-3.20992100	0.38659000	0.01762200
O	-4.36303700	0.49109400	0.28038000
O	-2.46886700	1.43239800	-0.29048600
H	-3.18061300	-1.65618800	0.48804100
H	3.15388900	1.37244100	-1.17802400
H	-1.57022400	1.17750300	-0.50377600
H	-1.11950200	-1.74578300	1.21652400

$TS4_N$

N	2.23084600	1.69842900	0.65585700
H	2.39946200	1.58383900	1.63817700

H	0.67076500	0.93994700	-0.22063300
H	2.13716300	2.68457600	0.49786600
C	3.35058100	1.16734500	-0.12296400
H	4.28214400	1.68071700	0.11264800
C	3.56134000	-0.33293300	0.08473100
H	3.72346500	-0.53230400	1.14354300
H	4.49524200	-0.58627000	-0.41022800
C	2.46947400	-1.27023100	-0.45701400
H	2.90833800	-2.25987600	-0.53332500
H	2.22188500	-0.97341100	-1.47122000
C	1.17859800	-1.37566000	0.38618600
H	1.29136500	-0.83551100	1.32203300
H	0.99572500	-2.41172500	0.65205400
C	-0.07642200	-0.86119400	-0.31706000
H	-0.26877100	-1.46164000	-1.20109900
O	0.10171700	0.44525300	-0.81631300
N	-1.19810600	-0.94184100	0.55921200
C	-2.55078500	-0.98928800	0.08364600
H	-2.58133400	-1.42119500	-0.91542800
C	-3.26371000	0.35975100	0.00206300
O	-4.43051200	0.45588900	0.20147200
O	-2.52684000	1.39805500	-0.34003600
H	-3.16077600	-1.61891800	0.71677700
H	3.13878000	1.35919100	-1.16924800
H	-1.61134200	1.15873700	-0.49488000
H	-1.04066000	-1.18259100	1.50897300

$IM4_N$

N	2.22096300	1.70426500	0.60240500
H	2.35909400	1.60799100	1.59160200
H	0.68168000	0.90950800	-0.23053000
H	2.12864900	2.68726200	0.42458800
C	3.36962200	1.16553800	-0.12879900
H	4.29245000	1.67744000	0.14086400
C	3.56206400	-0.33469100	0.09242700
H	3.71146500	-0.52935700	1.15377000
H	4.49726900	-0.60211500	-0.39238500
C	2.45746400	-1.25455600	-0.45350200
H	2.88649600	-2.24579100	-0.55781100
H	2.20001600	-0.93486500	-1.45887600
C	1.17661300	-1.36984900	0.40575000
H	1.28681300	-0.81189400	1.33194000
H	1.01161500	-2.40041100	0.69511000
C	-0.09516900	-0.89688600	-0.29506200
H	-0.26689200	-1.49888200	-1.17876200
O	0.04029400	0.42951500	-0.76337800
N	-1.22198500	-1.09231700	0.56816600
C	-2.56449900	-0.99557000	0.03755900
H	-2.56480400	-1.38931900	-0.97460500
C	-3.22859800	0.38275100	-0.00872400
O	-4.38948300	0.50393200	0.20876000
O	-2.47065000	1.41635900	-0.31800400
H	-3.23149300	-1.61894000	0.61703800
H	3.19950100	1.35355700	-1.18349300
H	-1.57109500	1.16078900	-0.53740700

H	-1.11988000	-0.62748100	1.44805100

TS5$_N$

[INSERT COORDINATES FOR THE TS FOR THE LEAVING OF THE WATER HERE]

IM5$_N$

N	-2.93170300	-1.67407700	0.13558300
H	-3.42605300	-1.71677900	1.00722700
H	-2.99733300	-2.58537200	-0.27575700
C	-3.52896200	-0.66415900	-0.73721200
H	-4.61694200	-0.70706600	-0.73589400
C	-3.06891900	0.73298600	-0.32018900
H	-3.24914700	0.84505100	0.74666700
H	-3.67921000	1.48355400	-0.81436500
C	-1.58295500	0.95844300	-0.63375400
H	-1.47263100	1.35176200	-1.64043500
H	-1.06662300	0.00958500	-0.61075300
C	-0.89577200	1.90155800	0.37502300
H	-1.09439200	1.55177100	1.38115900
H	-1.27347400	2.91391500	0.28961500
C	0.58801200	1.89669100	0.15670800
H	1.06289900	2.67803100	-0.41242200
N	1.31125200	0.95647400	0.57574600
C	2.73030600	0.72809600	0.27648700
H	3.09554500	1.53215700	-0.34507900
C	2.90970900	-0.66087400	-0.43191500
O	4.00110500	-0.79987500	-0.93535100
O	1.92118400	-1.40075300	-0.35533500
H	3.27322900	0.72828300	1.21263800
H	-3.20678500	-0.86575500	-1.75319600
H	0.84183400	0.18116600	1.04214700
H	0.57988500	-1.72995800	0.94393900
O	-0.09830100	-1.35788400	1.51524300
H	-0.92806000	-1.53547100	1.07787000

PC$_N$

N	-1.33434300	1.79875200	-0.71520100
H	-1.07659500	2.76666000	-0.68884300
H	-0.45110300	1.32124000	-0.64821100
C	-2.21445800	1.50778900	0.40777200
H	-2.87491700	2.36386200	0.52752300
C	-3.11534400	0.28456400	0.18043000
H	-3.48247000	0.33715900	-0.84146300
H	-3.98507000	0.38784000	0.83087900
C	-2.52797200	-1.11237500	0.43926000
H	-3.33267200	-1.82710200	0.27358700
H	-2.27201600	-1.20033800	1.49493400
C	-1.30970100	-1.51075500	-0.41503100
H	-1.31751400	-0.94283700	-1.34075800
H	-1.37721000	-2.55803700	-0.69882200
C	0.02213900	-1.29412500	0.30331600
H	0.10802100	-2.02546100	1.10687800
N	1.14486100	-1.46757300	-0.59844300
C	2.39190900	-0.99911900	-0.00886600
H	2.51827800	-1.43802700	0.97660100

C	2.54118800	0.54330900	0.10211800
O	3.51690200	0.92414200	0.74325600
O	1.68165400	1.21480800	-0.49290400
H	3.22369400	-1.36187300	-0.60965600
H	-1.68383000	1.39989200	1.35629400
H	0.98411000	-0.85406600	-1.37585100
H	0.03066000	-0.31450600	0.77559900

System: Protonated

RC_P

N	-0.91717800	1.59333100	0.93500400
H	0.06256400	1.30143700	0.86681800
H	-1.43364300	0.81063100	1.32872600
H	-0.95170900	2.36766900	1.57908900
C	-1.43219700	1.96504900	-0.42520600
H	-0.63339400	2.50114900	-0.91585600
C	-1.83856400	0.71580000	-1.20185100
H	-1.04140300	-0.01662900	-1.12163100
H	-1.88277400	0.99180400	-2.24872700
C	-3.20105000	0.12427700	-0.76318700
H	-3.93975500	0.33894700	-1.52553200
H	-3.55890200	0.61426700	0.13755300
C	-3.18771300	-1.38273000	-0.51374500
H	-2.74836400	-1.92774700	-1.34721200
H	-4.20251700	-1.76498900	-0.42292300
C	-2.45887600	-1.78503100	0.73909100
H	-2.43632100	-2.85171200	0.96153400
O	-1.92857300	-1.01544300	1.48656500
N	4.43077400	-1.06756500	-0.53215500
H	5.04743100	-0.82677800	-1.28156300
H	4.90566100	-1.71929500	0.05917700
C	3.94983200	0.09490100	0.19131200
H	4.20730900	0.06778600	1.24316100
C	2.43434800	0.19259900	0.11037100
O	1.82382700	1.07938000	0.64052800
O	1.83346900	-0.74286100	-0.57156600
H	-2.26712600	2.63751800	-0.28886100
H	4.34670400	1.02303700	-0.20170600
H	2.50659400	-1.34525700	-0.90691000

$TS1_P$

N	-2.09600500	1.91128700	-0.18556700
H	-2.16344000	2.10554500	-1.17275400
H	-1.22720900	1.36952400	0.00966400
H	-2.01561900	2.79824300	0.28945400
C	-3.27654000	1.13181000	0.31143200
H	-4.14623900	1.75520900	0.16250400
C	-3.45079500	-0.20352200	-0.41371100
H	-3.36191900	-0.05194000	-1.48752600
H	-4.48547700	-0.48202900	-0.24678300
C	-2.59282400	-1.40492100	0.02664200
H	-3.09969100	-2.28213200	-0.35626500
H	-2.62737000	-1.49778800	1.10919100
C	-1.12741600	-1.48409700	-0.46604000

H	-1.02047800	-0.99199200	-1.42885000
H	-0.87808400	-2.52908100	-0.60947200
C	-0.15816300	-0.86917600	0.52463700
H	0.02247100	-1.48153700	1.40331300
O	-0.13970900	0.37873400	0.67965600
N	1.50107100	-1.37310600	-0.24731600
H	1.39302600	1.18275100	0.28571100
H	1.52928000	-1.10033500	-1.21603200
H	1.54448300	-2.37788100	-0.24445300
C	2.68505400	-0.84204300	0.47534200
H	2.42659100	-0.74692500	1.52374200
C	3.13799900	0.50966200	-0.07218100
O	4.25043600	0.68183000	-0.42289900
O	2.21108700	1.44646800	-0.13940800
H	-3.12436400	0.99846700	1.37278200
H	3.52018500	-1.51789900	0.38044200

$IM1_P$

N	-2.13106400	1.91602600	-0.05565800
H	-2.23069000	2.18398000	-1.02213400
H	-1.23659400	1.34993700	0.06301100
H	-2.03658200	2.76485000	0.48078400
C	-3.29344300	1.10225900	0.41019500
H	-4.18009400	1.70686900	0.28294800
C	-3.43209000	-0.21274200	-0.35766700
H	-3.33889000	-0.02715000	-1.42563100
H	-4.46050400	-0.52173900	-0.20718900
C	-2.54616700	-1.40354500	0.05958800
H	-3.07130500	-2.28866600	-0.27835700
H	-2.52294000	-1.47649100	1.14417700
C	-1.11063500	-1.50530500	-0.50125500
H	-1.06201000	-1.08605100	-1.50409100
H	-0.87941100	-2.56374800	-0.58958200
C	-0.05193400	-0.83170700	0.37128800
H	-0.04895300	-1.28353200	1.36532600
O	-0.04120200	0.48197900	0.37212900
N	1.35111300	-1.31413600	-0.18277700
H	1.39388300	1.20019100	-0.17260700
H	1.41355900	-1.03060400	-1.14968200
H	1.38844200	-2.32119700	-0.18271600
C	2.53156600	-0.78565500	0.58058700
H	2.20172300	-0.57289900	1.58786600
C	3.14286700	0.46967700	-0.04821800
O	4.31507200	0.57502300	-0.11229900
O	2.29315100	1.36362800	-0.49516100
H	-3.14535100	0.93233800	1.46744600
H	3.29822700	-1.54224300	0.60503200

$TS2_P$

N	-2.38019800	-1.91981900	-0.46729300
H	-2.08987700	-2.52508000	0.28766700
H	-1.54211800	-1.40924100	-0.74999800
H	-2.68012400	-2.51603400	-1.22482600
C	-3.47386100	-0.97902500	-0.00914600
H	-4.14468700	-1.56952400	0.59685400

C	-2.89171800	0.20217200	0.76477200
H	-2.15433500	-0.16442600	1.47118400
H	-3.69998000	0.59868600	1.36714200
C	-2.31149200	1.33650800	-0.12084100
H	-2.96070900	2.19768400	-0.03122200
H	-2.35292800	1.05771100	-1.17181600
C	-0.87668600	1.77556800	0.22285000
H	-0.70060100	1.69344100	1.29160600
H	-0.75171400	2.82221400	-0.03786800
C	0.16998800	0.96963600	-0.53639300
H	0.11499100	1.18441600	-1.59527000
O	-0.02461200	-0.41052800	-0.39356300
N	1.52559400	1.29551800	-0.05004400
H	0.52860800	-0.75547100	0.31875900
H	1.67609800	0.68379200	0.93436500
H	1.66479000	2.28763800	0.01996300
C	2.65609400	0.57655300	-0.70614100
H	2.40773400	0.26121300	-1.70812500
C	2.89442600	-0.63921300	0.23361300
O	3.67384900	-1.48409600	-0.06450800
O	2.11877400	-0.54129200	1.25685300
H	-4.00178900	-0.66084100	-0.89597800
H	3.52427200	1.21806100	-0.73361700

$IM2_P$

N	2.55839600	1.58065600	0.80557300
H	2.75697200	1.25329600	1.73881800
H	1.58847800	1.33003100	0.55784200
H	2.62412500	2.58765200	0.82171400
C	3.50546800	0.99123400	-0.20510400
H	4.48229500	1.39685200	0.01583600
C	3.53677900	-0.53413100	-0.16869600
H	3.78361600	-0.87123200	0.83587900
H	4.38659200	-0.80788100	-0.78494500
C	2.30503200	-1.28810200	-0.70040400
H	2.64658500	-2.28756500	-0.93926800
H	1.99575700	-0.84800700	-1.64308300
C	1.09152500	-1.41327200	0.25122700
H	1.35518700	-1.11718100	1.26602000
H	0.79823200	-2.45456000	0.31605900
C	-0.14216800	-0.62730000	-0.17493400
H	-0.46192000	-0.95727900	-1.15793900
O	0.24507100	0.73734400	-0.30411600
N	-1.21418800	-0.76409300	0.75985300
H	-0.99191900	-1.32542300	1.55207700
C	-2.55869800	-1.00008300	0.28330600
H	-2.58799400	-1.64110200	-0.59748100
C	-3.33076200	0.25381100	-0.07359900
O	-2.56454800	1.23903000	-0.56638500
O	-4.50366300	0.35598500	0.01349100
H	3.18082300	1.36213600	-1.16627500
H	-3.13302300	-1.49485200	1.05214300
H	-0.55143100	1.24795400	-0.42524300
H	-3.13977200	1.95940300	-0.81484200

$TS3_P$

N	2.55644300	1.62953600	0.73704000
H	2.74266200	1.35725400	1.69028200
H	1.59249400	1.35410100	0.48894700
H	2.61150800	2.63649800	0.69751600
C	3.52848200	0.99515600	-0.22082000
H	4.49876400	1.41508200	0.00233900
C	3.56097000	-0.52626000	-0.10823200
H	3.78190400	-0.81343100	0.91765200
H	4.42680800	-0.82782300	-0.68808600
C	2.34317100	-1.30590300	-0.63539400
H	2.69492600	-2.31040700	-0.83507900
H	2.04995500	-0.89975900	-1.59854600
C	1.11039600	-1.41084600	0.29468400
H	1.34746800	-1.07200400	1.30288600
H	0.82710300	-2.45129700	0.39369300
C	-0.12457700	-0.66167300	-0.19040800
H	-0.39152600	-1.00269300	-1.18413400
O	0.24085700	0.71815700	-0.31266700
N	-1.23450700	-0.83947900	0.66619300
H	-1.06005300	-1.10042600	1.60898600
C	-2.57601900	-1.01903500	0.18650700
H	-2.58923600	-1.57107600	-0.75098300
C	-3.35615900	0.25971200	-0.06030200
O	-2.59657900	1.27098700	-0.51120600
O	-4.52468300	0.36381100	0.07292600
H	3.22583300	1.31683000	-1.20668100
H	-3.15136500	-1.59538600	0.89596500
H	-0.56209900	1.21463100	-0.44958200
H	-3.17305100	2.00895900	-0.69644600

$IM3_P$

N	2.53833000	1.64964500	0.68725400
H	2.71288400	1.40148300	1.64940100
H	1.58168300	1.35705800	0.43690400
H	2.58177500	2.65610500	0.62562600
C	3.53685400	1.00562300	-0.23750400
H	4.49848700	1.43699400	0.00076300
C	3.57338100	-0.51296500	-0.09811700
H	3.79192200	-0.78218700	0.93296300
H	4.44102200	-0.82226400	-0.67118300
C	2.35348400	-1.29640200	-0.61420000
H	2.70443400	-2.29999000	-0.81951800
H	2.05213200	-0.88935800	-1.57504200
C	1.12879300	-1.40413600	0.32737700
H	1.35635100	-1.02325100	1.32260200
H	0.87140100	-2.44508700	0.46734200
C	-0.12879300	-0.70889000	-0.18139700
H	-0.36537900	-1.07000900	-1.17475800
O	0.18523600	0.68309400	-0.28256300
N	-1.24433200	-0.98317500	0.65302800
H	-1.15839400	-0.64490100	1.58823600
C	-2.59263700	-1.02742900	0.15101400
H	-2.59989400	-1.54742900	-0.80164100
C	-3.33882800	0.27937300	-0.06890900

O	-2.55437200	1.31022600	-0.42820900
O	-4.51038600	0.39081100	0.01898500
H	3.25517600	1.30847800	-1.23559700
H	-3.20827800	-1.60982600	0.82179400
H	-0.61249900	1.16149700	-0.49266900
H	-3.11816500	2.06329400	-0.59248100

$TS4_P$

N	2.44140000	1.69218200	0.46163100
H	2.48921900	1.63095000	1.46387500
H	0.96717700	1.12493400	-0.21659500
H	2.49224000	2.67398600	0.25254700
C	3.60382900	1.01082700	-0.13684500
H	4.53241700	1.42699500	0.24084700
C	3.59766400	-0.49704300	0.10586900
H	3.67982800	-0.70459300	1.17066100
H	4.50532200	-0.88720800	-0.34144400
C	2.39740900	-1.25388000	-0.48967500
H	2.71145700	-2.26289400	-0.72549000
H	2.12657300	-0.80015700	-1.43787900
C	1.15721700	-1.34860500	0.43270800
H	1.27225800	-0.75025900	1.32914900
H	1.02187700	-2.37211200	0.76664800
C	-0.14555900	-0.97041200	-0.22607500
H	-0.27110300	-1.27229000	-1.24765500
O	0.16942200	0.71427000	-0.65032200
N	-1.26030400	-1.03981600	0.50794500
H	-1.16953000	-0.95228300	1.49810200
C	-2.60358700	-1.03149400	-0.02898500
H	-2.57791900	-1.36137900	-1.06093000
C	-3.32984600	0.30333800	-0.00006900
O	-2.51597300	1.36710600	0.01644200
O	-4.50336200	0.39297400	-0.02371000
H	3.57520400	1.20490600	-1.20273400
H	-3.21948400	-1.73429100	0.51309700
H	-0.61143500	1.24135000	-0.47811700
H	-3.05523400	2.15659200	0.00803800

$IM4_P$

N	-2.56660400	1.62113600	-0.55441400
H	-2.52602300	1.51441700	-1.55301000
H	-2.73318700	2.60149300	-0.40873600
C	-3.70726700	0.86254600	-0.01947900
H	-4.63992400	1.15965500	-0.49052300
C	-3.54273800	-0.65013000	-0.16838400
H	-3.55351800	-0.92493400	-1.22104500
H	-4.42347200	-1.11050800	0.26465600
C	-2.28820600	-1.22033500	0.51808600
H	-2.50918700	-2.19417400	0.93972700
H	-2.01332000	-0.57765600	1.34626000
C	-1.09254800	-1.36748900	-0.44276200
H	-1.09198400	-0.59065000	-1.19771800
H	-1.14630600	-2.31698000	-0.97142000
C	0.23180600	-1.31662400	0.25013900
H	0.28943000	-1.44212100	1.31580300

O	-0.06410000	1.27160700	0.91288800
N	1.32943400	-1.13365800	-0.36693000
H	1.29366400	-1.02383000	-1.36499200
C	2.65682000	-0.99039600	0.24486900
H	2.54938600	-1.08500300	1.31719000
C	3.31240400	0.35432100	-0.09781700
O	4.47527600	0.39240700	-0.27278100
O	2.52919600	1.40695300	-0.18405000
H	-3.79208500	1.10707900	1.03345300
H	3.30338900	-1.77415800	-0.11683900
H	-0.86060300	1.46617200	0.39764500
H	-0.06744700	1.87289500	1.64935100
H	1.63677800	1.30686900	0.15323500

PC_P

N	3.45255700	1.75775500	-0.00089900
H	3.51689100	1.96452800	0.97811300
H	3.80708000	2.55779900	-0.48885200
C	4.23670200	0.57353500	-0.32330800
H	5.28316900	0.67135700	-0.02730100
C	3.67610100	-0.69634600	0.31827100
H	3.62015800	-0.56091400	1.39792200
H	4.40739400	-1.48439200	0.15526300
C	2.32006100	-1.18318600	-0.21355800
H	2.21587400	-2.23455000	0.04800000
H	2.33163300	-1.14335800	-1.30132500
C	1.09624200	-0.42758300	0.31118400
H	1.15258200	0.61583400	0.03423100
H	1.09623100	-0.46737700	1.40081500
C	-0.20984100	-1.01997600	-0.20692100
H	-0.27461100	-2.07717900	0.05892800
N	-1.35489900	-0.26923200	0.29439300
H	-1.31864100	-0.24158000	1.29619700
C	-2.64877300	-0.76510300	-0.13123100
H	-2.58827500	-1.04484100	-1.17827300
C	-3.72165800	0.31271500	-0.03983700
O	-4.87801600	0.05918000	-0.00451700
O	-3.26159100	1.55165100	-0.04343700
H	4.23027900	0.45763400	-1.40294000
H	-3.00262500	-1.63640300	0.41146400
H	-2.30612400	1.51916600	-0.04957300
H	-0.23076400	-0.96637200	-1.29109200

Table S2. Cartesian x,y,z coordinates of the optimized structures noted in the manuscript for the QM/MM study. Partial charges and atom types have been included. Atoms fixed during optimization are indicated by a -1.

RC

C-CT--0.3662	-1	-12.886484	1.168828	1.907526	L
H-HC-0.1123	0	-12.347521	2.076023	1.638223	L
H-HC-0.1123	0	-13.952158	1.327182	1.751200	L
H-HC-0.1123	0	-12.534551	0.340005	1.297016	L
C-C-0.5972	0	-12.635247	0.858387	3.370653	L
O-O--0.5679	0	-11.903903	1.593525	4.023637	L
N-N--0.415700	0	-13.219004	-0.235644	3.870698	L
H-H-0.271900	0	-13.790826	-0.772543	3.239456	L

C-CT--0.025200	-1	-12.940711	-0.797012	5.205905	L
H-H1-0.069800	0	-13.686983	-1.555976	5.439851	L
H-H1-0.069800	0	-12.999279	-0.002186	5.950177	L
C-C-0.597300	0	-11.525477	-1.444057	5.286595	L
O-O--0.567900	0	-10.687695	-1.274287	4.395968	L
N-N--0.415700	0	-11.241994	-2.219830	6.344517	L
H-H-0.271900	0	-11.965919	-2.354291	7.032391	L
C-CT--0.025200	-1	-10.053570	-3.099820	6.419370	L
H-H1-0.069800	0	-9.967126	-3.514703	7.423253	L
H-H1-0.069800	0	-9.154719	-2.524846	6.193496	L
C-C-0.597300	0	-10.199856	-4.248888	5.432422	L
O-O--0.567900	0	-11.244278	-4.892887	5.377112	L
N-N--0.4157	0	-9.148408	-4.500700	4.663174	L
H-H-0.2719	0	-8.323753	-3.941522	4.796666	L
C-CT--0.0014	-1	-9.090100	-5.450399	3.553198	L
H-H1-0.0876	0	-9.965079	-5.312790	2.917172	L
C-C-0.5973	0	-9.057881	-6.938544	3.966541	L
O-O--0.5679	0	-8.437100	-7.771598	3.311555	L
C-CT--0.0152	0	-7.839533	-5.100902	2.729943	L
H-HC-0.0295	0	-7.968418	-4.096298	2.342904	L
H-HC-0.0295	0	-7.814174	-5.745405	1.851252	L
C-CA--0.0011	0	-6.488972	-5.185025	3.445185	L
C-CA--0.1906	0	-5.622887	-6.264004	3.174889	L
H-HA-0.1699	0	-5.957470	-7.069392	2.531065	L
C-CA--0.1906	0	-6.046693	-4.148878	4.297832	L
H-HA-0.1699	0	-6.674163	-3.305112	4.526703	L
C-CA--0.2341	0	-4.308495	-6.275189	3.684532	L
H-HA-0.1656	0	-3.630144	-7.072976	3.428129	L
C-CA--0.2341	0	-4.731802	-4.149776	4.799162	L
H-HA-0.1656	0	-4.357506	-3.321258	5.378804	L
C-C-0.3326	0	-3.848427	-5.195320	4.465205	L
O-OH--0.5579	0	-2.540321	-5.099957	4.815598	L
H-HO-0.3992	0	-2.269333	-4.178634	4.591356	L
N-N--0.4157	0	-9.736513	-7.295729	5.056338	L
H-H-0.2719	0	-10.325776	-6.586052	5.473169	L
C-CT--0.1490	-1	-9.698621	-8.635872	5.609961	L
H-H1-0.0976	0	-8.670463	-8.899949	5.865148	L
H-H1-0.0976	0	-10.320970	-8.692393	6.503570	L
H-H1-0.0976	0	-10.065994	-9.348890	4.868959	L
C-CT--0.3662	-1	-12.039538	-1.325150	-3.506004	L
H-HC-0.1123	0	-12.535898	-0.389014	-3.756633	L
H-HC-0.1123	0	-12.049725	-1.489693	-2.430836	L
H-HC-0.1123	0	-12.555465	-2.147269	-4.001158	L
C-C-0.5972	0	-10.606053	-1.288079	-4.000897	L
O-O--0.5679	0	-10.154012	-2.150883	-4.741391	L
N-N--0.415700	0	-9.870953	-0.286451	-3.540466	L
H-H-0.271900	0	-10.345884	0.386942	-2.955826	L
C-CT--0.038900	-1	-8.427702	-0.012906	-3.708990	L
H-H1-0.100700	0	-8.136853	-0.050681	-4.756706	L
C-C-0.597300	0	-7.592602	-1.000208	-2.891604	L
O-O--0.567900	0	-6.811223	-0.618762	-2.018316	L
C-CT-0.365400	0	-8.229168	1.414175	-3.148981	L
H-H1-0.004300	0	-7.170660	1.661993	-3.162374	L
O-OH--0.676100	0	-8.728168	1.491941	-1.824774	L
H-HO-0.410200	0	-8.284015	0.811520	-1.303359	L

C-CT--0.243800	0	-9.020769	2.494196	-3.883870	L
H-HC-0.064200	0	-10.080524	2.253038	-3.941419	L
H-HC-0.064200	0	-8.634351	2.633664	-4.884717	L
H-HC-0.064200	0	-8.924277	3.434463	-3.348321	L
N-N--0.415700	0	-7.798902	-2.288068	-3.117047	L
H-H-0.271900	0	-8.457589	-2.543204	-3.848901	L
C-CT--0.024900	-1	-7.256252	-3.375630	-2.309846	L
H-H1-0.084300	0	-6.421730	-3.023639	-1.716895	L
C-C-0.597300	0	-6.726527	-4.495358	-3.210759	L
O-O--0.567900	0	-7.174380	-4.639923	-4.345465	L
C-CT-0.211700	0	-8.348942	-4.007219	-1.434984	L
H-H1-0.035200	0	-8.084337	-5.038255	-1.193593	L
H-H1-0.035200	0	-9.304442	-4.008471	-1.962173	L
O-OH--0.654600	0	-8.450908	-3.294619	-0.230321	L
H-HO-0.427500	0	-7.540238	-3.321809	0.138675	L
N-N--0.4157	0	-5.799545	-5.318307	-2.716608	L
H-H-0.2719	0	-5.513371	-5.173333	-1.749942	L
C-CT--0.0014	0	-5.411779	-6.555022	-3.401655	L
H-H1-0.0876	0	-5.084086	-6.304371	-4.410620	L
C-C-0.5973	0	-6.575165	-7.545309	-3.497362	L
O-O--0.5679	0	-7.494434	-7.530991	-2.676698	L
C-CT--0.0152	0	-4.275761	-7.279230	-2.673090	L
H-HC-0.0295	0	-4.010193	-8.171083	-3.242090	L
H-HC-0.0295	0	-4.631750	-7.631306	-1.706874	L
C-CA--0.0011	-1	-3.013511	-6.492419	-2.461592	L
C-CA--0.1906	0	-2.072274	-6.365227	-3.504350	L
H-HA-0.1699	0	-2.295468	-6.767329	-4.482057	L
C-CA--0.1906	0	-2.705325	-5.995798	-1.180311	L
H-HA-0.1699	0	-3.418425	-6.111163	-0.371443	L
C-CA--0.2341	0	-0.829816	-5.742587	-3.266442	L
H-HA-0.1656	0	-0.100482	-5.645784	-4.055483	L
C-CA--0.2341	0	-1.460782	-5.384450	-0.936316	L
H-HA-0.1656	0	-1.228146	-5.030565	0.056527	L
C-C-0.3226	0	-0.520467	-5.254288	-1.978978	L
O-OH--0.5579	0	0.686092	-4.673371	-1.740126	L
H-HO-0.3992	0	0.627911	-4.157408	-0.926881	L
N-N--0.4157	0	-6.451609	-8.475436	-4.447650	L
H-H-0.2719	0	-5.671349	-8.389524	-5.077474	L
C-CT--0.1490	-1	-7.269104	-9.671008	-4.517920	L
H-H1-0.0976	0	-8.316935	-9.390391	-4.645737	L
H-H1-0.0976	0	-6.959897	-10.295553	-5.356860	L
H-H1-0.0976	0	-7.174984	-10.238401	-3.589439	L
C-CT--0.3662	-1	-6.388169	-3.426003	-6.994556	L
H-HC-0.1123	0	-5.953534	-4.191615	-7.633774	L
H-HC-0.1123	0	-7.431305	-3.670738	-6.798171	L
H-HC-0.1123	0	-5.836478	-3.372329	-6.062090	L
C-C-0.5972	0	-6.291880	-2.079786	-7.695494	L
O-O--0.5679	0	-5.403582	-1.916714	-8.520576	L
N-N--0.415700	0	-7.176643	-1.124542	-7.371784	L
H-H-0.271900	0	-7.893967	-1.391180	-6.714690	L
C-CT--0.025200	-1	-7.301119	0.194236	-8.043143	L
H-H1-0.069800	0	-8.250069	0.219502	-8.579144	L
H-H1-0.069800	0	-6.512620	0.315543	-8.785323	L
C-C-0.597300	0	-7.263011	1.404165	-7.114939	L
O-O--0.567900	0	-8.130951	2.259885	-7.222895	L

N-N--0.415700 0 -6.263251 1.475857 -6.230280 L
H-H-0.271900 0 -5.589826 0.717685 -6.246657 L
C-CT--0.025200 -1 -5.814968 2.708730 -5.571418 L
H-H1-0.069800 0 -5.635981 2.524216 -4.515326 L
H-H1-0.069800 0 -6.569255 3.490615 -5.672235 L
C-C-0.597300 0 -4.524887 3.210083 -6.230480 L
O-O--0.567900 0 -4.464560 3.352271 -7.448902 L
N-N--0.415700 0 -3.475338 3.414325 -5.433285 L
H-H-0.271900 0 -3.594402 3.241588 -4.444339 L
C-CT--0.051800 -1 -2.135201 3.804656 -5.886451 L
H-H1-0.092200 0 -2.211252 4.268844 -6.871291 L
C-C-0.597300 0 -1.212826 2.573975 -6.011132 L
O-O--0.567900 0 -0.493737 2.392908 -6.990649 L
C-CT--0.110200 0 -1.598701 4.844339 -4.882164 L
H-HC-0.045700 0 -1.592585 4.397871 -3.887185 L
H-HC-0.045700 0 -2.288954 5.688828 -4.859916 L
C-CT-0.353100 0 -0.186339 5.374963 -5.178058 L
H-HC--0.036100 0 0.521780 4.546273 -5.188849 L
C-CT--0.412100 0 -0.104911 6.116589 -6.511933 L
H-HC-0.100000 0 -0.848658 6.914023 -6.541363 L
H-HC-0.100000 0 -0.294333 5.423897 -7.332027 L
H-HC-0.100000 0 0.889375 6.541474 -6.643560 L
C-CT--0.412100 0 0.226679 6.340828 -4.064947 L
H-HC-0.100000 0 -0.471772 7.177888 -4.024753 L
H-HC-0.100000 0 1.229658 6.720416 -4.257219 L
H-HC-0.100000 0 0.219005 5.820595 -3.108271 L
N-N--0.516300 0 -1.198859 1.743469 -4.971842 L
H-H-0.293600 0 -1.810663 2.001320 -4.204365 L
C-CT-0.038100 -1 -0.314352 0.604746 -4.741315 L
H-H1-0.088000 0 -0.073356 0.104542 -5.677122 L
C-C-0.536600 0 -0.995722 -0.375257 -3.753334 L
O-O--0.581900 0 -0.831324 -0.234781 -2.540531 L
C-CT--0.030300 0 0.994432 1.185110 -4.121712 L
H-HC--0.012200 0 0.729217 1.794340 -3.257555 L
H-HC--0.012200 0 1.461933 1.846573 -4.853096 L
C-C-0.799400 -1 2.044643 0.148200 -3.688278 L
O-O2--0.801400 0 2.067537 -0.931545 -4.321353 L
O-O2--0.801400 0 2.900670 0.427841 -2.826024 L
N-N--0.254800 0 -1.790837 -1.352078 -4.227823 L
C-CT--0.026600 -1 -2.085342 -1.648719 -5.630043 L
H-H1-0.064100 0 -1.161352 -1.712274 -6.203518 L
C-C-0.589600 0 -3.023551 -0.622783 -6.291401 L
O-O--0.574800 0 -3.970666 -0.145415 -5.664185 L
C-CT--0.007000 0 -2.738128 -3.035034 -5.596711 L
H-HC-0.025300 0 -1.962074 -3.802071 -5.601860 L
H-HC-0.025300 0 -3.425564 -3.190123 -6.428829 L
C-CT-0.018900 0 -3.455938 -3.052226 -4.247611 L
H-HC-0.021300 0 -3.618932 -4.065279 -3.888711 L
H-HC-0.021300 0 -4.400857 -2.511660 -4.315864 L
C-CT-0.019200 0 -2.489851 -2.284034 -3.352100 L
H-H1-0.039100 0 -3.034208 -1.760506 -2.565144 L
H-H1-0.039100 0 -1.768475 -2.969116 -2.907620 L
N-N--0.4157 0 -2.763033 -0.318882 -7.567851 L
H-H-0.2719 0 -1.969315 -0.762267 -7.996797 L
C-CT--0.1490 -1 -3.550507 0.565421 -8.426907 L

H-H1-0.0976	0	-4.509264	0.791275	-7.972948	L
H-H1-0.0976	0	-3.733009	0.086279	-9.389844	L
H-H1-0.0976	0	-3.014543	1.503616	-8.586950	L
C-CT--0.3662	-1	8.749337	1.823802	-5.922852	L
H-HC-0.1123	0	9.308055	2.110759	-6.813335	L
H-HC-0.1123	0	9.443715	1.493591	-5.153174	L
H-HC-0.1123	0	8.053291	1.026099	-6.174592	L
C-C-0.5972	0	7.983586	3.028026	-5.419988	L
O-O--0.5679	0	8.218259	4.136468	-5.877851	L
N-N--0.415700	0	7.066644	2.820873	-4.474653	L
H-H-0.271900	0	6.943713	1.865944	-4.162317	L
C-CT-0.021300	-1	6.100830	3.840222	-4.033779	L
H-H1-0.112400	0	6.314681	4.789834	-4.529529	L
C-C-0.597300	0	6.147003	4.075102	-2.522678	L
O-O--0.567900	0	6.347851	3.143550	-1.738761	L
C-CT--0.123100	0	4.712901	3.361148	-4.495657	L
H-H1-0.111200	0	4.494849	2.379200	-4.067852	L
H-H1-0.111200	0	4.712902	3.272425	-5.584243	L
S-SH--0.311900	0	3.416417	4.530899	-4.000446	L
H-HS-0.193300	0	2.411041	3.973434	-4.693089	L
N-N--0.415700	0	5.905039	5.314228	-2.098794	L
H-H-0.271900	0	5.709187	6.022641	-2.794808	L
C-CT--0.025200	-1	5.860673	5.720912	-0.699693	L
H-H1-0.069800	0	6.821825	6.163809	-0.437610	L
H-H1-0.069800	0	5.710042	4.864531	-0.045645	L
C-C-0.597300	0	4.782498	6.771892	-0.439303	L
O-O--0.567900	0	4.891501	7.907382	-0.888915	L
N-N--0.415700	0	3.764077	6.406833	0.334274	L
H-H-0.271900	0	3.824147	5.498645	0.780087	L
C-CT--0.025200	-1	2.744152	7.283728	0.890014	L
H-H1-0.069800	0	1.894006	6.680787	1.196908	L
H-H1-0.069800	0	2.425105	8.019955	0.151340	L
C-C-0.597300	0	3.278141	7.998941	2.127486	L
O-O--0.567900	0	2.780244	7.772237	3.229274	L
N-N--0.4157	0	4.317231	8.818777	1.927680	L
H-H-0.2719	0	4.620241	8.905585	0.962927	L
C-CT--0.1490	-1	5.095118	9.481907	2.967530	L
H-H1-0.0976	0	5.459300	8.744195	3.684730	L
H-H1-0.0976	0	5.942997	10.006927	2.524795	L
H-H1-0.0976	0	4.464893	10.200502	3.495186	L
C-CT--0.3662	-1	-1.433444	9.448233	5.280514	L
H-HC-0.1123	0	-1.258923	10.105158	4.430435	L
H-HC-0.1123	0	-0.536388	8.866266	5.484797	L
H-HC-0.1123	0	-1.692531	10.047266	6.151672	L
C-C-0.5972	0	-2.608051	8.516869	4.957101	L
O-O--0.5679	0	-3.432829	8.859953	4.120685	L
N-N--0.4157	0	-2.657904	7.325760	5.566523	L
H-H-0.2719	0	-1.961285	7.134189	6.273872	L
C-CT--0.0275	-1	-3.564585	6.179554	5.294205	L
H-H1-0.1123	0	-4.577395	6.543191	5.119221	L
C-C-0.5973	0	-3.575962	5.227232	6.519476	L
O-O--0.5679	0	-2.717037	5.337058	7.393906	L
C-CT--0.0050	0	-3.062031	5.406881	4.050313	L
H-HC-0.0339	0	-3.355565	5.970180	3.170274	L
H-HC-0.0339	0	-3.614269	4.469539	3.993483	L

C-C*--0.1415	0	-1.584788	5.079864	3.936511	L
C-CW--0.1638	0	-0.569166	5.976058	3.847529	L
H-H4-0.2062	0	-0.677299	7.049943	3.841594	L
C-CB-0.1243	0	-0.935151	3.767754	3.848440	L
N-NA--0.3418	0	0.647188	5.327573	3.788459	L
H-H-0.3412	0	1.540770	5.810962	3.757153	L
C-CN-0.1380	0	0.481332	3.961451	3.802626	L
C-CA--0.238	0	-1.389158	2.429934	3.798439	L
H-HA-0.1700	0	-2.447545	2.222429	3.783738	L
C-CA--0.2601	0	1.393268	2.896231	3.772051	L
H-HA-0.1572	0	2.453263	3.095550	3.748842	L
C-CA--0.1972	0	-0.482165	1.352287	3.747285	L
H-HA-0.1447	0	-0.852384	0.334787	3.685210	L
C-CA--0.1134	0	0.904555	1.579039	3.753481	L
H-HA-0.1417	0	1.580349	0.734285	3.723029	L
N-N--0.4157	0	-4.526823	4.279154	6.588906	L
H-H-0.2719	0	-5.197797	4.256596	5.838570	L
C-CT--0.1490	-1	-4.641665	3.277620	7.663728	L
H-H1-0.0976	0	-3.741437	2.661522	7.687782	L
H-H1-0.0976	0	-4.743391	3.782943	8.626618	L
H-H1-0.0976	0	-5.511993	2.640614	7.498456	L
C-CT--0.3662	-1	-2.845359	-1.460663	7.254924	L
H-HC-0.1123	0	-3.068744	-2.509209	7.448002	L
H-HC-0.1123	0	-3.260782	-0.843276	8.047911	L
H-HC-0.1123	0	-3.268071	-1.183815	6.289759	L
C-C-0.5972	0	-1.351390	-1.291016	7.178923	L
O-O--0.5679	0	-0.646525	-2.262242	6.972559	L
N-N--0.415700	0	-0.842613	-0.084788	7.427009	L
H-H-0.271900	0	-1.495248	0.677058	7.517032	L
C-CT--0.087500	-1	0.603859	0.226276	7.403124	L
H-H1-0.096900	0	0.940240	0.136997	6.368993	L
C-C-0.597300	0	1.436034	-0.753753	8.241925	L
O-O--0.567900	0	2.426287	-1.293891	7.760408	L
C-CT-0.298500	0	0.889568	1.670515	7.881060	L
H-HC--0.029700	0	0.829056	1.701963	8.969632	L
C-CT--0.319200	0	2.300447	2.106300	7.464926	L
H-HC-0.079100	0	2.386983	2.093487	6.378398	L
H-HC-0.079100	0	3.045525	1.434542	7.891205	L
H-HC-0.079100	0	2.498152	3.115387	7.826972	L
C-CT--0.319200	0	-0.105527	2.707101	7.344577	L
H-HC-0.079100	0	-0.152757	2.642856	6.261771	L
H-HC-0.079100	0	0.209975	3.712899	7.623641	L
H-HC-0.079100	0	-1.098239	2.545778	7.761345	L
N-N--0.415700	0	1.018104	-1.013204	9.488567	L
H-H-0.271900	0	0.194152	-0.524930	9.799699	L
C-CT--0.025200	-1	1.617875	-2.020881	10.378360	L
H-H1-0.069800	0	1.069141	-2.052715	11.318962	L
H-H1-0.069800	0	2.649339	-1.739145	10.592912	L
C-C-0.597300	0	1.632372	-3.448766	9.780905	L
O-O--0.567900	0	2.470626	-4.254317	10.167421	L
N-N--0.415700	0	0.717155	-3.766595	8.853903	L
H-H-0.271900	0	0.106661	-3.030645	8.526893	L
C-CT-0.033700	-1	0.581538	-5.044750	8.143559	L
H-H1-0.082300	0	1.035638	-5.822487	8.760424	L
C-C-0.597300	0	1.311117	-5.098954	6.776872	L

O-O--0.567900 0 1.745627 -6.172112 6.355535 L
C-CT--0.182500 0 -0.907800 -5.387921 8.004807 L
H-HC-0.060300 0 -1.384236 -5.381330 8.985467 L
H-HC-0.060300 0 -1.013433 -6.381795 7.568989 L
H-HC-0.060300 0 -1.407182 -4.670466 7.354611 L
N-N--0.415700 0 1.455653 -3.968845 6.078526 L
H-H-0.271900 0 0.928953 -3.166348 6.405616 L
C-CT-0.033700 -1 2.393380 -3.751712 4.964184 L
H-H1-0.082300 0 2.255693 -4.525208 4.212436 L
C-C-0.597300 0 3.864485 -3.790110 5.435393 L
O-O--0.567900 0 4.772297 -4.054124 4.644617 L
C-CT--0.182500 0 2.075843 -2.376473 4.368594 L
H-HC-0.060300 0 2.542281 -1.589384 4.961838 L
H-HC-0.060300 0 1.001011 -2.204532 4.369884 L
H-HC-0.060300 0 2.445630 -2.330862 3.345664 L
N-N--0.4157 0 4.103811 -3.471431 6.714616 L
H-H-0.2719 0 3.306698 -3.124335 7.234304 L
C-CT--0.1490 -1 5.380648 -3.555224 7.409656 L
H-H1-0.0976 0 5.892426 -4.479108 7.133241 L
H-H1-0.0976 0 5.213242 -3.549912 8.488189 L
H-H1-0.0976 0 6.006591 -2.704872 7.134863 L
C-CT--0.3662 -1 14.075176 0.782264 -0.697681 L
H-HC-0.1123 0 13.468892 1.304246 0.039595 L
H-HC-0.1123 0 15.090694 1.175534 -0.685751 L
H-HC-0.1123 0 13.645679 0.906319 -1.689684 L
C-C-0.5972 0 14.116697 -0.698086 -0.356255 L
O-O--0.5679 0 15.191845 -1.268479 -0.234636 L
N-N--0.4157 0 12.949141 -1.325357 -0.218147 L
H-H-0.2719 0 12.113003 -0.768697 -0.349372 L
C-CT--0.0014 -1 12.757738 -2.776763 -0.015476 L
H-H1-0.0876 0 13.631294 -3.305376 -0.399736 L
C-C-0.5973 0 11.520107 -3.254010 -0.829291 L
O-O--0.5679 0 10.747346 -2.442168 -1.349882 L
C-CT--0.0152 0 12.618924 -3.095815 1.483582 L
H-HC-0.0295 0 13.565929 -2.859449 1.971337 L
H-HC-0.0295 0 12.460628 -4.167854 1.606222 L
C-CA--0.0011 -1 11.505824 -2.350444 2.200761 L
C-CA--0.1906 0 11.781261 -1.110432 2.807981 L
H-HA-0.1699 0 12.784684 -0.708184 2.772489 L
C-CA--0.1906 0 10.200053 -2.877601 2.251513 L
H-HA-0.1699 0 9.980344 -3.835127 1.800348 L
C-CA--0.2341 0 10.758790 -0.400070 3.463249 L
H-HA-0.1656 0 10.976504 0.537123 3.942779 L
C-CA--0.2341 0 9.172560 -2.165754 2.902002 L
H-HA-0.1656 0 8.175579 -2.578408 2.942799 L
C-C-0.3226 0 9.449349 -0.923403 3.514072 L
O-OH--0.557 0 8.466814 -0.229543 4.150704 L
H-HO-0.3992 0 7.657388 -0.778672 4.211001 L
N-N--0.415700 0 11.311358 -4.570036 -0.972020 L
H-H-0.271900 0 11.943183 -5.198315 -0.500370 L
C-CT--0.025200 -1 10.175674 -5.154524 -1.715212 L
H-H1-0.069800 0 10.372811 -6.211152 -1.895503 L
H-H1-0.069800 0 10.074616 -4.660883 -2.681247 L
C-C-0.597300 0 8.854070 -5.051113 -0.940006 L
O-O--0.567900 0 8.788569 -5.431652 0.227028 L

N-N--0.415700	0	7.786855	-4.558800	-1.575763	L
H-H-0.271900	0	7.868025	-4.449785	-2.579676	L
C-CT-0.014300	-1	6.448160	-4.528324	-0.987539	L
H-H1-0.104800	0	6.533234	-4.412780	0.096047	L
C-C-0.597300	0	5.716444	-5.842919	-1.267264	L
O-O--0.567900	0	5.019903	-5.980716	-2.278826	L
C-CT--0.204100	0	5.619917	-3.331618	-1.513853	L
H-HC-0.079700	0	5.436899	-3.449704	-2.581657	L
H-HC-0.079700	0	6.211635	-2.440681	-1.370139	L
C-C-0.713000	-1	4.266221	-3.145133	-0.803663	L
O-O--0.593100	0	3.861930	-3.905842	0.064669	L
N-N--0.919100	0	3.506478	-2.135328	-1.165478	L
H-H-0.419600	0	2.716220	-1.970904	-0.549576	L
H-H-0.419600	0	3.668881	-1.501767	-1.937742	L
N-N--0.347900	0	5.843980	-6.783808	-0.331190	L
H-H-0.274700	0	6.493036	-6.564211	0.414319	L
C-CT--0.263700	-1	5.265230	-8.130442	-0.405843	L
H-H1-0.156000	0	5.623163	-8.681650	0.463906	L
C-C-0.734100	0	5.768649	-8.933374	-1.615788	L
O-O--0.589400	0	6.797604	-8.618653	-2.207215	L
C-CT--0.000700	0	3.722847	-8.065381	-0.312692	L
H-HC-0.032700	0	3.321239	-9.078766	-0.279690	L
H-HC-0.032700	0	3.330106	-7.583763	-1.209318	L
C-CT-0.039000	0	3.185322	-7.335333	0.924160	L
H-HC-0.028500	0	3.582773	-6.323462	0.979251	L
H-HC-0.028500	0	3.464559	-7.881573	1.826258	L
C-CT-0.048600	0	1.661825	-7.274525	0.800875	L
H-H1-0.068700	0	1.269558	-8.289077	0.710008	L
H-H1-0.068700	0	1.392893	-6.726977	-0.104219	L
N-N2--0.529500	-1	1.036602	-6.638794	1.962645	L
H-H-0.345600	0	0.664810	-7.222273	2.706776	L
C-CA-0.807600	0	0.759861	-5.368589	2.130730	L
N-N2--0.862700	0	-0.066775	-5.050177	3.070897	L
H-H-0.447800	0	-0.477002	-5.740246	3.682974	L
H-H-0.447800	0	-0.334090	-4.064117	3.150932	L
N-N2--0.862700	0	1.266081	-4.410532	1.405003	L
H-H-0.447800	0	2.136093	-4.513860	0.899868	L
H-H-0.447800	0	0.908122	-3.480187	1.659426	L
N-N--0.4157	0	5.060003	-10.018813	-1.933192	L
H-H-0.2719	0	4.248180	-10.202949	-1.368848	L
C-CT--0.1490	-1	5.363402	-10.922907	-3.027403	L
H-H1-0.0976	0	6.355942	-11.355604	-2.883848	L
H-H1-0.0976	0	4.625694	-11.725567	-3.072458	L
H-H1-0.0976	0	5.357470	-10.374425	-3.971458	L
C-CT--0.3662	-1	10.070820	-6.025889	-7.790623	L
H-HC-0.1123	0	9.776715	-6.717662	-8.577528	L
H-HC-0.1123	0	10.979902	-5.505527	-8.090716	L
H-HC-0.1123	0	10.254468	-6.562917	-6.861573	L
C-C-0.5972	0	8.971791	-5.006464	-7.574018	L
O-O--0.5679	0	9.214475	-3.812088	-7.708862	L
N-N--0.347900	0	7.769130	-5.447528	-7.174764	L
H-H-0.274700	0	7.675425	-6.443224	-7.041780	L
C-CT--0.263700	-1	6.801889	-4.540089	-6.523341	L
H-H1-0.156000	0	6.624703	-3.679581	-7.169400	L
C-C-0.734100	0	7.457464	-4.045172	-5.230788	L

O-O--0.589400	0	7.824424	-4.853028	-4.382516	L
C-CT--0.000700	0	5.464345	-5.227502	-6.200284	L
H-HC-0.032700	0	4.875496	-4.520282	-5.617688	L
H-HC-0.032700	0	5.634582	-6.103115	-5.569989	L
C-CT-0.039000	0	4.667210	-5.648819	-7.446477	L
H-HC-0.028500	0	4.916021	-6.684758	-7.680304	L
H-HC-0.028500	0	4.952222	-5.033332	-8.301351	L
C-CT-0.048600	0	3.147046	-5.530632	-7.235604	L
H-H1-0.068700	0	2.879609	-5.911271	-6.247674	L
H-H1-0.068700	0	2.650283	-6.160275	-7.976184	L
N-N2--0.529500	0	2.680304	-4.139361	-7.423311	L
H-H-0.345600	0	2.596892	-3.816349	-8.370842	L
C-CA-0.807600	0	2.414174	-3.231653	-6.498783	L
N-N2--0.862700	0	2.412904	-3.467227	-5.224956	L
H-H-0.447800	0	2.565067	-4.389425	-4.827303	L
H-H-0.447800	0	2.242493	-2.668418	-4.608527	L
N-N2--0.862700	0	2.148243	-2.004582	-6.828816	L
H-H-0.447800	0	2.136272	-1.654151	-7.765429	L
H-H-0.447800	0	2.049724	-1.382896	-6.021387	L
N-N--0.415700	0	7.661243	-2.739084	-5.091275	L
H-H-0.271900	0	7.349569	-2.134263	-5.833696	L
C-CT--0.025200	-1	8.573588	-2.180693	-4.090315	L
H-H1-0.069800	0	9.486613	-1.868554	-4.598321	L
H-H1-0.069800	0	8.857098	-2.931907	-3.357869	L
C-C-0.597300	0	8.029609	-0.977742	-3.342681	L
O-O--0.567900	0	7.054754	-0.347037	-3.755006	L
N-N--0.415700	0	8.676519	-0.648565	-2.228527	L
H-H-0.271900	0	9.486929	-1.198453	-1.944628	L
C-CT--0.038900	-1	8.290162	0.473278	-1.379046	L
H-H1-0.100700	0	7.597161	1.090997	-1.944417	L
C-C-0.597300	0	9.444989	1.401299	-1.031842	L
O-O--0.567900	0	10.561047	0.979812	-0.734645	L
C-CT-0.365400	0	7.532374	0.000832	-0.126125	L
H-H1-0.004300	0	7.932532	-0.954729	0.216226	L
O-OH--0.676100	0	6.196362	-0.148109	-0.534283	L
H-HO-0.410200	0	6.203221	-0.693615	-1.327290	L
C-CT--0.243800	0	7.470327	0.972049	1.054177	L
H-HC-0.064200	0	7.057606	1.930810	0.742432	L
H-HC-0.064200	0	8.466103	1.112876	1.473250	L
H-HC-0.064200	0	6.832469	0.549727	1.831357	L
N-N--0.415700	0	9.123320	2.695054	-1.061254	L
H-H-0.271900	0	8.157955	2.915139	-1.276906	L
C-CT--0.025200	-1	9.978218	3.801421	-0.672770	L
H-H1-0.069800	0	9.811257	4.635166	-1.354698	L
H-H1-0.069800	0	11.024882	3.502031	-0.751910	L
C-C-0.597300	0	9.681009	4.276553	0.735888	L
O-O--0.567900	0	8.532869	4.504210	1.130406	L
N-N--0.347900	0	10.755920	4.466981	1.486309	L
H-H-0.274700	0	11.660739	4.302667	1.059267	L
C-CT--0.263700	-1	10.775551	5.065985	2.812051	L
H-H1-0.156000	0	9.873233	5.654778	2.967150	L
C-C-0.734100	0	11.949513	6.017884	2.907830	L
O-O--0.589400	0	12.880675	5.949799	2.111149	L
C-CT--0.000700	0	10.919977	3.964297	3.885401	L
H-HC-0.032700	0	11.206570	4.410410	4.839351	L

H-HC-0.032700	0	11.724114	3.286777	3.590364	L
C-CT-0.039000	0	9.648158	3.143995	4.113916	L
H-HC-0.028500	0	9.885764	2.304116	4.766066	L
H-HC-0.028500	0	9.285029	2.749956	3.164697	L
C-CT-0.048600	0	8.559073	3.973026	4.798483	L
H-H1-0.068700	0	8.398938	4.909668	4.265377	L
H-H1-0.068700	0	8.878915	4.211426	5.814687	L
N-N2--0.529500	-1	7.305859	3.216171	4.849985	L
H-H-0.345600	0	7.197493	2.540544	5.587884	L
C-CA-0.807600	0	6.333896	3.233212	3.963530	L
N-N2--0.862700	0	5.291095	2.472938	4.099623	L
H-H-0.447800	0	5.235698	1.756047	4.798007	L
H-H-0.447800	0	4.670919	2.418674	3.287988	L
N-N2--0.862700	0	6.374116	3.991051	2.914149	L
H-H-0.447800	0	7.233524	4.379101	2.554432	L
H-H-0.447800	0	5.501013	4.067122	2.397763	L
N-N--0.4157	0	11.886793	6.882282	3.911699	L
H-H-0.2719	0	11.083733	6.855796	4.516696	L
C-CT--0.1490	-1	12.957650	7.799919	4.204799	L
H-H1-0.0976	0	12.706956	8.414248	5.071166	L
H-H1-0.0976	0	13.876575	7.247041	4.413839	L
H-H1-0.0976	0	13.136106	8.451360	3.345992	L
C-CT--0.3662	-1	-2.047939	13.278738	-1.531874	L
H-HC-0.1123	0	-2.498708	13.792701	-0.685430	L
H-HC-0.1123	0	-0.963494	13.269557	-1.443208	L
H-HC-0.1123	0	-2.326892	13.791506	-2.452189	L
C-C-0.5972	0	-2.564529	11.852955	-1.597112	L
O-O--0.5679	0	-3.181280	11.471995	-2.579384	L
N-N--0.415700	0	-2.318788	11.071786	-0.541408	L
H-H-0.271900	0	-1.787154	11.493110	0.201458	L
C-CT--0.023700	-1	-2.753824	9.667107	-0.384569	L
H-H1-0.088000	0	-2.295009	9.079196	-1.181276	L
C-C-0.597300	0	-4.275164	9.502785	-0.547939	L
O-O--0.567900	0	-4.745111	8.734773	-1.387409	L
C-CT-0.034200	0	-2.215979	9.157741	0.958286	L
H-HC-0.024100	0	-2.565868	9.812511	1.757135	L
H-HC-0.024100	0	-1.126560	9.203155	0.940206	L
C-CT-0.001800	0	-2.637074	7.728911	1.290919	L
H-H1-0.044000	0	-3.711276	7.723627	1.479349	L
H-H1-0.044000	0	-2.139129	7.456521	2.216000	L
S-S--0.273700	0	-2.282596	6.448474	0.062130	L
C-CT--0.053600	0	-0.486132	6.333512	0.125383	L
H-H1-0.068400	0	-0.151767	5.601413	-0.608973	L
H-H1-0.068400	0	-0.184080	6.008514	1.119774	L
H-H1-0.068400	0	-0.047723	7.303270	-0.106710	L
N-N--0.4157	0	-5.051702	10.239007	0.251483	L
H-H-0.2719	0	-4.590382	10.836643	0.918085	L
C-CT--0.1490	-1	-6.501944	10.276425	0.147167	L
H-H1-0.0976	0	-6.916910	10.942006	0.905373	L
H-H1-0.0976	0	-6.789058	10.636933	-0.842956	L
H-H1-0.0976	0	-6.907672	9.273033	0.288848	L
C-CT--0.3662	-1	-4.369528	9.045647	-5.137748	L
H-HC-0.1123	0	-3.926573	9.393365	-4.205364	L
H-HC-0.1123	0	-3.614957	9.046673	-5.922234	L
H-HC-0.1123	0	-5.193554	9.694663	-5.425193	L

C-C-0.5972	0	-4.890217	7.626684	-4.957743	L
O-O--0.5679	0	-4.597986	6.763743	-5.773303	L
N-N--0.415700	0	-5.670078	7.389045	-3.899277	L
H-H-0.271900	0	-5.693513	8.136815	-3.217635	L
C-CT--0.087500	-1	-6.351383	6.106206	-3.578094	L
H-H1-0.096900	0	-6.072529	5.358395	-4.319766	L
C-C-0.597300	0	-7.862382	6.287669	-3.683933	L
O-O--0.567900	0	-8.506362	5.623528	-4.486491	L
C-CT-0.298500	0	-5.910904	5.601553	-2.194069	L
H-HC--0.029700	0	-6.099575	6.372166	-1.446059	L
C-CT--0.319200	0	-6.618792	4.320660	-1.748334	L
H-HC-0.079100	0	-6.429003	3.537045	-2.475481	L
H-HC-0.079100	0	-7.691081	4.488756	-1.657507	L
H-HC-0.079100	0	-6.239899	4.001051	-0.776954	L
C-CT--0.319200	0	-4.418197	5.288919	-2.225219	L
H-HC-0.079100	0	-4.203102	4.548974	-2.991942	L
H-HC-0.079100	0	-4.129590	4.904033	-1.255324	L
H-HC-0.079100	0	-3.843564	6.192129	-2.428291	L
N-N--0.4157	0	-8.437045	7.196484	-2.888189	L
H-H-0.2719	0	-7.824682	7.713521	-2.277407	L
C-CT--0.1490	-1	-9.863654	7.501817	-2.920847	L
H-H1-0.0976	0	-10.103482	8.276261	-2.191741	L
H-H1-0.0976	0	-10.140183	7.847410	-3.919391	L
H-H1-0.0976	0	-10.437508	6.600362	-2.697133	L
C-CT-0.1200	-1	-8.870609	2.051809	3.223204	L
H-HC-0.0800	0	-8.174771	2.881352	3.341326	L
H-HC-0.0800	0	-9.640566	2.330421	2.504114	L
H-HC-0.0800	0	-9.339936	1.832166	4.182493	L
C-CT-0.2000	0	-8.146109	0.836758	2.729765	L
H-H1-0.0800	0	-8.878038	0.044912	2.546612	L
O-OS--0.5600	0	-7.439551	1.162159	1.554161	L
C-CT-0.2000	0	-7.068584	0.347849	3.691639	L
H-H1-0.0800	0	-6.399917	1.160003	3.972274	L
O-OH--0.6800	0	-7.595973	-0.273019	4.855573	L
H-HO-0.4000	0	-8.139033	-1.015130	4.549938	L
C-CT-0.2000	0	-6.374674	-0.602027	2.717186	L
H-H1-0.0800	0	-5.353931	-0.829978	3.030223	L
O-OH--0.6800	0	-7.182180	-1.776667	2.682364	L
H-HO-0.4000	0	-6.767094	-2.408208	2.054588	L
C-CT-0.5691	0	-6.411129	0.176010	1.385987	H
H-H2-0.8000	0	-6.653302	-0.535987	0.573162	H
N-N*--0.5691	0	-5.172711	0.894847	1.092742	H
C-CM--0.0500	0	-4.686741	0.744952	-0.169585	H
H-H4-0.1500	0	-5.103516	-0.097496	-0.671754	H
C-CM--0.1238	0	-3.800552	1.548713	-0.764732	H
C-C-0.6156	0	-3.602158	1.530087	-2.224547	H
O-O--0.5700	0	-2.835607	2.289590	-2.767610	H
N-N--0.8000	0	-4.405298	0.723321	-2.982340	H
H-H-0.3700	0	-4.099738	0.505157	-3.909912	H
H-H-0.3700	0	-4.980655	0.026142	-2.557927	H
C-CT-0.1164	0	-3.036419	2.543998	0.072441	H
H-HC-0.0800	0	-2.785463	3.422282	-0.504834	H
H-HC-0.0800	0	-2.092144	2.111591	0.397078	H
C-CM--0.2882	0	-3.870104	2.888265	1.280450	H
H-HA-0.1500	0	-3.654276	3.804825	1.793950	H

C-CM--0.0500	0	-4.816568	2.090246	1.739322	H
H-H4-0.1500	0	-5.372338	2.338474	2.616278	H
N-N3--0.8530	0	4.065676	1.698372	-0.818241	H
H-H-0.4500	0	3.555821	1.203797	-1.533791	H
H-H-0.4500	0	4.059302	1.193637	0.058339	H
H-H-0.4500	0	5.035851	1.799752	-1.049427	H
C-CT-0.3170	0	3.513453	3.006885	-0.395643	H
H-HP-0.0800	0	4.127205	3.761388	-0.854742	H
C-CT--0.1600	0	2.056488	3.251554	-0.774797	H
H-HC-0.0800	0	1.954506	3.016468	-1.828647	H
H-HC-0.0800	0	1.934582	4.322902	-0.690518	H
C-CT--0.1600	0	0.961876	2.571492	0.049297	H
H-HC-0.0800	0	0.022634	2.958318	-0.316165	H
H-HC-0.0800	0	1.052113	2.905656	1.078080	H
C-CT--0.0990	0	0.891560	1.045840	0.011253	H
H-HC-0.0800	0	0.963879	0.663071	-1.002001	H
H-HC-0.0800	0	-0.077295	0.755493	0.393931	H
C-C-0.4490	0	1.872242	0.307271	0.890477	H
H-H-0.0600	0	2.463575	0.944511	1.578634	H
O-O--0.5700	0	1.998647	-0.875674	0.926123	H
N-N3--0.9900	0	-1.587302	-0.593932	1.087592	H
H-H-0.3600	0	-0.746487	-1.066107	0.823627	H
H-H-0.3600	0	-2.044481	-0.319645	0.230634	H
C-C-0.9060	0	3.824726	2.991808	1.094907	L
O-O2--0.9000	0	4.036609	1.913036	1.701863	L
O-O2--0.9000	0	3.992988	4.072960	1.680881	L
C-CT--0.205900	0	-2.394635	-1.561110	1.816074	H
H-H1-0.139900	0	-3.173868	-1.035410	2.354134	H
C-CT-0.007100	0	-3.023033	-2.539753	0.814631	H
H-HC--0.007800	0	-2.230111	-3.099811	0.335344	H
H-HC--0.007800	0	-3.524212	-1.971221	0.029648	H
C-CT-0.067500	0	-4.018634	-3.519479	1.418136	L
H-HC--0.054800	0	-4.291872	-3.159001	2.407508	L
H-HC--0.054800	0	-3.554697	-4.503453	1.511559	L
C-C-0.818300	0	-5.297661	-3.586720	0.590976	L
O-O2--0.822000	0	-6.027798	-2.560372	0.553045	L
O-O2--0.822000	0	-5.626378	-4.628765	-0.012450	L
C-C-0.742000	0	-1.461551	-2.305949	2.792031	H
O-O2--0.793000	0	-1.917417	-2.736326	3.848939	H
O-O2--0.793000	0	-0.317373	-2.471770	2.348115	H
O-OW--0.834000	0	2.701076	-5.710120	-3.578103	L
H-HW-0.417000	0	3.543957	-5.893599	-3.115667	L
H-HW-0.417000	0	2.098522	-5.479530	-2.853592	L
O-OW--0.834000	0	0.199017	-7.539340	4.583526	L
H-HW-0.417000	0	0.850340	-7.144732	5.196763	L
H-HW-0.417000	0	-0.403725	-7.998246	5.174781	L
O-OW--0.834000	0	4.276873	-0.448135	2.765785	L
H-HW-0.417000	0	3.563942	-0.917348	2.308244	L
H-HW-0.417000	0	4.233421	0.451318	2.374023	L
O-OW--0.834000	0	6.149609	-1.617601	4.282946	L
H-HW-0.417000	0	5.801636	-2.511599	4.446205	L
H-HW-0.417000	0	5.447442	-1.218087	3.726515	L
O-OW--0.834000	0	-6.460772	-7.161725	-0.022450	L
H-HW-0.417000	0	-6.192436	-6.222500	0.027707	L
H-HW-0.417000	0	-6.986167	-7.216760	-0.830895	L

O-OW--0.834000	0	-8.879444	-0.734473	0.028718	L
H-HW-0.417000	0	-9.820133	-0.538348	0.016979	L
H-HW-0.417000	0	-8.857913	-1.709829	-0.102887	L

TS1

C-CT--0.3662	-1	-12.856370	1.169262	1.878505	L
H-HC-0.1123	0	-12.313981	2.076268	1.615594	L
H-HC-0.1123	0	-13.921781	1.334727	1.727533	L
H-HC-0.1123	0	-12.511636	0.344559	1.258344	L
C-C-0.5972	0	-12.601477	0.843208	3.337898	L
O-O--0.5679	0	-11.856246	1.562267	3.993848	L
N-N--0.415700	0	-13.196943	-0.247483	3.831270	L
H-H-0.271900	0	-13.779857	-0.770967	3.198707	L
C-CT--0.025200	-1	-12.919684	-0.824110	5.160154	L
H-H1-0.069800	0	-13.670725	-1.580425	5.387497	L
H-H1-0.069800	0	-12.972221	-0.036450	5.912404	L
C-C-0.597300	0	-11.510319	-1.480849	5.232723	L
O-O--0.567900	0	-10.671149	-1.302406	4.345769	L
N-N--0.415700	0	-11.232236	-2.268091	6.283121	L
H-H-0.271900	0	-11.963284	-2.416857	6.960520	L
C-CT--0.025200	-1	-10.031914	-3.129712	6.366792	L
H-H1-0.069800	0	-9.954945	-3.551289	7.368564	L
H-H1-0.069800	0	-9.141032	-2.535731	6.160384	L
C-C-0.597300	0	-10.131606	-4.275823	5.366560	L
O-O--0.567900	0	-11.174560	-4.914269	5.272665	L
N-N--0.4157	0	-9.052160	-4.536026	4.629182	L
H-H-0.2719	0	-8.231863	-3.966796	4.754987	L
C-CT--0.0014	-1	-9.050255	-5.453921	3.485328	L
H-H1-0.0876	0	-9.857982	-5.145793	2.819517	L
C-C-0.5973	0	-9.326105	-6.933690	3.794958	L
O-O--0.5679	0	-9.587755	-7.710166	2.880508	L
C-CT--0.0152	0	-7.740650	-5.278707	2.698150	L
H-HC-0.0295	0	-7.667416	-4.239947	2.378662	L
H-HC-0.0295	0	-7.807377	-5.875178	1.787410	L
C-CA--0.0011	0	-6.461861	-5.658625	3.429101	L
C-CA--0.1906	0	-6.034612	-7.000231	3.439400	L
H-HA-0.1699	0	-6.626365	-7.754629	2.932878	L
C-CA--0.1906	0	-5.669194	-4.670908	4.049253	L
H-HA-0.1699	0	-5.972300	-3.638075	4.031474	L
C-CA--0.2341	0	-4.826112	-7.355121	4.069007	L
H-HA-0.1656	0	-4.500123	-8.384180	4.075739	L
C-CA--0.2341	0	-4.447917	-5.014342	4.658804	L
H-HA-0.1656	0	-3.821451	-4.245048	5.084751	L
C-C-0.3326	0	-4.028324	-6.360714	4.671602	L
O-OH--0.5579	0	-2.836160	-6.696752	5.230606	L
H-HO-0.3992	0	-2.439641	-5.937308	5.664799	L
N-N--0.4157	0	-9.279746	-7.328374	5.069753	L
H-H-0.2719	0	-9.086062	-6.614311	5.750242	L
C-CT--0.1490	-1	-9.659460	-8.657928	5.512892	L
H-H1-0.0976	0	-9.068641	-9.405995	4.980164	L
H-H1-0.0976	0	-9.492402	-8.762458	6.585290	L
H-H1-0.0976	0	-10.715339	-8.828560	5.293015	L
C-CT--0.3662	-1	-11.979996	-1.277414	-3.551899	L
H-HC-0.1123	0	-12.473970	-0.336333	-3.788468	L
H-HC-0.1123	0	-11.995837	-1.460680	-2.479790	L

H-HC-0.1123 0 -12.494886 -2.089782 -4.063970 L
C-C-0.5972 0 -10.545416 -1.233235 -4.039111 L
O-O--0.5679 0 -10.093541 -2.079376 -4.798494 L
N-N--0.415700 0 -9.809209 -0.247251 -3.548146 L
H-H-0.271900 0 -10.287588 0.411993 -2.950348 L
C-CT--0.038900 -1 -8.370669 0.045509 -3.728471 L
H-H1-0.100700 0 -8.101026 0.053853 -4.782510 L
C-C-0.597300 0 -7.492491 -0.955167 -2.974136 L
O-O--0.567900 0 -6.613633 -0.582838 -2.194829 L
C-CT-0.365400 0 -8.175388 1.453751 -3.120529 L
H-H1-0.004300 0 -7.117850 1.707186 -3.122900 L
O-OH--0.676100 0 -8.679378 1.493897 -1.795624 L
H-HO-0.410200 0 -8.355040 0.718414 -1.317729 L
C-CT--0.243800 0 -8.965151 2.552071 -3.830367 L
H-HC-0.064200 0 -10.016048 2.291743 -3.940637 L
H-HC-0.064200 0 -8.544226 2.749037 -4.808030 L
H-HC-0.064200 0 -8.910537 3.468269 -3.248688 L
N-N--0.415700 0 -7.766627 -2.240270 -3.142080 L
H-H-0.271900 0 -8.475149 -2.486736 -3.828718 L
C-CT--0.024900 -1 -7.196673 -3.325807 -2.352336 L
H-H1-0.084300 0 -6.367308 -2.965092 -1.758395 L
C-C-0.597300 0 -6.646761 -4.436370 -3.258550 L
O-O--0.567900 0 -7.103555 -4.579690 -4.390038 L
C-CT-0.211700 0 -8.266158 -3.999647 -1.476686 L
H-H1-0.035200 0 -7.971938 -5.027729 -1.256782 L
H-H1-0.035200 0 -9.224055 -4.020624 -1.999164 L
O-OH--0.654600 0 -8.389619 -3.322751 -0.252630 L
H-HO-0.427500 0 -7.476900 -3.304724 0.112077 L
N-N--0.4157 0 -5.707025 -5.263183 -2.780253 L
H-H-0.2719 0 -5.389690 -5.126205 -1.822520 L
C-CT--0.0014 0 -5.329772 -6.482221 -3.518074 L
H-H1-0.0876 0 -5.066879 -6.195748 -4.536664 L
C-C-0.5973 0 -6.481769 -7.488141 -3.571769 L
O-O--0.5679 0 -7.319958 -7.540160 -2.669707 L
C-CT--0.0152 0 -4.145539 -7.242922 -2.903059 L
H-HC-0.0295 0 -3.816877 -8.003503 -3.612409 L
H-HC-0.0295 0 -4.491675 -7.779416 -2.018853 L
C-CA--0.0011 -1 -2.945425 -6.430598 -2.511803 L
C-CA--0.1906 0 -1.950737 -6.083152 -3.448272 L
H-HA-0.1699 0 -2.057510 -6.379111 -4.481594 L
C-CA--0.1906 0 -2.790528 -6.060085 -1.162021 L
H-HA-0.1699 0 -3.545509 -6.326933 -0.433644 L
C-CA--0.2341 0 -0.827319 -5.334460 -3.035619 L
H-HA-0.1656 0 -0.077053 -5.031956 -3.744859 L
C-CA--0.2341 0 -1.664240 -5.330773 -0.746885 L
H-HA-0.1656 0 -1.560759 -5.039621 0.286904 L
C-C-0.3226 0 -0.688111 -4.942936 -1.684262 L
O-OH--0.5579 0 0.360413 -4.173983 -1.287587 L
H-HO-0.3992 0 0.128062 -3.751471 -0.427682 L
N-N--0.4157 0 -6.418371 -8.371063 -4.572026 L
H-H-0.2719 0 -5.706906 -8.225747 -5.269154 L
C-CT--0.1490 -1 -7.184121 -9.602589 -4.612734 L
H-H1-0.0976 0 -8.251279 -9.369397 -4.608281 L
H-H1-0.0976 0 -6.945540 -10.169850 -5.513246 L
H-H1-0.0976 0 -6.959670 -10.208031 -3.732113 L

C-CT--0.3662	-1	-6.308477	-3.334988	-7.033536	L
H-HC-0.1123	0	-5.860282	-4.093945	-7.671262	L
H-HC-0.1123	0	-7.351988	-3.588433	-6.850564	L
H-HC-0.1123	0	-5.769456	-3.281554	-6.094113	L
C-C-0.5972	0	-6.214738	-1.985161	-7.727025	L
O-O--0.5679	0	-5.319507	-1.812387	-8.542266	L
N-N--0.415700	0	-7.108731	-1.038155	-7.406050	L
H-H-0.271900	0	-7.830099	-1.312283	-6.756408	L
C-CT--0.025200	-1	-7.226130	0.291560	-8.055878	L
H-H1-0.069800	0	-8.165954	0.325405	-8.607246	L
H-H1-0.069800	0	-6.424406	0.431338	-8.780340	L
C-C-0.597300	0	-7.209203	1.476759	-7.094547	L
O-O--0.567900	0	-8.111928	2.300663	-7.153710	L
N-N--0.415700	0	-6.191784	1.559214	-6.230913	L
H-H-0.271900	0	-5.498085	0.820599	-6.274614	L
C-CT--0.025200	-1	-5.756928	2.789094	-5.556971	L
H-H1-0.069800	0	-5.593745	2.600307	-4.499927	L
H-H1-0.069800	0	-6.513005	3.568324	-5.664201	L
C-C-0.597300	0	-4.462285	3.301434	-6.197402	L
O-O--0.567900	0	-4.393563	3.443640	-7.415422	L
N-N--0.415700	0	-3.420903	3.517145	-5.390648	L
H-H-0.271900	0	-3.542546	3.358933	-4.400192	L
C-CT--0.051800	-1	-2.078643	3.896813	-5.847093	L
H-H1-0.092200	0	-2.159969	4.357464	-6.833041	L
C-C-0.597300	0	-1.172461	2.657450	-5.987263	L
O-O--0.567900	0	-0.513339	2.441382	-7.000383	L
C-CT--0.110200	0	-1.522185	4.943236	-4.861124	L
H-HC-0.045700	0	-1.486221	4.505477	-3.864109	L
H-HC-0.045700	0	-2.215269	5.785052	-4.826861	L
C-CT-0.353100	0	-0.120758	5.477626	-5.199471	L
H-HC--0.036100	0	0.590533	4.651861	-5.221178	L
C-CT--0.412100	0	-0.077099	6.208381	-6.541388	L
H-HC-0.100000	0	-0.828190	6.999041	-6.560830	L
H-HC-0.100000	0	-0.277151	5.506756	-7.351226	L
H-HC-0.100000	0	0.910469	6.640284	-6.698977	L
C-CT--0.412100	0	0.313574	6.457008	-4.106592	L
H-HC-0.100000	0	-0.391143	7.287722	-4.052336	L
H-HC-0.100000	0	1.307183	6.844159	-4.330534	L
H-HC-0.100000	0	0.341367	5.943385	-3.146797	L
N-N--0.516300	0	-1.119951	1.850092	-4.932418	L
H-H-0.293600	0	-1.676749	2.144623	-4.136723	L
C-CT-0.038100	-1	-0.254583	0.692041	-4.720814	L
H-H1-0.088000	0	-0.040430	0.192739	-5.663172	L
C-C-0.536600	0	-0.978714	-0.261332	-3.753181	L
O-O--0.581900	0	-0.914498	-0.051480	-2.549124	L
C-CT--0.030300	0	1.071694	1.256107	-4.151884	L
H-HC--0.012200	0	0.833956	1.924553	-3.324931	L
H-HC--0.012200	0	1.546802	1.861865	-4.925598	L
C-C-0.799400	-1	2.101047	0.232645	-3.661503	L
O-O2--0.801400	0	1.927012	-0.985801	-3.896079	L
O-O2--0.801400	0	3.117986	0.644642	-3.063392	L
N-N--0.254800	0	-1.741612	-1.258445	-4.233848	L
C-CT--0.026600	-1	-2.016037	-1.558367	-5.635845	L
H-H1-0.064100	0	-1.087895	-1.624597	-6.200992	L
C-C-0.589600	0	-2.943440	-0.520448	-6.295339	L

O-O--0.574800	0	-3.870035	-0.016636	-5.657811	L
C-CT--0.007000	0	-2.679690	-2.938483	-5.605851	L
H-HC-0.025300	0	-1.909840	-3.711018	-5.577725	L
H-HC-0.025300	0	-3.343566	-3.097868	-6.456141	L
C-CT-0.018900	0	-3.437575	-2.929880	-4.278726	L
H-HC-0.021300	0	-3.610568	-3.935475	-3.905484	L
H-HC-0.021300	0	-4.380863	-2.393159	-4.386202	L
C-CT-0.019200	0	-2.505786	-2.141742	-3.364814	L
H-H1-0.039100	0	-3.084525	-1.575578	-2.634798	L
H-H1-0.039100	0	-1.823028	-2.814186	-2.849711	L
N-N--0.4157	0	-2.696157	-0.229995	-7.576807	L
H-H-0.2719	0	-1.914002	-0.681739	-8.018066	L
C-CT--0.1490	-1	-3.474867	0.675317	-8.420451	L
H-H1-0.0976	0	-4.434664	0.896704	-7.967445	L
H-H1-0.0976	0	-3.653428	0.217969	-9.394589	L
H-H1-0.0976	0	-2.932398	1.613564	-8.557776	L
C-CT--0.3662	-1	8.810944	1.943575	-5.853284	L
H-HC-0.1123	0	9.345138	2.232247	-6.758067	L
H-HC-0.1123	0	9.526675	1.628060	-5.097193	L
H-HC-0.1123	0	8.118994	1.135981	-6.083982	L
C-C-0.5972	0	8.042108	3.141558	-5.340163	L
O-O--0.5679	0	8.284514	4.254253	-5.784293	L
N-N--0.415700	0	7.118013	2.924275	-4.403647	L
H-H-0.271900	0	6.981229	1.972761	-4.091252	L
C-CT-0.021300	-1	6.149289	3.937557	-3.958879	L
H-H1-0.112400	0	6.366294	4.890432	-4.446104	L
C-C-0.597300	0	6.184479	4.165269	-2.445602	L
O-O--0.567900	0	6.443344	3.242597	-1.672879	L
C-CT--0.123100	0	4.765131	3.464988	-4.432628	L
H-H1-0.111200	0	4.520131	2.511538	-3.959491	L
H-H1-0.111200	0	4.786806	3.319748	-5.514440	L
S-SH--0.311900	0	3.483080	4.684578	-4.030369	L
H-HS-0.193300	0	2.468904	4.062660	-4.649766	L
N-N--0.415700	0	5.903944	5.393947	-2.013454	L
H-H-0.271900	0	5.655223	6.091871	-2.701620	L
C-CT--0.025200	-1	5.890131	5.789804	-0.610327	L
H-H1-0.069800	0	6.858333	6.222667	-0.359352	L
H-H1-0.069800	0	5.744041	4.922155	0.028627	L
C-C-0.597300	0	4.821422	6.835444	-0.317615	L
O-O--0.567900	0	4.938216	7.983426	-0.736418	L
N-N--0.415700	0	3.805168	6.458352	0.452517	L
H-H-0.271900	0	3.843672	5.511177	0.821783	L
C-CT--0.025200	-1	2.762896	7.331521	0.978944	L
H-H1-0.069800	0	1.933429	6.712993	1.306321	L
H-H1-0.069800	0	2.422400	8.025696	0.210061	L
C-C-0.597300	0	3.251666	8.120305	2.192452	L
O-O--0.567900	0	2.646321	8.043357	3.259273	L
N-N--0.4157	0	4.361133	8.847359	2.023908	L
H-H-0.2719	0	4.738429	8.849452	1.081293	L
C-CT--0.1490	-1	5.099388	9.518198	3.084737	L
H-H1-0.0976	0	5.398788	8.792376	3.842703	L
H-H1-0.0976	0	5.986699	10.002947	2.674854	L
H-H1-0.0976	0	4.463630	10.271758	3.553728	L
C-CT--0.3662	-1	-1.438882	9.448922	5.369340	L
H-HC-0.1123	0	-1.660198	10.427187	4.945586	L

H-HC-0.1123	0	-0.502464	9.076038	4.957495	L
H-HC-0.1123	0	-1.365016	9.533168	6.452050	L
C-C-0.5972	0	-2.572336	8.482985	5.012268	L
O-O--0.5679	0	-3.465168	8.858017	4.262770	L
N-N--0.4157	0	-2.542392	7.242943	5.519515	L
H-H-0.2719	0	-1.788917	7.010619	6.151760	L
C-CT--0.0275	-1	-3.561758	6.174919	5.346697	L
H-H1-0.1123	0	-4.549180	6.635470	5.301211	L
C-C-0.5973	0	-3.539307	5.189922	6.545438	L
O-O--0.5679	0	-2.617505	5.225384	7.359185	L
C-CT--0.0050	0	-3.312360	5.396471	4.044137	L
H-HC-0.0339	0	-3.446327	6.075142	3.209284	L
H-HC-0.0339	0	-4.097054	4.647069	3.943895	L
C-C*--0.1415	0	-1.986304	4.696898	3.895210	L
C-CW--0.1638	0	-0.790058	5.297670	3.685937	L
H-H4-0.2062	0	-0.636971	6.366948	3.611898	L
C-CB-0.1243	0	-1.704261	3.261144	3.931294	L
N-NA--0.3418	0	0.205355	4.342751	3.591164	L
H-H-0.3412	0	1.186761	4.555633	3.462001	L
C-CN-0.1380	0	-0.302582	3.070334	3.733776	L
C-CA--0.238	0	-2.488658	2.099264	4.113642	L
H-HA-0.1700	0	-3.552532	2.202551	4.249496	L
C-CA--0.2601	0	0.292260	1.800016	3.726882	L
H-HA-0.1572	0	1.356042	1.689328	3.567139	L
C-CA--0.1972	0	-1.904252	0.817538	4.095353	L
H-HA-0.1447	0	-2.509404	-0.073085	4.202898	L
C-CA--0.1134	0	-0.520496	0.667244	3.904771	L
H-HA-0.1417	0	-0.094494	-0.327865	3.867134	L
N-N--0.4157	0	-4.537528	4.293856	6.651826	L
H-H-0.2719	0	-5.260657	4.345856	5.953246	L
C-CT--0.1490	-1	-4.641572	3.250665	7.687357	L
H-H1-0.0976	0	-3.786126	2.577246	7.614142	L
H-H1-0.0976	0	-4.637088	3.714394	8.676190	L
H-H1-0.0976	0	-5.563502	2.680728	7.561316	L
C-CT--0.3662	-1	-2.831518	-1.479538	7.246863	L
H-HC-0.1123	0	-3.140665	-1.477575	8.289995	L
H-HC-0.1123	0	-3.324685	-0.676329	6.702000	L
H-HC-0.1123	0	-3.095915	-2.431476	6.789211	L
C-C-0.5972	0	-1.339108	-1.299523	7.164022	L
O-O--0.5679	0	-0.639251	-2.260897	6.897360	L
N-N--0.415700	0	-0.832292	-0.103530	7.460967	L
H-H-0.271900	0	-1.483303	0.653675	7.597866	L
C-CT--0.087500	-1	0.612745	0.214734	7.423883	L
H-H1-0.096900	0	0.939499	0.108282	6.388466	L
C-C-0.597300	0	1.448022	-0.759136	8.265322	L
O-O--0.567900	0	2.443135	-1.288618	7.785850	L
C-CT-0.298500	0	0.904265	1.667353	7.869629	L
H-HC--0.029700	0	0.960780	1.701650	8.958278	L
C-CT--0.319200	0	2.251051	2.129502	7.300215	L
H-HC-0.079100	0	2.209710	2.142357	6.210208	L
H-HC-0.079100	0	3.045728	1.455668	7.621291	L
H-HC-0.079100	0	2.480034	3.132751	7.660387	L
C-CT--0.319200	0	-0.169062	2.677210	7.440473	L
H-HC-0.079100	0	-0.357772	2.590998	6.373944	L
H-HC-0.079100	0	0.160522	3.693670	7.658618	L

H-HC-0.079100	0	-1.096384	2.509573	7.986240	L
N-N--0.415700	0	1.021427	-1.036064	9.505245	L
H-H-0.271900	0	0.199957	-0.547916	9.822783	L
C-CT--0.025200	-1	1.619755	-2.054583	10.384645	L
H-H1-0.069800	0	1.080132	-2.085897	11.330535	L
H-H1-0.069800	0	2.655821	-1.783525	10.591349	L
C-C-0.597300	0	1.616374	-3.479450	9.780889	L
O-O--0.567900	0	2.445328	-4.293002	10.168425	L
N-N--0.415700	0	0.697664	-3.790253	8.852382	L
H-H-0.271900	0	0.100398	-3.047564	8.519285	L
C-CT-0.033700	-1	0.600643	-5.062317	8.120344	L
H-H1-0.082300	0	1.005561	-5.851524	8.757233	L
C-C-0.597300	0	1.437763	-5.085433	6.817367	L
O-O--0.567900	0	2.051294	-6.100868	6.502570	L
C-CT--0.182500	0	-0.874286	-5.397372	7.860049	L
H-HC-0.060300	0	-1.428128	-5.381111	8.799326	L
H-HC-0.060300	0	-0.940279	-6.398458	7.431818	L
H-HC-0.060300	0	-1.312863	-4.683753	7.164688	L
N-N--0.415700	0	1.481852	-3.978420	6.065952	L
H-H-0.271900	0	0.877112	-3.212483	6.337087	L
C-CT-0.033700	-1	2.422749	-3.738311	4.959635	L
H-H1-0.082300	0	2.356724	-4.561828	4.261007	L
C-C-0.597300	0	3.895206	-3.692041	5.433457	L
O-O--0.567900	0	4.816437	-3.849704	4.629055	L
C-CT--0.182500	0	2.034408	-2.413056	4.300838	L
H-HC-0.060300	0	2.245430	-1.580786	4.972726	L
H-HC-0.060300	0	0.976269	-2.415009	4.058958	L
H-HC-0.060300	0	2.605839	-2.292054	3.385655	L
N-N--0.4157	0	4.121732	-3.446238	6.730224	L
H-H-0.2719	0	3.310017	-3.187509	7.274991	L
C-CT--0.1490	-1	5.399045	-3.554723	7.419446	L
H-H1-0.0976	0	5.863387	-4.513846	7.182190	L
H-H1-0.0976	0	5.243079	-3.491139	8.497616	L
H-H1-0.0976	0	6.061241	-2.749587	7.097914	L
C-CT--0.3662	-1	14.117060	0.871883	-0.614161	L
H-HC-0.1123	0	13.465329	1.375553	0.096947	L
H-HC-0.1123	0	15.125093	1.277693	-0.542653	L
H-HC-0.1123	0	13.739255	1.005200	-1.625716	L
C-C-0.5972	0	14.160297	-0.612867	-0.293564	L
O-O--0.5679	0	15.236409	-1.185081	-0.187308	L
N-N--0.4157	0	12.993773	-1.242422	-0.157011	L
H-H-0.2719	0	12.156220	-0.683682	-0.272186	L
C-CT--0.0014	-1	12.805759	-2.695987	0.032758	L
H-H1-0.0876	0	13.683775	-3.217363	-0.351088	L
C-C-0.5973	0	11.578006	-3.172112	-0.795771	L
O-O--0.5679	0	10.834036	-2.360817	-1.355462	L
C-CT--0.0152	0	12.670794	-3.018008	1.533014	L
H-HC-0.0295	0	13.612848	-2.766137	2.022578	L
H-HC-0.0295	0	12.529137	-4.092398	1.654891	L
C-CA--0.0011	-1	11.543321	-2.291275	2.247073	L
C-CA--0.1906	0	11.791469	-1.064004	2.890752	L
H-HA-0.1699	0	12.793626	-0.657500	2.901558	L
C-CA--0.1906	0	10.235214	-2.817800	2.231198	L
H-HA-0.1699	0	10.031809	-3.763484	1.747981	L
C-CA--0.2341	0	10.738202	-0.365683	3.511764	L

H-HA-0.1656	0	10.931233	0.565688	4.012585	L
C-CA--0.2341	0	9.177898	-2.116439	2.843841	L
H-HA-0.1656	0	8.178420	-2.525202	2.827077	L
C-C-0.3226	0	9.425350	-0.884590	3.487630	L
O-OH--0.557	0	8.409549	-0.195710	4.077245	L
H-HO-0.3992	0	7.571297	-0.696571	4.000958	L
N-N--0.415700	0	11.348749	-4.486837	-0.915106	L
H-H-0.271900	0	11.956017	-5.115187	-0.412441	L
C-CT--0.025200	-1	10.237019	-5.065962	-1.697758	L
H-H1-0.069800	0	10.461381	-6.110390	-1.912662	L
H-H1-0.069800	0	10.142164	-4.542363	-2.648281	L
C-C-0.597300	0	8.900816	-5.021680	-0.945615	L
O-O--0.567900	0	8.818402	-5.474490	0.194772	L
N-N--0.415700	0	7.839789	-4.508276	-1.574681	L
H-H-0.271900	0	7.932089	-4.328176	-2.566751	L
C-CT-0.014300	-1	6.504854	-4.455177	-0.980896	L
H-H1-0.104800	0	6.611999	-4.366955	0.103392	L
C-C-0.597300	0	5.710912	-5.726795	-1.239401	L
O-O--0.567900	0	4.921034	-5.787426	-2.185224	L
C-CT--0.204100	0	5.717847	-3.197799	-1.430926	L
H-HC-0.079700	0	5.607691	-3.205115	-2.514777	L
H-HC-0.079700	0	6.296203	-2.331510	-1.143831	L
C-C-0.713000	-1	4.318645	-3.079027	-0.794881	L
O-O--0.593100	0	3.981684	-3.762469	0.161092	L
N-N--0.919100	0	3.431753	-2.278374	-1.348845	L
H-H-0.419600	0	2.526568	-2.290959	-0.903479	L
H-H-0.419600	0	3.582333	-1.706410	-2.170656	L
N-N--0.347900	0	5.904160	-6.713966	-0.358455	L
H-H-0.274700	0	6.612005	-6.517729	0.340035	L
C-CT--0.263700	-1	5.328598	-8.064962	-0.434262	L
H-H1-0.156000	0	5.726738	-8.646330	0.397682	L
C-C-0.734100	0	5.732857	-8.805678	-1.723684	L
O-O--0.589400	0	6.388715	-8.264956	-2.611978	L
C-CT--0.000700	0	3.793430	-8.018604	-0.304586	L
H-HC-0.032700	0	3.403859	-9.037570	-0.293206	L
H-HC-0.032700	0	3.383128	-7.528861	-1.188866	L
C-CT-0.039000	0	3.263033	-7.311070	0.946295	L
H-HC-0.028500	0	3.735984	-6.338595	1.077061	L
H-HC-0.028500	0	3.447860	-7.927507	1.827120	L
C-CT-0.048600	0	1.760910	-7.117383	0.736110	L
H-H1-0.068700	0	1.308839	-8.075976	0.475187	L
H-H1-0.068700	0	1.598366	-6.429690	-0.097663	L
N-N2--0.529500	-1	1.086128	-6.603680	1.928394	L
H-H-0.345600	0	0.375914	-7.189511	2.350676	L
C-CA-0.807600	0	0.992940	-5.363947	2.301845	L
N-N2--0.862700	0	0.024174	-5.084190	3.112947	L
H-H-0.447800	0	-0.663253	-5.805650	3.248319	L
H-H-0.447800	0	-0.270337	-4.105343	3.174525	L
N-N2--0.862700	0	1.780247	-4.429701	1.861130	L
H-H-0.447800	0	2.552538	-4.584882	1.229524	L
H-H-0.447800	0	1.279628	-3.544816	1.800062	L
N-N--0.4157	0	5.289427	-10.055265	-1.857683	L
H-H-0.2719	0	4.761083	-10.440781	-1.091680	L
C-CT--0.1490	-1	5.445046	-10.835242	-3.078513	L
H-H1-0.0976	0	6.478928	-10.781006	-3.427989	L

H-H1-0.0976	0	5.192634	-11.880303	-2.890304	L
H-H1-0.0976	0	4.787664	-10.445224	-3.857561	L
C-CT--0.3662	-1	10.160306	-5.886955	-7.780601	L
H-HC-0.1123	0	9.866633	-6.551514	-8.590767	L
H-HC-0.1123	0	11.078260	-5.368659	-8.056581	L
H-HC-0.1123	0	10.328718	-6.452118	-6.865622	L
C-C-0.5972	0	9.072396	-4.860801	-7.545847	L
O-O--0.5679	0	9.330471	-3.666033	-7.635782	L
N-N--0.347900	0	7.861426	-5.304666	-7.179153	L
H-H-0.274700	0	7.759220	-6.303944	-7.091562	L
C-CT--0.263700	-1	6.882235	-4.419947	-6.515035	L
H-H1-0.156000	0	6.692708	-3.551194	-7.146936	L
C-C-0.734100	0	7.527163	-3.937618	-5.213104	L
O-O--0.589400	0	7.884505	-4.756481	-4.370757	L
C-CT--0.000700	0	5.561981	-5.140917	-6.213839	L
H-HC-0.032700	0	4.971620	-4.470642	-5.591943	L
H-HC-0.032700	0	5.767375	-6.045662	-5.640884	L
C-CT-0.039000	0	4.755993	-5.507219	-7.470364	L
H-HC-0.028500	0	5.237375	-6.355812	-7.957901	L
H-HC-0.028500	0	4.760213	-4.667153	-8.166618	L
C-CT-0.048600	0	3.296228	-5.879496	-7.151105	L
H-H1-0.068700	0	3.261808	-6.616538	-6.350978	L
H-H1-0.068700	0	2.867246	-6.349038	-8.038324	L
N-N2--0.529500	0	2.481433	-4.692403	-6.819497	L
H-H-0.345600	0	2.121756	-4.173764	-7.600627	L
C-CA-0.807600	0	2.215370	-4.163351	-5.635432	L
N-N2--0.862700	0	2.540299	-4.716765	-4.501479	L
H-H-0.447800	0	2.994184	-5.626421	-4.485173	L
H-H-0.447800	0	2.315599	-4.253393	-3.633957	L
N-N2--0.862700	0	1.612190	-3.015144	-5.599589	L
H-H-0.447800	0	1.420401	-2.503830	-6.434328	L
H-H-0.447800	0	1.629201	-2.461225	-4.732581	L
N-N--0.415700	0	7.732694	-2.633203	-5.063481	L
H-H-0.271900	0	7.420442	-2.023231	-5.801544	L
C-CT--0.025200	-1	8.637540	-2.076472	-4.054893	L
H-H1-0.069800	0	9.519587	-1.688609	-4.565280	L
H-H1-0.069800	0	8.978526	-2.847575	-3.369112	L
C-C-0.597300	0	8.047873	-0.945820	-3.231688	L
O-O--0.567900	0	6.997005	-0.393543	-3.559288	L
N-N--0.415700	0	8.735023	-0.578528	-2.153146	L
H-H-0.271900	0	9.585764	-1.080455	-1.902583	L
C-CT--0.038900	-1	8.335809	0.554107	-1.322883	L
H-H1-0.100700	0	7.720148	1.196494	-1.949361	L
C-C-0.597300	0	9.479918	1.465258	-0.902328	L
O-O--0.567900	0	10.579154	1.033976	-0.558106	L
C-CT-0.365400	0	7.428898	0.118896	-0.157784	L
H-H1-0.004300	0	7.731264	-0.868611	0.193703	L
O-OH--0.676100	0	6.120610	0.068044	-0.686954	L
H-HO-0.410200	0	6.202376	-0.156636	-1.626546	L
C-CT--0.243800	0	7.339750	1.064238	1.040153	L
H-HC-0.064200	0	6.998361	2.049095	0.723853	L
H-HC-0.064200	0	8.312015	1.145907	1.524626	L
H-HC-0.064200	0	6.631027	0.659943	1.763085	L
N-N--0.415700	0	9.171541	2.761523	-0.960558	L
H-H-0.271900	0	8.210302	2.978274	-1.204185	L

C-CT--0.025200	-1	10.012382	3.880466	-0.581712 L
H-H1-0.069800	0	9.824321	4.713447	-1.259052 L
H-H1-0.069800	0	11.062315	3.595381	-0.667529 L
C-C-0.597300	0	9.710514	4.339052	0.832885 L
O-O--0.567900	0	8.561179	4.542446	1.237888 L
N-N--0.347900	0	10.783614	4.537175	1.583314 L
H-H-0.274700	0	11.690315	4.387215	1.155457 L
C-CT--0.263700	-1	10.791623	5.117952	2.916894 L
H-H1-0.156000	0	9.890669	5.709047	3.069968 L
C-C-0.734100	0	11.969347	6.062537	3.033920 L
O-O--0.589400	0	12.921411	5.979666	2.263498 L
C-CT--0.000700	0	10.928329	4.003549	3.976288 L
H-HC-0.032700	0	11.205062	4.439764	4.937731 L
H-HC-0.032700	0	11.735545	3.329765	3.681122 L
C-CT-0.039000	0	9.654858	3.182009	4.182746 L
H-HC-0.028500	0	9.878194	2.355346	4.856783 L
H-HC-0.028500	0	9.317248	2.769625	3.231553 L
C-CT-0.048600	0	8.549280	4.024974	4.820654 L
H-H1-0.068700	0	8.361316	4.921446	4.230758 L
H-H1-0.068700	0	8.865454	4.332857	5.819019 L
N-N2--0.529500	-1	7.317970	3.242551	4.924472 L
H-H-0.345600	0	7.227047	2.613574	5.703374 L
C-CA-0.807600	0	6.338453	3.185043	4.047322 L
N-N2--0.862700	0	5.318525	2.412854	4.261051 L
H-H-0.447800	0	5.305364	1.734077	4.995584 L
H-H-0.447800	0	4.682468	2.291634	3.466219 L
N-N2--0.862700	0	6.350708	3.875200	2.949421 L
H-H-0.447800	0	7.195419	4.281066	2.573261 L
H-H-0.447800	0	5.496260	3.870653	2.391931 L
N-N--0.4157	0	11.888766	6.935464	4.029092 L
H-H-0.2719	0	11.068002	6.923133	4.610335 L
C-CT--0.1490	-1	12.960812	7.845644	4.341681 L
H-H1-0.0976	0	12.694477	8.468870	5.196903 L
H-H1-0.0976	0	13.869154	7.285993	4.577076 L
H-H1-0.0976	0	13.165915	8.488738	3.482526 L
C-CT--0.3662	-1	-2.034012	13.334487	-1.413524 L
H-HC-0.1123	0	-2.446801	13.838486	-0.542111 L
H-HC-0.1123	0	-0.947300	13.307717	-1.363375 L
H-HC-0.1123	0	-2.336741	13.868963	-2.313723 L
C-C-0.5972	0	-2.573938	11.917969	-1.487751 L
O-O--0.5679	0	-3.233736	11.564412	-2.452266 L
N-N--0.415700	0	-2.301232	11.116488	-0.454742 L
H-H-0.271900	0	-1.733027	11.520437	0.270712 L
C-CT--0.023700	-1	-2.735621	9.711668	-0.299340 L
H-H1-0.088000	0	-2.268164	9.122278	-1.089719 L
C-C-0.597300	0	-4.255806	9.541206	-0.467372 L
O-O--0.567900	0	-4.720757	8.766224	-1.303119 L
C-CT-0.034200	0	-2.206426	9.218354	1.054591 L
H-HC-0.024100	0	-2.584861	9.864120	1.848150 L
H-HC-0.024100	0	-1.118542	9.293429	1.054628 L
C-CT-0.001800	0	-2.593060	7.779819	1.384779 L
H-H1-0.044000	0	-3.664903	7.754100	1.584575 L
H-H1-0.044000	0	-2.076656	7.506921	2.302128 L
S-S--0.273700	0	-2.233344	6.522226	0.134771 L
C-CT--0.053600	0	-0.433114	6.538629	0.083103 L

H-H1-0.068400	0	-0.090813	5.872006	-0.707929	L
H-H1-0.068400	0	-0.056453	6.188171	1.041969	L
H-H1-0.068400	0	-0.074579	7.546611	-0.122207	L
N-N--0.4157	0	-5.037299	10.278005	0.326660	L
H-H-0.2719	0	-4.578774	10.886398	0.985452	L
C-CT--0.1490	-1	-6.487512	10.307156	0.221366	L
H-H1-0.0976	0	-6.907047	10.965022	0.983762	L
H-H1-0.0976	0	-6.775378	10.673267	-0.766485	L
H-H1-0.0976	0	-6.887413	9.300405	0.355262	L
C-CT--0.3662	-1	-4.329440	9.125777	-5.064410	L
H-HC-0.1123	0	-3.905709	9.469668	-4.121644	L
H-HC-0.1123	0	-3.558603	9.130072	-5.832993	L
H-HC-0.1123	0	-5.147218	9.776082	-5.366383	L
C-C-0.5972	0	-4.853538	7.706122	-4.902116	L
O-O--0.5679	0	-4.556148	6.850018	-5.722848	L
N-N--0.415700	0	-5.642457	7.460145	-3.852408	L
H-H-0.271900	0	-5.670999	8.203023	-3.165970	L
C-CT--0.087500	-1	-6.310469	6.168498	-3.537776	L
H-H1-0.096900	0	-6.026854	5.428621	-4.285633	L
C-C-0.597300	0	-7.823772	6.334463	-3.635223	L
O-O--0.567900	0	-8.467905	5.649828	-4.420131	L
C-CT-0.298500	0	-5.859345	5.660060	-2.159857	L
H-HC--0.029700	0	-6.068261	6.415958	-1.402351	L
C-CT--0.319200	0	-6.541196	4.358497	-1.735479	L
H-HC-0.079100	0	-6.336691	3.589500	-2.474460	L
H-HC-0.079100	0	-7.616017	4.505452	-1.640954	L
H-HC-0.079100	0	-6.157261	4.032652	-0.769503	L
C-CT--0.319200	0	-4.358773	5.388658	-2.196282	L
H-HC-0.079100	0	-4.123483	4.681388	-2.986909	L
H-HC-0.079100	0	-4.058852	4.981240	-1.240426	L
H-HC-0.079100	0	-3.806946	6.312129	-2.368768	L
N-N--0.4157	0	-8.400720	7.252299	-2.851537	L
H-H-0.2719	0	-7.787961	7.786020	-2.255683	L
C-CT--0.1490	-1	-9.829042	7.549789	-2.884014	L
H-H1-0.0976	0	-10.070590	8.333315	-2.165251	L
H-H1-0.0976	0	-10.110196	7.879987	-3.886483	L
H-H1-0.0976	0	-10.397633	6.648687	-2.646033	L
C-CT-0.1200	-1	-8.848399	2.051301	3.218696	L
H-HC-0.0800	0	-8.211017	2.934479	3.222457	L
H-HC-0.0800	0	-9.674090	2.211271	2.525571	L
H-HC-0.0800	0	-9.250356	1.888049	4.219157	L
C-CT-0.2000	0	-8.069429	0.846537	2.794119	L
H-H1-0.0800	0	-8.735438	-0.020688	2.751109	L
O-OS--0.5600	0	-7.540969	1.145978	1.529460	L
C-CT-0.2000	0	-6.845578	0.545734	3.657710	L
H-H1-0.0800	0	-6.245721	1.441859	3.811548	L
O-OH--0.6800	0	-7.150336	-0.059056	4.902696	L
H-HO-0.4000	0	-7.419717	-0.964639	4.690017	L
C-CT-0.2000	0	-6.157229	-0.400095	2.674839	L
H-H1-0.0800	0	-5.090265	-0.489430	2.890754	L
O-OH--0.6800	0	-6.816985	-1.666855	2.817906	L
H-HO-0.4000	0	-6.548240	-2.236225	2.060685	L
C-CT-0.5691	0	-6.395039	0.325461	1.330030	H
H-H2-0.8000	0	-6.566573	-0.406501	0.521216	H
N-N*--0.5691	0	-5.290847	1.214789	0.986055	H

C-CM--0.0500	0	-4.694839	1.015500	-0.215892	H
H-H4-0.1500	0	-5.087286	0.177709	-0.742472	H
C-CM--0.1238	0	-3.741550	1.794867	-0.734126	H
C-C-0.6156	0	-3.452469	1.785509	-2.178860	H
O-O--0.5700	0	-2.642860	2.535972	-2.667028	H
N-N--0.8000	0	-4.218272	0.990744	-2.988601	H
H-H-0.3700	0	-3.860244	0.771664	-3.897101	H
H-H-0.3700	0	-4.812374	0.284017	-2.607475	H
C-CT-0.1164	0	-3.068682	2.789950	0.182774	H
H-HC-0.0800	0	-2.639201	3.602788	-0.383976	H
H-HC-0.0800	0	-2.240805	2.323787	0.716002	H
C-CM--0.2882	0	-4.098955	3.267189	1.176817	H
H-HA-0.1500	0	-4.018934	4.261545	1.570285	H
C-CM--0.0500	0	-5.090162	2.482819	1.558808	H
H-H4-0.1500	0	-5.819654	2.811624	2.266848	H
N-N3--0.8530	0	3.443871	1.017756	-0.427984	H
H-H-0.4500	0	3.368129	0.744009	-1.396550	H
H-H-0.4500	0	2.707944	0.616656	0.177246	H
H-H-0.4500	0	4.336395	0.719243	-0.060745	H
C-CT-0.3170	0	3.507755	2.484497	-0.319623	H
H-HP-0.0800	0	4.343562	2.800730	-0.924420	H
C-CT--0.1600	0	2.240437	3.208241	-0.759645	H
H-HC-0.0800	0	2.086876	3.027581	-1.816425	H
H-HC-0.0800	0	2.469093	4.261648	-0.659762	H
C-CT--0.1600	0	0.976816	2.870240	0.038287	H
H-HC-0.0800	0	0.249532	3.643164	-0.156622	H
H-HC-0.0800	0	1.211436	2.932365	1.097761	H
C-CT--0.0990	0	0.357479	1.499047	-0.310889	H
H-HC-0.0800	0	0.883983	1.054570	-1.149447	H
H-HC-0.0800	0	-0.652723	1.662681	-0.637789	H
C-C-0.4490	0	0.425004	0.507221	0.841835	H
H-H-0.0600	0	-0.185360	0.781782	1.696085	H
O-O--0.5700	0	1.456730	-0.157632	1.027560	H
N-N3--0.9900	0	-0.982417	-0.748821	0.175055	H
H-H-0.3600	0	-0.395931	-1.430845	-0.265195	H
H-H-0.3600	0	-1.466879	-0.276340	-0.580295	H
C-C-0.9060	0	3.875701	2.771806	1.130827	L
O-O2--0.9000	0	3.973560	1.856151	1.980245	L
O-O2--0.9000	0	4.087043	3.948740	1.481032	L
C-CT--0.205900	0	-1.893291	-1.444462	1.089575	H
H-H1-0.139900	0	-2.320672	-0.712208	1.763953	H
C-CT-0.007100	0	-2.993635	-2.165393	0.303894	H
H-HC--0.007800	0	-2.536981	-2.782781	-0.458406	H
H-HC--0.007800	0	-3.635507	-1.441790	-0.190339	H
C-CT-0.067500	0	-3.857195	-3.072597	1.181338	L
H-HC--0.054800	0	-4.052952	-2.567651	2.131090	L
H-HC--0.054800	0	-3.336506	-4.007974	1.394636	L
C-C-0.818300	0	-5.196608	-3.352916	0.509529	L
O-O2--0.822000	0	-6.039576	-2.419276	0.527498	L
O-O2--0.822000	0	-5.467685	-4.456634	-0.009110	L
C-C-0.742000	0	-1.038432	-2.486469	1.814844	H
O-O2--0.793000	0	-1.233584	-2.687930	3.006242	H
O-O2--0.793000	0	-0.269046	-3.065526	1.037278	H
O-OW--0.834000	0	4.000000	-7.240713	-4.107310	L
H-HW-0.417000	0	4.320898	-6.702829	-3.349684	L

H-HW-0.417000	0	4.692671	-7.906769	-4.171861	L
O-OW--0.834000	0	-1.570541	-7.466631	2.821916	L
H-HW-0.417000	0	-2.265954	-7.587106	2.164871	L
H-HW-0.417000	0	-2.052165	-7.519377	3.662854	L
O-OW--0.834000	0	4.078314	-0.677876	2.135831	L
H-HW-0.417000	0	3.162417	-0.900741	1.917583	L
H-HW-0.417000	0	4.055864	0.309600	2.158737	L
O-OW--0.834000	0	6.009031	-1.464036	3.859713	L
H-HW-0.417000	0	5.698116	-2.342284	4.146260	L
H-HW-0.417000	0	5.334062	-1.216450	3.192870	L
O-OW--0.834000	0	-6.017215	-7.065445	0.032908	L
H-HW-0.417000	0	-5.864598	-6.099287	0.077759	L
H-HW-0.417000	0	-6.647739	-7.168454	-0.689995	L
O-OW--0.834000	0	-8.744036	-0.750340	-0.101523	L
H-HW-0.417000	0	-9.674244	-0.584805	0.080692	L
H-HW-0.417000	0	-8.719770	-1.727114	-0.226996	L

I1

C-CT--0.3662	-1	-12.872538	1.010981	1.930043	L
H-HC-0.1123	0	-12.284625	1.879494	1.636442	L
H-HC-0.1123	0	-13.929886	1.237453	1.803248	L
H-HC-0.1123	0	-12.594097	0.157499	1.315711	L
C-C-0.5972	0	-12.600723	0.696565	3.388330	L
O-O--0.5679	0	-11.846256	1.419563	4.028434	L
N-N--0.415700	0	-13.191265	-0.388981	3.898611	L
H-H-0.271900	0	-13.788373	-0.913690	3.280529	L
C-CT--0.025200	-1	-12.902121	-0.950291	5.231452	L
H-H1-0.069800	0	-13.639431	-1.717779	5.466202	L
H-H1-0.069800	0	-12.967849	-0.157501	5.977313	L
C-C-0.597300	0	-11.478756	-1.582445	5.308674	L
O-O--0.567900	0	-10.643574	-1.409668	4.414468	L
N-N--0.415700	0	-11.184461	-2.349117	6.370167	L
H-H-0.271900	0	-11.904435	-2.485782	7.061913	L
C-CT--0.025200	-1	-9.987694	-3.217418	6.446879	L
H-H1-0.069800	0	-9.902624	-3.637213	7.448866	L
H-H1-0.069800	0	-9.093140	-2.632504	6.229146	L
C-C-0.597300	0	-10.116542	-4.363423	5.451928	L
O-O--0.567900	0	-11.155938	-5.015479	5.382442	L
N-N--0.4157	0	-9.060603	-4.600592	4.683539	L
H-H-0.2719	0	-8.229249	-4.054212	4.833799	L
C-CT--0.0014	-1	-8.998379	-5.561003	3.583785	L
H-H1-0.0876	0	-9.910668	-5.495680	2.989949	L
C-C-0.5973	0	-8.857535	-7.026620	4.054049	L
O-O--0.5679	0	-8.041845	-7.794013	3.550101	L
C-CT--0.0152	0	-7.812312	-5.151521	2.693623	L
H-HC-0.0295	0	-8.063808	-4.192749	2.259482	L
H-HC-0.0295	0	-7.758278	-5.845224	1.854980	L
C-CA--0.0011	0	-6.426212	-5.043948	3.341705	L
C-CA--0.1906	0	-5.471037	-6.057602	3.126155	L
H-HA-0.1699	0	-5.751864	-6.947579	2.575772	L
C-CA--0.1906	0	-6.046351	-3.899272	4.077853	L
H-HA-0.1699	0	-6.750716	-3.111948	4.284084	L
C-CA--0.2341	0	-4.146678	-5.911285	3.585515	L
H-HA-0.1656	0	-3.412953	-6.675879	3.387418	L
C-CA--0.2341	0	-4.717642	-3.733070	4.512395	L

H-HA-0.1656	0	-4.405481	-2.832649	5.016980	L
C-C-0.3326	0	-3.759007	-4.733117	4.254261	L
O-OH--0.5579	0	-2.462828	-4.534517	4.606734	L
H-HO-0.3992	0	-2.205708	-3.628899	4.313043	L
N-N--0.4157	0	-9.662875	-7.434640	5.035523	L
H-H-0.2719	0	-10.359585	-6.770986	5.354055	L
C-CT--0.1490	-1	-9.568645	-8.750192	5.645743	L
H-H1-0.0976	0	-8.569776	-8.893033	6.062508	L
H-H1-0.0976	0	-10.310774	-8.851845	6.438329	L
H-H1-0.0976	0	-9.741917	-9.518298	4.889167	L
C-CT--0.3662	-1	-11.999394	-1.481260	-3.480123	L
H-HC-0.1123	0	-12.520669	-0.570535	-3.770739	L
H-HC-0.1123	0	-12.004166	-1.597325	-2.398638	L
H-HC-0.1123	0	-12.493375	-2.337741	-3.937994	L
C-C-0.5972	0	-10.568658	-1.425511	-3.979564	L
O-O--0.5679	0	-10.107771	-2.285121	-4.718057	L
N-N--0.415700	0	-9.845183	-0.413709	-3.522518	L
H-H-0.271900	0	-10.335450	0.255049	-2.945132	L
C-CT--0.038900	-1	-8.403210	-0.127311	-3.687064	L
H-H1-0.100700	0	-8.110626	-0.156191	-4.734639	L
C-C-0.597300	0	-7.554009	-1.105541	-2.875456	L
O-O--0.567900	0	-6.751298	-0.709062	-2.026428	L
C-CT-0.365400	0	-8.220112	1.299823	-3.119199	L
H-H1-0.004300	0	-7.162062	1.551753	-3.118182	L
O-OH--0.676100	0	-8.743961	1.406239	-1.804269	L
H-HO-0.410200	0	-8.667169	0.554074	-1.346890	L
C-CT--0.243800	0	-9.002050	2.372191	-3.875057	L
H-HC-0.064200	0	-10.060461	2.128271	-3.945817	L
H-HC-0.064200	0	-8.602607	2.503725	-4.870915	L
H-HC-0.064200	0	-8.915210	3.317721	-3.347387	L
N-N--0.415700	0	-7.777711	-2.397606	-3.073458	L
H-H-0.271900	0	-8.449811	-2.661346	-3.790304	L
C-CT--0.024900	-1	-7.191904	-3.473998	-2.283413	L
H-H1-0.084300	0	-6.312739	-3.103405	-1.779693	L
C-C-0.597300	0	-6.696125	-4.608949	-3.183197	L
O-O--0.567900	0	-7.207633	-4.795684	-4.284881	L
C-CT-0.211700	0	-8.196577	-4.070726	-1.299952	L
H-H1-0.035200	0	-7.900249	-5.084011	-1.024614	L
H-H1-0.035200	0	-9.197418	-4.093569	-1.734200	L
O-OH--0.654600	0	-8.163380	-3.271812	-0.152475	L
H-HO-0.427500	0	-7.206332	-3.202670	0.061303	L
N-N--0.4157	0	-5.711100	-5.385914	-2.721209	L
H-H-0.2719	0	-5.318609	-5.162616	-1.807583	L
C-CT--0.0014	0	-5.311288	-6.625994	-3.397089	L
H-H1-0.0876	0	-4.999238	-6.380736	-4.412303	L
C-C-0.5973	0	-6.457712	-7.636869	-3.464323	L
O-O--0.5679	0	-7.334452	-7.656201	-2.598901	L
C-CT--0.0152	0	-4.161066	-7.340722	-2.677611	L
H-HC-0.0295	0	-3.875267	-8.211838	-3.268706	L
H-HC-0.0295	0	-4.515115	-7.730868	-1.725173	L
C-CA--0.0011	-1	-2.913191	-6.541342	-2.432682	L
C-CA--0.1906	0	-1.961852	-6.382450	-3.461398	L
H-HA-0.1699	0	-2.166812	-6.770470	-4.448775	L
C-CA--0.1906	0	-2.626698	-6.061513	-1.139986	L
H-HA-0.1699	0	-3.345521	-6.197281	-0.340055	L

C-CA--0.2341	0	-0.730377	-5.749926	-3.196661	L
H-HA-0.1656	0	0.007713	-5.634863	-3.974947	L
C-CA--0.2341	0	-1.399139	-5.428452	-0.870742	L
H-HA-0.1656	0	-1.198940	-5.068847	0.127452	L
C-C-0.3226	0	-0.444129	-5.278542	-1.897636	L
O-OH--0.5579	0	0.759998	-4.707060	-1.628450	L
H-HO-0.3992	0	0.721693	-4.308174	-0.751050	L
N-N--0.4157	0	-6.354272	-8.549437	-4.434228	L
H-H-0.2719	0	-5.609561	-8.429931	-5.100745	L
C-CT--0.1490	-1	-7.132510	-9.772480	-4.481853	L
H-H1-0.0976	0	-8.194565	-9.527079	-4.551645	L
H-H1-0.0976	0	-6.845851	-10.373126	-5.345948	L
H-H1-0.0976	0	-6.971045	-10.349845	-3.569044	L
C-CT--0.3662	-1	-6.325736	-3.521438	-6.968509	L
H-HC-0.1123	0	-5.819459	-4.269565	-7.575024	L
H-HC-0.1123	0	-7.367873	-3.809962	-6.836327	L
H-HC-0.1123	0	-5.840189	-3.444210	-6.000981	L
C-C-0.5972	0	-6.244429	-2.174178	-7.669470	L
O-O--0.5679	0	-5.357171	-2.001229	-8.493232	L
N-N--0.415700	0	-7.141145	-1.229490	-7.347547	L
H-H-0.271900	0	-7.857398	-1.504218	-6.692623	L
C-CT--0.025200	-1	-7.281381	0.086296	-8.022136	L
H-H1-0.069800	0	-8.231648	0.099698	-8.556247	L
H-H1-0.069800	0	-6.496059	0.213917	-8.766683	L
C-C-0.597300	0	-7.254225	1.299485	-7.098866	L
O-O--0.567900	0	-8.130739	2.145948	-7.210373	L
N-N--0.415700	0	-6.255501	1.383352	-6.213966	L
H-H-0.271900	0	-5.570726	0.635428	-6.230737	L
C-CT--0.025200	-1	-5.823357	2.621738	-5.555060	L
H-H1-0.069800	0	-5.646591	2.438878	-4.498090	L
H-H1-0.069800	0	-6.585075	3.395852	-5.659878	L
C-C-0.597300	0	-4.536009	3.136872	-6.208806	L
O-O--0.567900	0	-4.471079	3.263072	-7.428655	L
N-N--0.415700	0	-3.494161	3.372242	-5.408261	L
H-H-0.271900	0	-3.614805	3.229865	-4.415934	L
C-CT--0.051800	-1	-2.156783	3.760004	-5.873754	L
H-H1-0.092200	0	-2.241805	4.213551	-6.862752	L
C-C-0.597300	0	-1.237714	2.526979	-5.997551	L
O-O--0.567900	0	-0.552453	2.321069	-6.996118	L
C-CT--0.110200	0	-1.610561	4.812705	-4.888915	L
H-HC-0.045700	0	-1.579515	4.376635	-3.890169	L
H-HC-0.045700	0	-2.307566	5.651508	-4.860913	L
C-CT-0.353100	0	-0.209688	5.352593	-5.221397	L
H-HC--0.036100	0	0.505251	4.529933	-5.238101	L
C-CT--0.412100	0	-0.163428	6.080041	-6.564985	L
H-HC-0.100000	0	-0.916454	6.868784	-6.588762	L
H-HC-0.100000	0	-0.359191	5.376107	-7.373807	L
H-HC-0.100000	0	0.823464	6.514389	-6.720152	L
C-CT--0.412100	0	0.216167	6.335833	-4.128895	L
H-HC-0.100000	0	-0.491313	7.164571	-4.080029	L
H-HC-0.100000	0	1.209805	6.725296	-4.348766	L
H-HC-0.100000	0	0.240598	5.824579	-3.167808	L
N-N--0.516300	0	-1.193979	1.716536	-4.942948	L
H-H-0.293600	0	-1.769425	1.996091	-4.155071	L
C-CT-0.038100	-1	-0.298153	0.583301	-4.724708	L

H-H1-0.088000	0	-0.060972	0.088946	-5.664447	L
C-C-0.536600	0	-0.966215	-0.410810	-3.734697	L
O-O--0.581900	0	-0.801010	-0.280017	-2.519900	L
C-CT--0.030300	0	1.007674	1.175534	-4.119304	L
H-HC--0.012200	0	0.744987	1.796467	-3.263517	L
H-HC--0.012200	0	1.466589	1.828968	-4.863380	L
C-C-0.799400	-1	2.066553	0.155906	-3.672248	L
O-O2--0.801400	0	1.920654	-1.036143	-4.028549	L
O-O2--0.801400	0	3.127259	0.549343	-3.143937	L
N-N--0.254800	0	-1.751598	-1.395421	-4.208868	L
C-CT--0.026600	-1	-2.043212	-1.692018	-5.609036	L
H-H1-0.064100	0	-1.118462	-1.746434	-6.182253	L
C-C-0.589600	0	-2.987058	-0.667992	-6.266135	L
O-O--0.574800	0	-3.915454	-0.170115	-5.625760	L
C-CT--0.007000	0	-2.685898	-3.082814	-5.582328	L
H-HC-0.025300	0	-1.904399	-3.844313	-5.588049	L
H-HC-0.025300	0	-3.371449	-3.239779	-6.415776	L
C-CT-0.018900	0	-3.405924	-3.108217	-4.235555	L
H-HC-0.021300	0	-3.556941	-4.123096	-3.876990	L
H-HC-0.021300	0	-4.357002	-2.578893	-4.306535	L
C-CT-0.019200	0	-2.452974	-2.328240	-3.336784	L
H-H1-0.039100	0	-3.013403	-1.800777	-2.565597	L
H-H1-0.039100	0	-1.732962	-3.004789	-2.876883	L
N-N--0.4157	0	-2.749375	-0.387480	-7.552378	L
H-H-0.2719	0	-1.971167	-0.849052	-7.990625	L
C-CT--0.1490	-1	-3.535551	0.500593	-8.408490	L
H-H1-0.0976	0	-4.497830	0.717753	-7.958075	L
H-H1-0.0976	0	-3.709811	0.029164	-9.376693	L
H-H1-0.0976	0	-3.002097	1.442122	-8.557376	L
C-CT--0.3662	-1	8.750090	1.906126	-5.912999	L
H-HC-0.1123	0	9.251342	2.163731	-6.845602	L
H-HC-0.1123	0	9.495724	1.672395	-5.155962	L
H-HC-0.1123	0	8.094261	1.053651	-6.077977	L
C-C-0.5972	0	7.933200	3.094324	-5.456061	L
O-O--0.5679	0	8.084551	4.180029	-5.996601	L
N-N--0.415700	0	7.068313	2.897646	-4.460685	L
H-H-0.271900	0	6.994472	1.963815	-4.081399	L
C-CT-0.021300	-1	6.079239	3.894430	-4.025581	L
H-H1-0.112400	0	6.275321	4.847504	-4.521214	L
C-C-0.597300	0	6.112500	4.134254	-2.513723	L
O-O--0.567900	0	6.365671	3.216402	-1.733010	L
C-CT--0.123100	0	4.704545	3.386851	-4.490112	L
H-H1-0.111200	0	4.499492	2.416882	-4.032542	L
H-H1-0.111200	0	4.716624	3.263699	-5.574727	L
S-SH--0.311900	0	3.386302	4.551622	-4.043157	L
H-HS-0.193300	0	2.383124	3.902880	-4.652435	L
N-N--0.415700	0	5.835811	5.368151	-2.093473	L
H-H-0.271900	0	5.591926	6.061497	-2.787780	L
C-CT--0.025200	-1	5.818911	5.777308	-0.694246	L
H-H1-0.069800	0	6.784148	6.219298	-0.447783	L
H-H1-0.069800	0	5.680073	4.915064	-0.046634	L
C-C-0.597300	0	4.743490	6.819509	-0.410830	L
O-O--0.567900	0	4.851184	7.964384	-0.840365	L
N-N--0.415700	0	3.731272	6.441740	0.363590	L
H-H-0.271900	0	3.780358	5.498852	0.741007	L

C-CT--0.025200	-1	2.685210	7.306122	0.894752	L
H-H1-0.069800	0	1.864061	6.680829	1.229920	L
H-H1-0.069800	0	2.333459	7.995780	0.126896	L
C-C-0.597300	0	3.177465	8.100547	2.102606	L
O-O--0.567900	0	2.589822	8.008094	3.178150	L
N-N--0.4157	0	4.270465	8.848653	1.918298	L
H-H-0.2719	0	4.635887	8.855269	0.971025	L
C-CT--0.1490	-1	5.011470	9.534748	2.967636	L
H-H1-0.0976	0	5.334078	8.815878	3.722731	L
H-H1-0.0976	0	5.884499	10.033973	2.544639	L
H-H1-0.0976	0	4.369214	10.278059	3.444047	L
C-CT--0.3662	-1	-1.515047	9.428515	5.284191	L
H-HC-0.1123	0	-1.725082	10.391304	4.821150	L
H-HC-0.1123	0	-0.574750	9.036034	4.900547	L
H-HC-0.1123	0	-1.456228	9.552844	6.363980	L
C-C-0.5972	0	-2.648636	8.455742	4.947818	L
O-O--0.5679	0	-3.532497	8.807921	4.176935	L
N-N--0.4157	0	-2.621705	7.231486	5.490920	L
H-H-0.2719	0	-1.872733	7.017928	6.135254	L
C-CT--0.0275	-1	-3.607927	6.135236	5.304028	L
H-H1-0.1123	0	-4.608155	6.565600	5.246775	L
C-C-0.5973	0	-3.567597	5.154089	6.505215	L
O-O--0.5679	0	-2.645638	5.208527	7.317763	L
C-CT--0.0050	0	-3.312485	5.379509	4.001908	L
H-HC-0.0339	0	-3.507698	6.042699	3.167081	L
H-HC-0.0339	0	-4.036006	4.570000	3.913016	L
C-C*--0.1415	0	-1.936050	4.790382	3.853871	L
C-CW--0.1638	0	-0.782953	5.470991	3.642747	L
H-H4-0.2062	0	-0.699715	6.546393	3.555690	L
C-CB-0.1243	0	-1.553427	3.385527	3.929255	L
N-NA--0.3418	0	0.278809	4.587930	3.578145	L
H-H-0.3412	0	1.244509	4.868981	3.460208	L
C-CN-0.1380	0	-0.139644	3.288166	3.757952	L
C-CA--0.238	0	-2.263020	2.184922	4.141575	L
H-HA-0.1700	0	-3.333952	2.215851	4.259358	L
C-CA--0.2601	0	0.539518	2.062554	3.801678	L
H-HA-0.1572	0	1.609476	2.017619	3.658305	L
C-CA--0.1972	0	-1.592221	0.951211	4.188042	L
H-HA-0.1447	0	-2.148711	0.035777	4.334333	L
C-CA--0.1134	0	-0.198985	0.887513	4.018744	L
H-HA-0.1417	0	0.293461	-0.075663	4.026587	L
N-N--0.4157	0	-4.554898	4.247767	6.622160	L
H-H-0.2719	0	-5.277671	4.280939	5.922111	L
C-CT--0.1490	-1	-4.649863	3.224584	7.678565	L
H-H1-0.0976	0	-3.832640	2.509780	7.569031	L
H-H1-0.0976	0	-4.567931	3.700404	8.658370	L
H-H1-0.0976	0	-5.603416	2.698473	7.613930	L
C-CT--0.3662	-1	-2.798649	-1.493057	7.276037	L
H-HC-0.1123	0	-3.025420	-2.550741	7.141658	L
H-HC-0.1123	0	-3.157234	-1.163380	8.248453	L
H-HC-0.1123	0	-3.274385	-0.926530	6.477634	L
C-C-0.5972	0	-1.306485	-1.304564	7.185312	L
O-O--0.5679	0	-0.591034	-2.262022	6.946654	L
N-N--0.415700	0	-0.811439	-0.096863	7.453375	L
H-H-0.271900	0	-1.471804	0.656818	7.559778	L

C-CT--0.087500	-1	0.630743	0.234205	7.419797	L
H-H1-0.096900	0	0.964081	0.136539	6.386582	L
C-C-0.597300	0	1.475704	-0.732816	8.260048	L
O-O--0.567900	0	2.464937	-1.270763	7.774741	L
C-CT-0.298500	0	0.907188	1.685967	7.880384	L
H-HC--0.029700	0	0.956791	1.709380	8.969641	L
C-CT--0.319200	0	2.255642	2.164644	7.328879	L
H-HC-0.079100	0	2.225350	2.186527	6.239126	L
H-HC-0.079100	0	3.053036	1.494881	7.651597	L
H-HC-0.079100	0	2.472260	3.166644	7.699830	L
C-CT--0.319200	0	-0.170961	2.694864	7.462127	L
H-HC-0.079100	0	-0.349304	2.629661	6.392868	L
H-HC-0.079100	0	0.148039	3.709318	7.703580	L
H-HC-0.079100	0	-1.101933	2.507910	7.995171	L
N-N--0.415700	0	1.063724	-0.993336	9.508462	L
H-H-0.271900	0	0.241274	-0.505518	9.824454	L
C-CT--0.025200	-1	1.672442	-1.996409	10.397919	L
H-H1-0.069800	0	1.127785	-2.028935	11.340893	L
H-H1-0.069800	0	2.703082	-1.708311	10.608173	L
C-C-0.597300	0	1.693287	-3.425746	9.804821	L
O-O--0.567900	0	2.526447	-4.229720	10.204932	L
N-N--0.415700	0	0.790370	-3.748826	8.866889	L
H-H-0.271900	0	0.179291	-3.017548	8.530914	L
C-CT-0.033700	-1	0.670265	-5.035580	8.168303	L
H-H1-0.082300	0	1.127369	-5.804943	8.793508	L
C-C-0.597300	0	1.404259	-5.092243	6.804551	L
O-O--0.567900	0	1.838031	-6.164161	6.380319	L
C-CT--0.182500	0	-0.815728	-5.390965	8.021024	L
H-HC-0.060300	0	-1.303788	-5.362598	8.995663	L
H-HC-0.060300	0	-0.909527	-6.396680	7.610482	L
H-HC-0.060300	0	-1.311763	-4.693852	7.345728	L
N-N--0.415700	0	1.540559	-3.962062	6.106781	L
H-H-0.271900	0	1.017717	-3.162716	6.446854	L
C-CT-0.033700	-1	2.465249	-3.726389	4.985977	L
H-H1-0.082300	0	2.334006	-4.496809	4.229906	L
C-C-0.597300	0	3.938909	-3.743562	5.451188	L
O-O--0.567900	0	4.843758	-4.014672	4.660171	L
C-CT--0.182500	0	2.107733	-2.357743	4.416297	L
H-HC-0.060300	0	2.565151	-1.567862	5.012806	L
H-HC-0.060300	0	1.028849	-2.218443	4.446100	L
H-HC-0.060300	0	2.453909	-2.300509	3.385915	L
N-N--0.4157	0	4.178153	-3.409139	6.726982	L
H-H-0.2719	0	3.380907	-3.056063	7.242504	L
C-CT--0.1490	-1	5.451299	-3.491335	7.429534	L
H-H1-0.0976	0	5.955233	-4.425412	7.173711	L
H-H1-0.0976	0	5.280425	-3.462062	8.507094	L
H-H1-0.0976	0	6.086997	-2.653306	7.139305	L
C-CT--0.3662	-1	14.090435	0.934771	-0.689114	L
H-HC-0.1123	0	13.439428	1.441558	0.020413	L
H-HC-0.1123	0	15.095371	1.350153	-0.630039	L
H-HC-0.1123	0	13.704151	1.051899	-1.699454	L
C-C-0.5972	0	14.149005	-0.545463	-0.350097	L
O-O--0.5679	0	15.231033	-1.105765	-0.241006	L
N-N--0.4157	0	12.989035	-1.184510	-0.201974	L
H-H-0.2719	0	12.145656	-0.635469	-0.320651	L

C-CT--0.0014 -1 12.814937 -2.638322 -0.000758 L
H-H1-0.0876 0 13.696954 -3.154737 -0.382152 L
C-C-0.5973 0 11.590099 -3.132831 -0.822830 L
O-O--0.5679 0 10.830054 -2.332290 -1.376876 L
C-CT--0.0152 0 12.681445 -2.955666 1.499443 L
H-HC-0.0295 0 13.625592 -2.705434 1.985796 L
H-HC-0.0295 0 12.537380 -4.029327 1.625041 L
C-CA--0.0011 -1 11.559295 -2.223225 2.215500 L
C-CA--0.1906 0 11.817994 -0.987241 2.838319 L
H-HA-0.1699 0 12.818321 -0.576503 2.818894 L
C-CA--0.1906 0 10.255677 -2.758309 2.241591 L
H-HA-0.1699 0 10.047176 -3.711335 1.775822 L
C-CA--0.2341 0 10.780468 -0.289289 3.484308 L
H-HA-0.1656 0 10.982944 0.646552 3.973427 L
C-CA--0.2341 0 9.213609 -2.058509 2.881547 L
H-HA-0.1656 0 8.217643 -2.474956 2.900845 L
C-C-0.3226 0 9.472986 -0.820095 3.508443 L
O-OH--0.557 0 8.474836 -0.138995 4.134641 L
H-HO-0.3992 0 7.663502 -0.687194 4.162647 L
N-N--0.415700 0 11.380787 -4.451193 -0.940196 L
H-H-0.271900 0 12.001846 -5.070337 -0.443064 L
C-CT--0.025200 -1 10.259885 -5.048629 -1.695463 L
H-H1-0.069800 0 10.476952 -6.099921 -1.883509 L
H-H1-0.069800 0 10.155385 -4.549808 -2.658254 L
C-C-0.597300 0 8.933452 -4.976144 -0.926394 L
O-O--0.567900 0 8.867881 -5.378829 0.233721 L
N-N--0.415700 0 7.862539 -4.488340 -1.559368 L
H-H-0.271900 0 7.944933 -4.353551 -2.559868 L
C-CT-0.014300 -1 6.525703 -4.464813 -0.966738 L
H-H1-0.104800 0 6.622794 -4.369911 0.117723 L
C-C-0.597300 0 5.786581 -5.774530 -1.245967 L
O-O--0.567900 0 5.066086 -5.903146 -2.241068 L
C-CT--0.204100 0 5.712446 -3.239893 -1.440228 L
H-HC-0.079700 0 5.579591 -3.288044 -2.520731 L
H-HC-0.079700 0 6.286227 -2.354671 -1.202239 L
C-C-0.713000 -1 4.327882 -3.106872 -0.783800 L
O-O--0.593100 0 3.932341 -3.855676 0.098846 L
N-N--0.919100 0 3.513200 -2.188147 -1.255337 L
H-H-0.419600 0 2.619465 -2.111217 -0.804970 L
H-H-0.419600 0 3.738680 -1.574087 -2.027387 L
N-N--0.347900 0 5.937854 -6.720555 -0.316602 L
H-H-0.274700 0 6.609897 -6.496980 0.407561 L
C-CT--0.263700 -1 5.385155 -8.079579 -0.378898 L
H-H1-0.156000 0 5.765304 -8.620573 0.487850 L
C-C-0.734100 0 5.890825 -8.873118 -1.594321 L
O-O--0.589400 0 6.894671 -8.527096 -2.210335 L
C-CT--0.000700 0 3.843617 -8.050743 -0.272548 L
H-HC-0.032700 0 3.467356 -9.073379 -0.227079 L
H-HC-0.032700 0 3.432783 -7.586387 -1.170240 L
C-CT-0.039000 0 3.300484 -7.322146 0.961511 L
H-HC-0.028500 0 3.659832 -6.295129 0.987562 L
H-HC-0.028500 0 3.618009 -7.838429 1.868552 L
C-CT-0.048600 0 1.773313 -7.327681 0.864791 L
H-H1-0.068700 0 1.420390 -8.360546 0.850389 L
H-H1-0.068700 0 1.467000 -6.854631 -0.070263 L

N-N2--0.529500 -1 1.140659 -6.633697 1.989588 L
H-H-0.345600 0 0.738507 -7.182051 2.744568 L
C-CA-0.807600 0 0.890052 -5.360009 2.101569 L
N-N2--0.862700 0 0.049651 -4.982568 3.007110 L
H-H-0.447800 0 -0.382835 -5.633888 3.646449 L
H-H-0.447800 0 -0.175873 -3.985892 3.048864 L
N-N2--0.862700 0 1.441223 -4.457766 1.343416 L
H-H-0.447800 0 2.334791 -4.606921 0.889294 L
H-H-0.447800 0 1.144190 -3.513768 1.604760 L
N-N--0.4157 0 5.211463 -9.982653 -1.892053 L
H-H-0.2719 0 4.419726 -10.192560 -1.308677 L
C-CT--0.1490 -1 5.514511 -10.874732 -2.996238 L
H-H1-0.0976 0 6.528060 -11.266714 -2.886180 L
H-H1-0.0976 0 4.808956 -11.706736 -3.017211 L
H-H1-0.0976 0 5.455025 -10.327777 -3.939355 L
C-CT--0.3662 -1 10.162029 -5.930483 -7.769478 L
H-HC-0.1123 0 9.878597 -6.637852 -8.546319 L
H-HC-0.1123 0 11.061139 -5.398081 -8.078382 L
H-HC-0.1123 0 10.356779 -6.451359 -6.833537 L
C-C-0.5972 0 9.044963 -4.927328 -7.564073 L
O-O--0.5679 0 9.267825 -3.731154 -7.715142 L
N-N--0.347900 0 7.849670 -5.381741 -7.155997 L
H-H-0.274700 0 7.771865 -6.376918 -7.009875 L
C-CT--0.263700 -1 6.876658 -4.480950 -6.502704 L
H-H1-0.156000 0 6.692232 -3.621610 -7.148357 L
C-C-0.734100 0 7.533035 -3.979668 -5.213232 L
O-O--0.589400 0 7.914313 -4.784294 -4.367958 L
C-CT--0.000700 0 5.544130 -5.174371 -6.172072 L
H-HC-0.032700 0 4.962558 -4.477972 -5.568060 L
H-HC-0.032700 0 5.723332 -6.061412 -5.560588 L
C-CT-0.039000 0 4.729326 -5.569896 -7.415025 L
H-HC-0.028500 0 4.963790 -6.604883 -7.667134 L
H-HC-0.028500 0 5.009744 -4.944367 -8.264085 L
C-CT-0.048600 0 3.214052 -5.436917 -7.181905 L
H-H1-0.068700 0 2.952974 -5.857373 -6.208503 L
H-H1-0.068700 0 2.698659 -6.024897 -7.943702 L
N-N2--0.529500 0 2.768625 -4.030883 -7.300714 L
H-H-0.345600 0 2.725918 -3.648323 -8.228640 L
C-CA-0.807600 0 2.457504 -3.183276 -6.333819 L
N-N2--0.862700 0 2.409480 -3.494794 -5.077374 L
H-H-0.447800 0 2.578649 -4.432705 -4.727947 L
H-H-0.447800 0 2.191550 -2.738972 -4.421958 L
N-N2--0.862700 0 2.187435 -1.941712 -6.597973 L
H-H-0.447800 0 2.214160 -1.530105 -7.509371 L
H-H-0.447800 0 2.042202 -1.377569 -5.757421 L
N-N--0.415700 0 7.719547 -2.671250 -5.074979 L
H-H-0.271900 0 7.390078 -2.069868 -5.812170 L
C-CT--0.025200 -1 8.621997 -2.097328 -4.074247 L
H-H1-0.069800 0 9.504122 -1.716488 -4.589717 L
H-H1-0.069800 0 8.964339 -2.856324 -3.375650 L
C-C-0.597300 0 8.030948 -0.954426 -3.269315 L
O-O--0.567900 0 6.980121 -0.406901 -3.605971 L
N-N--0.415700 0 8.718052 -0.572228 -2.196157 L
H-H-0.271900 0 9.569802 -1.070121 -1.940514 L
C-CT--0.038900 -1 8.309062 0.557317 -1.366886 L

H-H1-0.100700	0	7.677921	1.188326	-1.989045	L
C-C-0.597300	0	9.443906	1.485689	-0.960449	L
O-O--0.567900	0	10.553968	1.070770	-0.631204	L
C-CT-0.365400	0	7.415482	0.107641	-0.194981	L
H-H1-0.004300	0	7.727203	-0.882792	0.139860	L
O-OH--0.676100	0	6.095108	0.052688	-0.700296	L
H-HO-0.410200	0	6.161293	-0.140278	-1.646757	L
C-CT--0.243800	0	7.351662	1.034653	1.018253	L
H-HC-0.064200	0	6.996656	2.021631	0.723446	L
H-HC-0.064200	0	8.334320	1.114121	1.481789	L
H-HC-0.064200	0	6.661701	0.615611	1.751127	L
N-N--0.415700	0	9.117887	2.777649	-1.018996	L
H-H-0.271900	0	8.149278	2.982480	-1.244007	L
C-CT--0.025200	-1	9.958568	3.905994	-0.666609	L
H-H1-0.069800	0	9.764061	4.726050	-1.357691	L
H-H1-0.069800	0	11.009154	3.623181	-0.752130	L
C-C-0.597300	0	9.660784	4.387871	0.740585	L
O-O--0.567900	0	8.512684	4.601534	1.142866	L
N-N--0.347900	0	10.734636	4.592143	1.487793	L
H-H-0.274700	0	11.640893	4.434481	1.061945	L
C-CT--0.263700	-1	10.742921	5.185115	2.815843	L
H-H1-0.156000	0	9.836626	5.768193	2.968863	L
C-C-0.734100	0	11.912271	6.141727	2.918444	L
O-O--0.589400	0	12.860985	6.061281	2.143555	L
C-CT--0.000700	0	10.895118	4.080769	3.883909	L
H-HC-0.032700	0	11.174021	4.526986	4.840125	L
H-HC-0.032700	0	11.706305	3.411516	3.589311	L
C-CT-0.039000	0	9.629927	3.249690	4.104260	L
H-HC-0.028500	0	9.866912	2.423123	4.773622	L
H-HC-0.028500	0	9.284000	2.836828	3.156198	L
C-CT-0.048600	0	8.525959	4.083217	4.757768	L
H-H1-0.068700	0	8.333245	4.985759	4.178621	L
H-H1-0.068700	0	8.846886	4.380517	5.757835	L
N-N2--0.529500	-1	7.296095	3.298107	4.858475	L
H-H-0.345600	0	7.202860	2.665844	5.634796	L
C-CA-0.807600	0	6.321827	3.239544	3.970744	L
N-N2--0.862700	0	5.302717	2.462385	4.161977	L
H-H-0.447800	0	5.279475	1.766015	4.880924	L
H-H-0.447800	0	4.711170	2.332896	3.336880	L
N-N2--0.862700	0	6.328160	3.928554	2.870790	L
H-H-0.447800	0	7.169575	4.338605	2.492207	L
H-H-0.447800	0	5.479524	3.893401	2.303116	L
N-N--0.4157	0	11.828848	7.022745	3.906146	L
H-H-0.2719	0	11.011791	7.007564	4.492559	L
C-CT--0.1490	-1	12.893723	7.946438	4.203232	L
H-H1-0.0976	0	12.626804	8.574659	5.054610	L
H-H1-0.0976	0	13.809185	7.398060	4.437595	L
H-H1-0.0976	0	13.086630	8.583729	3.336970	L
C-CT--0.3662	-1	-2.177713	13.241134	-1.533714	L
H-HC-0.1123	0	-2.600938	13.751510	-0.671057	L
H-HC-0.1123	0	-1.091015	13.228816	-1.478170	L
H-HC-0.1123	0	-2.482929	13.759612	-2.442391	L
C-C-0.5972	0	-2.699408	11.817024	-1.591101	L
O-O--0.5679	0	-3.350177	11.442847	-2.553996	L
N-N--0.415700	0	-2.421202	11.031349	-0.547222	L

H-H-0.271900 0 -1.861822 11.449831 0.176858 L
C-CT--0.023700 -1 -2.840844 9.623284 -0.380509 L
H-H1-0.088000 0 -2.372746 9.034727 -1.171158 L
C-C-0.597300 0 -4.360154 9.437663 -0.540058 L
O-O--0.567900 0 -4.821612 8.649715 -1.365604 L
C-CT-0.034200 0 -2.299974 9.139144 0.970013 L
H-HC-0.024100 0 -2.680375 9.782565 1.764415 L
H-HC-0.024100 0 -1.212619 9.221432 0.964341 L
C-CT-0.001800 0 -2.677332 7.698394 1.297488 L
H-H1-0.044000 0 -3.750210 7.663046 1.490041 L
H-H1-0.044000 0 -2.166079 7.435478 2.218849 L
S-S--0.273700 0 -2.295463 6.438135 0.055687 L
C-CT--0.053600 0 -0.495403 6.466571 0.020945 L
H-H1-0.068400 0 -0.140214 5.777696 -0.745114 L
H-H1-0.068400 0 -0.125853 6.148017 0.993372 L
H-H1-0.068400 0 -0.142773 7.470940 -0.210743 L
N-N--0.4157 0 -5.144624 10.175317 0.250213 L
H-H-0.2719 0 -4.688587 10.793569 0.901529 L
C-CT--0.1490 -1 -6.595537 10.189679 0.152318 L
H-H1-0.0976 0 -7.017076 10.855100 0.907020 L
H-H1-0.0976 0 -6.892060 10.538192 -0.839335 L
H-H1-0.0976 0 -6.985400 9.181454 0.303549 L
C-CT--0.3662 -1 -4.451672 8.975734 -5.131859 L
H-HC-0.1123 0 -4.023527 9.335697 -4.197134 L
H-HC-0.1123 0 -3.688739 8.982536 -5.908242 L
H-HC-0.1123 0 -5.281560 9.611224 -5.432388 L
C-C-0.5972 0 -4.954230 7.550512 -4.948837 L
O-O--0.5679 0 -4.649378 6.688240 -5.760476 L
N-N--0.415700 0 -5.733550 7.306377 -3.891623 L
H-H-0.271900 0 -5.768892 8.056593 -3.213374 L
C-CT--0.087500 -1 -6.398308 6.015785 -3.566644 L
H-H1-0.096900 0 -6.108081 5.267714 -4.303605 L
C-C-0.597300 0 -7.911058 6.181242 -3.676261 L
O-O--0.567900 0 -8.546272 5.513415 -4.482753 L
C-CT-0.298500 0 -5.958359 5.525878 -2.178774 L
H-HC--0.029700 0 -6.165968 6.296418 -1.435776 L
C-CT--0.319200 0 -6.647956 4.235338 -1.732941 L
H-HC-0.079100 0 -6.441050 3.450944 -2.454707 L
H-HC-0.079100 0 -7.723438 4.385874 -1.649601 L
H-HC-0.079100 0 -6.271837 3.926584 -0.757793 L
C-CT--0.319200 0 -4.460511 5.241822 -2.198506 L
H-HC-0.079100 0 -4.226885 4.484301 -2.942521 L
H-HC-0.079100 0 -4.172703 4.893901 -1.216342 L
H-HC-0.079100 0 -3.900332 6.149245 -2.422139 L
N-N--0.4157 0 -8.496626 7.081325 -2.878334 L
H-H-0.2719 0 -7.890175 7.602921 -2.265508 L
C-CT--0.1490 -1 -9.926267 7.371361 -2.909637 L
H-H1-0.0976 0 -10.174174 8.140948 -2.178101 L
H-H1-0.0976 0 -10.206636 7.717143 -3.907050 L
H-H1-0.0976 0 -10.490270 6.463036 -2.688737 L
C-CT-0.1200 -1 -8.866543 1.942740 3.242204 L
H-HC-0.0800 0 -8.118193 2.727443 3.341729 L
H-HC-0.0800 0 -9.670090 2.300319 2.598553 L
H-HC-0.0800 0 -9.276887 1.705157 4.224220 L
C-CT-0.2000 0 -8.261614 0.718779 2.639137 L

H-H1-0.0800	0	-9.041252	-0.034566	2.496073	L
O-OS--0.5600	0	-7.659329	1.059103	1.409512	L
C-CT-0.2000	0	-7.145497	0.169735	3.518441	L
H-H1-0.0800	0	-6.449510	0.957735	3.808596	L
O-OH--0.6800	0	-7.627580	-0.517675	4.667544	L
H-HO-0.4000	0	-8.320527	-1.130044	4.374011	L
C-CT-0.2000	0	-6.508450	-0.710270	2.455668	L
H-H1-0.0800	0	-5.487570	-0.986998	2.724733	L
O-OH--0.6800	0	-7.343313	-1.851621	2.302146	L
H-HO-0.4000	0	-6.821771	-2.540912	1.850183	L
C-CT-0.5691	0	-6.556537	0.171500	1.198783	H
H-H2-0.8000	0	-6.707546	-0.485945	0.321059	H
N-N*--0.5691	0	-5.351540	0.982969	1.022149	H
C-CM--0.0500	0	-4.711547	0.797512	-0.165740	H
H-H4-0.1500	0	-5.046972	-0.069232	-0.688702	H
C-CM--0.1238	0	-3.787212	1.605626	-0.686637	H
C-C-0.6156	0	-3.491279	1.571381	-2.135364	H
O-O--0.5700	0	-2.687934	2.319088	-2.635372	H
N-N--0.8000	0	-4.245100	0.749689	-2.923395	H
H-H-0.3700	0	-3.935836	0.549554	-3.852782	H
H-H-0.3700	0	-4.909153	0.108808	-2.544271	H
C-CT-0.1164	0	-3.176379	2.661921	0.203406	H
H-HC-0.0800	0	-2.819752	3.497124	-0.379640	H
H-HC-0.0800	0	-2.298499	2.275318	0.718457	H
C-CM--0.2882	0	-4.209603	3.069379	1.225854	H
H-HA-0.1500	0	-4.139629	4.048495	1.656489	H
C-CM--0.0500	0	-5.172594	2.250747	1.607935	H
H-H4-0.1500	0	-5.881057	2.532260	2.356029	H
N-N3--0.8530	0	3.294512	1.072410	-0.530037	H
H-H-0.4500	0	3.450271	0.839800	-1.497625	H
H-H-0.4500	0	2.140944	0.492138	0.782317	H
H-H-0.4500	0	4.143090	0.849304	-0.029193	H
C-CT-0.3170	0	3.351903	2.526508	-0.412776	H
H-HP-0.0800	0	4.110533	2.934727	-1.074367	H
C-CT--0.1600	0	2.034065	3.242978	-0.706101	H
H-HC-0.0800	0	1.796675	3.110884	-1.755571	H
H-HC-0.0800	0	2.215341	4.301746	-0.565158	H
C-CT--0.1600	0	0.851322	2.809842	0.165810	H
H-HC-0.0800	0	0.078118	3.562716	0.110789	H
H-HC-0.0800	0	1.188776	2.794124	1.197124	H
C-CT--0.0990	0	0.254474	1.443194	-0.223224	H
H-HC-0.0800	0	0.852953	0.995383	-1.006318	H
H-HC-0.0800	0	-0.737111	1.581615	-0.628550	H
C-C-0.4490	0	0.222668	0.458550	0.936157	H
H-H-0.0600	0	-0.319546	0.860580	1.781106	H
O-O--0.5700	0	1.468629	0.044066	1.330746	H
N-N3--0.9900	0	-0.550241	-0.790699	0.507393	H
H-H-0.3600	0	0.126518	-1.535471	0.597693	H
H-H-0.3600	0	-0.800230	-0.660543	-0.471191	H
C-C-0.9060	0	3.873381	2.787678	1.004987	L
O-O2--0.9000	0	4.163644	1.839189	1.770106	L
O-O2--0.9000	0	4.027836	3.954223	1.415661	L
C-CT--0.205900	0	-1.728657	-1.260086	1.328579	H
H-H1-0.139900	0	-2.285375	-0.386004	1.636301	H
C-CT-0.007100	0	-2.580725	-2.157283	0.419723	H

H-HC--0.007800 0 -1.938445 -2.844126 -0.120494 H
H-HC--0.007800 0 -3.100922 -1.546801 -0.310948 H
C-CT-0.067500 0 -3.601944 -2.999802 1.184249 L
H-HC--0.054800 0 -3.909534 -2.454346 2.077425 L
H-HC--0.054800 0 -3.135361 -3.936850 1.496118 L
C-C-0.818300 0 -4.873327 -3.277577 0.380227 L
O-O2--0.822000 0 -5.718826 -2.351522 0.300176 L
O-O2--0.822000 0 -5.120949 -4.395055 -0.123299 L
C-C-0.742000 0 -1.105180 -2.041996 2.521911 H
O-O2--0.793000 0 -1.780281 -2.259993 3.510804 H
O-O2--0.793000 0 0.032483 -2.429739 2.252044 H
O-OW--0.834000 0 2.731414 -5.789577 -3.514345 L
H-HW-0.417000 0 3.595658 -5.915311 -3.071480 L
H-HW-0.417000 0 2.143586 -5.547493 -2.782640 L
O-OW--0.834000 0 0.234106 -7.443705 4.606005 L
H-HW-0.417000 0 0.897828 -7.069952 5.219671 L
H-HW-0.417000 0 -0.288619 -8.014349 5.175691 L
O-OW--0.834000 0 4.053032 -0.301070 3.106270 L
H-HW-0.417000 0 3.157885 -0.585900 2.887426 L
H-HW-0.417000 0 4.153314 0.514647 2.554619 L
O-OW--0.834000 0 6.157248 -1.543169 4.223514 L
H-HW-0.417000 0 5.837257 -2.447801 4.381284 L
H-HW-0.417000 0 5.382033 -1.116565 3.800366 L
O-OW--0.834000 0 -6.103480 -6.891329 0.048871 L
H-HW-0.417000 0 -5.777222 -5.969828 0.025504 L
H-HW-0.417000 0 -6.627433 -6.988796 -0.753979 L
O-OW--0.834000 0 -9.111148 -0.833705 -0.205465 L
H-HW-0.417000 0 -9.974966 -0.825053 0.215365 L
H-HW-0.417000 0 -8.893175 -1.792582 -0.229172 L

TS2
C-CT--0.3662 -1 -12.873868 1.011123 1.930712 L
H-HC-0.1123 0 -12.286828 1.880210 1.637075 L
H-HC-0.1123 0 -13.931416 1.236318 1.803301 L
H-HC-0.1123 0 -12.594113 0.157718 1.316871 L
C-C-0.5972 0 -12.602391 0.697555 3.389283 L
O-O--0.5679 0 -11.848615 1.421290 4.029400 L
N-N--0.415700 0 -13.192480 -0.388104 3.899847 L
H-H-0.271900 0 -13.789017 -0.913440 3.281746 L
C-CT--0.025200 -1 -12.903134 -0.948893 5.232870 L
H-H1-0.069800 0 -13.640758 -1.715886 5.468246 L
H-H1-0.069800 0 -12.968197 -0.155709 5.978370 L
C-C-0.597300 0 -11.480190 -1.581687 5.309824 L
O-O--0.567900 0 -10.645370 -1.409567 4.415278 L
N-N--0.415700 0 -11.185843 -2.347988 6.371557 L
H-H-0.271900 0 -11.905921 -2.484493 7.063232 L
C-CT--0.025200 -1 -9.988504 -3.215393 6.448977 L
H-H1-0.069800 0 -9.903293 -3.634301 7.451323 L
H-H1-0.069800 0 -9.094492 -2.629788 6.230979 L
C-C-0.597300 0 -10.115554 -4.362261 5.454991 L
O-O--0.567900 0 -11.155452 -5.013222 5.384169 L
N-N--0.4157 0 -9.057883 -4.602397 4.689511 L
H-H-0.2719 0 -8.225943 -4.056759 4.839328 L
C-CT--0.0014 -1 -8.999238 -5.560013 3.586714 L
H-H1-0.0876 0 -9.910765 -5.487237 2.992554 L

C-C-0.5973	0	-8.867565	-7.028835	4.049097	L
O-O--0.5679	0	-8.072379	-7.804383	3.525454	L
C-CT--0.0152	0	-7.811522	-5.150760	2.698576	L
H-HC-0.0295	0	-8.054508	-4.183790	2.277132	L
H-HC-0.0295	0	-7.766802	-5.835034	1.851787	L
C-CA--0.0011	0	-6.422361	-5.064322	3.342930	L
C-CA--0.1906	0	-5.483964	-6.093058	3.125643	L
H-HA-0.1699	0	-5.780064	-6.978981	2.576752	L
C-CA--0.1906	0	-6.022217	-3.924623	4.076034	L
H-HA-0.1699	0	-6.714954	-3.127195	4.283387	L
C-CA--0.2341	0	-4.156193	-5.967642	3.580612	L
H-HA-0.1656	0	-3.436046	-6.744990	3.381910	L
C-CA--0.2341	0	-4.689406	-3.779589	4.506938	L
H-HA-0.1656	0	-4.362350	-2.885137	5.012404	L
C-C-0.3326	0	-3.747833	-4.795872	4.247755	L
O-OH--0.5579	0	-2.448389	-4.623139	4.602470	L
H-HO-0.3992	0	-2.164238	-3.730159	4.296332	L
N-N--0.4157	0	-9.658636	-7.430544	5.044678	L
H-H-0.2719	0	-10.344896	-6.761578	5.374221	L
C-CT--0.1490	-1	-9.569196	-8.748449	5.649922	L
H-H1-0.0976	0	-8.565603	-8.903097	6.050800	L
H-H1-0.0976	0	-10.300109	-8.844371	6.453567	L
H-H1-0.0976	0	-9.762354	-9.512603	4.894145	L
C-CT--0.3662	-1	-12.000924	-1.483129	-3.478559	L
H-HC-0.1123	0	-12.522038	-0.572645	-3.770253	L
H-HC-0.1123	0	-12.005613	-1.597867	-2.396953	L
H-HC-0.1123	0	-12.495243	-2.340015	-3.935323	L
C-C-0.5972	0	-10.570327	-1.428386	-3.978450	L
O-O--0.5679	0	-10.109583	-2.289550	-4.715170	L
N-N--0.415700	0	-9.846896	-0.415852	-3.523042	L
H-H-0.271900	0	-10.337203	0.254517	-2.947500	L
C-CT--0.038900	-1	-8.404828	-0.129058	-3.686241	L
H-H1-0.100700	0	-8.111237	-0.156776	-4.733559	L
C-C-0.597300	0	-7.556258	-1.107622	-2.874922	L
O-O--0.567900	0	-6.754797	-0.710794	-2.025333	L
C-CT-0.365400	0	-8.223263	1.297788	-3.116715	L
H-H1-0.004300	0	-7.165353	1.550374	-3.111921	L
O-OH--0.676100	0	-8.751762	1.400919	-1.803302	L
H-HO-0.410200	0	-8.653004	0.554319	-1.340504	L
C-CT--0.243800	0	-9.003581	2.370803	-3.873378	L
H-HC-0.064200	0	-10.061036	2.124831	-3.950819	L
H-HC-0.064200	0	-8.598916	2.506388	-4.866602	L
H-HC-0.064200	0	-8.921744	3.314980	-3.342469	L
N-N--0.415700	0	-7.779252	-2.399889	-3.072824	L
H-H-0.271900	0	-8.450475	-2.664329	-3.790257	L
C-CT--0.024900	-1	-7.193248	-3.475142	-2.281392	L
H-H1-0.084300	0	-6.313902	-3.103106	-1.778467	L
C-C-0.597300	0	-6.698011	-4.612122	-3.178681	L
O-O--0.567900	0	-7.210025	-4.802028	-4.279554	L
C-CT-0.211700	0	-8.196600	-4.067798	-1.293880	L
H-H1-0.035200	0	-7.908490	-5.085545	-1.026600	L
H-H1-0.035200	0	-9.200754	-4.078653	-1.720871	L
O-OH--0.654600	0	-8.147624	-3.276098	-0.141431	L
H-HO-0.427500	0	-7.188684	-3.211273	0.063523	L
N-N--0.4157	0	-5.712686	-5.387273	-2.714429	L

H-H-0.2719	0	-5.316512	-5.158043	-1.803939	L
C-CT--0.0014	0	-5.312287	-6.629760	-3.385748	L
H-H1-0.0876	0	-4.996586	-6.387367	-4.400533	L
C-C-0.5973	0	-6.458623	-7.640441	-3.455080	L
O-O--0.5679	0	-7.337135	-7.659020	-2.591580	L
C-CT--0.0152	0	-4.164394	-7.341901	-2.660312	L
H-HC-0.0295	0	-3.882811	-8.220595	-3.242157	L
H-HC-0.0295	0	-4.516921	-7.719730	-1.702529	L
C-CA--0.0011	-1	-2.914372	-6.542303	-2.429761	L
C-CA--0.1906	0	-1.964452	-6.403704	-3.462780	L
H-HA-0.1699	0	-2.170984	-6.810471	-4.442263	L
C-CA--0.1906	0	-2.624768	-6.037533	-1.147027	L
H-HA-0.1699	0	-3.340965	-6.159833	-0.342780	L
C-CA--0.2341	0	-0.732136	-5.766973	-3.212529	L
H-HA-0.1656	0	0.004932	-5.667806	-3.994024	L
C-CA--0.2341	0	-1.397816	-5.396092	-0.893137	L
H-HA-0.1656	0	-1.194727	-5.015380	0.096714	L
C-C-0.3226	0	-0.444179	-5.267896	-1.924331	L
O-OH--0.5579	0	0.759286	-4.687877	-1.670936	L
H-HO-0.3992	0	0.712339	-4.238548	-0.819057	L
N-N--0.4157	0	-6.353562	-8.552703	-4.425204	L
H-H-0.2719	0	-5.607303	-8.433012	-5.089960	L
C-CT--0.1490	-1	-7.133638	-9.774457	-4.477436	L
H-H1-0.0976	0	-8.195046	-9.527196	-4.550415	L
H-H1-0.0976	0	-6.845036	-10.374187	-5.341522	L
H-H1-0.0976	0	-6.976198	-10.353518	-3.565004	L
C-CT--0.3662	-1	-6.327370	-3.524318	-6.966523	L
H-HC-0.1123	0	-5.819048	-4.272436	-7.571341	L
H-HC-0.1123	0	-7.369359	-3.814017	-6.835816	L
H-HC-0.1123	0	-5.843673	-3.445501	-5.998151	L
C-C-0.5972	0	-6.246555	-2.177664	-7.668813	L
O-O--0.5679	0	-5.360224	-2.005439	-8.493745	L
N-N--0.415700	0	-7.143004	-1.232764	-7.346834	L
H-H-0.271900	0	-7.858423	-1.506822	-6.690772	L
C-CT--0.025200	-1	-7.283284	0.082961	-8.021465	L
H-H1-0.069800	0	-8.233213	0.096163	-8.556172	L
H-H1-0.069800	0	-6.497444	0.211016	-8.765385	L
C-C-0.597300	0	-7.257111	1.295747	-7.097548	L
O-O--0.567900	0	-8.135414	2.140621	-7.207245	L
N-N--0.415700	0	-6.257382	1.380767	-6.213897	L
H-H-0.271900	0	-5.572208	0.633175	-6.230967	L
C-CT--0.025200	-1	-5.825246	2.619424	-5.555446	L
H-H1-0.069800	0	-5.648852	2.437407	-4.498354	L
H-H1-0.069800	0	-6.586976	3.393420	-5.660850	L
C-C-0.597300	0	-4.538193	3.134647	-6.209625	L
O-O--0.567900	0	-4.473780	3.260914	-7.429524	L
N-N--0.415700	0	-3.496180	3.370256	-5.409498	L
H-H-0.271900	0	-3.615664	3.226266	-4.417067	L
C-CT--0.051800	-1	-2.158756	3.757774	-5.874803	L
H-H1-0.092200	0	-2.243684	4.211044	-6.863932	L
C-C-0.597300	0	-1.239596	2.524693	-5.998640	L
O-O--0.567900	0	-0.553832	2.319324	-6.997002	L
C-CT--0.110200	0	-1.612704	4.811163	-4.890510	L
H-HC-0.045700	0	-1.582786	4.376260	-3.891354	L
H-HC-0.045700	0	-2.309275	5.650386	-4.863955	L

C-CT-0.353100 0 -0.211045 5.349832 -5.221313 L
H-HC--0.036100 0 0.503223 4.526582 -5.237411 L
C-CT--0.412100 0 -0.162752 6.077558 -6.564637 L
H-HC-0.100000 0 -0.914406 6.867583 -6.588629 L
H-HC-0.100000 0 -0.359277 5.374098 -7.373689 L
H-HC-0.100000 0 0.825011 6.510200 -6.719017 L
C-CT--0.412100 0 0.214266 6.332376 -4.127907 L
H-HC-0.100000 0 -0.493110 7.161176 -4.078973 L
H-HC-0.100000 0 1.208099 6.721824 -4.346840 L
H-HC-0.100000 0 0.237941 5.820460 -3.167111 L
N-N--0.516300 0 -1.196695 1.713621 -4.944520 L
H-H-0.293600 0 -1.772105 1.994280 -4.157212 L
C-CT-0.038100 -1 -0.299877 0.581613 -4.724664 L
H-H1-0.088000 0 -0.060458 0.087951 -5.664200 L
C-C-0.536600 0 -0.964507 -0.415768 -3.735408 L
O-O--0.581900 0 -0.790065 -0.293474 -2.521694 L
C-CT--0.030300 0 1.004500 1.174285 -4.116977 L
H-HC--0.012200 0 0.739387 1.791615 -3.259440 L
H-HC--0.012200 0 1.462736 1.831014 -4.858580 L
C-C-0.799400 -1 2.064919 0.154751 -3.672190 L
O-O2--0.801400 0 1.922084 -1.036141 -4.033807 L
O-O2--0.801400 0 3.126351 0.549103 -3.145651 L
N-N--0.254800 0 -1.754404 -1.396780 -4.208417 L
C-CT--0.026600 -1 -2.044863 -1.694141 -5.608016 L
H-H1-0.064100 0 -1.119787 -1.747835 -6.180785 L
C-C-0.589600 0 -2.989988 -0.672386 -6.266455 L
O-O--0.574800 0 -3.922951 -0.180156 -5.628602 L
C-CT--0.007000 0 -2.685899 -3.085837 -5.580428 L
H-HC-0.025300 0 -1.903378 -3.846297 -5.586663 L
H-HC-0.025300 0 -3.371773 -3.243922 -6.413408 L
C-CT-0.018900 0 -3.405150 -3.112638 -4.233113 L
H-HC-0.021300 0 -3.553875 -4.127912 -3.874490 L
H-HC-0.021300 0 -4.357176 -2.584941 -4.303289 L
C-CT-0.019200 0 -2.452588 -2.331123 -3.335341 L
H-H1-0.039100 0 -3.011776 -1.805617 -2.561525 L
H-H1-0.039100 0 -1.730052 -3.006478 -2.877972 L
N-N--0.4157 0 -2.749020 -0.387861 -7.551188 L
H-H-0.2719 0 -1.965999 -0.843367 -7.987215 L
C-CT--0.1490 -1 -3.537501 0.497321 -8.408211 L
H-H1-0.0976 0 -4.499498 0.714277 -7.957113 L
H-H1-0.0976 0 -3.712312 0.023576 -9.375184 L
H-H1-0.0976 0 -3.005488 1.439242 -8.559734 L
C-CT--0.3662 -1 8.748217 1.904492 -5.914026 L
H-HC-0.1123 0 9.251923 2.164139 -6.844728 L
H-HC-0.1123 0 9.491686 1.665137 -5.156631 L
H-HC-0.1123 0 8.089772 1.054909 -6.083502 L
C-C-0.5972 0 7.934619 3.093616 -5.453593 L
O-O--0.5679 0 8.092680 4.181596 -5.987658 L
N-N--0.415700 0 7.065248 2.895334 -4.462425 L
H-H-0.271900 0 6.986811 1.959859 -4.087882 L
C-CT-0.021300 -1 6.077374 3.893366 -4.027198 L
H-H1-0.112400 0 6.274334 4.845996 -4.523321 L
C-C-0.597300 0 6.111296 4.133975 -2.515506 L
O-O--0.567900 0 6.365907 3.216883 -1.734392 L
C-CT--0.123100 0 4.702031 3.387353 -4.491259 L

H-H1-0.111200	0	4.493945	2.419776	-4.030026	L
H-H1-0.111200	0	4.714748	3.259856	-5.575354	L
S-SH--0.311900	0	3.386862	4.557752	-4.050427	L
H-HS-0.193300	0	2.381562	3.906771	-4.653846	L
N-N--0.415700	0	5.833678	5.367787	-2.095640	L
H-H-0.271900	0	5.588573	6.060410	-2.790255	L
C-CT--0.025200	-1	5.817149	5.777497	-0.696564	L
H-H1-0.069800	0	6.782703	6.218841	-0.450183	L
H-H1-0.069800	0	5.677324	4.915493	-0.048700	L
C-C-0.597300	0	4.742305	6.820439	-0.413605	L
O-O--0.567900	0	4.851112	7.965447	-0.842473	L
N-N--0.415700	0	3.729440	6.442871	0.360028	L
H-H-0.271900	0	3.777719	5.499762	0.736733	L
C-CT--0.025200	-1	2.683462	7.306741	0.892048	L
H-H1-0.069800	0	1.863626	6.680625	1.228792	L
H-H1-0.069800	0	2.329895	7.995205	0.123968	L
C-C-0.597300	0	3.175393	8.103117	2.098833	L
O-O--0.567900	0	2.585800	8.014750	3.173654	L
N-N--0.4157	0	4.269850	8.849030	1.914673	L
H-H-0.2719	0	4.636589	8.853556	0.967891	L
C-CT--0.1490	-1	5.009727	9.536286	2.963937	L
H-H1-0.0976	0	5.331149	8.818344	3.720413	L
H-H1-0.0976	0	5.883436	10.034644	2.541326	L
H-H1-0.0976	0	4.367120	10.280470	3.438511	L
C-CT--0.3662	-1	-1.516639	9.430571	5.280941	L
H-HC-0.1123	0	-1.731396	10.395328	4.824188	L
H-HC-0.1123	0	-0.576896	9.042953	4.890998	L
H-HC-0.1123	0	-1.453538	9.549038	6.361145	L
C-C-0.5972	0	-2.648840	8.456391	4.943814	L
O-O--0.5679	0	-3.536586	8.810119	4.178137	L
N-N--0.4157	0	-2.617879	7.229917	5.481798	L
H-H-0.2719	0	-1.866562	7.015447	6.123051	L
C-CT--0.0275	-1	-3.609332	6.137182	5.302164	L
H-H1-0.1123	0	-4.607708	6.572058	5.246226	L
C-C-0.5973	0	-3.570671	5.159828	6.506589	L
O-O--0.5679	0	-2.649408	5.215809	7.319790	L
C-CT--0.0050	0	-3.323536	5.370659	4.003194	L
H-HC-0.0339	0	-3.508597	6.032746	3.164960	L
H-HC-0.0339	0	-4.057403	4.570115	3.917100	L
C-C*--0.1415	0	-1.954804	4.762966	3.858312	L
C-CW--0.1638	0	-0.796451	5.433889	3.646136	L
H-H4-0.2062	0	-0.705562	6.508487	3.555742	L
C-CB-0.1243	0	-1.584250	3.353161	3.935130	L
N-NA--0.3418	0	0.258314	4.542937	3.586159	L
H-H-0.3412	0	1.226218	4.815764	3.467234	L
C-CN-0.1380	0	-0.170253	3.247225	3.769442	L
C-CA--0.238	0	-2.301092	2.154166	4.141757	L
H-HA-0.1700	0	-3.372709	2.191888	4.250847	L
C-CA--0.2601	0	0.501992	2.018834	3.820597	L
H-HA-0.1572	0	1.572334	1.968376	3.681558	L
C-CA--0.1972	0	-1.637250	0.915078	4.190036	L
H-HA-0.1447	0	-2.193999	-0.003261	4.324204	L
C-CA--0.1134	0	-0.242827	0.847409	4.034238	L
H-HA-0.1417	0	0.248031	-0.116232	4.045537	L
N-N--0.4157	0	-4.557450	4.252815	6.623591	L

H-H-0.2719	0	-5.279854	4.285822	5.923151	L
C-CT--0.1490	-1	-4.650958	3.227376	7.677875	L
H-H1-0.0976	0	-3.833504	2.513212	7.566121	L
H-H1-0.0976	0	-4.568281	3.701319	8.658541	L
H-H1-0.0976	0	-5.604446	2.701150	7.613170	L
C-CT--0.3662	-1	-2.799505	-1.490315	7.277028	L
H-HC-0.1123	0	-3.027071	-2.545778	7.127430	L
H-HC-0.1123	0	-3.154223	-1.176732	8.256254	L
H-HC-0.1123	0	-3.278804	-0.910173	6.490806	L
C-C-0.5972	0	-1.307496	-1.301138	7.185714	L
O-O--0.5679	0	-0.592702	-2.257846	6.942507	L
N-N--0.415700	0	-0.812353	-0.093896	7.455310	L
H-H-0.271900	0	-1.472074	0.659640	7.567100	L
C-CT--0.087500	-1	0.629800	0.237195	7.419915	L
H-H1-0.096900	0	0.962731	0.139061	6.386655	L
C-C-0.597300	0	1.474539	-0.729819	8.260158	L
O-O--0.567900	0	2.461098	-1.270791	7.773235	L
C-CT-0.298500	0	0.906967	1.689072	7.879916	L
H-HC--0.029700	0	0.964301	1.711691	8.968802	L
C-CT--0.319200	0	2.251000	2.169823	7.319714	L
H-HC-0.079100	0	2.213071	2.194197	6.230284	L
H-HC-0.079100	0	3.051005	1.499659	7.635151	L
H-HC-0.079100	0	2.469742	3.171105	7.691371	L
C-CT--0.319200	0	-0.176251	2.696431	7.470821	L
H-HC-0.079100	0	-0.366100	2.629211	6.403683	L
H-HC-0.079100	0	0.144441	3.711590	7.707114	L
H-HC-0.079100	0	-1.101311	2.509627	8.014151	L
N-N--0.415700	0	1.064260	-0.988172	9.509636	L
H-H-0.271900	0	0.244535	-0.497054	9.827558	L
C-CT--0.025200	-1	1.671811	-1.992226	10.398821	L
H-H1-0.069800	0	1.125240	-2.026711	11.340622	L
H-H1-0.069800	0	2.701825	-1.703741	10.611644	L
C-C-0.597300	0	1.694821	-3.420523	9.803228	L
O-O--0.567900	0	2.532560	-4.222327	10.197957	L
N-N--0.415700	0	0.788748	-3.745182	8.868781	L
H-H-0.271900	0	0.174526	-3.015295	8.535804	L
C-CT-0.033700	-1	0.669664	-5.032303	8.170425	L
H-H1-0.082300	0	1.128219	-5.801231	8.795139	L
C-C-0.597300	0	1.401752	-5.088331	6.805725	L
O-O--0.567900	0	1.829784	-6.161106	6.378142	L
C-CT--0.182500	0	-0.816238	-5.388669	8.024760	L
H-HC-0.060300	0	-1.303059	-5.361350	9.000056	L
H-HC-0.060300	0	-0.909846	-6.394217	7.613788	L
H-HC-0.060300	0	-1.313483	-4.691331	7.350597	L
N-N--0.415700	0	1.541414	-3.957140	6.110176	L
H-H-0.271900	0	1.023387	-3.156057	6.453242	L
C-CT-0.033700	-1	2.464376	-3.724224	4.987488	L
H-H1-0.082300	0	2.339044	-4.503433	4.239233	L
C-C-0.597300	0	3.938758	-3.728385	5.451027	L
O-O--0.567900	0	4.845244	-3.983403	4.656546	L
C-CT--0.182500	0	2.093665	-2.366549	4.403244	L
H-HC-0.060300	0	2.514756	-1.565989	5.012061	L
H-HC-0.060300	0	1.011503	-2.254869	4.400533	L
H-HC-0.060300	0	2.469782	-2.304072	3.383608	L
N-N--0.4157	0	4.176550	-3.405938	6.730019	L

H-H-0.2719	0	3.377365	-3.064715	7.250176	L
C-CT--0.1490	-1	5.450566	-3.488071	7.430769	L
H-H1-0.0976	0	5.957193	-4.419085	7.169183	L
H-H1-0.0976	0	5.280772	-3.465277	8.508664	L
H-H1-0.0976	0	6.083325	-2.646457	7.144504	L
C-CT--0.3662	-1	14.088945	0.935427	-0.690106	L
H-HC-0.1123	0	13.436023	1.442450	0.017498	L
H-HC-0.1123	0	15.093511	1.351413	-0.629094	L
H-HC-0.1123	0	13.704910	1.051480	-1.701426	L
C-C-0.5972	0	14.147569	-0.544527	-0.349764	L
O-O--0.5679	0	15.229645	-1.104672	-0.240245	L
N-N--0.4157	0	12.987654	-1.183598	-0.201224	L
H-H-0.2719	0	12.144219	-0.634662	-0.320065	L
C-CT--0.0014	-1	12.813689	-2.637476	-0.000309	L
H-H1-0.0876	0	13.695888	-3.153757	-0.381461	L
C-C-0.5973	0	11.589186	-3.132223	-0.822748	L
O-O--0.5679	0	10.829280	-2.331910	-1.377269	L
C-CT--0.0152	0	12.679077	-2.955854	1.499692	L
H-HC-0.0295	0	13.623574	-2.707787	1.986466	L
H-HC-0.0295	0	12.532863	-4.029305	1.624581	L
C-CA--0.0011	-1	11.558164	-2.221605	2.215869	L
C-CA--0.1906	0	11.820198	-0.986850	2.839758	L
H-HA-0.1699	0	12.821952	-0.579513	2.821963	L
C-CA--0.1906	0	10.252532	-2.751953	2.239426	L
H-HA-0.1699	0	10.041393	-3.703852	1.772589	L
C-CA--0.2341	0	10.784135	-0.285189	3.483960	L
H-HA-0.1656	0	10.989187	0.649927	3.973458	L
C-CA--0.2341	0	9.211820	-2.048402	2.877661	L
H-HA-0.1656	0	8.214101	-2.460807	2.894874	L
C-C-0.3226	0	9.474646	-0.811036	3.505253	L
O-OH--0.557	0	8.477916	-0.125914	4.129278	L
H-HO-0.3992	0	7.664249	-0.670806	4.156230	L
N-N--0.415700	0	11.379714	-4.450644	-0.939306	L
H-H-0.271900	0	12.000646	-5.069512	-0.441679	L
C-CT--0.025200	-1	10.258667	-5.048572	-1.693936	L
H-H1-0.069800	0	10.475771	-6.099943	-1.881495	L
H-H1-0.069800	0	10.153917	-4.550266	-2.656979	L
C-C-0.597300	0	8.932310	-4.975806	-0.924725	L
O-O--0.567900	0	8.866941	-5.377674	0.235692	L
N-N--0.415700	0	7.861305	-4.488477	-1.557870	L
H-H-0.271900	0	7.943530	-4.354317	-2.558477	L
C-CT-0.014300	-1	6.524498	-4.464688	-0.965199	L
H-H1-0.104800	0	6.621059	-4.367433	0.119091	L
C-C-0.597300	0	5.785882	-5.775101	-1.243486	L
O-O--0.567900	0	5.066451	-5.905448	-2.239188	L
C-CT--0.204100	0	5.709200	-3.241645	-1.443451	L
H-HC-0.079700	0	5.572779	-3.295047	-2.523247	L
H-HC-0.079700	0	6.283223	-2.354950	-1.211719	L
C-C-0.713000	-1	4.326611	-3.106800	-0.782641	L
O-O--0.593100	0	3.938462	-3.857760	0.100660	L
N-N--0.919100	0	3.510656	-2.184833	-1.246016	L
H-H-0.419600	0	2.622901	-2.097211	-0.784105	L
H-H-0.419600	0	3.732574	-1.569189	-2.017754	L
N-N--0.347900	0	5.935737	-6.719920	-0.312884	L
H-H-0.274700	0	6.606571	-6.495480	0.412097	L

C-CT--0.263700 -1 5.384189 -8.079292 -0.375911 L
H-H1-0.156000 0 5.765332 -8.620117 0.490499 L
C-C-0.734100 0 5.890369 -8.872870 -1.591134 L
O-O--0.589400 0 6.895336 -8.527851 -2.205844 L
C-CT--0.000700 0 3.842608 -8.053101 -0.268542 L
H-HC-0.032700 0 3.467566 -9.076254 -0.225097 L
H-HC-0.032700 0 3.430512 -7.586876 -1.164713 L
C-CT-0.039000 0 3.299585 -7.328733 0.967760 L
H-HC-0.028500 0 3.656200 -6.300778 0.993514 L
H-HC-0.028500 0 3.621385 -7.844560 1.873540 L
C-CT-0.048600 0 1.771820 -7.340193 0.875282 L
H-H1-0.068700 0 1.421862 -8.374157 0.874220 L
H-H1-0.068700 0 1.461344 -6.878604 -0.064502 L
N-N2--0.529500 -1 1.139761 -6.632747 1.992290 L
H-H-0.345600 0 0.723877 -7.171206 2.747034 L
C-CA-0.807600 0 0.904579 -5.354482 2.091716 L
N-N2--0.862700 0 0.063677 -4.949429 2.985989 L
H-H-0.447800 0 -0.385019 -5.590891 3.624977 L
H-H-0.447800 0 -0.157789 -3.951386 3.018583 L
N-N2--0.862700 0 1.476034 -4.471756 1.328843 L
H-H-0.447800 0 2.358120 -4.643077 0.861412 L
H-H-0.447800 0 1.200666 -3.519728 1.572058 L
N-N--0.4157 0 5.210407 -9.981961 -1.889295 L
H-H-0.2719 0 4.417763 -10.190660 -1.306756 L
C-CT--0.1490 -1 5.513537 -10.875435 -2.992194 L
H-H1-0.0976 0 6.526298 -11.268942 -2.880370 L
H-H1-0.0976 0 4.806647 -11.706293 -3.013476 L
H-H1-0.0976 0 5.456276 -10.329162 -3.935849 L
C-CT--0.3662 -1 10.160479 -5.932745 -7.767609 L
H-HC-0.1123 0 9.876856 -6.640465 -8.544056 L
H-HC-0.1123 0 11.059517 -5.400479 -8.076948 L
H-HC-0.1123 0 10.355414 -6.453178 -6.831461 L
C-C-0.5972 0 9.043476 -4.929497 -7.562397 L
O-O--0.5679 0 9.266415 -3.733389 -7.713823 L
N-N--0.347900 0 7.848182 -5.383805 -7.154229 L
H-H-0.274700 0 7.770361 -6.378926 -7.007784 L
C-CT--0.263700 -1 6.875107 -4.482913 -6.501181 L
H-H1-0.156000 0 6.690979 -3.623612 -7.146977 L
C-C-0.734100 0 7.531380 -3.981308 -5.211696 L
O-O--0.589400 0 7.913968 -4.785776 -4.366827 L
C-CT--0.000700 0 5.542335 -5.176167 -6.171321 L
H-HC-0.032700 0 4.960331 -4.479643 -5.567905 L
H-HC-0.032700 0 5.721011 -6.063156 -5.559613 L
C-CT-0.039000 0 4.728794 -5.571683 -7.415162 L
H-HC-0.028500 0 4.963146 -6.606836 -7.666678 L
H-HC-0.028500 0 5.010589 -4.946571 -8.264072 L
C-CT-0.048600 0 3.213235 -5.438063 -7.184439 L
H-H1-0.068700 0 2.950380 -5.858791 -6.211680 L
H-H1-0.068700 0 2.698805 -6.025546 -7.947256 L
N-N2--0.529500 0 2.768500 -4.031842 -7.303462 L
H-H-0.345600 0 2.726042 -3.649163 -8.231326 L
C-CA-0.807600 0 2.457288 -3.184138 -6.336687 L
N-N2--0.862700 0 2.409139 -3.495798 -5.080283 L
H-H-0.447800 0 2.578888 -4.433717 -4.731432 L
H-H-0.447800 0 2.191069 -2.739994 -4.424668 L

N-N2--0.862700	0	2.187462	-1.942626	-6.601211	L
H-H-0.447800	0	2.214281	-1.530853	-7.512461	L
H-H-0.447800	0	2.042621	-1.378395	-5.760485	L
N-N--0.415700	0	7.716887	-2.672728	-5.073209	L
H-H-0.271900	0	7.385628	-2.071175	-5.809473	L
C-CT--0.025200	-1	8.620464	-2.098269	-4.073741	L
H-H1-0.069800	0	9.503315	-1.720175	-4.589986	L
H-H1-0.069800	0	8.961228	-2.856398	-3.373439	L
C-C-0.597300	0	8.031350	-0.952587	-3.271479	L
O-O--0.567900	0	6.983963	-0.401065	-3.612221	L
N-N--0.415700	0	8.716208	-0.572712	-2.196026	L
H-H-0.271900	0	9.566080	-1.072746	-1.938356	L
C-CT--0.038900	-1	8.307550	0.557390	-1.367372	L
H-H1-0.100700	0	7.676538	1.188343	-1.989712	L
C-C-0.597300	0	9.442447	1.485710	-0.960888	L
O-O--0.567900	0	10.551728	1.070730	-0.629133	L
C-CT-0.365400	0	7.414038	0.109298	-0.194804	L
H-H1-0.004300	0	7.727908	-0.879031	0.144220	L
O-OH--0.676100	0	6.094763	0.049458	-0.701182	L
H-HO-0.410200	0	6.161964	-0.143093	-1.647650	L
C-CT--0.243800	0	7.343497	1.041093	1.014628	L
H-HC-0.064200	0	6.987992	2.026355	0.714687	L
H-HC-0.064200	0	8.324258	1.124272	1.481514	L
H-HC-0.064200	0	6.651184	0.623957	1.746509	L
N-N--0.415700	0	9.117031	2.777734	-1.021530	L
H-H-0.271900	0	8.148989	2.982531	-1.248902	L
C-CT--0.025200	-1	9.956912	3.906426	-0.668474	L
H-H1-0.069800	0	9.762059	4.726597	-1.359326	L
H-H1-0.069800	0	11.007638	3.624152	-0.754056	L
C-C-0.597300	0	9.658922	4.387824	0.738885	L
O-O--0.567900	0	8.510686	4.599755	1.141816	L
N-N--0.347900	0	10.732914	4.593875	1.485423	L
H-H-0.274700	0	11.639116	4.437329	1.059033	L
C-CT--0.263700	-1	10.741413	5.186917	2.813441	L
H-H1-0.156000	0	9.835048	5.769842	2.966588	L
C-C-0.734100	0	11.910705	6.143627	2.915686	L
O-O--0.589400	0	12.859289	6.063133	2.140643	L
C-CT--0.000700	0	10.893989	4.082472	3.881428	L
H-HC-0.032700	0	11.173181	4.528699	4.837556	L
H-HC-0.032700	0	11.705191	3.413360	3.586555	L
C-CT-0.039000	0	9.629013	3.251063	4.102272	L
H-HC-0.028500	0	9.866458	2.424419	4.771362	L
H-HC-0.028500	0	9.282540	2.838376	3.154357	L
C-CT-0.048600	0	8.525497	4.084469	4.756593	L
H-H1-0.068700	0	8.333407	4.987666	4.178274	L
H-H1-0.068700	0	8.846789	4.380832	5.756817	L
N-N2--0.529500	-1	7.294820	3.300493	4.857008	L
H-H-0.345600	0	7.201547	2.666588	5.631999	L
C-CA-0.807600	0	6.317369	3.248538	3.972005	L
N-N2--0.862700	0	5.294054	2.477736	4.165860	L
H-H-0.447800	0	5.269563	1.778764	4.882397	L
H-H-0.447800	0	4.696047	2.353631	3.344609	L
N-N2--0.862700	0	6.323730	3.938228	2.872274	L
H-H-0.447800	0	7.166863	4.342824	2.491412	L
H-H-0.447800	0	5.473741	3.907151	2.306314	L

N-N--0.4157	0	11.827386	7.024851	3.903208	L
H-H-0.2719	0	11.010454	7.009715	4.489790	L
C-CT--0.1490	-1	12.892146	7.948889	4.199644	L
H-H1-0.0976	0	12.625340	8.577362	5.050869	L
H-H1-0.0976	0	13.807798	7.400795	4.433929	L
H-H1-0.0976	0	13.084657	8.585904	3.333089	L
C-CT--0.3662	-1	-2.179945	13.240555	-1.538374	L
H-HC-0.1123	0	-2.599823	13.750584	-0.673879	L
H-HC-0.1123	0	-1.093073	13.227241	-1.486511	L
H-HC-0.1123	0	-2.487744	13.760053	-2.445596	L
C-C-0.5972	0	-2.703036	11.816941	-1.595259	L
O-O--0.5679	0	-3.356819	11.443792	-2.556503	L
N-N--0.415700	0	-2.422738	11.030808	-0.552349	L
H-H-0.271900	0	-1.860808	11.448906	0.169974	L
C-CT--0.023700	-1	-2.842802	9.623108	-0.383749	L
H-H1-0.088000	0	-2.374361	9.032814	-1.172898	L
C-C-0.597300	0	-4.362111	9.437389	-0.543111	L
O-O--0.567900	0	-4.823658	8.648735	-1.367903	L
C-CT-0.034200	0	-2.302635	9.142002	0.968709	L
H-HC-0.024100	0	-2.683351	9.787720	1.761125	L
H-HC-0.024100	0	-1.215328	9.224862	0.963394	L
C-CT-0.001800	0	-2.678983	7.702033	1.301909	L
H-H1-0.044000	0	-3.751852	7.666714	1.494531	L
H-H1-0.044000	0	-2.167525	7.441776	2.224293	L
S-S--0.273700	0	-2.296556	6.438525	0.063830	L
C-CT--0.053600	0	-0.496526	6.466757	0.029819	L
H-H1-0.068400	0	-0.141111	5.780318	-0.738283	L
H-H1-0.068400	0	-0.127237	6.145347	1.001472	L
H-H1-0.068400	0	-0.143678	7.471690	-0.198980	L
N-N--0.4157	0	-5.146554	10.175659	0.246614	L
H-H-0.2719	0	-4.690490	10.794838	0.897023	L
C-CT--0.1490	-1	-6.597494	10.189495	0.149097	L
H-H1-0.0976	0	-7.019050	10.855245	0.903497	L
H-H1-0.0976	0	-6.894358	10.537325	-0.842695	L
H-H1-0.0976	0	-6.986998	9.181234	0.301006	L
C-CT--0.3662	-1	-4.453891	8.973657	-5.134751	L
H-HC-0.1123	0	-4.026181	9.333293	-4.199699	L
H-HC-0.1123	0	-3.690354	8.979860	-5.910547	L
H-HC-0.1123	0	-5.283055	9.609781	-5.435929	L
C-C-0.5972	0	-4.957687	7.548840	-4.952212	L
O-O--0.5679	0	-4.654320	6.686789	-5.764600	L
N-N--0.415700	0	-5.736344	7.304930	-3.894482	L
H-H-0.271900	0	-5.770572	8.055038	-3.216069	L
C-CT--0.087500	-1	-6.400263	6.014195	-3.568287	L
H-H1-0.096900	0	-6.109054	5.265238	-4.303969	L
C-C-0.597300	0	-7.913132	6.178754	-3.677869	L
O-O--0.567900	0	-8.548594	5.508810	-4.482381	L
C-CT-0.298500	0	-5.961316	5.526958	-2.179193	L
H-HC--0.029700	0	-6.175075	6.296480	-1.436845	L
C-CT--0.319200	0	-6.645648	4.232840	-1.735462	L
H-HC-0.079100	0	-6.433496	3.449742	-2.457228	L
H-HC-0.079100	0	-7.721928	4.378524	-1.654215	L
H-HC-0.079100	0	-6.270105	3.925462	-0.759656	L
C-CT--0.319200	0	-4.462005	5.251297	-2.194224	L
H-HC-0.079100	0	-4.221672	4.497316	-2.939715	L

H-HC-0.079100	0	-4.176015	4.902616	-1.211781	L
H-HC-0.079100	0	-3.905894	6.162259	-2.413455	L
N-N--0.4157	0	-8.498518	7.080330	-2.881437	L
H-H-0.2719	0	-7.891586	7.603470	-2.270408	L
C-CT--0.1490	-1	-9.928257	7.369824	-2.911575	L
H-H1-0.0976	0	-10.175605	8.140430	-2.180924	L
H-H1-0.0976	0	-10.209814	7.714019	-3.909202	L
H-H1-0.0976	0	-10.491704	6.461635	-2.688718	L
C-CT-0.1200	-1	-8.867843	1.943606	3.242267	L
H-HC-0.0800	0	-8.126916	2.735242	3.343130	L
H-HC-0.0800	0	-9.672523	2.292822	2.595430	L
H-HC-0.0800	0	-9.279142	1.703498	4.223261	L
C-CT-0.2000	0	-8.249613	0.724653	2.643482	L
H-H1-0.0800	0	-9.021511	-0.036150	2.497580	L
O-OS--0.5600	0	-7.646687	1.073653	1.417388	L
C-CT-0.2000	0	-7.128822	0.186060	3.523443	L
H-H1-0.0800	0	-6.439562	0.980611	3.811667	L
O-OH--0.6800	0	-7.602967	-0.504309	4.673693	L
H-HO-0.4000	0	-8.279672	-1.133220	4.378025	L
C-CT-0.2000	0	-6.485389	-0.691723	2.462470	L
H-H1-0.0800	0	-5.461249	-0.957272	2.730364	L
O-OH--0.6800	0	-7.308379	-1.843066	2.316874	L
H-HO-0.4000	0	-6.790556	-2.520215	1.842351	L
C-CT-0.5691	0	-6.543851	0.187564	1.203470	H
H-H2-0.8000	0	-6.700127	-0.471804	0.328127	H
N-N*--0.5691	0	-5.340417	0.997566	1.019063	H
C-CM--0.0500	0	-4.712473	0.815526	-0.177496	H
H-H4-0.1500	0	-5.057349	-0.046693	-0.700454	H
C-CM--0.1238	0	-3.788881	1.620242	-0.701637	H
C-C-0.6156	0	-3.496188	1.582742	-2.150631	H
O-O--0.5700	0	-2.699290	2.334097	-2.653619	H
N-N--0.8000	0	-4.250220	0.757137	-2.936074	H
H-H-0.3700	0	-3.932356	0.548946	-3.861017	H
H-H-0.3700	0	-4.892428	0.098955	-2.548959	H
C-CT-0.1164	0	-3.165932	2.669744	0.186855	H
H-HC-0.0800	0	-2.809216	3.504989	-0.395916	H
H-HC-0.0800	0	-2.286472	2.275024	0.692026	H
C-CM--0.2882	0	-4.190469	3.079606	1.217355	H
H-HA-0.1500	0	-4.114417	4.058025	1.648300	H
C-CM--0.0500	0	-5.154836	2.264292	1.604700	H
H-H4-0.1500	0	-5.858608	2.547837	2.356546	H
N-N3--0.8530	0	3.307513	1.073046	-0.531843	H
H-H-0.4500	0	3.457966	0.843775	-1.501001	H
H-H-0.4500	0	2.152977	0.558230	0.857114	H
H-H-0.4500	0	4.157404	0.848719	-0.034504	H
C-CT-0.3170	0	3.356073	2.525833	-0.411212	H
H-HP-0.0800	0	4.121095	2.941021	-1.061711	H
C-CT--0.1600	0	2.038870	3.234543	-0.725162	H
H-HC-0.0800	0	1.818576	3.094056	-1.777368	H
H-HC-0.0800	0	2.214854	4.295240	-0.589292	H
C-CT--0.1600	0	0.842562	2.805702	0.130088	H
H-HC-0.0800	0	0.063741	3.549486	0.037688	H
H-HC-0.0800	0	1.156680	2.820410	1.169316	H
C-CT--0.0990	0	0.266239	1.422500	-0.233554	H
H-HC-0.0800	0	0.879980	0.960110	-0.995848	H

H-HC-0.0800	0	-0.722153	1.538233	-0.653486	H
C-C-0.4490	0	0.222132	0.471370	0.951889	H
H-H-0.0600	0	-0.335287	0.912524	1.769593	H
O-O--0.5700	0	1.478879	0.117403	1.405231	H
N-N3--0.9900	0	-0.491207	-0.800866	0.582581	H
H-H-0.3600	0	0.156210	-1.640376	1.083855	H
H-H-0.3600	0	-0.626344	-0.804969	-0.422337	H
C-C-0.9060	0	3.857196	2.790874	1.013651	L
O-O2--0.9000	0	4.127317	1.845031	1.788945	L
O-O2--0.9000	0	4.022164	3.958288	1.417614	L
C-CT--0.205900	0	-1.703769	-1.226962	1.339753	H
H-H1-0.139900	0	-2.258889	-0.372035	1.699416	H
C-CT-0.007100	0	-2.575062	-2.099818	0.421905	H
H-HC--0.007800	0	-1.942148	-2.767706	-0.152422	H
H-HC--0.007800	0	-3.103835	-1.462658	-0.276573	H
C-CT-0.067500	0	-3.583754	-2.967963	1.177047	L
H-HC--0.054800	0	-3.889408	-2.440642	2.082336	L
H-HC--0.054800	0	-3.105156	-3.904685	1.471640	L
C-C-0.818300	0	-4.856209	-3.250676	0.375081	L
O-O2--0.822000	0	-5.705406	-2.328548	0.297976	L
O-O2--0.822000	0	-5.105510	-4.370171	-0.123077	L
C-C-0.742000	0	-1.074081	-2.062842	2.481665	H
O-O2--0.793000	0	-1.665394	-2.398122	3.476392	H
O-O2--0.793000	0	0.099172	-2.388292	2.148957	H
O-OW--0.834000	0	2.737184	-5.796164	-3.525065	L
H-HW-0.417000	0	3.599124	-5.920167	-3.077366	L
H-HW-0.417000	0	2.146238	-5.547785	-2.797817	L
O-OW--0.834000	0	0.193593	-7.404270	4.602787	L
H-HW-0.417000	0	0.867098	-7.051714	5.217969	L
H-HW-0.417000	0	-0.370653	-7.928410	5.177550	L
O-OW--0.834000	0	4.023365	-0.259588	3.175356	L
H-HW-0.417000	0	3.132650	-0.556186	2.953664	L
H-HW-0.417000	0	4.122207	0.542101	2.602752	L
O-OW--0.834000	0	6.155225	-1.518319	4.220537	L
H-HW-0.417000	0	5.836259	-2.423853	4.376266	L
H-HW-0.417000	0	5.370210	-1.084382	3.824082	L
O-OW--0.834000	0	-6.105808	-6.863337	0.054706	L
H-HW-0.417000	0	-5.773368	-5.944425	0.027914	L
H-HW-0.417000	0	-6.624902	-6.963386	-0.750865	L
O-OW--0.834000	0	-9.086586	-0.834676	-0.184242	L
H-HW-0.417000	0	-9.962387	-0.826680	0.211219	L
H-HW-0.417000	0	-8.870865	-1.794115	-0.209762	L

I2

C-CT--0.3662	-1	-12.872316	1.019172	1.939563	L
H-HC-0.1123	0	-12.294403	1.895889	1.650508	L
H-HC-0.1123	0	-13.931974	1.232588	1.809489	L
H-HC-0.1123	0	-12.580696	0.171054	1.323922	L
C-C-0.5972	0	-12.601417	0.703924	3.397874	L
O-O--0.5679	0	-11.849913	1.428340	4.039934	L
N-N--0.415700	0	-13.189514	-0.383952	3.906035	L
H-H-0.271900	0	-13.784027	-0.909625	3.286264	L
C-CT--0.025200	-1	-12.900741	-0.946138	5.238579	L
H-H1-0.069800	0	-13.637789	-1.714182	5.472330	L
H-H1-0.069800	0	-12.967234	-0.153986	5.985048	L

C-C-0.597300	0	-11.477093	-1.577523	5.315823	L
O-O--0.567900	0	-10.640217	-1.400018	4.424273	L
N-N--0.415700	0	-11.184130	-2.348723	6.374376	L
H-H-0.271900	0	-11.905793	-2.489836	7.063493	L
C-CT--0.025200	-1	-9.986657	-3.216204	6.449334	L
H-H1-0.069800	0	-9.900283	-3.636316	7.451077	L
H-H1-0.069800	0	-9.092826	-2.630543	6.230842	L
C-C-0.597300	0	-10.115600	-4.361819	5.454216	L
O-O--0.567900	0	-11.155761	-5.012349	5.384157	L
N-N--0.4157	0	-9.058954	-4.601119	4.687206	L
H-H-0.2719	0	-8.227604	-4.054594	4.836048	L
C-CT--0.0014	-1	-9.000359	-5.556761	3.582724	L
H-H1-0.0876	0	-9.910827	-5.480918	2.987331	L
C-C-0.5973	0	-8.871773	-7.027091	4.041391	L
O-O--0.5679	0	-8.081306	-7.804111	3.512934	L
C-CT--0.0152	0	-7.810958	-5.147937	2.697566	L
H-HC-0.0295	0	-8.048765	-4.176892	2.283303	L
H-HC-0.0295	0	-7.770188	-5.826653	1.846347	L
C-CA--0.0011	0	-6.422204	-5.074089	3.343466	L
C-CA--0.1906	0	-5.488547	-6.104827	3.115755	L
H-HA-0.1699	0	-5.787902	-6.982631	2.555688	L
C-CA--0.1906	0	-6.018806	-3.945175	4.091351	L
H-HA-0.1699	0	-6.707784	-3.145844	4.304367	L
C-CA--0.2341	0	-4.161905	-5.992239	3.576814	L
H-HA-0.1656	0	-3.445121	-6.770769	3.370515	L
C-CA--0.2341	0	-4.687578	-3.813938	4.530915	L
H-HA-0.1656	0	-4.359176	-2.929334	5.051659	L
C-C-0.3326	0	-3.750766	-4.832060	4.262037	L
O-OH--0.5579	0	-2.452071	-4.670684	4.623622	L
H-HO-0.3992	0	-2.130688	-3.824545	4.234293	L
N-N--0.4157	0	-9.659650	-7.428670	5.039499	L
H-H-0.2719	0	-10.343469	-6.759080	5.372704	L
C-CT--0.1490	-1	-9.570876	-8.748199	5.641133	L
H-H1-0.0976	0	-8.565375	-8.907105	6.035518	L
H-H1-0.0976	0	-10.297234	-8.843646	6.448943	L
H-H1-0.0976	0	-9.771089	-9.509911	4.884721	L
C-CT--0.3662	-1	-12.003912	-1.466855	-3.474223	L
H-HC-0.1123	0	-12.523675	-0.556212	-3.767867	L
H-HC-0.1123	0	-12.008004	-1.578748	-2.392337	L
H-HC-0.1123	0	-12.500017	-2.324043	-3.928454	L
C-C-0.5972	0	-10.573460	-1.415812	-3.975483	L
O-O--0.5679	0	-10.113754	-2.280249	-4.709002	L
N-N--0.415700	0	-9.849543	-0.402262	-3.523680	L
H-H-0.271900	0	-10.339135	0.269507	-2.948943	L
C-CT--0.038900	-1	-8.407191	-0.114439	-3.681831	L
H-H1-0.100700	0	-8.109313	-0.137435	-4.728004	L
C-C-0.597300	0	-7.561009	-1.094615	-2.868802	L
O-O--0.567900	0	-6.767965	-0.701258	-2.009418	L
C-CT-0.365400	0	-8.230576	1.310008	-3.103793	L
H-H1-0.004300	0	-7.172994	1.563514	-3.087390	L
O-OH--0.676100	0	-8.771991	1.401432	-1.794507	L
H-HO-0.410200	0	-8.621587	0.569665	-1.320197	L
C-CT--0.243800	0	-9.005256	2.387842	-3.859557	L
H-HC-0.064200	0	-10.059144	2.135302	-3.960455	L
H-HC-0.064200	0	-8.583017	2.540672	-4.842945	L

H-HC-0.064200	0	-8.940510	3.325408	-3.314419	L
N-N--0.415700	0	-7.777201	-2.386228	-3.075865	L
H-H-0.271900	0	-8.443693	-2.649129	-3.798272	L
C-CT--0.024900	-1	-7.196642	-3.463447	-2.283075	L
H-H1-0.084300	0	-6.322024	-3.093639	-1.768364	L
C-C-0.597300	0	-6.697663	-4.598366	-3.181128	L
O-O--0.567900	0	-7.202324	-4.783534	-4.286128	L
C-CT-0.211700	0	-8.210107	-4.059160	-1.306826	L
H-H1-0.035200	0	-7.941057	-5.087925	-1.062462	L
H-H1-0.035200	0	-9.214584	-4.042896	-1.732902	L
O-OH--0.654600	0	-8.147544	-3.295776	-0.135463	L
H-HO-0.427500	0	-7.189991	-3.267530	0.081085	L
N-N--0.4157	0	-5.719157	-5.378633	-2.712258	L
H-H-0.2719	0	-5.334103	-5.157434	-1.795115	L
C-CT--0.0014	0	-5.318218	-6.620755	-3.382929	L
H-H1-0.0876	0	-4.998285	-6.378230	-4.396361	L
C-C-0.5973	0	-6.465297	-7.629883	-3.458030	L
O-O--0.5679	0	-7.348341	-7.647607	-2.599125	L
C-CT--0.0152	0	-4.173079	-7.331166	-2.652216	L
H-HC-0.0295	0	-3.897221	-8.218239	-3.224004	L
H-HC-0.0295	0	-4.523781	-7.694345	-1.688339	L
C-CA--0.0011	-1	-2.919553	-6.532731	-2.438882	L
C-CA--0.1906	0	-1.986434	-6.394404	-3.487338	L
H-HA-0.1699	0	-2.208361	-6.802062	-4.463067	L
C-CA--0.1906	0	-2.611408	-6.029540	-1.160644	L
H-HA-0.1699	0	-3.315809	-6.151683	-0.345771	L
C-CA--0.2341	0	-0.753859	-5.750397	-3.259609	L
H-HA-0.1656	0	-0.031731	-5.643514	-4.054003	L
C-CA--0.2341	0	-1.381548	-5.386483	-0.928728	L
H-HA-0.1656	0	-1.161535	-5.006790	0.057350	L
C-C-0.3226	0	-0.449335	-5.246404	-1.977680	L
O-OH--0.5579	0	0.744207	-4.636973	-1.750592	L
H-HO-0.3992	0	0.660328	-4.099338	-0.954476	L
N-N--0.4157	0	-6.357285	-8.540810	-4.429031	L
H-H-0.2719	0	-5.607188	-8.421775	-5.089573	L
C-CT--0.1490	-1	-7.141802	-9.759252	-4.489286	L
H-H1-0.0976	0	-8.201586	-9.507623	-4.570419	L
H-H1-0.0976	0	-6.848641	-10.358964	-5.351845	L
H-H1-0.0976	0	-6.994009	-10.340200	-3.576429	L
C-CT--0.3662	-1	-6.333528	-3.505563	-6.968784	L
H-HC-0.1123	0	-5.830722	-4.254488	-7.577180	L
H-HC-0.1123	0	-7.375459	-3.792642	-6.832103	L
H-HC-0.1123	0	-5.843542	-3.428604	-6.003526	L
C-C-0.5972	0	-6.253088	-2.158545	-7.670443	L
O-O--0.5679	0	-5.367696	-1.985817	-8.496343	L
N-N--0.415700	0	-7.149052	-1.213856	-7.346748	L
H-H-0.271900	0	-7.863500	-1.488155	-6.689778	L
C-CT--0.025200	-1	-7.288059	0.103936	-8.017362	L
H-H1-0.069800	0	-8.236874	0.119097	-8.553981	L
H-H1-0.069800	0	-6.500461	0.234970	-8.758871	L
C-C-0.597300	0	-7.264128	1.312438	-7.087497	L
O-O--0.567900	0	-8.148060	2.152620	-7.187667	L
N-N--0.415700	0	-6.260046	1.398353	-6.208904	L
H-H-0.271900	0	-5.573236	0.652378	-6.231585	L
C-CT--0.025200	-1	-5.827180	2.635622	-5.548118	L

H-H1-0.069800	0	-5.650716	2.451834	-4.491361	L
H-H1-0.069800	0	-6.588366	3.410241	-5.652383	L
C-C-0.597300	0	-4.540656	3.151452	-6.202633	L
O-O--0.567900	0	-4.478588	3.283113	-7.422148	L
N-N--0.415700	0	-3.496842	3.381580	-5.403596	L
H-H-0.271900	0	-3.613259	3.228301	-4.411698	L
C-CT--0.051800	-1	-2.160246	3.772457	-5.867789	L
H-H1-0.092200	0	-2.246518	4.228160	-6.855650	L
C-C-0.597300	0	-1.238396	2.541944	-5.995851	L
O-O--0.567900	0	-0.551958	2.340579	-6.994591	L
C-CT--0.110200	0	-1.615510	4.824491	-4.881320	L
H-HC-0.045700	0	-1.582460	4.386554	-3.883518	L
H-HC-0.045700	0	-2.314407	5.661657	-4.851077	L
C-CT-0.353100	0	-0.215998	5.368013	-5.213476	L
H-HC--0.036100	0	0.500626	4.546880	-5.232671	L
C-CT--0.412100	0	-0.172212	6.098833	-6.555343	L
H-HC-0.100000	0	-0.926835	6.886103	-6.576637	L
H-HC-0.100000	0	-0.367229	5.396385	-7.365634	L
H-HC-0.100000	0	0.813744	6.535401	-6.710196	L
C-CT--0.412100	0	0.208625	6.349535	-4.118870	L
H-HC-0.100000	0	-0.500479	7.176709	-4.067588	L
H-HC-0.100000	0	1.201376	6.741384	-4.338474	L
H-HC-0.100000	0	0.234776	5.835994	-3.158976	L
N-N--0.516300	0	-1.195011	1.728651	-4.944010	L
H-H-0.293600	0	-1.775667	2.004671	-4.158732	L
C-CT-0.038100	-1	-0.302454	0.593423	-4.723849	L
H-H1-0.088000	0	-0.065613	0.098020	-5.663027	L
C-C-0.536600	0	-0.984342	-0.392295	-3.740748	L
O-O--0.581900	0	-0.848068	-0.240492	-2.529571	L
C-CT--0.030300	0	1.004340	1.184169	-4.118303	L
H-HC--0.012200	0	0.742670	1.807159	-3.263892	L
H-HC--0.012200	0	1.463937	1.836465	-4.862958	L
C-C-0.799400	-1	2.062719	0.163560	-3.673446	L
O-O2--0.801400	0	1.934476	-1.018713	-4.067742	L
O-O2--0.801400	0	3.102992	0.548348	-3.098239	L
N-N--0.254800	0	-1.761222	-1.382866	-4.211994	L
C-CT--0.026600	-1	-2.049215	-1.679942	-5.609839	L
H-H1-0.064100	0	-1.123819	-1.732397	-6.182159	L
C-C-0.589600	0	-2.996245	-0.659816	-6.268184	L
O-O--0.574800	0	-3.936022	-0.176873	-5.633386	L
C-CT--0.007000	0	-2.688412	-3.072579	-5.582335	L
H-HC-0.025300	0	-1.904808	-3.831898	-5.585554	L
H-HC-0.025300	0	-3.371692	-3.232555	-6.417108	L
C-CT-0.018900	0	-3.411664	-3.098874	-4.236979	L
H-HC-0.021300	0	-3.559309	-4.113684	-3.876660	L
H-HC-0.021300	0	-4.364739	-2.573680	-4.310790	L
C-CT-0.019200	0	-2.463998	-2.312972	-3.337974	L
H-H1-0.039100	0	-3.027072	-1.784231	-2.569168	L
H-H1-0.039100	0	-1.743561	-2.985486	-2.873484	L
N-N--0.4157	0	-2.750382	-0.366517	-7.549919	L
H-H-0.2719	0	-1.961076	-0.812847	-7.984082	L
C-CT--0.1490	-1	-3.542274	0.516847	-8.405632	L
H-H1-0.0976	0	-4.503320	0.733373	-7.952379	L
H-H1-0.0976	0	-3.719020	0.041955	-9.371694	L
H-H1-0.0976	0	-3.011852	1.459313	-8.559355	L

C-CT--0.3662	-1	8.745675	1.913213	-5.916371	L
H-HC-0.1123	0	9.246867	2.173168	-6.848348	L
H-HC-0.1123	0	9.491243	1.674349	-5.160884	L
H-HC-0.1123	0	8.087312	1.063250	-6.084217	L
C-C-0.5972	0	7.932663	3.101919	-5.453900	L
O-O--0.5679	0	8.090853	4.190517	-5.986630	L
N-N--0.415700	0	7.063880	2.902531	-4.462424	L
H-H-0.271900	0	6.985732	1.966878	-4.088481	L
C-CT-0.021300	-1	6.077036	3.900521	-4.024784	L
H-H1-0.112400	0	6.273007	4.853344	-4.520944	L
C-C-0.597300	0	6.113259	4.139748	-2.512899	L
O-O--0.567900	0	6.373288	3.222520	-1.733651	L
C-CT--0.123100	0	4.700020	3.394603	-4.484997	L
H-H1-0.111200	0	4.485383	2.433593	-4.012176	L
H-H1-0.111200	0	4.713512	3.254552	-5.567529	L
S-SH--0.311900	0	3.391403	4.578437	-4.060833	L
H-HS-0.193300	0	2.382007	3.925781	-4.655499	L
N-N--0.415700	0	5.832576	5.372045	-2.090757	L
H-H-0.271900	0	5.581683	6.064317	-2.783656	L
C-CT--0.025200	-1	5.819798	5.779436	-0.690970	L
H-H1-0.069800	0	6.784634	6.223808	-0.447279	L
H-H1-0.069800	0	5.685140	4.915684	-0.044357	L
C-C-0.597300	0	4.742304	6.817000	-0.401647	L
O-O--0.567900	0	4.846751	7.962805	-0.829831	L
N-N--0.415700	0	3.732874	6.436844	0.376184	L
H-H-0.271900	0	3.779606	5.491690	0.750397	L
C-CT--0.025200	-1	2.687887	7.307852	0.901931	L
H-H1-0.069800	0	1.862968	6.686954	1.235746	L
H-H1-0.069800	0	2.341113	7.997051	0.131352	L
C-C-0.597300	0	3.179798	8.102986	2.109617	L
O-O--0.567900	0	2.590976	8.012690	3.184708	L
N-N--0.4157	0	4.274566	8.848640	1.926124	L
H-H-0.2719	0	4.640489	8.854233	0.979009	L
C-CT--0.1490	-1	5.016598	9.532774	2.976044	L
H-H1-0.0976	0	5.340386	8.812426	3.729206	L
H-H1-0.0976	0	5.888919	10.033212	2.553015	L
H-H1-0.0976	0	4.374662	10.274884	3.454761	L
C-CT--0.3662	-1	-1.508473	9.426937	5.296692	L
H-HC-0.1123	0	-1.726685	10.394406	4.847338	L
H-HC-0.1123	0	-0.570617	9.042741	4.898901	L
H-HC-0.1123	0	-1.439372	9.538618	6.377249	L
C-C-0.5972	0	-2.641731	8.453918	4.959672	L
O-O--0.5679	0	-3.533835	8.811746	4.201050	L
N-N--0.4157	0	-2.607606	7.224329	5.490482	L
H-H-0.2719	0	-1.853016	7.006704	6.126749	L
C-CT--0.0275	-1	-3.602977	6.134677	5.313839	L
H-H1-0.1123	0	-4.600213	6.572717	5.261903	L
C-C-0.5973	0	-3.563238	5.155402	6.516678	L
O-O--0.5679	0	-2.637298	5.204625	7.324986	L
C-CT--0.0050	0	-3.325949	5.364495	4.013974	L
H-HC-0.0339	0	-3.509521	6.027317	3.175830	L
H-HC-0.0339	0	-4.065152	4.568550	3.930031	L
C-C*--0.1415	0	-1.961359	4.747848	3.862882	L
C-CW--0.1638	0	-0.801159	5.416133	3.653108	L
H-H4-0.2062	0	-0.707021	6.491103	3.570546	L

C-CB-0.1243	0	-1.595870	3.334545	3.924454	L
N-NA--0.3418	0	0.250088	4.522414	3.582068	L
H-H-0.3412	0	1.218140	4.793343	3.461044	L
C-CN-0.1380	0	-0.182369	3.226689	3.754165	L
C-CA--0.238	0	-2.314135	2.133233	4.118743	L
H-HA-0.1700	0	-3.385622	2.172739	4.228434	L
C-CA--0.2601	0	0.487745	1.996982	3.793376	L
H-HA-0.1572	0	1.557807	1.947497	3.653092	L
C-CA--0.1972	0	-1.652737	0.891218	4.152001	L
H-HA-0.1447	0	-2.208574	-0.029683	4.274631	L
C-CA--0.1134	0	-0.258311	0.825059	3.996987	L
H-HA-0.1417	0	0.233533	-0.136845	4.002549	L
N-N--0.4157	0	-4.553272	4.252128	6.635945	L
H-H-0.2719	0	-5.279770	4.291766	5.940029	L
C-CT--0.1490	-1	-4.644825	3.221628	7.685473	L
H-H1-0.0976	0	-3.829025	2.506656	7.567435	L
H-H1-0.0976	0	-4.557982	3.690767	8.668098	L
H-H1-0.0976	0	-5.599563	2.697539	7.621606	L
C-CT--0.3662	-1	-2.796220	-1.496434	7.275955	L
H-HC-0.1123	0	-3.023134	-2.551629	7.123118	L
H-HC-0.1123	0	-3.147241	-1.188905	8.258559	L
H-HC-0.1123	0	-3.280144	-0.911846	6.495952	L
C-C-0.5972	0	-1.304395	-1.305166	7.180656	L
O-O--0.5679	0	-0.590013	-2.257337	6.918114	L
N-N--0.415700	0	-0.808278	-0.100774	7.460807	L
H-H-0.271900	0	-1.466580	0.650986	7.592029	L
C-CT--0.087500	-1	0.634125	0.228948	7.419614	L
H-H1-0.096900	0	0.962875	0.127257	6.385172	L
C-C-0.597300	0	1.479732	-0.737709	8.260421	L
O-O--0.567900	0	2.468854	-1.276362	7.775899	L
C-CT-0.298500	0	0.914623	1.682064	7.872618	L
H-HC--0.029700	0	0.972200	1.710151	8.961366	L
C-CT--0.319200	0	2.258882	2.157191	7.308042	L
H-HC-0.079100	0	2.219702	2.175959	6.218472	L
H-HC-0.079100	0	3.058215	1.487505	7.626243	L
H-HC-0.079100	0	2.479912	3.160069	7.674101	L
C-CT--0.319200	0	-0.167001	2.688069	7.455989	L
H-HC-0.079100	0	-0.357852	2.612224	6.389432	L
H-HC-0.079100	0	0.155698	3.704587	7.683722	L
H-HC-0.079100	0	-1.091997	2.507770	8.001647	L
N-N--0.415700	0	1.066455	-1.001245	9.507500	L
H-H-0.271900	0	0.245385	-0.513174	9.826697	L
C-CT--0.025200	-1	1.676641	-2.005841	10.394319	L
H-H1-0.069800	0	1.140622	-2.031020	11.342488	L
H-H1-0.069800	0	2.710822	-1.723439	10.594823	L
C-C-0.597300	0	1.684303	-3.438204	9.809157	L
O-O--0.567900	0	2.491735	-4.254828	10.235040	L
N-N--0.415700	0	0.799945	-3.750274	8.849928	L
H-H-0.271900	0	0.205905	-3.012005	8.499498	L
C-CT-0.033700	-1	0.671509	-5.041773	8.161622	L
H-H1-0.082300	0	1.130750	-5.809502	8.787353	L
C-C-0.597300	0	1.396391	-5.106270	6.792275	L
O-O--0.567900	0	1.828546	-6.179229	6.370323	L
C-CT--0.182500	0	-0.816644	-5.392435	8.027344	L
H-HC-0.060300	0	-1.296072	-5.361620	9.006209	L

H-HC-0.060300	0	-0.917348	-6.398251	7.618641	L
H-HC-0.060300	0	-1.315753	-4.693769	7.355926	L
N-N--0.415700	0	1.530752	-3.978117	6.090600	L
H-H-0.271900	0	1.005995	-3.180705	6.431405	L
C-CT-0.033700	-1	2.465084	-3.729565	4.979745	L
H-H1-0.082300	0	2.316832	-4.459673	4.187864	L
C-C-0.597300	0	3.934676	-3.791288	5.452667	L
O-O--0.567900	0	4.836352	-4.083087	4.665987	L
C-CT--0.182500	0	2.161966	-2.321917	4.483743	L
H-HC-0.060300	0	2.624132	-1.581044	5.136046	L
H-HC-0.060300	0	1.086986	-2.155700	4.501790	L
H-HC-0.060300	0	2.545504	-2.216592	3.470529	L
N-N--0.4157	0	4.175599	-3.442709	6.724699	L
H-H-0.2719	0	3.381588	-3.075267	7.234966	L
C-CT--0.1490	-1	5.452832	-3.498996	7.421656	L
H-H1-0.0976	0	5.973938	-4.423478	7.165137	L
H-H1-0.0976	0	5.286462	-3.471342	8.499921	L
H-H1-0.0976	0	6.071280	-2.649320	7.127738	L
C-CT--0.3662	-1	14.088914	0.932788	-0.697142	L
H-HC-0.1123	0	13.441153	1.440251	0.014853	L
H-HC-0.1123	0	15.094545	1.346996	-0.641794	L
H-HC-0.1123	0	13.698950	1.050494	-1.706012	L
C-C-0.5972	0	14.146954	-0.547555	-0.358364	L
O-O--0.5679	0	15.228773	-1.108089	-0.248389	L
N-N--0.4157	0	12.986743	-1.186453	-0.211450	L
H-H-0.2719	0	12.143662	-0.637117	-0.330877	L
C-CT--0.0014	-1	12.812084	-2.640514	-0.012348	L
H-H1-0.0876	0	13.693681	-3.156841	-0.394835	L
C-C-0.5973	0	11.586495	-3.133191	-0.834424	L
O-O--0.5679	0	10.825941	-2.331459	-1.386057	L
C-CT--0.0152	0	12.678120	-2.960771	1.486793	L
H-HC-0.0295	0	13.623090	-2.713903	1.973274	L
H-HC-0.0295	0	12.531826	-4.034394	1.610142	L
C-CA--0.0011	-1	11.558084	-2.227517	2.205230	L
C-CA--0.1906	0	11.822674	-0.995576	2.833245	L
H-HA-0.1699	0	12.823981	-0.587283	2.812014	L
C-CA--0.1906	0	10.254271	-2.761050	2.234238	L
H-HA-0.1699	0	10.042301	-3.711560	1.764931	L
C-CA--0.2341	0	10.790412	-0.300183	3.489556	L
H-HA-0.1656	0	10.998139	0.631022	3.985107	L
C-CA--0.2341	0	9.217479	-2.063696	2.884585	L
H-HA-0.1656	0	8.221558	-2.479693	2.907427	L
C-C-0.3226	0	9.482842	-0.830086	3.518489	L
O-OH--0.557	0	8.490416	-0.154531	4.158743	L
H-HO-0.3992	0	7.691232	-0.718176	4.213879	L
N-N--0.415700	0	11.376760	-4.451322	-0.953767	L
H-H-0.271900	0	11.998293	-5.071368	-0.458354	L
C-CT--0.025200	-1	10.254740	-5.047467	-1.708359	L
H-H1-0.069800	0	10.471016	-6.098722	-1.897524	L
H-H1-0.069800	0	10.149640	-4.547762	-2.670633	L
C-C-0.597300	0	8.928975	-4.974895	-0.938153	L
O-O--0.567900	0	8.864445	-5.377017	0.222210	L
N-N--0.415700	0	7.857486	-4.487481	-1.570399	L
H-H-0.271900	0	7.938861	-4.352869	-2.571021	L
C-CT-0.014300	-1	6.521319	-4.462694	-0.976500	L

H-H1-0.104800	0	6.619661	-4.368789	0.107913	L
C-C-0.597300	0	5.778677	-5.770734	-1.255556	L
O-O--0.567900	0	5.053805	-5.896406	-2.247939	L
C-CT--0.204100	0	5.708599	-3.236738	-1.448064	L
H-HC-0.079700	0	5.574993	-3.283957	-2.528541	L
H-HC-0.079700	0	6.283410	-2.352344	-1.210122	L
C-C-0.713000	-1	4.324293	-3.103887	-0.790472	L
O-O--0.593100	0	3.935386	-3.844040	0.101981	L
N-N--0.919100	0	3.503610	-2.194089	-1.271324	L
H-H-0.419600	0	2.605726	-2.110492	-0.827425	L
H-H-0.419600	0	3.726056	-1.588050	-2.050779	L
N-N--0.347900	0	5.931709	-6.718562	-0.328372	L
H-H-0.274700	0	6.606070	-6.496906	0.394203	L
C-CT--0.263700	-1	5.379353	-8.077611	-0.392362	L
H-H1-0.156000	0	5.760655	-8.619666	0.473208	L
C-C-0.734100	0	5.884017	-8.869357	-1.609342	L
O-O--0.589400	0	6.887241	-8.522326	-2.225803	L
C-CT--0.000700	0	3.837608	-8.050755	-0.284559	L
H-HC-0.032700	0	3.463152	-9.073987	-0.237572	L
H-HC-0.032700	0	3.424953	-7.587923	-1.182257	L
C-CT-0.039000	0	3.294147	-7.321843	0.949013	L
H-HC-0.028500	0	3.646190	-6.292119	0.968626	L
H-HC-0.028500	0	3.620364	-7.831538	1.856692	L
C-CT-0.048600	0	1.766187	-7.339174	0.861390	L
H-H1-0.068700	0	1.419803	-8.374334	0.863140	L
H-H1-0.068700	0	1.450622	-6.879676	-0.078056	L
N-N2--0.529500	-1	1.137110	-6.632532	1.980647	L
H-H-0.345600	0	0.739959	-7.171278	2.745199	L
C-CA-0.807600	0	0.875491	-5.354990	2.065037	L
N-N2--0.862700	0	0.048119	-4.949789	2.972062	L
H-H-0.447800	0	-0.369093	-5.603667	3.620045	L
H-H-0.447800	0	-0.202676	-3.957654	3.027884	L
N-N2--0.862700	0	1.411930	-4.472507	1.278169	L
H-H-0.447800	0	2.285246	-4.646778	0.796918	L
H-H-0.447800	0	1.145341	-3.523361	1.526072	L
N-N--0.4157	0	5.204454	-9.978654	-1.907654	L
H-H-0.2719	0	4.413149	-10.188873	-1.323816	L
C-CT--0.1490	-1	5.505627	-10.869610	-3.013216	L
H-H1-0.0976	0	6.519975	-11.260359	-2.906199	L
H-H1-0.0976	0	4.801065	-11.702487	-3.032820	L
H-H1-0.0976	0	5.442827	-10.322080	-3.955786	L
C-CT--0.3662	-1	10.152515	-5.921813	-7.783387	L
H-HC-0.1123	0	9.867722	-6.626621	-8.562056	L
H-HC-0.1123	0	11.052056	-5.389607	-8.091368	L
H-HC-0.1123	0	10.347244	-6.445588	-6.849064	L
C-C-0.5972	0	9.036891	-4.918012	-7.574427	L
O-O--0.5679	0	9.260953	-3.721703	-7.722631	L
N-N--0.347900	0	7.841466	-5.372412	-7.166939	L
H-H-0.274700	0	7.762614	-6.367840	-7.023140	L
C-CT--0.263700	-1	6.868685	-4.472204	-6.512707	L
H-H1-0.156000	0	6.686054	-3.611567	-7.157158	L
C-C-0.734100	0	7.524325	-3.973139	-5.222071	L
O-O--0.589400	0	7.904450	-4.779109	-4.377630	L
C-CT--0.000700	0	5.534500	-5.164767	-6.187107	L
H-HC-0.032700	0	4.950755	-4.467926	-5.585841	L

H-HC-0.032700	0	5.710673	-6.051941	-5.574950	L
C-CT-0.039000	0	4.725562	-5.559767	-7.434254	L
H-HC-0.028500	0	4.961958	-6.594418	-7.685920	L
H-HC-0.028500	0	5.010019	-4.933677	-8.281559	L
C-CT-0.048600	0	3.208848	-5.428144	-7.209942	L
H-H1-0.068700	0	2.942416	-5.848662	-6.238103	L
H-H1-0.068700	0	2.698538	-6.016958	-7.974495	L
N-N2--0.529500	0	2.762244	-4.022749	-7.331965	L
H-H-0.345600	0	2.718961	-3.642053	-8.260602	L
C-CA-0.807600	0	2.450974	-3.173343	-6.366732	L
N-N2--0.862700	0	2.403504	-3.483210	-5.109899	L
H-H-0.447800	0	2.573204	-4.420871	-4.760303	L
H-H-0.447800	0	2.187314	-2.726052	-4.455319	L
N-N2--0.862700	0	2.180922	-1.932247	-6.633286	L
H-H-0.447800	0	2.206001	-1.522456	-7.545451	L
H-H-0.447800	0	2.038037	-1.365708	-5.793575	L
N-N--0.415700	0	7.713006	-2.665056	-5.082870	L
H-H-0.271900	0	7.384173	-2.062921	-5.819742	L
C-CT--0.025200	-1	8.616781	-2.092434	-4.082456	L
H-H1-0.069800	0	9.498846	-1.711934	-4.598279	L
H-H1-0.069800	0	8.958758	-2.852230	-3.384536	L
C-C-0.597300	0	8.026631	-0.949533	-3.276717	L
O-O--0.567900	0	6.976734	-0.400132	-3.613108	L
N-N--0.415700	0	8.714109	-0.569377	-2.203220	L
H-H-0.271900	0	9.565594	-1.068411	-1.948924	L
C-CT--0.038900	-1	8.306917	0.559039	-1.371634	L
H-H1-0.100700	0	7.670888	1.189727	-1.989012	L
C-C-0.597300	0	9.443790	1.486567	-0.968802	L
O-O--0.567900	0	10.554451	1.070549	-0.642948	L
C-CT-0.365400	0	7.423604	0.106697	-0.192637	L
H-H1-0.004300	0	7.741516	-0.882435	0.140261	L
O-OH--0.676100	0	6.099705	0.046268	-0.686568	L
H-HO-0.410200	0	6.158507	-0.137380	-1.635347	L
C-CT--0.243800	0	7.363145	1.033636	1.021490	L
H-HC-0.064200	0	7.005768	2.020519	0.728817	L
H-HC-0.064200	0	8.347662	1.113880	1.481061	L
H-HC-0.064200	0	6.676769	0.613893	1.757452	L
N-N--0.415700	0	9.118028	2.778599	-1.023230	L
H-H-0.271900	0	8.149411	2.984436	-1.247122	L
C-CT--0.025200	-1	9.958542	3.906032	-0.668313	L
H-H1-0.069800	0	9.764279	4.727192	-1.358165	L
H-H1-0.069800	0	11.009056	3.623180	-0.754626	L
C-C-0.597300	0	9.661852	4.386400	0.739303	L
O-O--0.567900	0	8.514040	4.595771	1.144132	L
N-N--0.347900	0	10.736344	4.593794	1.484442	L
H-H-0.274700	0	11.642353	4.440076	1.056537	L
C-CT--0.263700	-1	10.745783	5.180484	2.815199	L
H-H1-0.156000	0	9.839297	5.762183	2.972542	L
C-C-0.734100	0	11.915966	6.135782	2.920004	L
O-O--0.589400	0	12.864600	6.055865	2.144914	L
C-CT--0.000700	0	10.899888	4.069299	3.876636	L
H-HC-0.032700	0	11.182904	4.509791	4.834316	L
H-HC-0.032700	0	11.709744	3.401368	3.575419	L
C-CT-0.039000	0	9.635108	3.236448	4.097220	L
H-HC-0.028500	0	9.875523	2.401815	4.754909	L

H-HC-0.028500	0	9.280060	2.835936	3.147358	L
C-CT-0.048600	0	8.539664	4.064152	4.771236	L
H-H1-0.068700	0	8.356441	4.982262	4.213853	L
H-H1-0.068700	0	8.865803	4.335753	5.776928	L
N-N2--0.529500	-1	7.299339	3.292682	4.857743	L
H-H-0.345600	0	7.194082	2.652183	5.625793	L
C-CA-0.807600	0	6.311904	3.289742	3.984397	L
N-N2--0.862700	0	5.260059	2.563592	4.188544	L
H-H-0.447800	0	5.225586	1.850546	4.891075	L
H-H-0.447800	0	4.665258	2.451368	3.363747	L
N-N2--0.862700	0	6.334144	3.984893	2.888256	L
H-H-0.447800	0	7.190315	4.354601	2.500274	L
H-H-0.447800	0	5.487030	3.964206	2.317899	L
N-N--0.4157	0	11.833574	7.015304	3.909012	L
H-H-0.2719	0	11.016903	7.000277	4.495955	L
C-CT--0.1490	-1	12.898855	7.939033	4.204585	L
H-H1-0.0976	0	12.633586	8.566490	5.057040	L
H-H1-0.0976	0	13.814954	7.390707	4.436593	L
H-H1-0.0976	0	13.089805	8.577098	3.338455	L
C-CT--0.3662	-1	-2.173652	13.248258	-1.516095	L
H-HC-0.1123	0	-2.590746	13.756502	-0.649207	L
H-HC-0.1123	0	-1.086664	13.233561	-1.467079	L
H-HC-0.1123	0	-2.483165	13.770483	-2.421167	L
C-C-0.5972	0	-2.698549	11.825407	-1.575432	L
O-O--0.5679	0	-3.355527	11.455485	-2.535723	L
N-N--0.415700	0	-2.416274	11.036602	-0.535150	L
H-H-0.271900	0	-1.851644	11.452776	0.186162	L
C-CT--0.023700	-1	-2.837838	9.629322	-0.366904	L
H-H1-0.088000	0	-2.370221	9.038172	-1.155914	L
C-C-0.597300	0	-4.357404	9.445101	-0.525710	L
O-O--0.567900	0	-4.820044	8.658694	-1.352008	L
C-CT-0.034200	0	-2.297290	9.148387	0.985830	L
H-HC-0.024100	0	-2.673853	9.797584	1.777453	L
H-HC-0.024100	0	-1.209693	9.227266	0.978348	L
C-CT-0.001800	0	-2.677951	7.710944	1.325408	L
H-H1-0.044000	0	-3.750475	7.680411	1.520772	L
H-H1-0.044000	0	-2.165621	7.452142	2.247864	L
S-S--0.273700	0	-2.303595	6.440444	0.092124	L
C-CT--0.053600	0	-0.503641	6.466363	0.045064	L
H-H1-0.068400	0	-0.154633	5.774549	-0.721257	L
H-H1-0.068400	0	-0.127737	6.150597	1.015961	L
H-H1-0.068400	0	-0.151095	7.469346	-0.192685	L
N-N--0.4157	0	-5.140925	10.182191	0.266018	L
H-H-0.2719	0	-4.684264	10.799871	0.917416	L
C-CT--0.1490	-1	-6.591902	10.196929	0.169047	L
H-H1-0.0976	0	-7.012931	10.860670	0.925504	L
H-H1-0.0976	0	-6.888939	10.547742	-0.821644	L
H-H1-0.0976	0	-6.981796	9.188393	0.318140	L
C-CT--0.3662	-1	-4.452059	8.988412	-5.118003	L
H-HC-0.1123	0	-4.023986	9.345185	-4.182021	L
H-HC-0.1123	0	-3.688205	8.994720	-5.893488	L
H-HC-0.1123	0	-5.279826	9.626836	-5.418140	L
C-C-0.5972	0	-4.958765	7.564304	-4.938771	L
O-O--0.5679	0	-4.657955	6.703949	-5.753863	L
N-N--0.415700	0	-5.736832	7.319248	-3.880845	L

H-H-0.271900	0	-5.769012	8.067822	-3.200666	L
C-CT--0.087500	-1	-6.399156	6.027509	-3.555164	L
H-H1-0.096900	0	-6.108856	5.280093	-4.292803	L
C-C-0.597300	0	-7.912466	6.190318	-3.660675	L
O-O--0.567900	0	-8.549781	5.517063	-4.460899	L
C-CT-0.298500	0	-5.956541	5.537991	-2.168157	L
H-HC--0.029700	0	-6.174802	6.303049	-1.422485	L
C-CT--0.319200	0	-6.632660	4.237492	-1.730337	L
H-HC-0.079100	0	-6.416647	3.459611	-2.456670	L
H-HC-0.079100	0	-7.709595	4.376813	-1.647191	L
H-HC-0.079100	0	-6.254097	3.927133	-0.756570	L
C-CT--0.319200	0	-4.455537	5.272499	-2.185436	L
H-HC-0.079100	0	-4.210227	4.529658	-2.940369	L
H-HC-0.079100	0	-4.167600	4.913625	-1.207317	L
H-HC-0.079100	0	-3.905186	6.189539	-2.393393	L
N-N--0.4157	0	-8.496257	7.094264	-2.865753	L
H-H-0.2719	0	-7.887866	7.619874	-2.258317	L
C-CT--0.1490	-1	-9.926014	7.384029	-2.894209	L
H-H1-0.0976	0	-10.171890	8.156810	-2.165367	L
H-H1-0.0976	0	-10.209227	7.725381	-3.892343	L
H-H1-0.0976	0	-10.489275	6.476617	-2.667746	L
C-CT-0.1200	-1	-8.865012	1.947329	3.250273	L
H-HC-0.0800	0	-8.128565	2.742679	3.355049	L
H-HC-0.0800	0	-9.667021	2.291257	2.597314	L
H-HC-0.0800	0	-9.281444	1.705885	4.228730	L
C-CT-0.2000	0	-8.234456	0.729469	2.656458	L
H-H1-0.0800	0	-9.002752	-0.033478	2.503145	L
O-OS--0.5600	0	-7.618425	1.076879	1.435663	L
C-CT-0.2000	0	-7.118747	0.189805	3.542978	L
H-H1-0.0800	0	-6.430179	0.984272	3.832631	L
O-OH--0.6800	0	-7.597672	-0.497002	4.693177	L
H-HO-0.4000	0	-8.274027	-1.125874	4.396499	L
C-CT-0.2000	0	-6.469958	-0.693601	2.488671	L
H-H1-0.0800	0	-5.446615	-0.959722	2.760119	L
O-OH--0.6800	0	-7.290919	-1.845751	2.342256	L
H-HO-0.4000	0	-6.777513	-2.513353	1.848212	L
C-CT-0.5691	0	-6.522703	0.178559	1.225106	H
H-H2-0.8000	0	-6.687161	-0.487303	0.355896	H
N-N*--0.5691	0	-5.312235	0.975943	1.033919	H
C-CM--0.0500	0	-4.701748	0.796304	-0.174298	H
H-H4-0.1500	0	-5.052515	-0.067451	-0.691364	H
C-CM--0.1238	0	-3.792303	1.606612	-0.712619	H
C-C-0.6156	0	-3.526873	1.577927	-2.167704	H
O-O--0.5700	0	-2.753061	2.343754	-2.683798	H
N-N--0.8000	0	-4.285087	0.745301	-2.942342	H
H-H-0.3700	0	-3.973172	0.538386	-3.869547	H
H-H-0.3700	0	-4.905320	0.073260	-2.543478	H
C-CT-0.1164	0	-3.156291	2.655670	0.167121	H
H-HC-0.0800	0	-2.812219	3.493213	-0.419592	H
H-HC-0.0800	0	-2.268141	2.259318	0.653284	H
C-CM--0.2882	0	-4.163126	3.060441	1.216812	H
H-HA-0.1500	0	-4.078817	4.036526	1.652177	H
C-CM--0.0500	0	-5.119136	2.242758	1.618082	H
H-H4-0.1500	0	-5.809941	2.522531	2.383644	H
N-N3--0.8530	0	3.292874	1.010445	-0.465538	H

H-H-0.4500	0	3.436754	0.763368	-1.431435	H
H-H-0.4500	0	2.049072	0.466090	0.830080	H
H-H-0.4500	0	4.139742	0.782996	0.034843	H
C-CT-0.3170	0	3.386619	2.466361	-0.383620	H
H-HP-0.0800	0	4.168113	2.836492	-1.041902	H
C-CT--0.1600	0	2.092694	3.206292	-0.718871	H
H-HC-0.0800	0	1.884079	3.071863	-1.774284	H
H-HC-0.0800	0	2.289014	4.262900	-0.579007	H
C-CT--0.1600	0	0.884387	2.785401	0.121995	H
H-HC-0.0800	0	0.131840	3.560444	0.073687	H
H-HC-0.0800	0	1.203297	2.736190	1.158308	H
C-CT--0.0990	0	0.262621	1.444472	-0.311121	H
H-HC-0.0800	0	0.894007	0.965846	-1.047521	H
H-HC-0.0800	0	-0.688543	1.629428	-0.787909	H
C-C-0.4490	0	0.087159	0.460653	0.836020	H
H-H-0.0600	0	-0.452801	0.943372	1.647805	H
O-O--0.5700	0	1.335406	0.067100	1.360687	H
N-N3--0.9900	0	-0.640368	-0.734363	0.411085	H
H-H-0.3600	0	0.703241	-1.598899	1.666056	H
H-H-0.3600	0	-0.966668	-0.593275	-0.533750	H
C-C-0.9060	0	3.888890	2.756382	1.035575	L
O-O2--0.9000	0	4.154621	1.821953	1.827583	L
O-O2--0.9000	0	4.046170	3.932513	1.421607	L
C-CT--0.205900	0	-1.663035	-1.296460	1.275259	H
H-H1-0.139900	0	-2.282529	-0.527344	1.733596	H
C-CT-0.007100	0	-2.565284	-2.199904	0.415410	H
H-HC--0.007800	0	-1.946765	-2.856588	-0.183282	H
H-HC--0.007800	0	-3.104217	-1.553576	-0.267640	H
C-CT-0.067500	0	-3.576848	-3.054078	1.180663	L
H-HC--0.054800	0	-3.855119	-2.527091	2.095629	L
H-HC--0.054800	0	-3.113836	-4.004128	1.456463	L
C-C-0.818300	0	-4.868921	-3.297194	0.395696	L
O-O2--0.822000	0	-5.695424	-2.352892	0.330845	L
O-O2--0.822000	0	-5.153726	-4.407409	-0.104561	L
C-C-0.742000	0	-0.987019	-2.079212	2.391365	H
O-O2--0.793000	0	-1.542343	-2.599440	3.309961	H
O-O2--0.793000	0	0.303653	-2.257366	2.249675	H
O-OW--0.834000	0	2.734375	-5.786051	-3.557869	L
H-HW-0.417000	0	3.590970	-5.910664	-3.100485	L
H-HW-0.417000	0	2.135975	-5.533269	-2.838136	L
O-OW--0.834000	0	0.188063	-7.397763	4.588625	L
H-HW-0.417000	0	0.864005	-7.059958	5.209608	L
H-HW-0.417000	0	-0.385447	-7.919733	5.156395	L
O-OW--0.834000	0	4.133835	-0.166166	3.393137	L
H-HW-0.417000	0	3.247062	-0.497341	3.219760	L
H-HW-0.417000	0	4.208037	0.579119	2.744353	L
O-OW--0.834000	0	6.212817	-1.611278	4.316827	L
H-HW-0.417000	0	5.863002	-2.511052	4.430740	L
H-HW-0.417000	0	5.437631	-1.119646	3.974939	L
O-OW--0.834000	0	-6.167012	-6.899412	0.041670	L
H-HW-0.417000	0	-5.828353	-5.982792	0.021381	L
H-HW-0.417000	0	-6.671646	-6.995977	-0.774060	L
O-OW--0.834000	0	-9.054501	-0.841210	-0.148069	L
H-HW-0.417000	0	-9.946673	-0.830829	0.208695	L
H-HW-0.417000	0	-8.847694	-1.802409	-0.182888	L

TS3

C-CT--0.3662	-1	-12.877445	1.028906	1.947285	L
H-HC-0.1123	0	-12.340037	1.935520	1.672653	L
H-HC-0.1123	0	-13.944718	1.190843	1.806101	L
H-HC-0.1123	0	-12.536224	0.202042	1.328072	L
C-C-0.5972	0	-12.605876	0.713066	3.405672	L
O-O--0.5679	0	-11.850276	1.434114	4.046962	L
N-N--0.415700	0	-13.197653	-0.372071	3.915370	L
H-H-0.271900	0	-13.793007	-0.896964	3.295800	L
C-CT--0.025200	-1	-12.904480	-0.937089	5.245904	L
H-H1-0.069800	0	-13.647048	-1.698058	5.485180	L
H-H1-0.069800	0	-12.957412	-0.144422	5.992898	L
C-C-0.597300	0	-11.487417	-1.582256	5.311444	L
O-O--0.567900	0	-10.665418	-1.429496	4.402250	L
N-N--0.415700	0	-11.184747	-2.336831	6.379372	L
H-H-0.271900	0	-11.896191	-2.457385	7.082751	L
C-CT--0.025200	-1	-9.990679	-3.208901	6.454063	L
H-H1-0.069800	0	-9.902941	-3.625414	7.457174	L
H-H1-0.069800	0	-9.095970	-2.626269	6.231018	L
C-C-0.597300	0	-10.123946	-4.358101	5.463298	L
O-O--0.567900	0	-11.167121	-5.003134	5.390991	L
N-N--0.4157	0	-9.064769	-4.607870	4.703666	L
H-H-0.2719	0	-8.229193	-4.069185	4.859499	L
C-CT--0.0014	-1	-9.007658	-5.549352	3.586241	L
H-H1-0.0876	0	-9.907809	-5.450019	2.978772	L
C-C-0.5973	0	-8.904298	-7.028059	4.020022	L
O-O--0.5679	0	-8.166878	-7.820152	3.439661	L
C-CT--0.0152	0	-7.799446	-5.135146	2.732840	L
H-HC-0.0295	0	-8.026267	-4.147546	2.353425	L
H-HC-0.0295	0	-7.744878	-5.790157	1.863176	L
C-CA--0.0011	0	-6.422400	-5.074016	3.400618	L
C-CA--0.1906	0	-5.498663	-6.120634	3.208890	L
H-HA-0.1699	0	-5.801576	-7.010224	2.669566	L
C-CA--0.1906	0	-6.012920	-3.925517	4.113208	L
H-HA-0.1699	0	-6.696424	-3.112369	4.287018	L
C-CA--0.2341	0	-4.170549	-5.999842	3.665300	L
H-HA-0.1656	0	-3.456854	-6.786540	3.480101	L
C-CA--0.2341	0	-4.681790	-3.788566	4.550146	L
H-HA-0.1656	0	-4.347844	-2.887072	5.037095	L
C-C-0.3326	0	-3.750429	-4.818308	4.308839	L
O-OH--0.5579	0	-2.448075	-4.647296	4.653221	L
H-HO-0.3992	0	-2.114969	-3.828017	4.220843	L
N-N--0.4157	0	-9.654480	-7.416989	5.052010	L
H-H-0.2719	0	-10.308392	-6.733407	5.414708	L
C-CT--0.1490	-1	-9.578311	-8.740936	5.644387	L
H-H1-0.0976	0	-8.561031	-8.931667	5.991656	L
H-H1-0.0976	0	-10.269514	-8.818388	6.484338	L
H-H1-0.0976	0	-9.835785	-9.492644	4.895358	L
C-CT--0.3662	-1	-12.014242	-1.456414	-3.467656	L
H-HC-0.1123	0	-12.525522	-0.544249	-3.771457	L
H-HC-0.1123	0	-12.025952	-1.559428	-2.384883	L
H-HC-0.1123	0	-12.514159	-2.313242	-3.918311	L
C-C-0.5972	0	-10.581131	-1.419259	-3.961244	L
O-O--0.5679	0	-10.126508	-2.283253	-4.698392	L

N-N--0.415700	0	-9.852303	-0.415097	-3.498801	L
H-H-0.271900	0	-10.340716	0.250264	-2.915668	L
C-CT--0.038900	-1	-8.416981	-0.105792	-3.677589	L
H-H1-0.100700	0	-8.143902	-0.087892	-4.730456	L
C-C-0.597300	0	-7.522835	-1.088830	-2.919033	L
O-O--0.567900	0	-6.672417	-0.692568	-2.119055	L
C-CT-0.365400	0	-8.247289	1.300908	-3.057194	L
H-H1-0.004300	0	-7.192676	1.567022	-3.046217	L
O-OH--0.676100	0	-8.766767	1.323952	-1.735082	L
H-HO-0.410200	0	-8.581197	0.475904	-1.306545	L
C-CT--0.243800	0	-9.038447	2.395821	-3.770904	L
H-HC-0.064200	0	-10.082917	2.121597	-3.905704	L
H-HC-0.064200	0	-8.599813	2.608784	-4.737857	L
H-HC-0.064200	0	-9.008465	3.306996	-3.179026	L
N-N--0.415700	0	-7.763146	-2.380828	-3.097372	L
H-H-0.271900	0	-8.457734	-2.640127	-3.793639	L
C-CT--0.024900	-1	-7.207129	-3.455715	-2.280421	L
H-H1-0.084300	0	-6.360997	-3.082685	-1.722910	L
C-C-0.597300	0	-6.675260	-4.588040	-3.160941	L
O-O--0.567900	0	-7.138311	-4.763039	-4.285886	L
C-CT-0.211700	0	-8.273816	-4.055227	-1.361610	L
H-H1-0.035200	0	-8.192987	-5.143845	-1.336376	L
H-H1-0.035200	0	-9.275155	-3.784065	-1.700174	L
O-OH--0.654600	0	-8.038554	-3.564500	-0.071473	L
H-HO-0.427500	0	-7.111033	-3.828639	0.128922	L
N-N--0.4157	0	-5.729106	-5.387930	-2.658985	L
H-H-0.2719	0	-5.388430	-5.203173	-1.718475	L
C-CT--0.0014	0	-5.334992	-6.620975	-3.354095	L
H-H1-0.0876	0	-5.034565	-6.359363	-4.368962	L
C-C-0.5973	0	-6.482192	-7.630508	-3.432796	L
O-O--0.5679	0	-7.371592	-7.650640	-2.580092	L
C-CT--0.0152	0	-4.170690	-7.341909	-2.668999	L
H-HC-0.0295	0	-3.886564	-8.198393	-3.281796	L
H-HC-0.0295	0	-4.502195	-7.752772	-1.717772	L
C-CA--0.0011	-1	-2.931722	-6.527149	-2.439982	L
C-CA--0.1906	0	-2.006769	-6.335642	-3.487299	L
H-HA-0.1699	0	-2.225659	-6.714376	-4.475257	L
C-CA--0.1906	0	-2.630259	-6.060808	-1.146290	L
H-HA-0.1699	0	-3.331242	-6.220372	-0.334143	L
C-CA--0.2341	0	-0.789580	-5.668101	-3.244374	L
H-HA-0.1656	0	-0.077045	-5.510166	-4.038848	L
C-CA--0.2341	0	-1.408576	-5.408923	-0.897685	L
H-HA-0.1656	0	-1.185555	-5.062913	0.100208	L
C-C-0.3226	0	-0.490243	-5.200738	-1.947400	L
O-OH--0.5579	0	0.678011	-4.547722	-1.709892	L
H-HO-0.3992	0	0.543632	-3.966854	-0.950131	L
N-N--0.4157	0	-6.370524	-8.535433	-4.409293	L
H-H-0.2719	0	-5.615415	-8.412041	-5.063281	L
C-CT--0.1490	-1	-7.157107	-9.751078	-4.488006	L
H-H1-0.0976	0	-8.215841	-9.496254	-4.572391	L
H-H1-0.0976	0	-6.860061	-10.341592	-5.355561	L
H-H1-0.0976	0	-7.015617	-10.342522	-3.580924	L
C-CT--0.3662	-1	-6.347437	-3.497277	-6.966765	L
H-HC-0.1123	0	-5.862130	-4.252694	-7.581216	L
H-HC-0.1123	0	-7.388258	-3.776297	-6.807195	L

H-HC-0.1123 0 -5.836761 -3.418024 -6.012858 L
C-C-0.5972 0 -6.270730 -2.154418 -7.676119 L
O-O--0.5679 0 -5.382668 -1.980640 -8.499026 L
N-N--0.415700 0 -7.171738 -1.213049 -7.359034 L
H-H-0.271900 0 -7.886189 -1.487339 -6.701984 L
C-CT--0.025200 -1 -7.300886 0.112932 -8.013884 L
H-H1-0.069800 0 -8.241256 0.137372 -8.564807 L
H-H1-0.069800 0 -6.500271 0.258300 -8.738380 L
C-C-0.597300 0 -7.294673 1.298093 -7.051640 L
O-O--0.567900 0 -8.219454 2.098457 -7.089225 L
N-N--0.415700 0 -6.262360 1.404540 -6.208719 L
H-H-0.271900 0 -5.555558 0.679021 -6.260792 L
C-CT--0.025200 -1 -5.836921 2.643348 -5.545165 L
H-H1-0.069800 0 -5.665654 2.460540 -4.487599 L
H-H1-0.069800 0 -6.600865 3.414661 -5.653679 L
C-C-0.597300 0 -4.550245 3.161329 -6.196857 L
O-O--0.567900 0 -4.489652 3.295047 -7.416451 L
N-N--0.415700 0 -3.505263 3.389902 -5.398455 L
H-H-0.271900 0 -3.620372 3.238282 -4.406257 L
C-CT--0.051800 -1 -2.169640 3.778377 -5.867257 L
H-H1-0.092200 0 -2.260055 4.227738 -6.857617 L
C-C-0.597300 0 -1.249350 2.547162 -5.997231 L
O-O--0.567900 0 -0.571927 2.340407 -7.001040 L
C-CT--0.110200 0 -1.619315 4.840550 -4.894726 L
H-HC-0.045700 0 -1.583117 4.415736 -3.892070 L
H-HC-0.045700 0 -2.316290 5.679621 -4.872412 L
C-CT-0.353100 0 -0.219261 5.376197 -5.237142 L
H-HC--0.036100 0 0.494892 4.552731 -5.248060 L
C-CT--0.412100 0 -0.176491 6.090283 -6.588070 L
H-HC-0.100000 0 -0.928890 6.879403 -6.617253 L
H-HC-0.100000 0 -0.375415 5.378453 -7.389178 L
H-HC-0.100000 0 0.810329 6.522097 -6.750464 L
C-CT--0.412100 0 0.211542 6.369880 -4.155939 L
H-HC-0.100000 0 -0.492583 7.201889 -4.115806 L
H-HC-0.100000 0 1.206721 6.752428 -4.380835 L
H-HC-0.100000 0 0.234441 5.869683 -3.188747 L
N-N--0.516300 0 -1.199506 1.738003 -4.943182 L
H-H-0.293600 0 -1.763597 2.026708 -4.150502 L
C-CT-0.038100 -1 -0.312642 0.598152 -4.725338 L
H-H1-0.088000 0 -0.079596 0.103064 -5.665756 L
C-C-0.536600 0 -1.002248 -0.381614 -3.749628 L
O-O--0.581900 0 -0.887533 -0.210237 -2.544055 L
C-CT--0.030300 0 0.998514 1.189153 -4.125409 L
H-HC--0.012200 0 0.739636 1.820608 -3.276259 L
H-HC--0.012200 0 1.460220 1.833928 -4.875204 L
C-C-0.799400 -1 2.053073 0.166858 -3.676744 L
O-O2--0.801400 0 2.016458 -0.965497 -4.210390 L
O-O2--0.801400 0 2.974685 0.497398 -2.900317 L
N-N--0.254800 0 -1.773774 -1.376627 -4.215602 L
C-CT--0.026600 -1 -2.061207 -1.674132 -5.610542 L
H-H1-0.064100 0 -1.135759 -1.736021 -6.181881 L
C-C-0.589600 0 -2.997599 -0.644759 -6.269463 L
O-O--0.574800 0 -3.912727 -0.128917 -5.625785 L
C-CT--0.007000 0 -2.714820 -3.059825 -5.579332 L
H-HC-0.025300 0 -1.939137 -3.827108 -5.572419 L

H-HC-0.025300 0 -3.393431 -3.217655 -6.418201 L
C-CT-0.018900 0 -3.447844 -3.069581 -4.238723 L
H-HC-0.021300 0 -3.609350 -4.080188 -3.872143 L
H-HC-0.021300 0 -4.394655 -2.534625 -4.322854 L
C-CT-0.019200 0 -2.499000 -2.286497 -3.338363 L
H-H1-0.039100 0 -3.063941 -1.740066 -2.583203 L
H-H1-0.039100 0 -1.793594 -2.962380 -2.856414 L
N-N--0.4157 0 -2.770706 -0.375670 -7.559549 L
H-H-0.2719 0 -2.005775 -0.851486 -8.005774 L
C-CT--0.1490 -1 -3.555172 0.524013 -8.404785 L
H-H1-0.0976 0 -4.513748 0.744037 -7.948807 L
H-H1-0.0976 0 -3.736392 0.061592 -9.376038 L
H-H1-0.0976 0 -3.016539 1.463505 -8.548014 L
C-CT--0.3662 -1 8.735291 1.913575 -5.924149 L
H-HC-0.1123 0 9.227730 2.162860 -6.863732 L
H-HC-0.1123 0 9.488600 1.700690 -5.168518 L
H-HC-0.1123 0 8.088974 1.050844 -6.072336 L
C-C-0.5972 0 7.906513 3.098686 -5.480883 L
O-O--0.5679 0 8.029774 4.173910 -6.048475 L
N-N--0.415700 0 7.062421 2.910093 -4.466448 L
H-H-0.271900 0 7.010274 1.983775 -4.066934 L
C-CT-0.021300 -1 6.069042 3.901843 -4.030203 L
H-H1-0.112400 0 6.254424 4.854883 -4.530164 L
C-C-0.597300 0 6.106614 4.141812 -2.518254 L
O-O--0.567900 0 6.362556 3.222422 -1.739526 L
C-CT--0.123100 0 4.693453 3.378856 -4.479315 L
H-H1-0.111200 0 4.488012 2.418269 -3.999222 L
H-H1-0.111200 0 4.701935 3.234362 -5.561430 L
S-SH--0.311900 0 3.377940 4.554556 -4.052961 L
H-HS-0.193300 0 2.369979 3.901665 -4.649852 L
N-N--0.415700 0 5.830498 5.375109 -2.096191 L
H-H-0.271900 0 5.581353 6.068893 -2.788173 L
C-CT--0.025200 -1 5.815183 5.780181 -0.695806 L
H-H1-0.069800 0 6.778771 6.226096 -0.450025 L
H-H1-0.069800 0 5.681344 4.915109 -0.050815 L
C-C-0.597300 0 4.735068 6.814456 -0.404716 L
O-O--0.567900 0 4.840353 7.961792 -0.828938 L
N-N--0.415700 0 3.723845 6.431864 0.370561 L
H-H-0.271900 0 3.766774 5.484518 0.740731 L
C-CT--0.025200 -1 2.685211 7.309859 0.899693 L
H-H1-0.069800 0 1.851518 6.696644 1.227268 L
H-H1-0.069800 0 2.346831 8.006683 0.132187 L
C-C-0.597300 0 3.183757 8.094102 2.111550 L
O-O--0.567900 0 2.606940 7.987164 3.191654 L
N-N--0.4157 0 4.271258 8.849493 1.924577 L
H-H-0.2719 0 4.629397 8.864056 0.974544 L
C-CT--0.1490 -1 5.016562 9.533150 2.972586 L
H-H1-0.0976 0 5.350720 8.811065 3.719535 L
H-H1-0.0976 0 5.882417 10.041532 2.545751 L
H-H1-0.0976 0 4.373330 10.268474 3.459946 L
C-CT--0.3662 -1 -1.506876 9.430153 5.297948 L
H-HC-0.1123 0 -1.719906 10.395994 4.842643 L
H-HC-0.1123 0 -0.568645 9.040773 4.906244 L
H-HC-0.1123 0 -1.442100 9.547308 6.378204 L
C-C-0.5972 0 -2.642163 8.459380 4.961227 L

O-O--0.5679	0	-3.530040	8.816075	4.197237 L
N-N--0.4157	0	-2.612797	7.232315	5.497740 L
H-H-0.2719	0	-1.862465	7.017354	6.139937 L
C-CT--0.0275	-1	-3.603049	6.138959	5.315917 L
H-H1-0.1123	0	-4.601822	6.572065	5.253367 L
C-C-0.5973	0	-3.567728	5.164124	6.522547 L
O-O--0.5679	0	-2.649311	5.222393	7.338917 L
C-CT--0.0050	0	-3.309065	5.363180	4.021820 L
H-HC-0.0339	0	-3.498257	6.019755	3.179824 L
H-HC-0.0339	0	-4.038289	4.557917	3.939514 L
C-C*--0.1415	0	-1.936051	4.761416	3.872812 L
C-CW--0.1638	0	-0.781838	5.445479	3.681336 L
H-H4-0.2062	0	-0.699143	6.522569	3.616042 L
C-CB-0.1243	0	-1.554498	3.350144	3.905626 L
N-NA--0.3418	0	0.279949	4.565221	3.599765 L
H-H-0.3412	0	1.244854	4.850814	3.487887 L
C-CN-0.1380	0	-0.138459	3.261141	3.741511 L
C-CA--0.238	0	-2.258868	2.135613	4.067808 L
H-HA-0.1700	0	-3.331664	2.159356	4.170332 L
C-CA--0.2601	0	0.546426	2.038243	3.758471 L
H-HA-0.1572	0	1.618126	2.004582	3.627822 L
C-CA--0.1972	0	-1.582482	0.900677	4.079010 L
H-HA-0.1447	0	-2.127965	-0.029126	4.181211 L
C-CA--0.1134	0	-0.186264	0.853516	3.931689 L
H-HA-0.1417	0	0.317149	-0.102375	3.925347 L
N-N--0.4157	0	-4.551998	4.253921	6.635898 L
H-H-0.2719	0	-5.271574	4.284316	5.932343 L
C-CT--0.1490	-1	-4.644663	3.225940	7.687690 L
H-H1-0.0976	0	-3.820068	2.519299	7.581444 L
H-H1-0.0976	0	-4.574269	3.699082	8.669697 L
H-H1-0.0976	0	-5.592918	2.691400	7.614369 L
C-CT--0.3662	-1	-2.798767	-1.492979	7.275829 L
H-HC-0.1123	0	-3.025921	-2.548355	7.124183 L
H-HC-0.1123	0	-3.149336	-1.184514	8.258295 L
H-HC-0.1123	0	-3.282782	-0.908872	6.495371 L
C-C-0.5972	0	-1.306862	-1.301832	7.179360 L
O-O--0.5679	0	-0.592674	-2.253222	6.913540 L
N-N--0.415700	0	-0.810088	-0.098076	7.461019 L
H-H-0.271900	0	-1.467939	0.653181	7.596939 L
C-CT--0.087500	-1	0.632563	0.230621	7.417365 L
H-H1-0.096900	0	0.959244	0.125593	6.382385 L
C-C-0.597300	0	1.479001	-0.734195	8.259940 L
O-O--0.567900	0	2.472391	-1.268207	7.779117 L
C-CT-0.298500	0	0.913897	1.685134	7.864437 L
H-HC--0.029700	0	0.963364	1.719311	8.953426 L
C-CT--0.319200	0	2.262896	2.154702	7.306268 L
H-HC-0.079100	0	2.231778	2.164744	6.216338 L
H-HC-0.079100	0	3.059235	1.486884	7.635662 L
H-HC-0.079100	0	2.482147	3.160197	7.666154 L
C-CT--0.319200	0	-0.163006	2.689518	7.432015 L
H-HC-0.079100	0	-0.347937	2.602279	6.365189 L
H-HC-0.079100	0	0.160589	3.707646	7.651116 L
H-HC-0.079100	0	-1.091397	2.516666	7.974321 L
N-N--0.415700	0	1.062816	-1.001677	9.505028 L
H-H-0.271900	0	0.238160	-0.518731	9.822708 L

C-CT--0.025200	-1	1.676095	-2.005332	10.390837	L
H-H1-0.069800	0	1.146572	-2.025422	11.342770	L
H-H1-0.069800	0	2.712411	-1.725314	10.583531	L
C-C-0.597300	0	1.675938	-3.440033	9.811786	L
O-O--0.567900	0	2.470109	-4.262562	10.251058	L
N-N--0.415700	0	0.799920	-3.747673	8.843590	L
H-H-0.271900	0	0.215100	-3.005921	8.485018	L
C-CT-0.033700	-1	0.667793	-5.040277	8.158228	L
H-H1-0.082300	0	1.125687	-5.808068	8.784839	L
C-C-0.597300	0	1.393297	-5.107342	6.789002	L
O-O--0.567900	0	1.832936	-6.179567	6.372841	L
C-CT--0.182500	0	-0.821048	-5.388202	8.025368	L
H-HC-0.060300	0	-1.299127	-5.357787	9.004887	L
H-HC-0.060300	0	-0.924051	-6.393204	7.615200	L
H-HC-0.060300	0	-1.319771	-4.687725	7.355656	L
N-N--0.415700	0	1.522684	-3.981055	6.083175	L
H-H-0.271900	0	0.991007	-3.185930	6.418391	L
C-CT-0.033700	-1	2.459728	-3.728310	4.975328	L
H-H1-0.082300	0	2.313359	-4.451743	4.177601	L
C-C-0.597300	0	3.928477	-3.795388	5.449818	L
O-O--0.567900	0	4.830215	-4.092568	4.665192	L
C-CT--0.182500	0	2.162391	-2.316362	4.490712	L
H-HC-0.060300	0	2.605976	-1.581890	5.162387	L
H-HC-0.060300	0	1.086231	-2.156447	4.486678	L
H-HC-0.060300	0	2.570588	-2.196023	3.488691	L
N-N--0.4157	0	4.170609	-3.443214	6.720607	L
H-H-0.2719	0	3.378119	-3.071128	7.229866	L
C-CT--0.1490	-1	5.449365	-3.499784	7.415118	L
H-H1-0.0976	0	5.973411	-4.420637	7.151567	L
H-H1-0.0976	0	5.284683	-3.480045	8.493788	L
H-H1-0.0976	0	6.064362	-2.645899	7.126217	L
C-CT--0.3662	-1	14.081815	0.929313	-0.709006	L
H-HC-0.1123	0	13.444777	1.439023	0.010973	L
H-HC-0.1123	0	15.089339	1.340466	-0.666603	L
H-HC-0.1123	0	13.679359	1.047798	-1.712875	L
C-C-0.5972	0	14.139554	-0.551013	-0.369927	L
O-O--0.5679	0	15.221166	-1.111997	-0.260516	L
N-N--0.4157	0	12.979128	-1.189361	-0.222293	L
H-H-0.2719	0	12.136506	-0.639555	-0.342162	L
C-CT--0.0014	-1	12.803658	-2.643482	-0.024043	L
H-H1-0.0876	0	13.684861	-3.160154	-0.406994	L
C-C-0.5973	0	11.577571	-3.134806	-0.846262	L
O-O--0.5679	0	10.817485	-2.332210	-1.397558	L
C-CT--0.0152	0	12.669327	-2.965102	1.474400	L
H-HC-0.0295	0	13.615159	-2.721145	1.960683	L
H-HC-0.0295	0	12.520720	-4.038540	1.596621	L
C-CA--0.0011	-1	11.551478	-2.230316	2.194532	L
C-CA--0.1906	0	11.821835	-1.003334	2.829629	L
H-HA-0.1699	0	12.824552	-0.598453	2.808994	L
C-CA--0.1906	0	10.246533	-2.760696	2.224283	L
H-HA-0.1699	0	10.030810	-3.708279	1.750869	L
C-CA--0.2341	0	10.794194	-0.310161	3.495150	L
H-HA-0.1656	0	11.006829	0.615862	3.998313	L
C-CA--0.2341	0	9.214222	-2.065255	2.883495	L
H-HA-0.1656	0	8.217594	-2.479446	2.908236	L

C-C-0.3226 0 9.485611 -0.837308 3.525795 L
O-OH--0.557 0 8.498122 -0.164835 4.176407 L
H-HO-0.3992 0 7.701531 -0.731440 4.239374 L
N-N--0.415700 0 11.366909 -4.452803 -0.965597 L
H-H-0.271900 0 11.988116 -5.073132 -0.470076 L
C-CT--0.025200 -1 10.243854 -5.048768 -1.718707 L
H-H1-0.069800 0 10.459311 -6.100313 -1.907241 L
H-H1-0.069800 0 10.138151 -4.549756 -2.681240 L
C-C-0.597300 0 8.918973 -4.974863 -0.947127 L
O-O--0.567900 0 8.855655 -5.375455 0.213730 L
N-N--0.415700 0 7.847062 -4.487535 -1.578642 L
H-H-0.271900 0 7.927346 -4.354773 -2.579580 L
C-CT-0.014300 -1 6.511265 -4.462244 -0.984015 L
H-H1-0.104800 0 6.609822 -4.367655 0.100352 L
C-C-0.597300 0 5.767626 -5.769861 -1.262245 L
O-O--0.567900 0 5.039780 -5.893490 -2.252870 L
C-CT--0.204100 0 5.697577 -3.235896 -1.457891 L
H-HC-0.079700 0 5.560798 -3.286292 -2.537819 L
H-HC-0.079700 0 6.273075 -2.350870 -1.224214 L
C-C-0.713000 -1 4.315068 -3.102356 -0.796104 L
O-O--0.593100 0 3.934203 -3.839416 0.101424 L
N-N--0.919100 0 3.484350 -2.197081 -1.270412 L
H-H-0.419600 0 2.582553 -2.150512 -0.823289 L
H-H-0.419600 0 3.681146 -1.605516 -2.068648 L
N-N--0.347900 0 5.923011 -6.718799 -0.336551 L
H-H-0.274700 0 6.597881 -6.498038 0.385785 L
C-CT--0.263700 -1 5.367877 -8.076702 -0.399815 L
H-H1-0.156000 0 5.746793 -8.619285 0.466452 L
C-C-0.734100 0 5.871512 -8.869134 -1.616693 L
O-O--0.589400 0 6.874990 -8.523187 -2.233526 L
C-CT--0.000700 0 3.825710 -8.045153 -0.294035 L
H-HC-0.032700 0 3.448145 -9.067230 -0.246995 L
H-HC-0.032700 0 3.415335 -7.582182 -1.192675 L
C-CT-0.039000 0 3.281866 -7.313771 0.938183 L
H-HC-0.028500 0 3.643084 -6.287293 0.963858 L
H-HC-0.028500 0 3.598076 -7.829665 1.845904 L
C-CT-0.048600 0 1.754364 -7.314570 0.842606 L
H-H1-0.068700 0 1.397747 -8.345916 0.818119 L
H-H1-0.068700 0 1.448068 -6.829149 -0.086858 L
N-N2--0.529500 -1 1.128095 -6.629960 1.976579 L
H-H-0.345600 0 0.740235 -7.181656 2.736204 L
C-CA-0.807600 0 0.870108 -5.350099 2.086588 L
N-N2--0.862700 0 0.054424 -4.968084 3.011720 L
H-H-0.447800 0 -0.349745 -5.639001 3.649393 L
H-H-0.447800 0 -0.225430 -3.985267 3.075553 L
N-N2--0.862700 0 1.393365 -4.446079 1.313882 L
H-H-0.447800 0 2.244860 -4.613229 0.795495 L
H-H-0.447800 0 1.123626 -3.489828 1.550805 L
N-N--0.4157 0 5.190560 -9.977488 -1.915247 L
H-H-0.2719 0 4.398999 -10.186715 -1.331318 L
C-CT--0.1490 -1 5.490821 -10.868208 -3.021351 L
H-H1-0.0976 0 6.503207 -11.263408 -2.912209 L
H-H1-0.0976 0 4.782764 -11.698045 -3.043856 L
H-H1-0.0976 0 5.432757 -10.319140 -3.963316 L
C-CT--0.3662 -1 10.136773 -5.921773 -7.793845 L

H-HC-0.1123	0	9.850178	-6.625249	-8.573067	L
H-HC-0.1123	0	11.036792	-5.390564	-8.102163	L
H-HC-0.1123	0	10.331774	-6.446971	-6.860372	L
C-C-0.5972	0	9.023020	-4.916632	-7.582602	L
O-O--0.5679	0	9.248021	-3.720370	-7.729833	L
N-N--0.347900	0	7.827907	-5.370530	-7.174267	L
H-H-0.274700	0	7.748890	-6.366057	-7.031187	L
C-CT--0.263700	-1	6.854608	-4.470756	-6.520475	L
H-H1-0.156000	0	6.669418	-3.611365	-7.165841	L
C-C-0.734100	0	7.510404	-3.971111	-5.230464	L
O-O--0.589400	0	7.886498	-4.776570	-4.383853	L
C-CT--0.000700	0	5.522544	-5.166812	-6.192881	L
H-HC-0.032700	0	4.931362	-4.464408	-5.606002	L
H-HC-0.032700	0	5.700739	-6.043053	-5.565697	L
C-CT-0.039000	0	4.722818	-5.588940	-7.437078	L
H-HC-0.028500	0	4.974412	-6.623444	-7.674221	L
H-HC-0.028500	0	5.001852	-4.970232	-8.291601	L
C-CT-0.048600	0	3.203440	-5.476026	-7.219114	L
H-H1-0.068700	0	2.941140	-5.871535	-6.235472	L
H-H1-0.068700	0	2.704384	-6.095042	-7.967069	L
N-N2--0.529500	0	2.735321	-4.082256	-7.383719	L
H-H-0.345600	0	2.674250	-3.734773	-8.324307	L
C-CA-0.807600	0	2.439921	-3.200369	-6.443260	L
N-N2--0.862700	0	2.410942	-3.468438	-5.176342	L
H-H-0.447800	0	2.570425	-4.395913	-4.795000	L
H-H-0.447800	0	2.215864	-2.686359	-4.545829	L
N-N2--0.862700	0	2.169724	-1.967606	-6.747244	L
H-H-0.447800	0	2.178280	-1.590943	-7.673818	L
H-H-0.447800	0	2.047440	-1.368814	-5.926267	L
N-N--0.415700	0	7.704229	-2.663646	-5.094212	L
H-H-0.271900	0	7.381152	-2.063277	-5.835025	L
C-CT--0.025200	-1	8.605682	-2.092396	-4.090989	L
H-H1-0.069800	0	9.484941	-1.703423	-4.605296	L
H-H1-0.069800	0	8.953125	-2.854506	-3.398270	L
C-C-0.597300	0	8.009064	-0.957951	-3.277276	L
O-O--0.567900	0	6.947288	-0.422201	-3.598666	L
N-N--0.415700	0	8.705109	-0.568546	-2.213086	L
H-H-0.271900	0	9.563682	-1.059948	-1.967450	L
C-CT--0.038900	-1	8.299140	0.558659	-1.379381	L
H-H1-0.100700	0	7.652476	1.185679	-1.989217	L
C-C-0.597300	0	9.438389	1.488170	-0.988124	L
O-O--0.567900	0	10.552416	1.072785	-0.673375	L
C-CT-0.365400	0	7.435274	0.099996	-0.187568	L
H-H1-0.004300	0	7.759424	-0.890690	0.134616	L
O-OH--0.676100	0	6.101598	0.040196	-0.656626	L
H-HO-0.410200	0	6.145564	-0.149790	-1.605266	L
C-CT--0.243800	0	7.397809	1.019957	1.032709	L
H-HC-0.064200	0	7.034710	2.008653	0.753156	L
H-HC-0.064200	0	8.390980	1.097732	1.473774	L
H-HC-0.064200	0	6.727067	0.595381	1.780185	L
N-N--0.415700	0	9.110836	2.779849	-1.036324	L
H-H-0.271900	0	8.140324	2.986737	-1.250557	L
C-CT--0.025200	-1	9.952985	3.904659	-0.676550	L
H-H1-0.069800	0	9.760642	4.728723	-1.363454	L
H-H1-0.069800	0	11.003198	3.620626	-0.762947	L

C-C-0.597300	0	9.655990	4.380376	0.732709 L
O-O--0.567900	0	8.507393	4.576132	1.142589 L
N-N--0.347900	0	10.731619	4.597876	1.473177 L
H-H-0.274700	0	11.637285	4.454190	1.040924 L
C-CT--0.263700	-1	10.743405	5.177969	2.806661 L
H-H1-0.156000	0	9.837367	5.759170	2.968152 L
C-C-0.734100	0	11.914520	6.131855	2.912728 L
O-O--0.589400	0	12.861110	6.054144	2.134824 L
C-CT--0.000700	0	10.899020	4.059987	3.861239 L
H-HC-0.032700	0	11.185702	4.494846	4.820416 L
H-HC-0.032700	0	11.707353	3.392999	3.553894 L
C-CT-0.039000	0	9.633948	3.226416	4.081478 L
H-HC-0.028500	0	9.875095	2.388450	4.734417 L
H-HC-0.028500	0	9.274142	2.831389	3.131283 L
C-CT-0.048600	0	8.544078	4.053621	4.764089 L
H-H1-0.068700	0	8.365237	4.977370	4.214775 L
H-H1-0.068700	0	8.875301	4.316580	5.770427 L
N-N2--0.529500	-1	7.297481	3.291494	4.851305 L
H-H-0.345600	0	7.190802	2.648778	5.617268 L
C-CA-0.807600	0	6.290373	3.332834	4.007803 L
N-N2--0.862700	0	5.215960	2.655639	4.252730 L
H-H-0.447800	0	5.181411	1.945938	4.958695 L
H-H-0.447800	0	4.627155	2.533539	3.426462 L
N-N2--0.862700	0	6.318661	4.024944	2.909799 L
H-H-0.447800	0	7.184312	4.353331	2.505526 L
H-H-0.447800	0	5.469847	4.011115	2.342520 L
N-N--0.4157	0	11.834590	7.008902	3.903991 L
H-H-0.2719	0	11.019890	6.992367	4.493654 L
C-CT--0.1490	-1	12.898894	7.935123	4.195068 L
H-H1-0.0976	0	12.636705	8.560320	5.050139 L
H-H1-0.0976	0	13.817825	7.388996	4.421021 L
H-H1-0.0976	0	13.083193	8.575257	3.329031 L
C-CT--0.3662	-1	-2.175046	13.253260	-1.513545 L
H-HC-0.1123	0	-2.590124	13.761597	-0.645745 L
H-HC-0.1123	0	-1.087998	13.237406	-1.466427 L
H-HC-0.1123	0	-2.485631	13.776098	-2.417890 L
C-C-0.5972	0	-2.701479	11.830958	-1.572433 L
O-O--0.5679	0	-3.359654	11.461919	-2.532260 L
N-N--0.415700	0	-2.418615	11.041697	-0.532626 L
H-H-0.271900	0	-1.853172	11.457292	0.188343 L
C-CT--0.023700	-1	-2.840247	9.634420	-0.364640 L
H-H1-0.088000	0	-2.375074	9.044436	-1.156005 L
C-C-0.597300	0	-4.360124	9.450647	-0.521666 L
O-O--0.567900	0	-4.823897	8.669698	-1.352661 L
C-CT-0.034200	0	-2.293392	9.150459	0.984841 L
H-HC-0.024100	0	-2.659979	9.802793	1.778559 L
H-HC-0.024100	0	-1.205326	9.222076	0.968749 L
C-CT-0.001800	0	-2.679640	7.715789	1.329760 L
H-H1-0.044000	0	-3.750725	7.692364	1.533836 L
H-H1-0.044000	0	-2.161440	7.453761	2.248239 L
S-S--0.273700	0	-2.324450	6.442598	0.094217 L
C-CT--0.053600	0	-0.523121	6.425188	0.056865 L
H-H1-0.068400	0	-0.187233	5.717478	-0.701152 L
H-H1-0.068400	0	-0.156137	6.110305	1.031730 L
H-H1-0.068400	0	-0.147014	7.417714	-0.188730 L

N-N--0.4157	0	-5.142776	10.184158	0.274245	L
H-H-0.2719	0	-4.685556	10.796947	0.929855	L
C-CT--0.1490	-1	-6.593630	10.203830	0.174157	L
H-H1-0.0976	0	-7.014525	10.862019	0.935515	L
H-H1-0.0976	0	-6.887479	10.564071	-0.814085	L
H-H1-0.0976	0	-6.986623	9.195084	0.313560	L
C-CT--0.3662	-1	-4.458243	8.995343	-5.114701	L
H-HC-0.1123	0	-4.026128	9.345424	-4.178020	L
H-HC-0.1123	0	-3.695555	8.999214	-5.891343	L
H-HC-0.1123	0	-5.281860	9.640920	-5.410873	L
C-C-0.5972	0	-4.974805	7.574430	-4.940188	L
O-O--0.5679	0	-4.684634	6.716559	-5.761780	L
N-N--0.415700	0	-5.749168	7.329221	-3.879428	L
H-H-0.271900	0	-5.771367	8.073954	-3.194511	L
C-CT--0.087500	-1	-6.405717	6.035103	-3.551077	L
H-H1-0.096900	0	-6.121813	5.292151	-4.295742	L
C-C-0.597300	0	-7.921264	6.185481	-3.637437	L
O-O--0.567900	0	-8.566152	5.479561	-4.402748	L
C-CT-0.298500	0	-5.940301	5.538938	-2.173386	L
H-HC--0.029700	0	-6.142980	6.301008	-1.420379	L
C-CT--0.319200	0	-6.613442	4.239066	-1.730054	L
H-HC-0.079100	0	-6.418147	3.465983	-2.467146	L
H-HC-0.079100	0	-7.687043	4.384603	-1.620784	L
H-HC-0.079100	0	-6.214765	3.919598	-0.767532	L
C-CT--0.319200	0	-4.439996	5.269150	-2.222852	L
H-HC-0.079100	0	-4.211886	4.548823	-3.003747	L
H-HC-0.079100	0	-4.131028	4.876264	-1.264076	L
H-HC-0.079100	0	-3.890996	6.190569	-2.414535	L
N-N--0.4157	0	-8.499631	7.111896	-2.865068	L
H-H-0.2719	0	-7.886345	7.662004	-2.284858	L
C-CT--0.1490	-1	-9.931401	7.393284	-2.887275	L
H-H1-0.0976	0	-10.173383	8.188646	-2.181784	L
H-H1-0.0976	0	-10.227398	7.699924	-3.892935	L
H-H1-0.0976	0	-10.487255	6.491134	-2.624470	L
C-CT-0.1200	-1	-8.868718	1.954738	3.255284	L
H-HC-0.0800	0	-8.146378	2.763018	3.358449	L
H-HC-0.0800	0	-9.683623	2.287889	2.612845	L
H-HC-0.0800	0	-9.270231	1.697122	4.235882	L
C-CT-0.2000	0	-8.218528	0.749954	2.643540	L
H-H1-0.0800	0	-8.972632	-0.029461	2.503258	L
O-OS--0.5600	0	-7.632079	1.104056	1.408432	L
C-CT-0.2000	0	-7.075287	0.226931	3.506316	L
H-H1-0.0800	0	-6.399619	1.034382	3.790916	L
O-OH--0.6800	0	-7.521795	-0.479980	4.655737	L
H-HO-0.4000	0	-8.129451	-1.168556	4.346435	L
C-CT-0.2000	0	-6.422180	-0.641589	2.441380	L
H-H1-0.0800	0	-5.389330	-0.888135	2.696452	L
O-OH--0.6800	0	-7.218069	-1.814093	2.293721	L
H-HO-0.4000	0	-6.739789	-2.426621	1.699768	L
C-CT-0.5691	0	-6.506745	0.239962	1.186907	H
H-H2-0.8000	0	-6.647186	-0.426870	0.313436	H
N-N*--0.5691	0	-5.323396	1.070971	0.991012	H
C-CM--0.0500	0	-4.683831	0.883181	-0.196808	H
H-H4-0.1500	0	-5.037264	0.029976	-0.728780	H
C-CM--0.1238	0	-3.745733	1.682138	-0.704892	H

C-C-0.6156	0	-3.456463	1.667795	-2.151607	H
O-O--0.5700	0	-2.662503	2.428637	-2.647262	H
N-N--0.8000	0	-4.222357	0.864343	-2.954830	H
H-H-0.3700	0	-3.871101	0.637865	-3.863537	H
H-H-0.3700	0	-4.814124	0.160986	-2.563077	H
C-CT-0.1164	0	-3.110894	2.704211	0.206372	H
H-HC-0.0800	0	-2.728038	3.539038	-0.361296	H
H-HC-0.0800	0	-2.251992	2.276236	0.719611	H
C-CM--0.2882	0	-4.147376	3.130541	1.219789	H
H-HA-0.1500	0	-4.069167	4.109716	1.648697	H
C-CM--0.0500	0	-5.132068	2.329420	1.586484	H
H-H4-0.1500	0	-5.851533	2.621832	2.320377	H
N-N3--0.8530	0	3.301376	0.893611	-0.272507	H
H-H-0.4500	0	3.247130	0.542050	-1.214851	H
H-H-0.4500	0	2.546608	0.547063	0.366466	H
H-H-0.4500	0	4.190010	0.662362	0.149476	H
C-CT-0.3170	0	3.423006	2.367043	-0.312231	H
H-HP-0.0800	0	4.214986	2.590077	-1.012894	H
C-CT--0.1600	0	2.139198	3.098194	-0.687691	H
H-HC-0.0800	0	1.951158	2.954855	-1.743984	H
H-HC-0.0800	0	2.350982	4.151430	-0.553013	H
C-CT--0.1600	0	0.931307	2.667205	0.149501	H
H-HC-0.0800	0	0.198221	3.460487	0.134445	H
H-HC-0.0800	0	1.257034	2.569211	1.179465	H
C-CT--0.0990	0	0.289401	1.354667	-0.335701	H
H-HC-0.0800	0	0.899680	0.899778	-1.111843	H
H-HC-0.0800	0	-0.652390	1.591979	-0.807608	H
C-C-0.4490	0	0.105129	0.312623	0.763726	H
H-H-0.0600	0	-0.404478	0.811559	1.596962	H
O-O--0.5700	0	1.322171	-0.178032	1.201761	H
N-N3--0.9900	0	-0.717237	-0.799786	0.292254	H
H-H-0.3600	0	0.929332	-1.212372	1.696959	H
H-H-0.3600	0	-1.175518	-0.519104	-0.563256	H
C-C-0.9060	0	3.949178	2.727852	1.071820	L
O-O2--0.9000	0	4.264468	1.829359	1.885822	L
O-O2--0.9000	0	4.052400	3.921611	1.419944	L
C-CT--0.205900	0	-1.654245	-1.383986	1.231171	H
H-H1-0.139900	0	-2.279216	-0.622868	1.704386	H
C-CT-0.007100	0	-2.566768	-2.308884	0.416096	H
H-HC--0.007800	0	-1.952493	-2.969583	-0.179637	H
H-HC--0.007800	0	-3.106494	-1.675459	-0.283897	H
C-CT-0.067500	0	-3.583414	-3.148134	1.189323	L
H-HC--0.054800	0	-3.799374	-2.649106	2.135770	L
H-HC--0.054800	0	-3.162246	-4.133192	1.402120	L
C-C-0.818300	0	-4.913041	-3.272021	0.440609	L
O-O2--0.822000	0	-5.581409	-2.223315	0.278596	L
O-O2--0.822000	0	-5.400427	-4.373215	0.089488	L
C-C-0.742000	0	-0.878264	-2.098520	2.335053	H
O-O2--0.793000	0	-1.445364	-2.661620	3.241316	H
O-O2--0.793000	0	0.393890	-2.128611	2.202830	H
O-OW--0.834000	0	2.699326	-5.704616	-3.525613	L
H-HW-0.417000	0	3.553343	-5.854325	-3.071408	L
H-HW-0.417000	0	2.106923	-5.447636	-2.802607	L
O-OW--0.834000	0	0.221412	-7.434503	4.592262	L
H-HW-0.417000	0	0.891160	-7.076627	5.208904	L

H-HW-0.417000	0	-0.315566	-7.995064	5.158756	L
O-OW--0.834000	0	4.163314	-0.119380	3.505001	L
H-HW-0.417000	0	3.294073	-0.470614	3.286332	L
H-HW-0.417000	0	4.266967	0.611892	2.845474	L
O-OW--0.834000	0	6.230913	-1.633110	4.351706	L
H-HW-0.417000	0	5.869325	-2.529889	4.453051	L
H-HW-0.417000	0	5.453661	-1.118211	4.052929	L
O-OW--0.834000	0	-6.023220	-6.970775	0.110983	L
H-HW-0.417000	0	-5.830724	-6.014434	0.176932	L
H-HW-0.417000	0	-6.674954	-7.041116	-0.594089	L
O-OW--0.834000	0	-8.849546	-1.034423	-0.148354	L
H-HW-0.417000	0	-9.726194	-1.054573	0.246442	L
H-HW-0.417000	0	-8.602171	-1.986993	-0.158933	L

I3

C-CT--0.3662	-1	-12.873741	1.033742	1.929819	L
H-HC-0.1123	0	-12.301000	1.914824	1.643799	L
H-HC-0.1123	0	-13.934401	1.239518	1.795819	L
H-HC-0.1123	0	-12.573473	0.188218	1.314738	L
C-C-0.5972	0	-12.605958	0.719006	3.388927	L
O-O--0.5679	0	-11.857641	1.444977	4.033005	L
N-N--0.415700	0	-13.193550	-0.369852	3.895599	L
H-H-0.271900	0	-13.784189	-0.897377	3.273735	L
C-CT--0.025200	-1	-12.905880	-0.932598	5.228186	L
H-H1-0.069800	0	-13.644378	-1.699230	5.461949	L
H-H1-0.069800	0	-12.970624	-0.140358	5.974704	L
C-C-0.597300	0	-11.483836	-1.566737	5.304987	L
O-O--0.567900	0	-10.649003	-1.393110	4.411191	L
N-N--0.415700	0	-11.190421	-2.335554	6.365217	L
H-H-0.271900	0	-11.911117	-2.473473	7.055912	L
C-CT--0.025200	-1	-9.993664	-3.203990	6.440943	L
H-H1-0.069800	0	-9.907928	-3.623815	7.442849	L
H-H1-0.069800	0	-9.099500	-2.618784	6.222613	L
C-C-0.597300	0	-10.122362	-4.349739	5.446098	L
O-O--0.567900	0	-11.163433	-4.998562	5.374490	L
N-N--0.4157	0	-9.064063	-4.591893	4.682240	L
H-H-0.2719	0	-8.231821	-4.047371	4.833149	L
C-CT--0.0014	-1	-9.005452	-5.543965	3.574516	L
H-H1-0.0876	0	-9.911753	-5.458423	2.974128	L
C-C-0.5973	0	-8.890399	-7.018169	4.023903	L
O-O--0.5679	0	-8.125684	-7.805049	3.472684	L
C-CT--0.0152	0	-7.808373	-5.140691	2.697408	L
H-HC-0.0295	0	-8.032511	-4.163840	2.288387	L
H-HC-0.0295	0	-7.767564	-5.813678	1.841315	L
C-CA--0.0011	0	-6.425385	-5.086261	3.354730	L
C-CA--0.1906	0	-5.501030	-6.125782	3.128705	L
H-HA-0.1699	0	-5.804120	-6.996305	2.559206	L
C-CA--0.1906	0	-6.017787	-3.964892	4.111158	L
H-HA-0.1699	0	-6.698701	-3.157816	4.319675	L
C-CA--0.2341	0	-4.176655	-6.027666	3.599832	L
H-HA-0.1656	0	-3.463849	-6.809154	3.391152	L
C-CA--0.2341	0	-4.688968	-3.848332	4.560139	L
H-HA-0.1656	0	-4.352892	-2.966127	5.080225	L
C-C-0.3326	0	-3.759834	-4.873029	4.291383	L
O-OH--0.5579	0	-2.459928	-4.714131	4.647929	L

H-HO-0.3992	0	-2.157962	-3.854984	4.269087	L
N-N--0.4157	0	-9.661570	-7.412725	5.037640	L
H-H-0.2719	0	-10.331790	-6.736688	5.384613	L
C-CT--0.1490	-1	-9.578924	-8.735865	5.631385	L
H-H1-0.0976	0	-8.569072	-8.908483	6.008517	L
H-H1-0.0976	0	-10.293205	-8.826703	6.450387	L
H-H1-0.0976	0	-9.800318	-9.491426	4.874693	L
C-CT--0.3662	-1	-12.001091	-1.450860	-3.483940	L
H-HC-0.1123	0	-12.518033	-0.536978	-3.772404	L
H-HC-0.1123	0	-12.006823	-1.569710	-2.402781	L
H-HC-0.1123	0	-12.498843	-2.303880	-3.944153	L
C-C-0.5972	0	-10.569748	-1.400658	-3.982879	L
O-O--0.5679	0	-10.109993	-2.263433	-4.718372	L
N-N--0.415700	0	-9.845433	-0.388994	-3.527836	L
H-H-0.271900	0	-10.333720	0.280924	-2.949808	L
C-CT--0.038900	-1	-8.403739	-0.099547	-3.687764	L
H-H1-0.100700	0	-8.107177	-0.121692	-4.734308	L
C-C-0.597300	0	-7.553559	-1.078476	-2.875684	L
O-O--0.567900	0	-6.756593	-0.685359	-2.019044	L
C-CT-0.365400	0	-8.227810	1.324382	-3.109413	L
H-H1-0.004300	0	-7.170248	1.577557	-3.092371	L
O-OH--0.676100	0	-8.770427	1.413274	-1.800566	L
H-HO-0.410200	0	-8.593100	0.591154	-1.319349	L
C-CT--0.243800	0	-9.002229	2.402284	-3.865308	L
H-HC-0.064200	0	-10.056410	2.150284	-3.964674	L
H-HC-0.064200	0	-8.580951	2.553654	-4.849439	L
H-HC-0.064200	0	-8.935894	3.340160	-3.320784	L
N-N--0.415700	0	-7.770986	-2.369823	-3.082522	L
H-H-0.271900	0	-8.438811	-2.631184	-3.804117	L
C-CT--0.024900	-1	-7.195582	-3.449389	-2.288936	L
H-H1-0.084300	0	-6.327560	-3.082215	-1.761176	L
C-C-0.597300	0	-6.687810	-4.579993	-3.187868	L
O-O--0.567900	0	-7.180527	-4.756340	-4.299729	L
C-CT-0.211700	0	-8.221751	-4.055506	-1.330204	L
H-H1-0.035200	0	-7.953351	-5.086128	-1.092181	L
H-H1-0.035200	0	-9.219510	-4.039774	-1.771803	L
O-OH--0.654600	0	-8.184508	-3.307526	-0.147127	L
H-HO-0.427500	0	-7.233139	-3.296537	0.096408	L
N-N--0.4157	0	-5.717853	-5.368116	-2.714091	L
H-H-0.2719	0	-5.352046	-5.164603	-1.784965	L
C-CT--0.0014	0	-5.317330	-6.605961	-3.393065	L
H-H1-0.0876	0	-5.001650	-6.356931	-4.406235	L
C-C-0.5973	0	-6.465623	-7.613785	-3.470446	L
O-O--0.5679	0	-7.354922	-7.627516	-2.617692	L
C-CT--0.0152	0	-4.168385	-7.321258	-2.672740	L
H-HC-0.0295	0	-3.886088	-8.196938	-3.258677	L
H-HC-0.0295	0	-4.519964	-7.702060	-1.715828	L
C-CA--0.0011	-1	-2.919345	-6.520010	-2.441720	L
C-CA--0.1906	0	-1.992957	-6.337305	-3.489530	L
H-HA-0.1699	0	-2.216064	-6.714425	-4.477087	L
C-CA--0.1906	0	-2.610602	-6.059334	-1.148173	L
H-HA-0.1699	0	-3.311926	-6.211566	-0.335198	L
C-CA--0.2341	0	-0.770511	-5.678631	-3.248187	L
H-HA-0.1656	0	-0.057693	-5.526112	-4.043392	L
C-CA--0.2341	0	-1.385807	-5.413494	-0.901336	L

H-HA-0.1656	0	-1.163688	-5.064806	0.095745	L
C-C-0.3226	0	-0.467873	-5.210890	-1.952049	L
O-OH--0.5579	0	0.702193	-4.561139	-1.715510	L
H-HO-0.3992	0	0.570009	-3.992421	-0.946086	L
N-N--0.4157	0	-6.353565	-8.528069	-4.437629	L
H-H-0.2719	0	-5.598001	-8.414023	-5.092746	L
C-CT--0.1490	-1	-7.140726	-9.744516	-4.497081	L
H-H1-0.0976	0	-8.199526	-9.490590	-4.583647	L
H-H1-0.0976	0	-6.845231	-10.348030	-5.356161	L
H-H1-0.0976	0	-6.998313	-10.322523	-3.581480	L
C-CT--0.3662	-1	-6.328110	-3.490308	-6.973852	L
H-HC-0.1123	0	-5.835152	-4.241528	-7.587412	L
H-HC-0.1123	0	-7.370288	-3.771798	-6.827736	L
H-HC-0.1123	0	-5.828450	-3.416547	-6.013503	L
C-C-0.5972	0	-6.246611	-2.143187	-7.675083	L
O-O--0.5679	0	-5.360592	-1.970689	-8.500451	L
N-N--0.415700	0	-7.142027	-1.197964	-7.351445	L
H-H-0.271900	0	-7.856597	-1.472022	-6.694438	L
C-CT--0.025200	-1	-7.280491	0.119832	-8.022180	L
H-H1-0.069800	0	-8.229299	0.135262	-8.558802	L
H-H1-0.069800	0	-6.492814	0.250442	-8.763672	L
C-C-0.597300	0	-7.256136	1.328594	-7.092593	L
O-O--0.567900	0	-8.137962	2.170718	-7.195345	L
N-N--0.415700	0	-6.253826	1.413152	-6.211817	L
H-H-0.271900	0	-5.567812	0.666431	-6.231561	L
C-CT--0.025200	-1	-5.821096	2.650265	-5.550777	L
H-H1-0.069800	0	-5.646312	2.466662	-4.493727	L
H-H1-0.069800	0	-6.581782	3.425237	-5.656036	L
C-C-0.597300	0	-4.533517	3.165106	-6.203903	L
O-O--0.567900	0	-4.470315	3.298525	-7.423206	L
N-N--0.415700	0	-3.489975	3.392915	-5.403843	L
H-H-0.271900	0	-3.608561	3.238672	-4.412576	L
C-CT--0.051800	-1	-2.153497	3.786010	-5.866666	L
H-H1-0.092200	0	-2.238910	4.242367	-6.854329	L
C-C-0.597300	0	-1.230473	2.556283	-5.994273	L
O-O--0.567900	0	-0.540168	2.357759	-6.990906	L
C-CT--0.110200	0	-1.611011	4.837908	-4.878713	L
H-HC-0.045700	0	-1.584488	4.401136	-3.880257	L
H-HC-0.045700	0	-2.307856	5.676914	-4.853083	L
C-CT-0.353100	0	-0.208147	5.377194	-5.203521	L
H-HC--0.036100	0	0.506145	4.553935	-5.218186	L
C-CT--0.412100	0	-0.154953	6.106624	-6.545770	L
H-HC-0.100000	0	-0.906581	6.896636	-6.571440	L
H-HC-0.100000	0	-0.348840	5.404170	-7.356353	L
H-HC-0.100000	0	0.833312	6.539466	-6.696151	L
C-CT--0.412100	0	0.214057	6.358195	-4.107453	L
H-HC-0.100000	0	-0.490734	7.189468	-4.063041	L
H-HC-0.100000	0	1.210695	6.743772	-4.320387	L
H-HC-0.100000	0	0.229612	5.846718	-3.146132	L
N-N--0.516300	0	-1.189531	1.741242	-4.943647	L
H-H-0.293600	0	-1.770432	2.017262	-4.158758	L
C-CT-0.038100	-1	-0.297804	0.606012	-4.721996	L
H-H1-0.088000	0	-0.058535	0.113680	-5.662345	L
C-C-0.536600	0	-0.978570	-0.385622	-3.747637	L
O-O--0.581900	0	-0.840653	-0.241396	-2.540080	L

C-CT--0.030300	0	1.009146	1.197068	-4.115219	L
H-HC--0.012200	0	0.744742	1.819865	-3.261240	L
H-HC--0.012200	0	1.470578	1.849344	-4.858644	L
C-C-0.799400	-1	2.066248	0.175051	-3.669522	L
O-O2--0.801400	0	2.023843	-0.962844	-4.190466	L
O-O2--0.801400	0	2.998572	0.519125	-2.914308	L
N-N--0.254800	0	-1.758512	-1.372432	-4.215477	L
C-CT--0.026600	-1	-2.044476	-1.666506	-5.610334	L
H-H1-0.064100	0	-1.118519	-1.722671	-6.181455	L
C-C-0.589600	0	-2.986531	-0.642051	-6.269132	L
O-O--0.574800	0	-3.918373	-0.147807	-5.631662	L
C-CT--0.007000	0	-2.691112	-3.055777	-5.584285	L
H-HC-0.025300	0	-1.911643	-3.819286	-5.583564	L
H-HC-0.025300	0	-3.371602	-3.212909	-6.421823	L
C-CT-0.018900	0	-3.420162	-3.076189	-4.241797	L
H-HC-0.021300	0	-3.576446	-4.089337	-3.880860	L
H-HC-0.021300	0	-4.369191	-2.544281	-4.319563	L
C-CT-0.019200	0	-2.469968	-2.295759	-3.340935	L
H-H1-0.039100	0	-3.031333	-1.761521	-2.574555	L
H-H1-0.039100	0	-1.755758	-2.971828	-2.872321	L
N-N--0.4157	0	-2.745656	-0.357007	-7.553574	L
H-H-0.2719	0	-1.965093	-0.814800	-7.991490	L
C-CT--0.1490	-1	-3.534212	0.531649	-8.406825	L
H-H1-0.0976	0	-4.495716	0.747869	-7.954298	L
H-H1-0.0976	0	-3.710237	0.061401	-9.375279	L
H-H1-0.0976	0	-3.001798	1.473808	-8.555386	L
C-CT--0.3662	-1	8.751861	1.923242	-5.905660	L
H-HC-0.1123	0	9.246361	2.173905	-6.843827	L
H-HC-0.1123	0	9.503669	1.706091	-5.149757	L
H-HC-0.1123	0	8.103453	1.062719	-6.057553	L
C-C-0.5972	0	7.925920	3.109678	-5.460246	L
O-O--0.5679	0	8.056822	4.187703	-6.020611	L
N-N--0.415700	0	7.075813	2.919094	-4.451297	L
H-H-0.271900	0	7.018186	1.991138	-4.056727	L
C-CT-0.021300	-1	6.082102	3.910820	-4.015935	L
H-H1-0.112400	0	6.265957	4.863573	-4.517045	L
C-C-0.597300	0	6.120829	4.151877	-2.504347	L
O-O--0.567900	0	6.374490	3.232548	-1.724803	L
C-CT--0.123100	0	4.706910	3.385223	-4.463594	L
H-H1-0.111200	0	4.510248	2.417813	-3.993803	L
H-H1-0.111200	0	4.710588	3.252904	-5.547310	L
S-SH--0.311900	0	3.384541	4.545510	-4.016049	L
H-HS-0.193300	0	2.380652	3.896465	-4.624057	L
N-N--0.415700	0	5.846212	5.385576	-2.082579	L
H-H-0.271900	0	5.600990	6.080453	-2.774965	L
C-CT--0.025200	-1	5.822364	5.788767	-0.681772	L
H-H1-0.069800	0	6.786034	6.229906	-0.427753	L
H-H1-0.069800	0	5.678507	4.923303	-0.039092	L
C-C-0.597300	0	4.743868	6.828101	-0.398401	L
O-O--0.567900	0	4.854078	7.975028	-0.821873	L
N-N--0.415700	0	3.727802	6.446942	0.370408	L
H-H-0.271900	0	3.775259	5.503653	0.747148	L
C-CT--0.025200	-1	2.689463	7.317698	0.908689	L
H-H1-0.069800	0	1.858842	6.700170	1.236316	L
H-H1-0.069800	0	2.346117	8.017453	0.146101	L

C-C-0.597300	0	3.189433	8.097882	2.122518	L
O-O--0.567900	0	2.614764	7.986587	3.203277	L
N-N--0.4157	0	4.274934	8.856252	1.936273	L
H-H-0.2719	0	4.632248	8.875112	0.986032	L
C-CT--0.1490	-1	5.016958	9.541211	2.985675	L
H-H1-0.0976	0	5.344512	8.820890	3.737234	L
H-H1-0.0976	0	5.886840	10.045046	2.561646	L
H-H1-0.0976	0	4.373644	10.280599	3.466746	L
C-CT--0.3662	-1	-1.510309	9.436761	5.300201	L
H-HC-0.1123	0	-1.722621	10.402244	4.843800	L
H-HC-0.1123	0	-0.570004	9.048379	4.912540	L
H-HC-0.1123	0	-1.450315	9.554201	6.380703	L
C-C-0.5972	0	-2.642950	8.464524	4.958835	L
O-O--0.5679	0	-3.527312	8.819711	4.190100	L
N-N--0.4157	0	-2.615317	7.238105	5.496955	L
H-H-0.2719	0	-1.867934	7.024205	6.142924	L
C-CT--0.0275	-1	-3.605896	6.145174	5.314355	L
H-H1-0.1123	0	-4.604308	6.578889	5.250206	L
C-C-0.5973	0	-3.572856	5.171065	6.521629	L
O-O--0.5679	0	-2.656575	5.230934	7.340335	L
C-CT--0.0050	0	-3.310726	5.368687	4.021047	L
H-HC-0.0339	0	-3.494738	6.025461	3.178075	L
H-HC-0.0339	0	-4.043110	4.566568	3.936414	L
C-C*--0.1415	0	-1.939780	4.761008	3.877065	L
C-CW--0.1638	0	-0.781565	5.438867	3.687529	L
H-H4-0.2062	0	-0.693701	6.515532	3.621191	L
C-CB-0.1243	0	-1.565600	3.348079	3.912343	L
N-NA--0.3418	0	0.275622	4.552473	3.607090	L
H-H-0.3412	0	1.242601	4.831083	3.494952	L
C-CN-0.1380	0	-0.150197	3.250634	3.747871	L
C-CA--0.238	0	-2.276991	2.138211	4.076266	L
H-HA-0.1700	0	-3.349219	2.167360	4.181849	L
C-CA--0.2601	0	0.527169	2.023320	3.762629	L
H-HA-0.1572	0	1.598809	1.982626	3.629513	L
C-CA--0.1972	0	-1.608524	0.899334	4.083674	L
H-HA-0.1447	0	-2.160256	-0.027015	4.185833	L
C-CA--0.1134	0	-0.213162	0.843004	3.932298	L
H-HA-0.1417	0	0.282632	-0.116733	3.922246	L
N-N--0.4157	0	-4.556634	4.260075	6.632843	L
H-H-0.2719	0	-5.274494	4.289477	5.927508	L
C-CT--0.1490	-1	-4.650901	3.231717	7.684097	L
H-H1-0.0976	0	-3.831862	2.519388	7.572804	L
H-H1-0.0976	0	-4.572286	3.703220	8.666270	L
H-H1-0.0976	0	-5.603180	2.703753	7.615134	L
C-CT--0.3662	-1	-2.803445	-1.486816	7.274815	L
H-HC-0.1123	0	-3.027531	-2.547902	7.164923	L
H-HC-0.1123	0	-3.167128	-1.136194	8.238094	L
H-HC-0.1123	0	-3.277520	-0.938770	6.462603	L
C-C-0.5972	0	-1.311168	-1.297235	7.186319	L
O-O--0.5679	0	-0.595650	-2.251075	6.934518	L
N-N--0.415700	0	-0.815203	-0.092102	7.463023	L
H-H-0.271900	0	-1.474582	0.660194	7.584913	L
C-CT--0.087500	-1	0.627323	0.237407	7.422220	L
H-H1-0.096900	0	0.954657	0.131670	6.387422	L
C-C-0.597300	0	1.475803	-0.724373	8.266045	L

O-O--0.567900	0	2.476411	-1.248401	7.788207	L
C-CT-0.298500	0	0.905434	1.693100	7.867794	L
H-HC--0.029700	0	0.956563	1.728090	8.956681	L
C-CT--0.319200	0	2.252352	2.166336	7.307415	L
H-HC-0.079100	0	2.218494	2.176786	6.217564	L
H-HC-0.079100	0	3.050661	1.500642	7.636084	L
H-HC-0.079100	0	2.468997	3.172438	7.667253	L
C-CT--0.319200	0	-0.174489	2.694779	7.436534	L
H-HC-0.079100	0	-0.361964	2.605327	6.370332	L
H-HC-0.079100	0	0.147209	3.713989	7.653321	L
H-HC-0.079100	0	-1.101214	2.520584	7.981321	L
N-N--0.415700	0	1.055651	-0.995274	9.509297	L
H-H-0.271900	0	0.224823	-0.519058	9.821102	L
C-CT--0.025200	-1	1.666338	-1.998658	10.397192	L
H-H1-0.069800	0	1.126992	-2.027278	11.343368	L
H-H1-0.069800	0	2.699006	-1.713498	10.601433	L
C-C-0.597300	0	1.679608	-3.429528	9.808432	L
O-O--0.567900	0	2.494517	-4.242334	10.227402	L
N-N--0.415700	0	0.791074	-3.743825	8.853952	L
H-H-0.271900	0	0.192179	-3.007442	8.507677	L
C-CT-0.033700	-1	0.662306	-5.033560	8.162599	L
H-H1-0.082300	0	1.120567	-5.802378	8.787676	L
C-C-0.597300	0	1.389281	-5.096454	6.794083	L
O-O--0.567900	0	1.832125	-6.167559	6.378419	L
C-CT--0.182500	0	-0.825697	-5.383997	8.026076	L
H-HC-0.060300	0	-1.306294	-5.355258	9.004396	L
H-HC-0.060300	0	-0.925939	-6.388857	7.614882	L
H-HC-0.060300	0	-1.324254	-4.684247	7.355469	L
N-N--0.415700	0	1.518376	-3.969576	6.088646	L
H-H-0.271900	0	0.983620	-3.175051	6.420589	L
C-CT-0.033700	-1	2.459275	-3.720931	4.982811	L
H-H1-0.082300	0	2.304184	-4.437346	4.180174	L
C-C-0.597300	0	3.926679	-3.804716	5.460299	L
O-O--0.567900	0	4.827668	-4.110693	4.677775	L
C-CT--0.182500	0	2.191162	-2.296792	4.503014	L
H-HC-0.060300	0	2.715463	-1.570779	5.124861	L
H-HC-0.060300	0	1.125579	-2.085069	4.565109	L
H-HC-0.060300	0	2.531812	-2.205456	3.475587	L
N-N--0.4157	0	4.167408	-3.446433	6.730191	L
H-H-0.2719	0	3.372973	-3.073556	7.235632	L
C-CT--0.1490	-1	5.444818	-3.492101	7.427581	L
H-H1-0.0976	0	5.948692	-4.436510	7.212666	L
H-H1-0.0976	0	5.281009	-3.412424	8.503623	L
H-H1-0.0976	0	6.077074	-2.667985	7.093426	L
C-CT--0.3662	-1	14.089910	0.939438	-0.681757	L
H-HC-0.1123	0	13.434672	1.443968	0.025447	L
H-HC-0.1123	0	15.094378	1.354928	-0.615560	L
H-HC-0.1123	0	13.709755	1.059387	-1.694075	L
C-C-0.5972	0	14.147217	-0.541649	-0.346906	L
O-O--0.5679	0	15.228717	-1.102281	-0.235133	L
N-N--0.4157	0	12.986584	-1.180532	-0.203835	L
H-H-0.2719	0	12.143660	-0.631424	-0.324692	L
C-CT--0.0014	-1	12.811282	-2.633665	0.000718	L
H-H1-0.0876	0	13.691885	-3.151442	-0.382127	L
C-C-0.5973	0	11.584034	-3.127072	-0.818087	L

O-O--0.5679	0	10.822034	-2.324844	-1.367143	L
C-CT--0.0152	0	12.685797	-2.942869	1.503462	L
H-HC-0.0295	0	13.627640	-2.678073	1.986578	L
H-HC-0.0295	0	12.555218	-4.017580	1.634916	L
C-CA--0.0011	-1	11.555346	-2.220960	2.217255	L
C-CA--0.1906	0	11.791773	-0.977915	2.834172	L
H-HA-0.1699	0	12.785343	-0.551082	2.815823	L
C-CA--0.1906	0	10.261116	-2.777670	2.242254	L
H-HA-0.1699	0	10.070216	-3.736775	1.781427	L
C-CA--0.2341	0	10.739377	-0.294783	3.472519	L
H-HA-0.1656	0	10.925102	0.646148	3.957424	L
C-CA--0.2341	0	9.204769	-2.092582	2.872969	L
H-HA-0.1656	0	8.217282	-2.528250	2.888901	L
C-C-0.3226	0	9.440093	-0.846587	3.493605	L
O-OH--0.557	0	8.425913	-0.179371	4.109418	L
H-HO-0.3992	0	7.620040	-0.735294	4.119743	L
N-N--0.415700	0	11.376315	-4.445042	-0.941358	L
H-H-0.271900	0	11.999156	-5.066305	-0.449088	L
C-CT--0.025200	-1	10.254741	-5.039254	-1.698437	L
H-H1-0.069800	0	10.469913	-6.090695	-1.887902	L
H-H1-0.069800	0	10.151511	-4.538859	-2.660452	L
C-C-0.597300	0	8.928464	-4.965557	-0.929159	L
O-O--0.567900	0	8.863371	-5.366644	0.231459	L
N-N--0.415700	0	7.857702	-4.477755	-1.562260	L
H-H-0.271900	0	7.939350	-4.345403	-2.563113	L
C-CT-0.014300	-1	6.520829	-4.453499	-0.969877	L
H-H1-0.104800	0	6.617932	-4.362462	0.114939	L
C-C-0.597300	0	5.778187	-5.760857	-1.251605	L
O-O--0.567900	0	5.055534	-5.884739	-2.245876	L
C-CT--0.204100	0	5.710767	-3.223881	-1.438102	L
H-HC-0.079700	0	5.581717	-3.264722	-2.519363	L
H-HC-0.079700	0	6.285227	-2.342037	-1.190499	L
C-C-0.713000	-1	4.324070	-3.094038	-0.785470	L
O-O--0.593100	0	3.929949	-3.840395	0.098933	L
N-N--0.919100	0	3.500053	-2.182011	-1.259628	L
H-H-0.419600	0	2.589624	-2.143749	-0.830745	L
H-H-0.419600	0	3.709167	-1.582832	-2.048537	L
N-N--0.347900	0	5.930589	-6.709599	-0.324936	L
H-H-0.274700	0	6.601561	-6.487359	0.400484	L
C-CT--0.263700	-1	5.377146	-8.068229	-0.387945	L
H-H1-0.156000	0	5.755027	-8.609875	0.479355	L
C-C-0.734100	0	5.883415	-8.860914	-1.603492	L
O-O--0.589400	0	6.889751	-8.516440	-2.216559	L
C-CT--0.000700	0	3.834855	-8.037058	-0.284425	L
H-HC-0.032700	0	3.457409	-9.059219	-0.238289	L
H-HC-0.032700	0	3.425906	-7.573618	-1.183497	L
C-CT-0.039000	0	3.288946	-7.306178	0.947310	L
H-HC-0.028500	0	3.648089	-6.279010	0.972983	L
H-HC-0.028500	0	3.605348	-7.821129	1.855494	L
C-CT-0.048600	0	1.761631	-7.309935	0.850594	L
H-H1-0.068700	0	1.406855	-8.341986	0.829561	L
H-H1-0.068700	0	1.455158	-6.828528	-0.080845	L
N-N2--0.529500	-1	1.133160	-6.622521	1.981560	L
H-H-0.345600	0	0.748856	-7.171687	2.745085	L
C-CA-0.807600	0	0.860624	-5.345193	2.081973	L

N-N2--0.862700	0	0.039708	-4.958924	3.002424	L
H-H-0.447800	0	-0.376300	-5.617851	3.644880	L
H-H-0.447800	0	-0.196087	-3.965141	3.068322	L
N-N2--0.862700	0	1.375449	-4.441477	1.301896	L
H-H-0.447800	0	2.235843	-4.596769	0.794196	L
H-H-0.447800	0	1.070473	-3.492254	1.530905	L
N-N--0.4157	0	5.201760	-9.968066	-1.904821	L
H-H-0.2719	0	4.407537	-10.175881	-1.323972	L
C-CT--0.1490	-1	5.504959	-10.859444	-3.009560	L
H-H1-0.0976	0	6.516845	-11.255013	-2.897214	L
H-H1-0.0976	0	4.796606	-11.688987	-3.033704	L
H-H1-0.0976	0	5.449845	-10.310815	-3.951950	L
C-CT--0.3662	-1	10.157902	-5.911651	-7.773833	L
H-HC-0.1123	0	9.872727	-6.614267	-8.554360	L
H-HC-0.1123	0	11.058639	-5.380312	-8.079797	L
H-HC-0.1123	0	10.350903	-6.437924	-6.840545	L
C-C-0.5972	0	9.043878	-4.906571	-7.563749	L
O-O--0.5679	0	9.269470	-3.710127	-7.708990	L
N-N--0.347900	0	7.847811	-5.360705	-7.158422	L
H-H-0.274700	0	7.768091	-6.356397	-7.016767	L
C-CT--0.263700	-1	6.873359	-4.461377	-6.505761	L
H-H1-0.156000	0	6.688822	-3.601908	-7.151174	L
C-C-0.734100	0	7.527115	-3.961989	-5.214589	L
O-O--0.589400	0	7.902342	-4.767310	-4.367508	L
C-CT--0.000700	0	5.540944	-5.157688	-6.180007	L
H-HC-0.032700	0	4.949363	-4.455831	-5.592864	L
H-HC-0.032700	0	5.718500	-6.034596	-5.553568	L
C-CT-0.039000	0	4.741965	-5.578308	-7.425163	L
H-HC-0.028500	0	4.994834	-6.612040	-7.664311	L
H-HC-0.028500	0	5.020389	-4.957535	-8.278379	L
C-CT-0.048600	0	3.222406	-5.467620	-7.207156	L
H-H1-0.068700	0	2.960422	-5.865592	-6.224466	L
H-H1-0.068700	0	2.724218	-6.085735	-7.956434	L
N-N2--0.529500	0	2.752463	-4.074092	-7.368936	L
H-H-0.345600	0	2.690172	-3.725340	-8.308956	L
C-CA-0.807600	0	2.455545	-3.194403	-6.426844	L
N-N2--0.862700	0	2.427130	-3.464455	-5.160362	L
H-H-0.447800	0	2.587811	-4.392864	-4.781685	L
H-H-0.447800	0	2.229600	-2.684694	-4.527750	L
N-N2--0.862700	0	2.183123	-1.961640	-6.728719	L
H-H-0.447800	0	2.191565	-1.583475	-7.654672	L
H-H-0.447800	0	2.059364	-1.365146	-5.906416	L
N-N--0.415700	0	7.720610	-2.654506	-5.078105	L
H-H-0.271900	0	7.398850	-2.055013	-5.820130	L
C-CT--0.025200	-1	8.619957	-2.082941	-4.073128	L
H-H1-0.069800	0	9.493744	-1.681580	-4.587207	L
H-H1-0.069800	0	8.977166	-2.847450	-3.388008	L
C-C-0.597300	0	8.015772	-0.960890	-3.247911	L
O-O--0.567900	0	6.946096	-0.435299	-3.559812	L
N-N--0.415700	0	8.712935	-0.567607	-2.185462	L
H-H-0.271900	0	9.574663	-1.054155	-1.941146	L
C-CT--0.038900	-1	8.308423	0.567777	-1.361761	L
H-H1-0.100700	0	7.675043	1.195784	-1.984861	L
C-C-0.597300	0	9.447098	1.495492	-0.963863	L
O-O--0.567900	0	10.556021	1.079053	-0.633599	L

C-CT-0.365400	0	7.425283	0.129056	-0.178130	L
H-H1-0.004300	0	7.744194	-0.853724	0.172159	L
O-OH--0.676100	0	6.106523	0.060545	-0.681201	L
H-HO-0.410200	0	6.176700	-0.170875	-1.620086	L
C-CT--0.243800	0	7.349077	1.078421	1.018087	L
H-HC-0.064200	0	6.991667	2.058796	0.704893	L
H-HC-0.064200	0	8.329514	1.170783	1.484175	L
H-HC-0.064200	0	6.659655	0.669944	1.756956	L
N-N--0.415700	0	9.123470	2.788008	-1.023858	L
H-H-0.271900	0	8.156177	2.995190	-1.251315	L
C-CT--0.025200	-1	9.960476	3.914013	-0.655844	L
H-H1-0.069800	0	9.765612	4.741783	-1.337583	L
H-H1-0.069800	0	11.011747	3.634244	-0.743410	L
C-C-0.597300	0	9.661252	4.379718	0.756636	L
O-O--0.567900	0	8.511408	4.567634	1.167283	L
N-N--0.347900	0	10.735900	4.599720	1.498269	L
H-H-0.274700	0	11.642319	4.461341	1.065709	L
C-CT--0.263700	-1	10.744881	5.187111	2.828804	L
H-H1-0.156000	0	9.839161	5.770359	2.983773	L
C-C-0.734100	0	11.914368	6.143328	2.933342	L
O-O--0.589400	0	12.859870	6.067881	2.154053	L
C-CT--0.000700	0	10.897878	4.077650	3.891964	L
H-HC-0.032700	0	11.177846	4.520243	4.849561	L
H-HC-0.032700	0	11.709108	3.409896	3.594087	L
C-CT-0.039000	0	9.633217	3.244651	4.111532	L
H-HC-0.028500	0	9.868757	2.422129	4.786230	L
H-HC-0.028500	0	9.289704	2.827477	3.164693	L
C-CT-0.048600	0	8.528549	4.080855	4.758476	L
H-H1-0.068700	0	8.337267	4.980708	4.174890	L
H-H1-0.068700	0	8.850291	4.385059	5.756234	L
N-N2--0.529500	-1	7.295921	3.299784	4.867535	L
H-H-0.345600	0	7.205830	2.676287	5.650910	L
C-CA-0.807600	0	6.295803	3.277186	4.018686	L
N-N2--0.862700	0	5.247549	2.562218	4.281633	L
H-H-0.447800	0	5.213964	1.920604	5.047533	L
H-H-0.447800	0	4.601490	2.436168	3.497018	L
N-N2--0.862700	0	6.317376	3.947982	2.909568	L
H-H-0.447800	0	7.171287	4.314892	2.513683	L
H-H-0.447800	0	5.457090	3.958900	2.362620	L
N-N--0.4157	0	11.833700	7.019235	3.925717	L
H-H-0.2719	0	11.019297	7.000507	4.515678	L
C-CT--0.1490	-1	12.897549	7.944523	4.221067	L
H-H1-0.0976	0	12.633454	8.568501	5.076451	L
H-H1-0.0976	0	13.815729	7.397720	4.448437	L
H-H1-0.0976	0	13.084324	8.585999	3.356542	L
C-CT--0.3662	-1	-2.167891	13.260444	-1.511998	L
H-HC-0.1123	0	-2.587234	13.770403	-0.647204	L
H-HC-0.1123	0	-1.081077	13.245177	-1.459772	L
H-HC-0.1123	0	-2.474521	13.781275	-2.418855	L
C-C-0.5972	0	-2.693513	11.837788	-1.570298	L
O-O--0.5679	0	-3.347669	11.466882	-2.532146	L
N-N--0.415700	0	-2.414074	11.049541	-0.528699	L
H-H-0.271900	0	-1.852258	11.465542	0.194871	L
C-CT--0.023700	-1	-2.834322	9.641362	-0.364570	L
H-H1-0.088000	0	-2.370259	9.055002	-1.159318	L

C-C-0.597300	0	-4.353846	9.455325	-0.520673	L
O-O--0.567900	0	-4.816046	8.668957	-1.347337	L
C-CT-0.034200	0	-2.283596	9.149589	0.980574	L
H-HC-0.024100	0	-2.646029	9.799699	1.777988	L
H-HC-0.024100	0	-1.195446	9.219165	0.960649	L
C-CT-0.001800	0	-2.671018	7.714008	1.322831	L
H-H1-0.044000	0	-3.742627	7.691099	1.524202	L
H-H1-0.044000	0	-2.155798	7.451947	2.243107	L
S-S--0.273700	0	-2.313252	6.437529	0.090257	L
C-CT--0.053600	0	-0.511805	6.405863	0.070963	L
H-H1-0.068400	0	-0.174076	5.684379	-0.673106	L
H-H1-0.068400	0	-0.155651	6.102692	1.053531	L
H-H1-0.068400	0	-0.126697	7.392300	-0.185179	L
N-N--0.4157	0	-5.137916	10.192314	0.270520	L
H-H-0.2719	0	-4.682094	10.809513	0.922942	L
C-CT--0.1490	-1	-6.588701	10.210018	0.168058	L
H-H1-0.0976	0	-7.011247	10.874577	0.922941	L
H-H1-0.0976	0	-6.881391	10.561347	-0.823745	L
H-H1-0.0976	0	-6.981162	9.202260	0.315755	L
C-CT--0.3662	-1	-4.444318	9.002472	-5.117374	L
H-HC-0.1123	0	-4.016669	9.359662	-4.181361	L
H-HC-0.1123	0	-3.680263	9.008587	-5.892649	L
H-HC-0.1123	0	-5.272149	9.640594	-5.417980	L
C-C-0.5972	0	-4.950943	7.578378	-4.937672	L
O-O--0.5679	0	-4.650587	6.718455	-5.753423	L
N-N--0.415700	0	-5.728558	7.332749	-3.879379	L
H-H-0.271900	0	-5.760660	8.080254	-3.198001	L
C-CT--0.087500	-1	-6.393831	6.041710	-3.557287	L
H-H1-0.096900	0	-6.106738	5.297209	-4.299140	L
C-C-0.597300	0	-7.907014	6.205581	-3.661702	L
O-O--0.567900	0	-8.544218	5.534408	-4.463832	L
C-CT-0.298500	0	-5.945729	5.543060	-2.174145	L
H-HC--0.029700	0	-6.148128	6.308964	-1.424974	L
C-CT--0.319200	0	-6.634074	4.250470	-1.732589	L
H-HC-0.079100	0	-6.431442	3.471458	-2.461216	L
H-HC-0.079100	0	-7.708896	4.401565	-1.642349	L
H-HC-0.079100	0	-6.252120	3.934574	-0.761777	L
C-CT--0.319200	0	-4.447776	5.256378	-2.205244	L
H-HC-0.079100	0	-4.219610	4.518310	-2.969933	L
H-HC-0.079100	0	-4.152693	4.879724	-1.235019	L
H-HC-0.079100	0	-3.887720	6.168454	-2.409441	L
N-N--0.4157	0	-8.491245	7.108274	-2.865911	L
H-H-0.2719	0	-7.883999	7.632404	-2.256064	L
C-CT--0.1490	-1	-9.920865	7.399165	-2.899196	L
H-H1-0.0976	0	-10.168972	8.170467	-2.169549	L
H-H1-0.0976	0	-10.200137	7.742880	-3.897625	L
H-H1-0.0976	0	-10.485612	6.491653	-2.676801	L
C-CT-0.1200	-1	-8.867359	1.960185	3.244560	L
H-HC-0.0800	0	-8.139317	2.763484	3.347388	L
H-HC-0.0800	0	-9.670435	2.291672	2.586564	L
H-HC-0.0800	0	-9.284769	1.719534	4.222802	L
C-CT-0.2000	0	-8.220650	0.744483	2.659793	L
H-H1-0.0800	0	-8.981817	-0.026543	2.511107	L
O-OS--0.5600	0	-7.604473	1.085234	1.436728	L
C-CT-0.2000	0	-7.099561	0.218476	3.548611	L

H-H1-0.0800	0	-6.414811	1.018724	3.830669	L
O-OH--0.6800	0	-7.573342	-0.461410	4.704283	L
H-HO-0.4000	0	-8.214762	-1.123782	4.404366	L
C-CT-0.2000	0	-6.448989	-0.674015	2.502006	L
H-H1-0.0800	0	-5.423672	-0.933946	2.772942	L
O-OH--0.6800	0	-7.268450	-1.830546	2.374553	L
H-HO-0.4000	0	-6.766029	-2.487684	1.853552	L
C-CT-0.5691	0	-6.508793	0.184052	1.229075	H
H-H2-0.8000	0	-6.675209	-0.489814	0.366197	H
N-N*--0.5691	0	-5.304860	0.986503	1.022680	H
C-CM--0.0500	0	-4.683668	0.797263	-0.174126	H
H-H4-0.1500	0	-5.032605	-0.067471	-0.691645	H
C-CM--0.1238	0	-3.763750	1.604626	-0.705130	H
C-C-0.6156	0	-3.493353	1.586711	-2.155070	H
O-O--0.5700	0	-2.703751	2.345779	-2.662845	H
N-N--0.8000	0	-4.269131	0.782620	-2.945269	H
H-H-0.3700	0	-3.936331	0.562307	-3.862441	H
H-H-0.3700	0	-4.871696	0.093686	-2.546791	H
C-CT-0.1164	0	-3.119410	2.640964	0.183157	H
H-HC-0.0800	0	-2.769527	3.481150	-0.398185	H
H-HC-0.0800	0	-2.236868	2.230946	0.671752	H
C-CM--0.2882	0	-4.127935	3.051609	1.228604	H
H-HA-0.1500	0	-4.036188	4.024287	1.670244	H
C-CM--0.0500	0	-5.097165	2.243209	1.617262	H
H-H4-0.1500	0	-5.790442	2.529189	2.378239	H
N-N3--0.8530	0	3.414261	0.989883	-0.321730	H
H-H-0.4500	0	3.271692	0.668698	-1.266066	H
H-H-0.4500	0	2.743773	0.613254	0.347791	H
H-H-0.4500	0	4.339804	0.742556	-0.005067	H
C-CT-0.3170	0	3.416628	2.463140	-0.269766	H
H-HP-0.0800	0	4.206908	2.799525	-0.923923	H
C-CT--0.1600	0	2.080796	3.097131	-0.644589	H
H-HC-0.0800	0	1.904854	2.950686	-1.702588	H
H-HC-0.0800	0	2.222211	4.160731	-0.499707	H
C-CT--0.1600	0	0.899178	2.590456	0.193310	H
H-HC-0.0800	0	0.132163	3.350730	0.194400	H
H-HC-0.0800	0	1.231962	2.497744	1.220995	H
C-CT--0.0990	0	0.303393	1.258386	-0.300506	H
H-HC-0.0800	0	0.931529	0.824200	-1.074376	H
H-HC-0.0800	0	-0.641496	1.473796	-0.774620	H
C-C-0.4490	0	0.122591	0.199870	0.781656	H
H-H-0.0600	0	-0.338026	0.674424	1.648281	H
O-O--0.5700	0	1.400166	-0.256503	1.167672	H
N-N3--0.9900	0	-0.680450	-0.917389	0.335916	H
H-H-0.3600	0	1.192283	-1.065965	1.670937	H
H-H-0.3600	0	-1.100110	-0.658927	-0.547476	H
C-C-0.9060	0	3.833861	2.790342	1.156871	L
O-O2--0.9000	0	3.956467	1.892117	2.023321	L
O-O2--0.9000	0	4.045956	3.976495	1.470686	L
C-CT--0.205900	0	-1.680909	-1.435398	1.268408	H
H-H1-0.139900	0	-2.282208	-0.621254	1.681389	H
C-CT-0.007100	0	-2.614418	-2.328658	0.442626	H
H-HC--0.007800	0	-2.018293	-3.008237	-0.150794	H
H-HC--0.007800	0	-3.148677	-1.690513	-0.259065	H
C-CT-0.067500	0	-3.641242	-3.142567	1.224705	L

H-HC--0.054800 0 -3.909278 -2.592377 2.128577 L
H-HC--0.054800 0 -3.202824 -4.100420 1.512770 L
C-C-0.818300 0 -4.932704 -3.355749 0.433501 L
O-O2--0.822000 0 -5.732804 -2.388376 0.357270 L
O-O2--0.822000 0 -5.236244 -4.458216 -0.072922 L
C-C-0.742000 0 -0.978816 -2.143541 2.444111 H
O-O2--0.793000 0 -1.645875 -2.589966 3.364435 H
O-O2--0.793000 0 0.258614 -2.258294 2.336436 H
O-OW--0.834000 0 2.718866 -5.711435 -3.525042 L
H-HW-0.417000 0 3.572259 -5.856351 -3.068005 L
H-HW-0.417000 0 2.122647 -5.458347 -2.803576 L
O-OW--0.834000 0 0.224311 -7.427283 4.594658 L
H-HW-0.417000 0 0.892884 -7.070030 5.212548 L
H-HW-0.417000 0 -0.340865 -7.952401 5.167553 L
O-OW--0.834000 0 4.244965 -0.601291 2.457941 L
H-HW-0.417000 0 3.340610 -0.895857 2.314128 L
H-HW-0.417000 0 4.172429 0.378637 2.351372 L
O-OW--0.834000 0 6.116872 -1.626243 4.107713 L
H-HW-0.417000 0 5.795816 -2.522197 4.306228 L
H-HW-0.417000 0 5.443348 -1.293118 3.478098 L
O-OW--0.834000 0 -6.185656 -6.967749 0.029830 L
H-HW-0.417000 0 -5.869591 -6.042333 0.031833 L
H-HW-0.417000 0 -6.705903 -7.048469 -0.777972 L
O-OW--0.834000 0 -9.024740 -0.832001 -0.142382 L
H-HW-0.417000 0 -9.921523 -0.807387 0.202011 L
H-HW-0.417000 0 -8.838251 -1.797167 -0.188069 L

TS4
C-CT--0.3662 -1 -12.884138 1.046748 1.881253 L
H-HC-0.1123 0 -12.386195 1.976791 1.611368 L
H-HC-0.1123 0 -13.956219 1.158481 1.728070 L
H-HC-0.1123 0 -12.497720 0.235490 1.267814 L
C-C-0.5972 0 -12.611909 0.744565 3.342509 L
O-O--0.5679 0 -11.839841 1.459881 3.970800 L
N-N--0.415700 0 -13.220718 -0.322862 3.869028 L
H-H-0.271900 0 -13.827149 -0.846404 3.259085 L
C-CT--0.025200 -1 -12.929838 -0.876155 5.204971 L
H-H1-0.069800 0 -13.680782 -1.625188 5.455553 L
H-H1-0.069800 0 -12.970412 -0.074382 5.942950 L
C-C-0.597300 0 -11.521520 -1.538369 5.273210 L
O-O--0.567900 0 -10.714404 -1.433625 4.344289 L
N-N--0.415700 0 -11.210949 -2.253507 6.365841 L
H-H-0.271900 0 -11.911028 -2.334623 7.086036 L
C-CT--0.025200 -1 -10.022963 -3.131920 6.459010 L
H-H1-0.069800 0 -9.940266 -3.531112 7.469562 L
H-H1-0.069800 0 -9.124370 -2.557958 6.228363 L
C-C-0.597300 0 -10.160003 -4.296690 5.488579 L
O-O--0.567900 0 -11.200194 -4.946908 5.431800 L
N-N--0.4157 0 -9.100506 -4.555595 4.734190 L
H-H-0.2719 0 -8.276073 -3.994356 4.879170 L
C-CT--0.0014 -1 -9.023847 -5.509453 3.627482 L
H-H1-0.0876 0 -9.864372 -5.361706 2.948526 L
C-C-0.5973 0 -9.009137 -6.996543 4.042077 L
O-O--0.5679 0 -8.392216 -7.831930 3.383329 L
C-CT--0.0152 0 -7.720186 -5.163209 2.890997 L

H-HC-0.0295	0	-7.889842	-4.197723	2.435115	L
H-HC-0.0295	0	-7.566547	-5.858302	2.063998	L
C-CA--0.0011	0	-6.423425	-5.061913	3.701941	L
C-CA--0.1906	0	-5.630939	-6.199019	3.963121	L
H-HA-0.1699	0	-6.004428	-7.186761	3.719756	L
C-CA--0.1906	0	-5.928359	-3.793604	4.066900	L
H-HA-0.1699	0	-6.531758	-2.915224	3.949505	L
C-CA--0.2341	0	-4.310432	-6.046479	4.444357	L
H-HA-0.1656	0	-3.668289	-6.902370	4.589358	L
C-CA--0.2341	0	-4.602920	-3.632181	4.503636	L
H-HA-0.1656	0	-4.188806	-2.649277	4.673001	L
C-C-0.3326	0	-3.770581	-4.755862	4.640798	L
O-OH--0.5579	0	-2.448924	-4.580557	4.893674	L
H-HO-0.3992	0	-2.136896	-3.757030	4.458262	L
N-N--0.4157	0	-9.670481	-7.345465	5.144500	L
H-H-0.2719	0	-10.256930	-6.633760	5.562989	L
C-CT--0.1490	-1	-9.606195	-8.674007	5.723735	L
H-H1-0.0976	0	-8.574476	-8.913331	5.987998	L
H-H1-0.0976	0	-10.230082	-8.725356	6.616870	L
H-H1-0.0976	0	-9.959314	-9.408878	4.997167	L
C-CT--0.3662	-1	-11.990471	-1.508791	-3.495932	L
H-HC-0.1123	0	-12.495891	-0.551253	-3.608299	L
H-HC-0.1123	0	-11.990425	-1.823370	-2.454734	L
H-HC-0.1123	0	-12.502186	-2.256962	-4.100672	L
C-C-0.5972	0	-10.563691	-1.383105	-3.987831	L
O-O--0.5679	0	-10.141731	-2.092743	-4.890523	L
N-N--0.415700	0	-9.791884	-0.501810	-3.361607	L
H-H-0.271900	0	-10.186813	0.000639	-2.582205	L
C-CT--0.038900	-1	-8.392044	-0.160855	-3.703126	L
H-H1-0.100700	0	-8.290009	-0.094017	-4.784316	L
C-C-0.597300	0	-7.394545	-1.206363	-3.188732	L
O-O--0.567900	0	-6.250662	-0.895606	-2.867734	L
C-CT-0.365400	0	-8.070731	1.213482	-3.096445	L
H-H1-0.004300	0	-7.087621	1.522216	-3.433050	L
O-OH--0.676100	0	-8.064832	1.173059	-1.685306	L
H-HO-0.410200	0	-7.942284	2.083364	-1.392910	L
C-CT--0.243800	0	-9.095289	2.281792	-3.480874	L
H-HC-0.064200	0	-10.094530	2.026804	-3.132326	L
H-HC-0.064200	0	-9.114253	2.406824	-4.562734	L
H-HC-0.064200	0	-8.825057	3.228849	-3.030883	L
N-N--0.415700	0	-7.833764	-2.453433	-3.057232	L
H-H-0.271900	0	-8.713330	-2.669127	-3.510105	L
C-CT--0.024900	-1	-7.190189	-3.492259	-2.255680	L
H-H1-0.084300	0	-6.349762	-3.082830	-1.727128	L
C-C-0.597300	0	-6.635845	-4.621466	-3.117111	L
O-O--0.567900	0	-7.040410	-4.815353	-4.260178	L
C-CT-0.211700	0	-8.220373	-4.149944	-1.325657	L
H-H1-0.035200	0	-8.155468	-5.239049	-1.373374	L
H-H1-0.035200	0	-9.233748	-3.858972	-1.606732	L
O-OH--0.654600	0	-7.953091	-3.754238	-0.010429	L
H-HO-0.427500	0	-7.013599	-4.018136	0.149057	L
N-N--0.4157	0	-5.750086	-5.423152	-2.531709	L
H-H-0.2719	0	-5.491370	-5.209988	-1.572638	L
C-CT--0.0014	0	-5.324741	-6.691517	-3.120329	L
H-H1-0.0876	0	-4.924033	-6.494185	-4.114987	L

C-C-0.5973	0	-6.462664	-7.712196	-3.264446	L
O-O--0.5679	0	-7.438096	-7.699419	-2.515695	L
C-CT--0.0152	0	-4.225958	-7.298216	-2.254953	L
H-HC-0.0295	0	-4.051167	-8.329399	-2.564892	L
H-HC-0.0295	0	-4.558331	-7.329227	-1.215658	L
C-CA--0.0011	-1	-2.914039	-6.565318	-2.351135	L
C-CA--0.1906	0	-2.015135	-6.853881	-3.400747	L
H-HA-0.1699	0	-2.301631	-7.547284	-4.178480	L
C-CA--0.1906	0	-2.513374	-5.674618	-1.336799	L
H-HA-0.1699	0	-3.186219	-5.433985	-0.533327	L
C-CA--0.2341	0	-0.731265	-6.267250	-3.424212	L
H-HA-0.1656	0	-0.037688	-6.487950	-4.221017	L
C-CA--0.2341	0	-1.224332	-5.111572	-1.335491	L
H-HA-0.1656	0	-0.918617	-4.464486	-0.526532	L
C-C-0.3226	0	-0.332483	-5.399904	-2.384603	L
O-OH--0.5579	0	0.906394	-4.840287	-2.381873	L
H-HO-0.3992	0	0.909603	-4.102478	-1.762502	L
N-N--0.4157	0	-6.273446	-8.652342	-4.197668	L
H-H-0.2719	0	-5.454052	-8.563330	-4.775086	L
C-CT--0.1490	-1	-7.127897	-9.815816	-4.380834	L
H-H1-0.0976	0	-8.139980	-9.489433	-4.629973	L
H-H1-0.0976	0	-6.745759	-10.446917	-5.184043	L
H-H1-0.0976	0	-7.169711	-10.392485	-3.454509	L
C-CT--0.3662	-1	-6.304077	-3.594764	-6.936162	L
H-HC-0.1123	0	-5.816823	-4.366644	-7.527789	L
H-HC-0.1123	0	-7.343110	-3.871978	-6.763203	L
H-HC-0.1123	0	-5.790311	-3.481198	-5.987422	L
C-C-0.5972	0	-6.234302	-2.272844	-7.684424	L
O-O--0.5679	0	-5.339624	-2.108903	-8.503103	L
N-N--0.415700	0	-7.147990	-1.334763	-7.400003	L
H-H-0.271900	0	-7.862536	-1.598873	-6.737458	L
C-CT--0.025200	-1	-7.251498	0.001471	-8.035536	L
H-H1-0.069800	0	-8.174955	0.045233	-8.612991	L
H-H1-0.069800	0	-6.424561	0.165575	-8.725023	L
C-C-0.597300	0	-7.278423	1.143582	-7.013130	L
O-O--0.567900	0	-8.271687	1.854440	-6.921005	L
N-N--0.415700	0	-6.200064	1.308272	-6.241519	L
H-H-0.271900	0	-5.459220	0.621391	-6.334081	L
C-CT--0.025200	-1	-5.801410	2.563862	-5.591738	L
H-H1-0.069800	0	-5.621003	2.407875	-4.534280	L
H-H1-0.069800	0	-6.580511	3.318005	-5.713616	L
C-C-0.597300	0	-4.518701	3.087653	-6.247134	L
O-O--0.567900	0	-4.462007	3.226703	-7.466358	L
N-N--0.415700	0	-3.472468	3.311468	-5.448395	L
H-H-0.271900	0	-3.593525	3.147700	-4.459572	L
C-CT--0.051800	-1	-2.132337	3.694768	-5.907877	L
H-H1-0.092200	0	-2.211126	4.123692	-6.908246	L
C-C-0.597300	0	-1.216248	2.458137	-6.002577	L
O-O--0.567900	0	-0.536428	2.229470	-7.000222	L
C-CT--0.110200	0	-1.593757	4.775458	-4.949994	L
H-HC-0.045700	0	-1.573964	4.371567	-3.938331	L
H-HC-0.045700	0	-2.288514	5.616752	-4.956515	L
C-CT-0.353100	0	-0.186920	5.298929	-5.283127	L
H-HC--0.036100	0	0.523968	4.472693	-5.268557	L
C-CT--0.412100	0	-0.122988	5.986715	-6.646792	L

H-HC-0.100000	0	-0.870421	6.779173	-6.700860	L
H-HC-0.100000	0	-0.315340	5.260659	-7.436672	L
H-HC-0.100000	0	0.868242	6.410139	-6.804519	L
C-CT--0.412100	0	0.233040	6.311471	-4.215421	L
H-HC-0.100000	0	-0.470586	7.144630	-4.197480	L
H-HC-0.100000	0	1.231003	6.689232	-4.435948	L
H-HC-0.100000	0	0.245044	5.827755	-3.239785	L
N-N--0.516300	0	-1.172751	1.667395	-4.932457	L
H-H-0.293600	0	-1.719394	1.974412	-4.133999	L
C-CT-0.038100	-1	-0.281904	0.529754	-4.714213	L
H-H1-0.088000	0	-0.053095	0.032203	-5.654501	L
C-C-0.536600	0	-0.934225	-0.467386	-3.723800	L
O-O--0.581900	0	-0.718341	-0.364700	-2.519053	L
C-CT--0.030300	0	1.032087	1.127869	-4.130976	L
H-HC--0.012200	0	0.776045	1.783410	-3.298935	L
H-HC--0.012200	0	1.499324	1.748681	-4.897379	L
C-C-0.799400	-1	2.077846	0.112250	-3.646765	L
O-O2--0.801400	0	2.034041	-1.044540	-4.128418	L
O-O2--0.801400	0	3.015994	0.476209	-2.908596	L
N-N--0.254800	0	-1.750484	-1.430372	-4.184421	L
C-CT--0.026600	-1	-2.025514	-1.753934	-5.579622	L
H-H1-0.064100	0	-1.093163	-1.835213	-6.137058	L
C-C-0.589600	0	-2.939129	-0.729899	-6.275525	L
O-O--0.574800	0	-3.821737	-0.151526	-5.647336	L
C-CT--0.007000	0	-2.688551	-3.134703	-5.528632	L
H-HC-0.025300	0	-1.917595	-3.906677	-5.505048	L
H-HC-0.025300	0	-3.363304	-3.302413	-6.368588	L
C-CT-0.018900	0	-3.428537	-3.122490	-4.192035	L
H-HC-0.021300	0	-3.587453	-4.129146	-3.809924	L
H-HC-0.021300	0	-4.375630	-2.590931	-4.291792	L
C-CT-0.019200	0	-2.487146	-2.321521	-3.298914	L
H-H1-0.039100	0	-3.056473	-1.760034	-2.556911	L
H-H1-0.039100	0	-1.785288	-2.987893	-2.801925	L
N-N--0.4157	0	-2.732653	-0.515067	-7.577617	L
H-H-0.2719	0	-2.009760	-1.044195	-8.033717	L
C-CT--0.1490	-1	-3.503627	0.407594	-8.410617	L
H-H1-0.0976	0	-4.463942	0.626983	-7.959790	L
H-H1-0.0976	0	-3.680400	-0.034699	-9.391988	L
H-H1-0.0976	0	-2.956529	1.345345	-8.531167	L
C-CT--0.3662	-1	8.772689	1.829867	-5.878978	L
H-HC-0.1123	0	9.268989	2.067804	-6.819471	L
H-HC-0.1123	0	9.522970	1.625553	-5.117981	L
H-HC-0.1123	0	8.126667	0.965611	-6.019439	L
C-C-0.5972	0	7.943104	3.020474	-5.452857	L
O-O--0.5679	0	8.076354	4.091230	-6.026641	L
N-N--0.415700	0	7.087723	2.842143	-4.445930	L
H-H-0.271900	0	7.028137	1.919285	-4.039391	L
C-CT-0.021300	-1	6.095852	3.842497	-4.026138	L
H-H1-0.112400	0	6.286992	4.787749	-4.538583	L
C-C-0.597300	0	6.130918	4.105265	-2.518650	L
O-O--0.567900	0	6.394983	3.200032	-1.727031	L
C-CT--0.123100	0	4.720067	3.320650	-4.474531	L
H-H1-0.111200	0	4.500305	2.375607	-3.970884	L
H-H1-0.111200	0	4.737571	3.146742	-5.552181	L
S-SH--0.311900	0	3.414563	4.522906	-4.095093	L

H-HS-0.193300	0	2.404463	3.863657	-4.681339	L
N-N--0.415700	0	5.843294	5.341185	-2.113084	L
H-H-0.271900	0	5.590163	6.023948	-2.814700	L
C-CT--0.025200	-1	5.823244	5.764055	-0.717955	L
H-H1-0.069800	0	6.788373	6.206563	-0.471953	L
H-H1-0.069800	0	5.678530	4.908291	-0.062724	L
C-C-0.597300	0	4.747727	6.810076	-0.449206	L
O-O--0.567900	0	4.860211	7.950826	-0.888469	L
N-N--0.415700	0	3.729875	6.440827	0.322422	L
H-H-0.271900	0	3.773962	5.502171	0.710764	L
C-CT--0.025200	-1	2.684363	7.314228	0.839819	L
H-H1-0.069800	0	1.852151	6.698741	1.169074	L
H-H1-0.069800	0	2.344249	8.000633	0.063807	L
C-C-0.597300	0	3.173828	8.116451	2.043662	L
O-O--0.567900	0	2.585946	8.030123	3.119517	L
N-N--0.4157	0	4.265215	8.865834	1.854121	L
H-H-0.2719	0	4.631180	8.864735	0.907020	L
C-CT--0.1490	-1	5.004047	9.564413	2.896723	L
H-H1-0.0976	0	5.327062	8.854213	3.659802	L
H-H1-0.0976	0	5.876659	10.060557	2.469265	L
H-H1-0.0976	0	4.360037	10.311651	3.364534	L
C-CT--0.3662	-1	-1.532409	9.491396	5.186391	L
H-HC-0.1123	0	-1.739840	10.450457	4.714561	L
H-HC-0.1123	0	-0.592507	9.093412	4.807425	L
H-HC-0.1123	0	-1.474479	9.625205	6.265115	L
C-C-0.5972	0	-2.667855	8.517982	4.857689	L
O-O--0.5679	0	-3.554791	8.867348	4.088931	L
N-N--0.4157	0	-2.639857	7.296765	5.407114	L
H-H-0.2719	0	-1.890410	7.088958	6.052777	L
C-CT--0.0275	-1	-3.628744	6.200619	5.235364	L
H-H1-0.1123	0	-4.626964	6.631921	5.155033	L
C-C-0.5973	0	-3.605550	5.248243	6.460345	L
O-O--0.5679	0	-2.703497	5.331288	7.292473	L
C-CT--0.0050	0	-3.320421	5.402279	3.959697	L
H-HC-0.0339	0	-3.519348	6.036134	3.102418	L
H-HC-0.0339	0	-4.038255	4.585592	3.898315	L
C-C*--0.1415	0	-1.939537	4.815188	3.822601	L
C-CW--0.1638	0	-0.795649	5.505978	3.594930	L
H-H4-0.2062	0	-0.725665	6.580826	3.492741	L
C-CB-0.1243	0	-1.539514	3.411068	3.901035	L
N-NA--0.3418	0	0.275139	4.635287	3.524779	L
H-H-0.3412	0	1.235955	4.924774	3.389465	L
C-CN-0.1380	0	-0.126150	3.331387	3.710038	L
C-CA--0.238	0	-2.226269	2.196327	4.123259	L
H-HA-0.1700	0	-3.296428	2.210381	4.250982	L
C-CA--0.2601	0	0.572111	2.116219	3.742506	L
H-HA-0.1572	0	1.639726	2.088810	3.578248	L
C-CA--0.1972	0	-1.534941	0.970453	4.160899	L
H-HA-0.1447	0	-2.067407	0.038810	4.305141	L
C-CA--0.1134	0	-0.143505	0.929977	3.971079	L
H-HA-0.1417	0	0.365248	-0.023813	3.969900	L
N-N--0.4157	0	-4.582771	4.330208	6.572311	L
H-H-0.2719	0	-5.287509	4.338504	5.853524	L
C-CT--0.1490	-1	-4.683807	3.318666	7.638930	L
H-H1-0.0976	0	-3.855130	2.613874	7.553500	L

H-H1-0.0976	0	-4.626444	3.807355	8.614167	L
H-H1-0.0976	0	-5.628863	2.778961	7.563222	L
C-CT--0.3662	-1	-2.835752	-1.405129	7.298936	L
H-HC-0.1123	0	-3.067235	-2.448546	7.083215	L
H-HC-0.1123	0	-3.178484	-1.157568	8.301058	L
H-HC-0.1123	0	-3.323964	-0.774263	6.558662	L
C-C-0.5972	0	-1.343098	-1.212770	7.206005	L
O-O--0.5679	0	-0.625855	-2.163764	6.949226	L
N-N--0.415700	0	-0.848204	-0.007116	7.481819	L
H-H-0.271900	0	-1.506469	0.744952	7.609111	L
C-CT--0.087500	-1	0.594774	0.320317	7.437384	L
H-H1-0.096900	0	0.926268	0.199474	6.405973	L
C-C-0.597300	0	1.440324	-0.627756	8.299457	L
O-O--0.567900	0	2.453722	-1.141739	7.839730	L
C-CT-0.298500	0	0.874573	1.780801	7.867159	L
H-HC--0.029700	0	0.919044	1.828019	8.955906	L
C-CT--0.319200	0	2.226020	2.245308	7.310154	L
H-HC-0.079100	0	2.199828	2.241172	6.220044	L
H-HC-0.079100	0	3.021339	1.583126	7.652845	L
H-HC-0.079100	0	2.441695	3.255719	7.658140	L
C-CT--0.319200	0	-0.198166	2.782710	7.419505	L
H-HC-0.079100	0	-0.367329	2.693025	6.350556	L
H-HC-0.079100	0	0.121650	3.801569	7.640494	L
H-HC-0.079100	0	-1.133562	2.609959	7.949470	L
N-N--0.415700	0	1.009507	-0.888963	9.541337	L
H-H-0.271900	0	0.169878	-0.419366	9.838393	L
C-CT--0.025200	-1	1.621449	-1.876704	10.445544	L
H-H1-0.069800	0	1.086380	-1.887293	11.394439	L
H-H1-0.069800	0	2.656355	-1.592072	10.639003	L
C-C-0.597300	0	1.625372	-3.316512	9.878985	L
O-O--0.567900	0	2.445329	-4.120481	10.304786	L
N-N--0.415700	0	0.723793	-3.648442	8.941178	L
H-H-0.271900	0	0.126478	-2.918973	8.579434	L
C-CT-0.033700	-1	0.625672	-4.940490	8.246965	L
H-H1-0.082300	0	1.032339	-5.710658	8.905232	L
C-C-0.597300	0	1.462554	-5.005118	6.944462	L
O-O--0.567900	0	2.105717	-6.014933	6.670726	L
C-CT--0.182500	0	-0.850028	-5.284329	8.007469	L
H-HC-0.060300	0	-1.389432	-5.284804	8.954769	L
H-HC-0.060300	0	-0.921696	-6.276271	7.559713	L
H-HC-0.060300	0	-1.307522	-4.565274	7.331205	L
N-N--0.415700	0	1.491076	-3.924104	6.158407	L
H-H-0.271900	0	0.848950	-3.178237	6.400093	L
C-CT-0.033700	-1	2.435573	-3.669975	5.057421	L
H-H1-0.082300	0	2.317752	-4.435704	4.295052	L
C-C-0.597300	0	3.907941	-3.671393	5.529824	L
O-O--0.567900	0	4.822542	-3.895680	4.733828	L
C-CT--0.182500	0	2.082285	-2.296057	4.496991	L
H-HC-0.060300	0	2.499580	-1.508052	5.124414	L
H-HC-0.060300	0	1.000361	-2.180109	4.474667	L
H-HC-0.060300	0	2.483753	-2.214374	3.491312	L
N-N--0.4157	0	4.136954	-3.349435	6.810295	L
H-H-0.2719	0	3.325518	-3.047828	7.334178	L
C-CT--0.1490	-1	5.411404	-3.409586	7.510855	L
H-H1-0.0976	0	5.898492	-4.364631	7.304603	L

H-H1-0.0976	0	5.245542	-3.319364	8.585672	L
H-H1-0.0976	0	6.057849	-2.598955	7.171592	L
C-CT--0.3662	-1	14.089677	0.913795	-0.621390	L
H-HC-0.1123	0	13.437598	1.428127	0.081675	L
H-HC-0.1123	0	15.095040	1.328349	-0.563692	L
H-HC-0.1123	0	13.706174	1.021690	-1.633799	L
C-C-0.5972	0	14.145470	-0.563111	-0.268136	L
O-O--0.5679	0	15.226554	-1.123440	-0.150492	L
N-N--0.4157	0	12.984223	-1.199058	-0.116908	L
H-H-0.2719	0	12.141739	-0.650541	-0.243697	L
C-CT--0.0014	-1	12.807573	-2.649838	0.102788	L
H-H1-0.0876	0	13.690356	-3.171932	-0.268976	L
C-C-0.5973	0	11.585577	-3.153304	-0.717865	L
O-O--0.5679	0	10.828890	-2.358367	-1.284565	L
C-CT--0.0152	0	12.672179	-2.944374	1.608012	L
H-HC-0.0295	0	13.613204	-2.680805	2.093360	L
H-HC-0.0295	0	12.533933	-4.016847	1.749458	L
C-CA--0.0011	-1	11.542908	-2.207892	2.308698	L
C-CA--0.1906	0	11.786616	-0.960281	2.913633	L
H-HA-0.1699	0	12.784481	-0.543515	2.898830	L
C-CA--0.1906	0	10.242476	-2.750648	2.328552	L
H-HA-0.1699	0	10.045304	-3.712967	1.877178	L
C-CA--0.2341	0	10.735700	-0.257783	3.532976	L
H-HA-0.1656	0	10.926590	0.687826	4.006897	L
C-CA--0.2341	0	9.187349	-2.046089	2.940337	L
H-HA-0.1656	0	8.194551	-2.470161	2.952391	L
C-C-0.3226	0	9.430054	-0.794450	3.546865	L
O-OH--0.557	0	8.416947	-0.105098	4.139811	L
H-HO-0.3992	0	7.595869	-0.638921	4.126673	L
N-N--0.415700	0	11.376424	-4.472710	-0.822361	L
H-H-0.271900	0	11.994094	-5.087147	-0.315209	L
C-CT--0.025200	-1	10.257300	-5.077091	-1.574845	L
H-H1-0.069800	0	10.473001	-6.130976	-1.749564	L
H-H1-0.069800	0	10.156215	-4.589502	-2.543643	L
C-C-0.597300	0	8.929249	-4.993127	-0.809115	L
O-O--0.567900	0	8.859399	-5.385653	0.353998	L
N-N--0.415700	0	7.862581	-4.505386	-1.448610	L
H-H-0.271900	0	7.948218	-4.382087	-2.450064	L
C-CT-0.014300	-1	6.520643	-4.481221	-0.868916	L
H-H1-0.104800	0	6.607561	-4.373254	0.215269	L
C-C-0.597300	0	5.790484	-5.797199	-1.141598	L
O-O--0.567900	0	5.086980	-5.941771	-2.146864	L
C-CT--0.204100	0	5.706582	-3.262988	-1.368515	L
H-HC-0.079700	0	5.573045	-3.331712	-2.447758	L
H-HC-0.079700	0	6.279842	-2.373297	-1.146304	L
C-C-0.713000	-1	4.323459	-3.119099	-0.711110	L
O-O--0.593100	0	3.951028	-3.845742	0.197061	L
N-N--0.919100	0	3.488005	-2.222173	-1.193577	L
H-H-0.419600	0	2.608055	-2.147338	-0.711736	L
H-H-0.419600	0	3.686793	-1.630174	-1.991300	L
N-N--0.347900	0	5.932870	-6.730731	-0.198049	L
H-H-0.274700	0	6.591850	-6.495691	0.534349	L
C-CT--0.263700	-1	5.373873	-8.087821	-0.244186	L
H-H1-0.156000	0	5.745623	-8.619080	0.632225	L
C-C-0.734100	0	5.883956	-8.899075	-1.445817	L

O-O--0.589400	0	6.898034	-8.569641	-2.054345	L
C-CT--0.000700	0	3.834125	-8.042768	-0.144959	L
H-HC-0.032700	0	3.444018	-9.060438	-0.106520	L
H-HC-0.032700	0	3.433976	-7.568209	-1.042367	L
C-CT-0.039000	0	3.298855	-7.313495	1.092979	L
H-HC-0.028500	0	3.701601	-6.303812	1.152033	L
H-HC-0.028500	0	3.566940	-7.864462	1.995559	L
C-CT-0.048600	0	1.778559	-7.249828	0.947376	L
H-H1-0.068700	0	1.387145	-8.263996	0.849339	L
H-H1-0.068700	0	1.525656	-6.702769	0.035939	L
N-N2--0.529500	-1	1.120795	-6.610464	2.089222	L
H-H-0.345600	0	0.421260	-7.150469	2.580580	L
C-CA-0.807600	0	0.903811	-5.326587	2.234328	L
N-N2--0.862700	0	-0.082223	-4.937819	2.976588	L
H-H-0.447800	0	-0.760505	-5.601345	3.320616	L
H-H-0.447800	0	-0.295484	-3.938597	3.040259	L
N-N2--0.862700	0	1.613542	-4.416506	1.643340	L
H-H-0.447800	0	2.482567	-4.581424	1.154324	L
H-H-0.447800	0	1.275094	-3.467043	1.797978	L
N-N--0.4157	0	5.198783	-10.006021	-1.739127	L
H-H-0.2719	0	4.399293	-10.203378	-1.162019	L
C-CT--0.1490	-1	5.511524	-10.913202	-2.828437	L
H-H1-0.0976	0	6.522012	-11.307843	-2.701097	L
H-H1-0.0976	0	4.802745	-11.742441	-2.847742	L
H-H1-0.0976	0	5.465923	-10.377585	-3.778849	L
C-CT--0.3662	-1	10.184469	-6.029084	-7.638614	L
H-HC-0.1123	0	9.897184	-6.725473	-8.423940	L
H-HC-0.1123	0	11.090477	-5.503066	-7.938116	L
H-HC-0.1123	0	10.369735	-6.561723	-6.707366	L
C-C-0.5972	0	9.078278	-5.017627	-7.428061	L
O-O--0.5679	0	9.310988	-3.822286	-7.571883	L
N-N--0.347900	0	7.878815	-5.467129	-7.030184	L
H-H-0.274700	0	7.791808	-6.462144	-6.887764	L
C-CT--0.263700	-1	6.895214	-4.561766	-6.402771	L
H-H1-0.156000	0	6.738414	-3.703728	-7.057388	L
C-C-0.734100	0	7.519517	-4.048711	-5.102468	L
O-O--0.589400	0	7.866872	-4.842569	-4.234284	L
C-CT--0.000700	0	5.544619	-5.244071	-6.137513	L
H-HC-0.032700	0	4.945021	-4.548161	-5.553670	L
H-HC-0.032700	0	5.686351	-6.142475	-5.533066	L
C-CT-0.039000	0	4.795394	-5.606222	-7.433239	L
H-HC-0.028500	0	5.026003	-6.642293	-7.684363	L
H-HC-0.028500	0	5.142479	-4.979015	-8.255999	L
C-CT-0.048600	0	3.269647	-5.448195	-7.312208	L
H-H1-0.068700	0	2.923974	-5.896665	-6.380228	L
H-H1-0.068700	0	2.806565	-6.003012	-8.130301	L
N-N2--0.529500	0	2.849542	-4.032892	-7.419580	L
H-H-0.345600	0	2.806861	-3.643822	-8.344735	L
C-CA-0.807600	0	2.535798	-3.193699	-6.445899	L
N-N2--0.862700	0	2.499611	-3.516841	-5.191963	L
H-H-0.447800	0	2.703390	-4.456852	-4.867606	L
H-H-0.447800	0	2.282634	-2.769160	-4.528523	L
N-N2--0.862700	0	2.253295	-1.952342	-6.699352	L
H-H-0.447800	0	2.255606	-1.538764	-7.610250	L
H-H-0.447800	0	2.107778	-1.391551	-5.856232	L

N-N--0.415700	0	7.732321	-2.740515	-4.990658	L
H-H-0.271900	0	7.434098	-2.156819	-5.754693	L
C-CT--0.025200	-1	8.632624	-2.151912	-3.994602	L
H-H1-0.069800	0	9.509001	-1.763557	-4.514213	L
H-H1-0.069800	0	8.984549	-2.906245	-3.295585	L
C-C-0.597300	0	8.030206	-1.012609	-3.192294	L
O-O--0.567900	0	6.969911	-0.480648	-3.525226	L
N-N--0.415700	0	8.718493	-0.611799	-2.126754	L
H-H-0.271900	0	9.574559	-1.100664	-1.867635	L
C-CT--0.038900	-1	8.310865	0.534192	-1.319497	L
H-H1-0.100700	0	7.685064	1.156344	-1.955752	L
C-C-0.597300	0	9.448220	1.464664	-0.923449	L
O-O--0.567900	0	10.554366	1.051388	-0.580146	L
C-CT-0.365400	0	7.412371	0.111812	-0.140733	L
H-H1-0.004300	0	7.725129	-0.867462	0.224605	L
O-OH--0.676100	0	6.097071	0.039978	-0.654557	L
H-HO-0.410200	0	6.173866	-0.197044	-1.591143	L
C-CT--0.243800	0	7.328091	1.075333	1.043367	L
H-HC-0.064200	0	6.975873	2.052739	0.715620	L
H-HC-0.064200	0	8.304591	1.171260	1.516818	L
H-HC-0.064200	0	6.630798	0.676993	1.780592	L
N-N--0.415700	0	9.126925	2.756540	-1.004116	L
H-H-0.271900	0	8.161291	2.960390	-1.240901	L
C-CT--0.025200	-1	9.960815	3.889129	-0.650950	L
H-H1-0.069800	0	9.766996	4.706091	-1.345875	L
H-H1-0.069800	0	11.012699	3.609315	-0.730494	L
C-C-0.597300	0	9.655535	4.375402	0.753423	L
O-O--0.567900	0	8.504133	4.572501	1.155577	L
N-N--0.347900	0	10.727247	4.602704	1.497167	L
H-H-0.274700	0	11.635173	4.455216	1.070963	L
C-CT--0.263700	-1	10.731610	5.207695	2.819799	L
H-H1-0.156000	0	9.825369	5.792970	2.963665	L
C-C-0.734100	0	11.900690	6.165246	2.916142	L
O-O--0.589400	0	12.848202	6.081329	2.140118	L
C-CT--0.000700	0	10.879818	4.112227	3.897929	L
H-HC-0.032700	0	11.155927	4.567011	4.850912	L
H-HC-0.032700	0	11.691852	3.440209	3.612091	L
C-CT-0.039000	0	9.613624	3.283010	4.122596	L
H-HC-0.028500	0	9.845928	2.468521	4.808127	L
H-HC-0.028500	0	9.273651	2.854392	3.179555	L
C-CT-0.048600	0	8.506746	4.127896	4.754573	L
H-H1-0.068700	0	8.317401	5.019874	4.158433	L
H-H1-0.068700	0	8.824798	4.445037	5.749468	L
N-N2--0.529500	-1	7.274153	3.347833	4.869348	L
H-H-0.345600	0	7.183330	2.730964	5.657961	L
C-CA-0.807600	0	6.278507	3.309764	4.016063	L
N-N2--0.862700	0	5.234681	2.588778	4.279846	L
H-H-0.447800	0	5.204138	1.949698	5.048320	L
H-H-0.447800	0	4.598433	2.448354	3.489725	L
N-N2--0.862700	0	6.300652	3.970663	2.901005	L
H-H-0.447800	0	7.154375	4.336570	2.504259	L
H-H-0.447800	0	5.441940	3.969883	2.351379	L
N-N--0.4157	0	11.817108	7.052822	3.897852	L
H-H-0.2719	0	11.001405	7.039993	4.486205	L
C-CT--0.1490	-1	12.879310	7.982779	4.184344	L

H-H1-0.0976	0	12.612946	8.616169	5.032067	L
H-H1-0.0976	0	13.797717	7.439701	4.419589	L
H-H1-0.0976	0	13.066789	8.614454	3.312800	L
C-CT--0.3662	-1	-2.162032	13.225505	-1.677938	L
H-HC-0.1123	0	-2.588763	13.743279	-0.821431	L
H-HC-0.1123	0	-1.075503	13.215084	-1.618549	L
H-HC-0.1123	0	-2.464687	13.735282	-2.592369	L
C-C-0.5972	0	-2.681681	11.800329	-1.723981	L
O-O--0.5679	0	-3.328911	11.416767	-2.685672	L
N-N--0.415700	0	-2.407169	11.025505	-0.670943	L
H-H-0.271900	0	-1.849606	11.451818	0.049994	L
C-CT--0.023700	-1	-2.833808	9.621895	-0.485808	L
H-H1-0.088000	0	-2.359346	9.017325	-1.260398	L
C-C-0.597300	0	-4.352620	9.442351	-0.660176	L
O-O--0.567900	0	-4.811073	8.666395	-1.499342	L
C-CT-0.034200	0	-2.311649	9.159313	0.880437	L
H-HC-0.024100	0	-2.686686	9.827150	1.657145	L
H-HC-0.024100	0	-1.223149	9.224770	0.881380	L
C-CT-0.001800	0	-2.713954	7.732998	1.241912	L
H-H1-0.044000	0	-3.785532	7.721764	1.444345	L
H-H1-0.044000	0	-2.200695	7.478882	2.164843	L
S-S--0.273700	0	-2.367348	6.440011	0.024376	L
C-CT--0.053600	0	-0.566298	6.467852	-0.062906	L
H-H1-0.068400	0	-0.235056	5.764954	-0.826900	L
H-H1-0.068400	0	-0.164614	6.168506	0.903107	L
H-H1-0.068400	0	-0.219680	7.466644	-0.325569	L
N-N--0.4157	0	-5.140155	10.174592	0.132305	L
H-H-0.2719	0	-4.686034	10.781940	0.795302	L
C-CT--0.1490	-1	-6.590154	10.198100	0.024351	L
H-H1-0.0976	0	-7.013716	10.849655	0.789987	L
H-H1-0.0976	0	-6.877632	10.569050	-0.961758	L
H-H1-0.0976	0	-6.985899	9.188899	0.151758	L
C-CT--0.3662	-1	-4.425001	8.920983	-5.236201	L
H-HC-0.1123	0	-3.988713	9.267563	-4.300084	L
H-HC-0.1123	0	-3.661040	8.911507	-6.011626	L
H-HC-0.1123	0	-5.238071	9.578376	-5.535457	L
C-C-0.5972	0	-4.962488	7.508537	-5.058791	L
O-O--0.5679	0	-4.686087	6.643992	-5.877787	L
N-N--0.415700	0	-5.739117	7.276673	-3.996796	L
H-H-0.271900	0	-5.743129	8.024486	-3.314266	L
C-CT--0.087500	-1	-6.381349	5.981255	-3.645198	L
H-H1-0.096900	0	-6.106816	5.234711	-4.390039	L
C-C-0.597300	0	-7.900212	6.103341	-3.694667	L
O-O--0.567900	0	-8.557066	5.298460	-4.342972	L
C-CT-0.298500	0	-5.867113	5.505398	-2.274549	L
H-HC--0.029700	0	-6.017222	6.291992	-1.535146	L
C-CT--0.319200	0	-6.555820	4.241636	-1.756645	L
H-HC-0.079100	0	-6.430281	3.443570	-2.484174	L
H-HC-0.079100	0	-7.610950	4.441865	-1.579671	L
H-HC-0.079100	0	-6.104953	3.938422	-0.812571	L
C-CT--0.319200	0	-4.375989	5.193788	-2.381388	L
H-HC-0.079100	0	-4.202838	4.461472	-3.163520	L
H-HC-0.079100	0	-4.024624	4.793658	-1.438314	L
H-HC-0.079100	0	-3.812308	6.098892	-2.606652	L
N-N--0.4157	0	-8.472586	7.099035	-3.010319	L

H-H-0.2719	0	-7.853430	7.730561	-2.526864	L
C-CT--0.1490	-1	-9.910682	7.347802	-3.019027	L
H-H1-0.0976	0	-10.150069	8.212214	-2.398799	L
H-H1-0.0976	0	-10.241907	7.536920	-4.042654	L
H-H1-0.0976	0	-10.439852	6.470781	-2.641891	L
C-CT-0.1200	-1	-8.882826	1.989701	3.199685	L
H-HC-0.0800	0	-8.100539	2.744439	3.252804	L
H-HC-0.0800	0	-9.719871	2.391654	2.629226	L
H-HC-0.0800	0	-9.227479	1.751402	4.206845	L
C-CT-0.2000	0	-8.389065	0.755462	2.534909	L
H-H1-0.0800	0	-9.190293	0.013651	2.471232	L
O-OS--0.5600	0	-7.927522	1.111117	1.252164	L
C-CT-0.2000	0	-7.205310	0.206581	3.317418	L
H-H1-0.0800	0	-6.503334	0.995736	3.587934	L
O-OH--0.6800	0	-7.597375	-0.528686	4.469372	L
H-HO-0.4000	0	-8.218408	-1.210095	4.169638	L
C-CT-0.2000	0	-6.630684	-0.620918	2.187482	L
H-H1-0.0800	0	-5.582037	-0.852320	2.373148	L
O-OH--0.6800	0	-7.423025	-1.801894	2.081657	L
H-HO-0.4000	0	-6.995290	-2.395759	1.433127	L
C-CT-0.5691	0	-6.799649	0.281762	0.951827	H
H-H2-0.8000	0	-6.960522	-0.369808	0.066514	H
N-N*--0.5691	0	-5.665923	1.167309	0.724702	H
C-CM--0.0500	0	-4.977908	1.000052	-0.434614	H
H-H4-0.1500	0	-5.417736	0.258356	-1.064630	H
C-CM--0.1238	0	-3.904296	1.703483	-0.798294	H
C-C-0.6156	0	-3.425398	1.690984	-2.189955	H
O-O--0.5700	0	-2.526519	2.406773	-2.562516	H
N-N--0.8000	0	-4.114009	0.941673	-3.101443	H
H-H-0.3700	0	-3.667826	0.718538	-3.968476	H
H-H-0.3700	0	-4.723986	0.201656	-2.805254	H
C-CT-0.1164	0	-3.233330	2.589358	0.224629	H
H-HC-0.0800	0	-2.765630	3.438611	-0.254379	H
H-HC-0.0800	0	-2.435559	2.046810	0.726258	H
C-CM--0.2882	0	-4.274597	3.028542	1.226242	H
H-HA-0.1500	0	-4.118025	3.951784	1.746269	H
C-CM--0.0500	0	-5.362234	2.317876	1.464180	H
H-H4-0.1500	0	-6.089268	2.628313	2.182070	H
N-N3--0.8530	0	3.380029	0.982449	-0.316921	H
H-H-0.4500	0	3.270247	0.648756	-1.261226	H
H-H-0.4500	0	2.653002	0.653753	0.327138	H
H-H-0.4500	0	4.274801	0.698991	0.054452	H
C-CT-0.3170	0	3.468797	2.452900	-0.301767	H
H-HP-0.0800	0	4.304482	2.719036	-0.930741	H
C-CT--0.1600	0	2.198627	3.162203	-0.761431	H
H-HC-0.0800	0	2.041613	2.949176	-1.811717	H
H-HC-0.0800	0	2.425611	4.218227	-0.693307	H
C-CT--0.1600	0	0.949273	2.826262	0.062466	H
H-HC-0.0800	0	0.242338	3.633846	-0.051430	H
H-HC-0.0800	0	1.229391	2.816690	1.111294	H
C-CT--0.0990	0	0.272734	1.499643	-0.329289	H
H-HC-0.0800	0	0.876610	0.978058	-1.068869	H
H-HC-0.0800	0	-0.663328	1.715678	-0.817300	H
C-C-0.4490	0	0.049360	0.524276	0.823140	H
H-H-0.0600	0	-0.288913	1.071403	1.701005	H

O-O--0.5700	0	1.306553	-0.061148	1.148076	H
N-N3--0.9900	0	-0.914381	-0.447845	0.480596	H
H-H-0.3600	0	1.063680	-0.926508	1.540337	H
H-H-0.3600	0	-1.010046	-0.599693	-0.505553	H
C-C-0.9060	0	3.854762	2.794582	1.131177	L
O-O2--0.9000	0	3.989032	1.903254	2.002187	L
O-O2--0.9000	0	4.036180	3.985324	1.447553	L
C-CT--0.205900	0	-1.753921	-1.184867	1.399853	H
H-H1-0.139900	0	-2.375700	-0.484718	1.955684	H
C-CT-0.007100	0	-2.674544	-2.030215	0.518667	H
H-HC--0.007800	0	-2.084152	-2.700688	-0.094170	H
H-HC--0.007800	0	-3.198721	-1.354635	-0.151582	H
C-CT-0.067500	0	-3.691692	-2.857701	1.285072	L
H-HC--0.054800	0	-3.985813	-2.311408	2.181589	L
H-HC--0.054800	0	-3.237438	-3.802881	1.588840	L
C-C-0.818300	0	-4.949106	-3.106432	0.459381	L
O-O2--0.822000	0	-5.646444	-2.114107	0.133278	L
O-O2--0.822000	0	-5.351365	-4.275840	0.237818	L
C-C-0.742000	0	-0.961016	-2.035245	2.411801	H
O-O2--0.793000	0	-1.560909	-2.540805	3.351767	H
O-O2--0.793000	0	0.251197	-2.197634	2.158316	H
O-OW--0.834000	0	3.034429	-5.944433	-3.845679	L
H-HW-0.417000	0	3.826880	-6.040414	-3.278304	L
H-HW-0.417000	0	2.329224	-5.768852	-3.202902	L
O-OW--0.834000	0	-1.768918	-7.332217	2.470127	L
H-HW-0.417000	0	-2.495505	-6.863772	2.011321	L
H-HW-0.417000	0	-2.134558	-8.212286	2.581148	L
O-OW--0.834000	0	4.133726	-0.597408	2.409352	L
H-HW-0.417000	0	3.200687	-0.830373	2.381117	L
H-HW-0.417000	0	4.114154	0.386957	2.315486	L
O-OW--0.834000	0	6.069473	-1.476409	4.075062	L
H-HW-0.417000	0	5.759024	-2.362894	4.332571	L
H-HW-0.417000	0	5.386074	-1.195758	3.431456	L
O-OW--0.834000	0	-4.168558	-6.360754	1.472212	L
H-HW-0.417000	0	-4.611114	-5.683504	0.919841	L
H-HW-0.417000	0	-4.596262	-6.227691	2.327485	L
O-OW--0.834000	0	-9.054194	-1.318015	-0.208179	L
H-HW-0.417000	0	-9.903929	-1.391913	0.230899	L
H-HW-0.417000	0	-8.715240	-2.237472	-0.172440	L

I4

C-CT--0.3662	-1	-12.88308200	1.04685200	1.88034200	L
H-HC-0.1123	0	-12.37888600	1.97252200	1.60702900	L
H-HC-0.1123	0	-13.95466400	1.16616400	1.72942500	L
H-HC-0.1123	0	-12.50411700	0.23140400	1.26778700	L
C-C-0.5972	0	-12.60975800	0.74614100	3.34167200	L
O-O--0.5679	0	-11.83587300	1.46095200	3.96829100	L
N-N--0.415700	0	-13.21975400	-0.31955800	3.87029300	L
H-H-0.271900	0	-13.82791700	-0.84275500	3.26176900	L
C-CT--0.025200	-1	-12.92875200	-0.87104600	5.20695100	L
H-H1-0.069800	0	-13.67978000	-1.61960800	5.45869000	L
H-H1-0.069800	0	-12.96913100	-0.06821600	5.94379200	L
C-C-0.597300	0	-11.52042600	-1.53337800	5.27603500	L
O-O--0.567900	0	-10.71319600	-1.43054800	4.34690700	L
N-N--0.415700	0	-11.20978400	-2.24657200	6.36988100	L

H-H-0.271900	0	-11.90964200	-2.32614000	7.09046600	L
C-CT--0.025200	-1	-10.02184300	-3.12487600	6.46438500	L
H-H1-0.069800	0	-9.93923100	-3.52255300	7.47553800	L
H-H1-0.069800	0	-9.12316200	-2.55134200	6.23299300	L
C-C-0.597300	0	-10.15889900	-4.29113100	5.49578700	L
O-O--0.567900	0	-11.19853600	-4.94246100	5.44124900	L
N-N--0.4157	0	-9.10004000	-4.54984700	4.74045600	L
H-H-0.2719	0	-8.27660300	-3.98642800	4.88282300	L
C-CT--0.0014	-1	-9.02269000	-5.50665300	3.63643900	L
H-H1-0.0876	0	-9.86357700	-5.36217400	2.95722700	L
C-C-0.5973	0	-9.00484000	-6.99256300	4.05549800	L
O-O--0.5679	0	-8.38100300	-7.82777300	3.40295700	L
C-CT--0.0152	0	-7.71907800	-5.16142000	2.89870500	L
H-HC-0.0295	0	-7.89130300	-4.19907400	2.43819400	L
H-HC-0.0295	0	-7.56400500	-5.85967400	2.07464000	L
C-CA--0.0011	0	-6.42153700	-5.05244000	3.70808700	L
C-CA--0.1906	0	-5.62307400	-6.18520400	3.96992200	L
H-HA-0.1699	0	-5.99271500	-7.17499000	3.72891000	L
C-CA--0.1906	0	-5.93157700	-3.78126300	4.07032700	L
H-HA-0.1699	0	-6.53938100	-2.90600000	3.95119500	L
C-CA--0.2341	0	-4.30263800	-6.02564400	4.44939900	L
H-HA-0.1656	0	-3.65666600	-6.87849300	4.59537500	L
C-CA--0.2341	0	-4.60650800	-3.61293900	4.50640700	L
H-HA-0.1656	0	-4.19792700	-2.62774400	4.67533800	L
C-C-0.3326	0	-3.76819100	-4.73230000	4.64330200	L
O-OH--0.5579	0	-2.44665200	-4.55148500	4.89483500	L
H-HO-0.3992	0	-2.13074800	-3.73251300	4.45538000	L
N-N--0.4157	0	-9.67105000	-7.34093500	5.15509800	L
H-H-0.2719	0	-10.26147100	-6.62999900	5.56944600	L
C-CT--0.1490	-1	-9.60498800	-8.66805700	5.73745300	L
H-H1-0.0976	0	-8.57441800	-8.90156200	6.01118800	L
H-H1-0.0976	0	-10.23641200	-8.72088200	6.62519400	L
H-H1-0.0976	0	-9.94803600	-9.40615700	5.00932400	L
C-CT--0.3662	-1	-11.98937400	-1.51676500	-3.49299000	L
H-HC-0.1123	0	-12.49639900	-0.56039100	-3.60797100	L
H-HC-0.1123	0	-11.98794500	-1.82792600	-2.45076000	L
H-HC-0.1123	0	-12.50025300	-2.26769700	-4.09498900	L
C-C-0.5972	0	-10.56324900	-1.39004600	-3.98629200	L
O-O--0.5679	0	-10.14193000	-2.09954900	-4.88938600	L
N-N--0.415700	0	-9.79114300	-0.50860800	-3.36061100	L
H-H-0.271900	0	-10.18473700	-0.00731300	-2.57972900	L
C-CT--0.038900	-1	-8.39096800	-0.16908600	-3.70221100	L
H-H1-0.100700	0	-8.28918800	-0.10239400	-4.78343800	L
C-C-0.597300	0	-7.39454800	-1.21588000	-3.18893200	L
O-O--0.567900	0	-6.24816400	-0.90806500	-2.87555100	L
C-CT-0.365400	0	-8.06758900	1.20480400	-3.09630800	L
H-H1-0.004300	0	-7.08301000	1.51137700	-3.43084100	L
O-OH--0.676100	0	-8.06530100	1.16446200	-1.68540500	L
H-HO-0.410200	0	-7.93640700	2.07392700	-1.39285000	L
C-CT--0.243800	0	-9.08940000	2.27446800	-3.48397300	L
H-HC-0.064200	0	-10.08989500	2.02128600	-3.13771500	L
H-HC-0.064200	0	-9.10551500	2.39872500	-4.56596300	L
H-HC-0.064200	0	-8.81870700	3.22134900	-3.03400400	L
N-N--0.415700	0	-7.83665700	-2.46082700	-3.04941700	L
H-H-0.271900	0	-8.71908900	-2.67592300	-3.49669700	L

C-CT--0.024900	-1	-7.18906200	-3.49828900	-2.24975300	L
H-H1-0.084300	0	-6.34798700	-3.08848500	-1.72263100	L
C-C-0.597300	0	-6.63464900	-4.62657800	-3.11240800	L
O-O--0.567900	0	-7.04167700	-4.82182300	-4.25430200	L
C-CT-0.211700	0	-8.21681100	-4.15989100	-1.31976400	L
H-H1-0.035200	0	-8.14569100	-5.24871300	-1.36442900	L
H-H1-0.035200	0	-9.23147700	-3.87558700	-1.60303500	L
O-OH--0.654600	0	-7.95365400	-3.75982800	-0.00536800	L
H-HO-0.427500	0	-7.01308600	-4.01869500	0.15595400	L
N-N--0.4157	0	-5.74729800	-5.42754600	-2.52821800	L
H-H-0.2719	0	-5.48805900	-5.21415600	-1.56951600	L
C-CT--0.0014	0	-5.32304300	-6.69657700	-3.11659700	L
H-H1-0.0876	0	-4.92257000	-6.50055300	-4.11162000	L
C-C-0.5973	0	-6.46172700	-7.71690000	-3.25784100	L
O-O--0.5679	0	-7.43528400	-7.70341500	-2.50664000	L
C-CT--0.0152	0	-4.22507200	-7.30461300	-2.25117900	L
H-HC-0.0295	0	-4.04964400	-8.33505700	-2.56322700	L
H-HC-0.0295	0	-4.55952300	-7.33864900	-1.21257900	L
C-CA--0.0011	-1	-2.91286500	-6.57142200	-2.34058200	L
C-CA--0.1906	0	-1.99500200	-6.88080800	-3.36741700	L
H-HA-0.1699	0	-2.26748200	-7.58909400	-4.13676800	L
C-CA--0.1906	0	-2.53041200	-5.66152100	-1.33636800	L
H-HA-0.1699	0	-3.21827400	-5.40647600	-0.55034300	L
C-CA--0.2341	0	-0.70960200	-6.29728800	-3.37666900	L
H-HA-0.1656	0	-0.00101200	-6.53538000	-4.15510900	L
C-CA--0.2341	0	-1.24107000	-5.09940800	-1.32091300	L
H-HA-0.1656	0	-0.95225800	-4.43559000	-0.51921300	L
C-C-0.3226	0	-0.32886600	-5.41128300	-2.34598000	L
O-OH--0.5579	0	0.91290000	-4.85814500	-2.32884300	L
H-HO-0.3992	0	0.91245400	-4.11687800	-1.71396800	L
N-N--0.4157	0	-6.27427000	-8.65888100	-4.18954000	L
H-H-0.2719	0	-5.45674500	-8.57025800	-4.76969700	L
C-CT--0.1490	-1	-7.12667200	-9.82503700	-4.36538500	L
H-H1-0.0976	0	-8.14141400	-9.50179300	-4.60775400	L
H-H1-0.0976	0	-6.74845900	-10.45582900	-5.17069600	L
H-H1-0.0976	0	-7.16032000	-10.40086400	-3.43821900	L
C-CT--0.3662	-1	-6.30294800	-3.60782600	-6.93007400	L
H-HC-0.1123	0	-5.81439500	-4.38052800	-7.51953400	L
H-HC-0.1123	0	-7.34193300	-3.88545300	-6.75753200	L
H-HC-0.1123	0	-5.79008200	-3.49140000	-5.98113200	L
C-C-0.5972	0	-6.23367900	-2.28736800	-7.68106900	L
O-O--0.5679	0	-5.34061900	-2.12567500	-8.50202200	L
N-N--0.415700	0	-7.14615600	-1.34812300	-7.39655400	L
H-H-0.271900	0	-7.85924900	-1.61033600	-6.73169700	L
C-CT--0.025200	-1	-7.25042400	-0.01326500	-8.03486200	L
H-H1-0.069800	0	-8.17414100	0.02889400	-8.61201200	L
H-H1-0.069800	0	-6.42385200	0.14954700	-8.72509400	L
C-C-0.597300	0	-7.27716100	1.13131800	-7.01531200	L
O-O--0.567900	0	-8.26964000	1.84370500	-6.92634500	L
N-N--0.415700	0	-6.19940000	1.29657200	-6.24303300	L
H-H-0.271900	0	-5.45915700	0.60865700	-6.33285600	L
C-CT--0.025200	-1	-5.80037600	2.55282500	-5.59492200	L
H-H1-0.069800	0	-5.61902800	2.39725000	-4.53745000	L
H-H1-0.069800	0	-6.57961200	3.30683000	-5.71670600	L
C-C-0.597300	0	-4.51787500	3.07617400	-6.25117700	L

O-O--0.567900	0	-4.46083200	3.21228700	-7.47076300	L
N-N--0.415700	0	-3.47213700	3.30304800	-5.45262000	L
H-H-0.271900	0	-3.59311600	3.14147000	-4.46332400	L
C-CT--0.051800	-1	-2.13132100	3.68331000	-5.91276400	L
H-H1-0.092200	0	-2.20875300	4.10856500	-6.91477300	L
C-C-0.597300	0	-1.21751500	2.44469100	-6.00104000	L
O-O--0.567900	0	-0.53584300	2.21365100	-6.99703100	L
C-CT--0.110200	0	-1.59195700	4.76601500	-4.95772400	L
H-HC-0.045700	0	-1.57072100	4.36377500	-3.94533200	L
H-HC-0.045700	0	-2.28698500	5.60709200	-4.96488200	L
C-CT-0.353100	0	-0.18581200	5.28924300	-5.29415000	L
H-HC--0.036100	0	0.52523600	4.46312400	-5.28039400	L
C-CT--0.412100	0	-0.12474800	5.97605700	-6.65848100	L
H-HC-0.100000	0	-0.87235200	6.76842400	-6.71155400	L
H-HC-0.100000	0	-0.31872400	5.24941900	-7.44741200	L
H-HC-0.100000	0	0.86611900	6.39941600	-6.81860900	L
C-CT--0.412100	0	0.23623900	6.30277700	-4.22823500	L
H-HC-0.100000	0	-0.46817400	7.13522600	-4.20866000	L
H-HC-0.100000	0	1.23318800	6.68142200	-4.45176600	L
H-HC-0.100000	0	0.25152600	5.81938300	-3.25250100	L
N-N--0.516300	0	-1.17656000	1.65496000	-4.92925300	L
H-H-0.293600	0	-1.72397100	1.96105600	-4.13077600	L
C-CT-0.038100	-1	-0.28083900	0.52012600	-4.71433600	L
H-H1-0.088000	0	-0.05285100	0.02578300	-5.65655800	L
C-C-0.536600	0	-0.91410900	-0.48983700	-3.72304400	L
O-O--0.581900	0	-0.65557300	-0.42000800	-2.52332700	L
C-CT--0.030300	0	1.03463000	1.12013900	-4.13583200	L
H-HC--0.012200	0	0.78008200	1.78029500	-3.30699700	L
H-HC--0.012200	0	1.50208200	1.73629200	-4.90582800	L
C-C-0.799400	-1	2.07891800	0.10426400	-3.64626100	L
O-O2--0.801400	0	2.04429800	-1.04983100	-4.13531100	L
O-O2--0.801400	0	3.01182600	0.46726400	-2.90101700	L
N-N--0.254800	0	-1.74692000	-1.43982300	-4.18140400	L
C-CT--0.026600	-1	-2.02441300	-1.76489000	-5.57630700	L
H-H1-0.064100	0	-1.09280100	-1.84820100	-6.13469900	L
C-C-0.589600	0	-2.93788000	-0.74158900	-6.27351300	L
O-O--0.574800	0	-3.82190100	-0.16382600	-5.64693200	L
C-CT--0.007000	0	-2.68955600	-3.14455900	-5.52252100	L
H-HC-0.025300	0	-1.91985100	-3.91782400	-5.49995800	L
H-HC-0.025300	0	-3.36650800	-3.31197900	-6.36074100	L
C-CT-0.018900	0	-3.42634700	-3.12965900	-4.18418700	L
H-HC-0.021300	0	-3.58765700	-4.13578300	-3.80179200	L
H-HC-0.021300	0	-4.37177200	-2.59468400	-4.28153600	L
C-CT-0.019200	0	-2.47888000	-2.33242000	-3.29414900	L
H-H1-0.039100	0	-3.04204700	-1.77239900	-2.54620800	L
H-H1-0.039100	0	-1.77411100	-3.00101300	-2.80482400	L
N-N--0.4157	0	-2.73071500	-0.52798000	-7.57574400	L
H-H-0.2719	0	-2.00642300	-1.05631900	-8.03046100	L
C-CT--0.1490	-1	-3.50256000	0.39235200	-8.41055200	L
H-H1-0.0976	0	-4.46300400	0.61182600	-7.95999300	L
H-H1-0.0976	0	-3.67909100	-0.05201700	-9.39102100	L
H-H1-0.0976	0	-2.95627300	1.33033300	-8.53291600	L
C-CT--0.3662	-1	8.77373400	1.81862400	-5.88105700	L
H-HC-0.1123	0	9.27600400	2.05930900	-6.81766800	L
H-HC-0.1123	0	9.51901600	1.60614300	-5.11740400	L

H-HC-0.1123	0	8.12401100	0.95850700	-6.02964200	L
C-C-0.5972	0	7.94799100	3.01101200	-5.45237300	L
O-O--0.5679	0	8.08872300	4.08391800	-6.02035800	L
N-N--0.415700	0	7.08759500	2.83187000	-4.44983200	L
H-H-0.271900	0	7.02302300	1.90756100	-4.04719200	L
C-CT-0.021300	-1	6.09686600	3.83399900	-4.03124900	L
H-H1-0.112400	0	6.28923900	4.77828700	-4.54500100	L
C-C-0.597300	0	6.13191300	4.09915400	-2.52420300	L
O-O--0.567900	0	6.39727400	3.19551500	-1.73129400	L
C-CT--0.123100	0	4.72049500	3.31349500	-4.47936100	L
H-H1-0.111200	0	4.49698100	2.37177200	-3.97109100	L
H-H1-0.111200	0	4.73925500	3.13394700	-5.55606000	L
S-SH--0.311900	0	3.41836700	4.52206000	-4.10885900	L
H-HS-0.193300	0	2.40643400	3.86190700	-4.69096400	L
N-N--0.415700	0	5.84316700	5.33539500	-2.12042200	L
H-H-0.271900	0	5.58883500	6.01670000	-2.82303100	L
C-CT--0.025200	-1	5.82422700	5.76053100	-0.72596200	L
H-H1-0.069800	0	6.78956800	6.20335400	-0.48137100	L
H-H1-0.069800	0	5.67982300	4.90576000	-0.06932200	L
C-C-0.597300	0	4.74894700	6.80691800	-0.45802100	L
O-O--0.567900	0	4.86100900	7.94696500	-0.89924700	L
N-N--0.415700	0	3.73168700	6.43886400	0.31497700	L
H-H-0.271900	0	3.77567500	5.50069100	0.70473300	L
C-CT--0.025200	-1	2.68532200	7.31299800	0.82947700	L
H-H1-0.069800	0	1.85344600	6.69762700	1.15958100	L
H-H1-0.069800	0	2.34548000	7.99777800	0.05190800	L
C-C-0.597300	0	3.17432700	8.11722400	2.03218600	L
O-O--0.567900	0	2.58718400	8.03120500	3.10848300	L
N-N--0.4157	0	4.26516200	8.86715300	1.84140100	L
H-H-0.2719	0	4.63063200	8.86512700	0.89410000	L
C-CT--0.1490	-1	5.00497100	9.56631200	2.88299200	L
H-H1-0.0976	0	5.32229700	8.85768900	3.64992700	L
H-H1-0.0976	0	5.88127600	10.05604800	2.45570300	L
H-H1-0.0976	0	4.36365300	10.31887000	3.34592500	L
C-CT--0.3662	-1	-1.53148300	9.49664100	5.17276600	L
H-HC-0.1123	0	-1.73882900	10.45541300	4.70031600	L
H-HC-0.1123	0	-0.59271400	9.09727900	4.79246200	L
H-HC-0.1123	0	-1.47140000	9.63151300	6.25123700	L
C-C-0.5972	0	-2.66857000	8.52407400	4.84721600	L
O-O--0.5679	0	-3.55625700	8.87321700	4.07925100	L
N-N--0.4157	0	-2.64074000	7.30371400	5.39839500	L
H-H-0.2719	0	-1.89079300	7.09679000	6.04372600	L
C-CT--0.0275	-1	-3.62776800	6.20591000	5.22669200	L
H-H1-0.1123	0	-4.62649500	6.63503100	5.14122300	L
C-C-0.5973	0	-3.60656100	5.25730900	6.45469200	L
O-O--0.5679	0	-2.70779100	5.34508500	7.28993100	L
C-CT--0.0050	0	-3.31227700	5.40345000	3.95486400	L
H-HC-0.0339	0	-3.51440700	6.03208300	3.09441400	L
H-HC-0.0339	0	-4.02463200	4.58183800	3.89645500	L
C-C*--0.1415	0	-1.92743300	4.82418800	3.82055200	L
C-CW--0.1638	0	-0.78804700	5.52109000	3.58869000	L
H-H4-0.2062	0	-0.72483600	6.59577800	3.48129200	L
C-CB-0.1243	0	-1.51816800	3.42276400	3.90378500	L
N-NA--0.3418	0	0.28822100	4.65699700	3.52095600	L
H-H-0.3412	0	1.24683600	4.95226400	3.38269200	L

C-CN-0.1380	0	-0.10458500	3.35113600	3.71071200	L
C-CA--0.238	0	-2.19640500	2.20429700	4.13166500	L
H-HA-0.1700	0	-3.26658400	2.21180200	4.25976500	L
C-CA--0.2601	0	0.60149200	2.14034100	3.74492300	L
H-HA-0.1572	0	1.66869300	2.12009900	3.57748500	L
C-CA--0.1972	0	-1.49700600	0.98299900	4.17277900	L
H-HA-0.1447	0	-2.02391300	0.04889200	4.32062800	L
C-CA--0.1134	0	-0.10593000	0.95002000	3.97878500	L
H-HA-0.1417	0	0.40781600	-0.00113300	3.97837000	L
N-N--0.4157	0	-4.58141500	4.33667000	6.56572800	L
H-H-0.2719	0	-5.28313400	4.34048800	5.84392600	L
C-CT--0.1490	-1	-4.68278600	3.32756100	7.63459400	L
H-H1-0.0976	0	-3.85131900	2.62551500	7.55400800	L
H-H1-0.0976	0	-4.63069100	3.81902500	8.60873200	L
H-H1-0.0976	0	-5.62568400	2.78439600	7.55683800	L
C-CT--0.3662	-1	-2.83465800	-1.39671200	7.30171100	L
H-HC-0.1123	0	-3.06645000	-2.44013700	7.08637600	L
H-HC-0.1123	0	-3.17668300	-1.14908300	8.30408200	L
H-HC-0.1123	0	-3.32349200	-0.76565300	6.56199100	L
C-C-0.5972	0	-1.34218600	-1.20423300	7.20793100	L
O-O--0.5679	0	-0.62544300	-2.15560100	6.95129400	L
N-N--0.415700	0	-0.84714400	0.00177600	7.48196000	L
H-H-0.271900	0	-1.50517100	0.75413700	7.60899400	L
C-CT--0.087500	-1	0.59584100	0.32899400	7.43756200	L
H-H1-0.096900	0	0.92807900	0.20935500	6.40644000	L
C-C-0.597300	0	1.44032900	-0.61980900	8.29934500	L
O-O--0.567900	0	2.44976800	-1.13874100	7.83678300	L
C-CT-0.298500	0	0.87602600	1.78872100	7.87016000	L
H-HC--0.029700	0	0.91727200	1.83435800	8.95909500	L
C-CT--0.319200	0	2.22977300	2.25254900	7.31820500	L
H-HC-0.079100	0	2.20713200	2.24997700	6.22803200	L
H-HC-0.079100	0	3.02340400	1.58923000	7.66259900	L
H-HC-0.079100	0	2.44528000	3.26231600	7.66818700	L
C-CT--0.319200	0	-0.19392600	2.79294700	7.42144400	L
H-HC-0.079100	0	-0.35732900	2.70800800	6.35132600	L
H-HC-0.079100	0	0.12546200	3.81066300	7.64825600	L
H-HC-0.079100	0	-1.13220600	2.61867600	7.94573800	L
N-N--0.415700	0	1.01268200	-0.87560100	9.54360600	L
H-H-0.271900	0	0.17664600	-0.40065200	9.84231400	L
C-CT--0.025200	-1	1.62255000	-1.86348100	10.44902500	L
H-H1-0.069800	0	1.08234000	-1.87823700	11.39494600	L
H-H1-0.069800	0	2.65566800	-1.57648800	10.64847100	L
C-C-0.597300	0	1.63299200	-3.30139800	9.87734300	L
O-O--0.567900	0	2.46765800	-4.09842800	10.28730800	L
N-N--0.415700	0	0.72085400	-3.63939500	8.95179900	L
H-H-0.271900	0	0.11314400	-2.91422100	8.59909800	L
C-CT-0.033700	-1	0.62682000	-4.93058800	8.25506100	L
H-H1-0.082300	0	1.03330900	-5.70085300	8.91334100	L
C-C-0.597300	0	1.46633500	-4.99206000	6.95443300	L
O-O--0.567900	0	2.11036100	-6.00130600	6.68048700	L
C-CT--0.182500	0	-0.84773100	-5.27644200	8.01120900	L
H-HC-0.060300	0	-1.38970500	-5.27874300	8.95705100	L
H-HC-0.060300	0	-0.91662800	-6.26809800	7.56238000	L
H-HC-0.060300	0	-1.30459500	-4.55752900	7.33436500	L
N-N--0.415700	0	1.49467700	-3.91098400	6.16820000	L

H-H-0.271900 0 0.85431300 -3.16359000 6.40988300 L
C-CT-0.033700 -1 2.43670200 -3.66484700 5.06360800 L
H-H1-0.082300 0 2.32820600 -4.44936100 4.31871700 L
C-C-0.597300 0 3.90976200 -3.64492900 5.53367300 L
O-O--0.567900 0 4.82566800 -3.84831000 4.73352500 L
C-CT--0.182500 0 2.06305600 -2.31145400 4.46726900 L
H-HC-0.060300 0 2.44658800 -1.50230600 5.08947000 L
H-HC-0.060300 0 0.97957400 -2.22425100 4.41526000 L
H-HC-0.060300 0 2.48482800 -2.23969600 3.46912200 L
N-N--0.4157 0 4.13746800 -3.33795500 6.81773700 L
H-H-0.2719 0 3.32388900 -3.05342900 7.34760100 L
C-CT--0.1490 -1 5.41252900 -3.40072000 7.51664800 L
H-H1-0.0976 0 5.90518900 -4.34979200 7.29657800 L
H-H1-0.0976 0 5.24653300 -3.32701700 8.59274900 L
H-H1-0.0976 0 6.05397500 -2.58143100 7.18899500 L
C-CT--0.3662 -1 14.09073600 0.91055000 -0.62209600 L
H-HC-0.1123 0 13.43413800 1.42526900 0.07648400 L
H-HC-0.1123 0 15.09525300 1.32644100 -0.55954100 L
H-HC-0.1123 0 13.71274300 1.01628500 -1.63679800 L
C-C-0.5972 0 14.14645100 -0.56574400 -0.26622600 L
O-O--0.5679 0 15.22752600 -1.12593600 -0.14778300 L
N-N--0.4157 0 12.98521600 -1.20146000 -0.11386000 L
H-H-0.2719 0 12.14269900 -0.65309700 -0.24120100 L
C-CT--0.0014 -1 12.80868700 -2.65201000 0.10744600 L
H-H1-0.0876 0 13.69159600 -3.17446700 -0.26351000 L
C-C-0.5973 0 11.58689800 -3.15672200 -0.71274800 L
O-O--0.5679 0 10.83023000 -2.36273000 -1.28072600 L
C-CT--0.0152 0 12.67255800 -2.94531600 1.61295600 L
H-HC-0.0295 0 13.61379500 -2.68245500 2.09827700 L
H-HC-0.0295 0 12.53295900 -4.01748700 1.75532500 L
C-CA--0.0011 -1 11.54401500 -2.20676400 2.31268900 L
C-CA--0.1906 0 11.78988300 -0.95921000 2.91688600 L
H-HA-0.1699 0 12.78876300 -0.54482800 2.90308000 L
C-CA--0.1906 0 10.24208200 -2.74607800 2.33087600 L
H-HA-0.1699 0 10.04322200 -3.70822000 1.87989200 L
C-CA--0.2341 0 10.73974000 -0.25309200 3.53335000 L
H-HA-0.1656 0 10.93227200 0.69281600 4.00607000 L
C-CA--0.2341 0 9.18761600 -2.03782100 2.93972500 L
H-HA-0.1656 0 8.19352400 -2.45891300 2.95023300 L
C-C-0.3226 0 9.43254900 -0.78595300 3.54497400 L
O-OH--0.557 0 8.42009900 -0.09227300 4.13403500 L
H-HO-0.3992 0 7.59527700 -0.62003900 4.11367200 L
N-N--0.415700 0 11.37756900 -4.47628200 -0.81498500 L
H-H-0.271900 0 11.99528500 -5.08990900 -0.30691200 L
C-CT--0.025200 -1 10.25845200 -5.08182500 -1.56653100 L
H-H1-0.069800 0 10.47430400 -6.13591000 -1.73984100 L
H-H1-0.069800 0 10.15731800 -4.59559200 -2.53601700 L
C-C-0.597300 0 8.93031300 -4.99703100 -0.80105000 L
O-O--0.567900 0 8.86023500 -5.38829800 0.36250300 L
N-N--0.415700 0 7.86369100 -4.51008600 -1.44123700 L
H-H-0.271900 0 7.94948200 -4.38771300 -2.44280800 L
C-CT-0.014300 -1 6.52178500 -4.48495000 -0.86150100 L
H-H1-0.104800 0 6.60891000 -4.37565900 0.22252200 L
C-C-0.597300 0 5.79151800 -5.80122300 -1.13228500 L
O-O--0.567900 0 5.08776800 -5.94710900 -2.13713400 L

C-CT--0.204100 0 5.70806700 -3.26676700 -1.36224700 L
H-HC-0.079700 0 5.57508500 -3.33618200 -2.44151800 L
H-HC-0.079700 0 6.28093200 -2.37658800 -1.14025100 L
C-C-0.713000 -1 4.32458000 -3.12262600 -0.70574500 L
O-O--0.593100 0 3.95238000 -3.84884200 0.20278200 L
N-N--0.919100 0 3.48924600 -2.22655800 -1.18963500 L
H-H-0.419600 0 2.60949800 -2.15258700 -0.70854100 L
H-H-0.419600 0 3.68882800 -1.63405000 -1.98703700 L
N-N--0.347900 0 5.93399800 -6.73340200 -0.18737900 L
H-H-0.274700 0 6.59391100 -6.49753200 0.54391600 L
C-CT--0.263700 -1 5.37507100 -8.09062400 -0.23134300 L
H-H1-0.156000 0 5.74724800 -8.62066300 0.64562300 L
C-C-0.734100 0 5.88492700 -8.90351100 -1.43198800 L
O-O--0.589400 0 6.89863400 -8.57462800 -2.04139600 L
C-CT--0.000700 0 3.83545800 -8.04577000 -0.13170700 L
H-HC-0.032700 0 3.44529700 -9.06340200 -0.09289200 L
H-HC-0.032700 0 3.43509500 -7.57141800 -1.02913600 L
C-CT-0.039000 0 3.30082900 -7.31629400 1.10630900 L
H-HC-0.028500 0 3.70488700 -6.30716200 1.16564900 L
H-HC-0.028500 0 3.56780600 -7.86786500 2.00884500 L
C-CT-0.048600 0 1.78073900 -7.25109700 0.95956700 L
H-H1-0.068700 0 1.38836800 -8.26494100 0.86194200 L
H-H1-0.068700 0 1.52930000 -6.70465900 0.04735200 L
N-N2--0.529500 -1 1.12197000 -6.60982200 2.09983900 L
H-H-0.345600 0 0.41532900 -7.14665300 2.58483200 L
C-CA-0.807600 0 0.90955000 -5.32545400 2.24396900 L
N-N2--0.862700 0 -0.08417800 -4.93315500 2.97394200 L
H-H-0.447800 0 -0.76446700 -5.59822700 3.31144300 L
H-H-0.447800 0 -0.30429500 -3.93509200 3.03209000 L
N-N2--0.862700 0 1.63073000 -4.41980500 1.66135300 L
H-H-0.447800 0 2.49986700 -4.58987600 1.17415000 L
H-H-0.447800 0 1.29069500 -3.46917000 1.80037900 L
N-N--0.4157 0 5.20006700 -10.01116800 -1.72332300 L
H-H-0.2719 0 4.40091000 -10.20806100 -1.14560900 L
C-CT--0.1490 -1 5.51276600 -10.91988900 -2.81133800 L
H-H1-0.0976 0 6.52310100 -11.31468000 -2.68324600 L
H-H1-0.0976 0 4.80373800 -11.74893900 -2.82972300 L
H-H1-0.0976 0 5.46757400 -10.38554000 -3.76248500 L
C-CT--0.3662 -1 10.18563600 -6.04294500 -7.62886100 L
H-HC-0.1123 0 9.89827800 -6.74109300 -8.41259600 L
H-HC-0.1123 0 11.09144000 -5.51737600 -7.92977300 L
H-HC-0.1123 0 10.37128900 -6.57350100 -6.69650500 L
C-C-0.5972 0 9.07928200 -5.03124100 -7.42017400 L
O-O--0.5679 0 9.31192100 -3.83614400 -7.56599900 L
N-N--0.347900 0 7.87983400 -5.48014600 -7.02151600 L
H-H-0.274700 0 7.79296400 -6.47493200 -6.87750200 L
C-CT--0.263700 -1 6.89635900 -4.57381900 -6.39522800 L
H-H1-0.156000 0 6.73881200 -3.71697800 -7.05124800 L
C-C-0.734100 0 7.52124400 -4.05864700 -5.09600100 L
O-O--0.589400 0 7.86954000 -4.85127300 -4.22699500 L
C-CT--0.000700 0 5.54618600 -5.25608900 -6.12710600 L
H-HC-0.032700 0 4.94645900 -4.55865000 -5.54518100 L
H-HC-0.032700 0 5.68879400 -6.15233900 -5.51966900 L
C-CT-0.039000 0 4.79603700 -5.62333800 -7.42080200 L
H-HC-0.028500 0 5.02903700 -6.65939300 -7.66977600 L

H-HC-0.028500	0	5.14017900	-4.99710300	-8.24553900	L
C-CT-0.048600	0	3.27005500	-5.46911300	-7.29731300	L
H-H1-0.068700	0	2.92771200	-5.91338200	-6.36205100	L
H-H1-0.068700	0	2.80673700	-6.02961300	-8.11138900	L
N-N2--0.529500	0	2.84594000	-4.05557000	-7.41149800	L
H-H-0.345600	0	2.80027200	-3.67158300	-8.33863300	L
C-CA-0.807600	0	2.53408000	-3.21122500	-6.44170100	L
N-N2--0.862700	0	2.50048800	-3.52773500	-5.18597700	L
H-H-0.447800	0	2.70301600	-4.46630100	-4.85675300	L
H-H-0.447800	0	2.28661700	-2.77556200	-4.52651000	L
N-N2--0.862700	0	2.25122800	-1.97121100	-6.70133000	L
H-H-0.447800	0	2.25167300	-1.56247000	-7.61438100	L
H-H-0.447800	0	2.10864500	-1.40538300	-5.86093600	L
N-N--0.415700	0	7.73303700	-2.75019600	-4.98560000	L
H-H-0.271900	0	7.43368400	-2.16713200	-5.74970200	L
C-CT--0.025200	-1	8.63373000	-2.16031700	-3.99069000	L
H-H1-0.069800	0	9.51093500	-1.77466600	-4.51091900	L
H-H1-0.069800	0	8.98419800	-2.91324100	-3.28944400	L
C-C-0.597300	0	8.03271000	-1.01787400	-3.19175600	L
O-O--0.567900	0	6.97539000	-0.48270400	-3.52915900	L
N-N--0.415700	0	8.71901000	-0.61809200	-2.12440900	L
H-H-0.271900	0	9.57341400	-1.10847000	-1.86277000	L
C-CT--0.038900	-1	8.31193000	0.52980600	-1.31963000	L
H-H1-0.100700	0	7.68888600	1.15179200	-1.95873000	L
C-C-0.597300	0	9.44899000	1.46029600	-0.92270200	L
O-O--0.567900	0	10.55421100	1.04719100	-0.57622200	L
C-CT-0.365400	0	7.40746400	0.11168400	-0.14325600	L
H-H1-0.004300	0	7.71682300	-0.86770600	0.22462600	L
O-OH--0.676100	0	6.09194300	0.04209900	-0.65832900	L
H-HO-0.410200	0	6.16846200	-0.19014500	-1.59602100	L
C-CT--0.243800	0	7.32300700	1.07690800	1.03948100	L
H-HC-0.064200	0	6.97378300	2.05467600	0.70965900	L
H-HC-0.064200	0	8.29874400	1.17130300	1.51478000	L
H-HC-0.064200	0	6.62306600	0.68127600	1.77566900	L
N-N--0.415700	0	9.12837300	2.75215600	-1.00691200	L
H-H-0.271900	0	8.16324000	2.95561600	-1.24611300	L
C-CT--0.025200	-1	9.96182800	3.88577200	-0.65613500	L
H-H1-0.069800	0	9.76749200	4.70129800	-1.35260500	L
H-H1-0.069800	0	11.01378300	3.60616500	-0.73541400	L
C-C-0.597300	0	9.65670100	4.37460500	0.74735600	L
O-O--0.567900	0	8.50540800	4.57304500	1.14909300	L
N-N--0.347900	0	10.72838700	4.60255700	1.49092100	L
H-H-0.274700	0	11.63632100	4.45383200	1.06517200	L
C-CT--0.263700	-1	10.73260200	5.20957300	2.81262500	L
H-H1-0.156000	0	9.82635300	5.79507300	2.95555900	L
C-C-0.734100	0	11.90179800	6.16711400	2.90767400	L
O-O--0.589400	0	12.84990500	6.08121400	2.13259800	L
C-CT--0.000700	0	10.88080000	4.11577300	3.89243200	L
H-HC-0.032700	0	11.15687400	4.57202800	4.84472400	L
H-HC-0.032700	0	11.69286800	3.44335500	3.60763600	L
C-CT-0.039000	0	9.61465200	3.28684900	4.11829700	L
H-HC-0.028500	0	9.84712900	2.47305200	4.80459100	L
H-HC-0.028500	0	9.27443900	2.85726200	3.17577300	L
C-CT-0.048600	0	8.50797300	4.13243700	4.74971800	L
H-H1-0.068700	0	8.31885600	5.02405400	4.15295300	L

H-H1-0.068700	0	8.82610500	4.45019200	5.74439100	L
N-N2--0.529500	-1	7.27517300	3.35274500	4.86497200	L
H-H-0.345600	0	7.18377400	2.73713500	5.65447400	L
C-CA-0.807600	0	6.28043300	3.31316400	4.01060600	L
N-N2--0.862700	0	5.23664700	2.59208800	4.27430500	L
H-H-0.447800	0	5.20593400	1.95400000	5.04355200	L
H-H-0.447800	0	4.59863100	2.45247600	3.48545500	L
N-N2--0.862700	0	6.30337700	3.97254400	2.89467500	L
H-H-0.447800	0	7.15722400	4.33836900	2.49805600	L
H-H-0.447800	0	5.44501200	3.97183800	2.34455600	L
N-N--0.4157	0	11.81779400	7.05677100	3.88746700	L
H-H-0.2719	0	11.00156700	7.04557500	4.47512200	L
C-CT--0.1490	-1	12.88025900	7.98674100	4.17299300	L
H-H1-0.0976	0	12.61383700	8.62146600	5.01969700	L
H-H1-0.0976	0	13.79838300	7.44366300	4.40934100	L
H-H1-0.0976	0	13.06827400	8.61707100	3.30059000	L
C-CT--0.3662	-1	-2.16116300	13.22040400	-1.69717600	L
H-HC-0.1123	0	-2.59149900	13.73895900	-0.84295000	L
H-HC-0.1123	0	-1.07483500	13.21141600	-1.63406100	L
H-HC-0.1123	0	-2.46132300	13.72837900	-2.61342900	L
C-C-0.5972	0	-2.67897600	11.79454800	-1.74279600	L
O-O--0.5679	0	-3.32433800	11.40932400	-2.70504100	L
N-N--0.415700	0	-2.40534300	11.02144100	-0.68832200	L
H-H-0.271900	0	-1.84910400	11.44953700	0.03256200	L
C-CT--0.023700	-1	-2.83288400	9.61858200	-0.49962300	L
H-H1-0.088000	0	-2.35661200	9.01057900	-1.27036900	L
C-C-0.597300	0	-4.35164300	9.44015900	-0.67613900	L
O-O--0.567900	0	-4.80996100	8.66516100	-1.51621700	L
C-CT-0.034200	0	-2.31613600	9.16283800	0.87100500	L
H-HC-0.024100	0	-2.69573800	9.83305500	1.64344000	L
H-HC-0.024100	0	-1.22780800	9.23041500	0.87695100	L
C-CT-0.001800	0	-2.71785000	7.73735500	1.23586900	L
H-H1-0.044000	0	-3.78951800	7.72536300	1.43778700	L
H-H1-0.044000	0	-2.20466400	7.48560500	2.15941200	L
S-S--0.273700	0	-2.36897600	6.44335300	0.02056500	L
C-CT--0.053600	0	-0.56923800	6.48942700	-0.08050900	L
H-H1-0.068400	0	-0.23747800	5.79508100	-0.85188400	L
H-H1-0.068400	0	-0.15831900	6.18761100	0.88078900	L
H-H1-0.068400	0	-0.23345300	7.49275900	-0.33962400	L
N-N--0.4157	0	-5.13923200	10.17228700	0.11647900	L
H-H-0.2719	0	-4.68500900	10.77863100	0.78030800	L
C-CT--0.1490	-1	-6.58923900	10.19549600	0.00966800	L
H-H1-0.0976	0	-7.01236900	10.84700000	0.77558200	L
H-H1-0.0976	0	-6.87754600	10.56625900	-0.97626800	L
H-H1-0.0976	0	-6.98463300	9.18619700	0.13742400	L
C-CT--0.3662	-1	-4.42406500	8.91049600	-5.24895500	L
H-HC-0.1123	0	-3.98593800	9.25652400	-4.31349700	L
H-HC-0.1123	0	-3.66131600	8.90021600	-6.02555600	L
H-HC-0.1123	0	-5.23690000	9.56877600	-5.54686800	L
C-C-0.5972	0	-4.96272800	7.49862100	-5.07071000	L
O-O--0.5679	0	-4.68682300	6.63323800	-5.88897400	L
N-N--0.415700	0	-5.73961600	7.26839200	-4.00859700	L
H-H-0.271900	0	-5.74300200	8.01721300	-3.32716600	L
C-CT--0.087500	-1	-6.38036800	5.97313600	-3.65352900	L
H-H1-0.096900	0	-6.10520800	5.22479200	-4.39633200	L

C-C-0.597300	0	-7.89933000	6.09454500	-3.70296600	L
O-O--0.567900	0	-8.55614800	5.28924500	-4.35078500	L
C-CT-0.298500	0	-5.86564800	5.50230500	-2.28181300	L
H-HC--0.029700	0	-6.01919100	6.28993600	-1.54417900	L
C-CT--0.319200	0	-6.55054300	4.23724900	-1.76219900	L
H-HC-0.079100	0	-6.42187900	3.43785400	-2.48782900	L
H-HC-0.079100	0	-7.60636000	4.43443800	-1.58617400	L
H-HC-0.079100	0	-6.09944500	3.93768900	-0.81716300	L
C-CT--0.319200	0	-4.37329300	5.19740800	-2.38818900	L
H-HC-0.079100	0	-4.19626100	4.46571500	-3.17007000	L
H-HC-0.079100	0	-4.02123000	4.79932400	-1.44495100	L
H-HC-0.079100	0	-3.81311400	6.10483100	-2.61275000	L
N-N--0.4157	0	-8.47178800	7.09092600	-3.01962600	L
H-H-0.2719	0	-7.85249700	7.72263300	-2.53658800	L
C-CT--0.1490	-1	-9.90972200	7.34056900	-3.02941700	L
H-H1-0.0976	0	-10.14891400	8.20585800	-2.41034900	L
H-H1-0.0976	0	-10.24024800	7.52863000	-4.05346200	L
H-H1-0.0976	0	-10.43958900	6.46429000	-2.65153100	L
C-CT-0.1200	-1	-8.88178400	1.99185100	3.19735400	L
H-HC-0.0800	0	-8.09311700	2.73997000	3.24558000	L
H-HC-0.0800	0	-9.71740900	2.40014600	2.62928300	L
H-HC-0.0800	0	-9.22494000	1.76012200	4.20656000	L
C-CT-0.2000	0	-8.40395500	0.75152800	2.53480700	L
H-H1-0.0800	0	-9.21195100	0.01661500	2.47753800	L
O-OS--0.5600	0	-7.94739500	1.10081400	1.24864700	L
C-CT-0.2000	0	-7.22287700	0.19734400	3.31732400	L
H-H1-0.0800	0	-6.51691500	0.98265500	3.58859400	L
O-OH--0.6800	0	-7.61925200	-0.53647200	4.46920400	L
H-HO-0.4000	0	-8.25544500	-1.20530900	4.17228200	L
C-CT-0.2000	0	-6.65231300	-0.62993200	2.18596100	L
H-H1-0.0800	0	-5.60459400	-0.86563000	2.37128800	L
O-OH--0.6800	0	-7.44973100	-1.80741500	2.07939700	L
H-HO-0.4000	0	-7.02377000	-2.40438900	1.43271900	L
C-CT-0.5691	0	-6.81736600	0.27464000	0.95061900	H
H-H2-0.8000	0	-6.97587000	-0.37712200	0.06518700	H
N-N*--0.5691	0	-5.68567700	1.16305000	0.72483700	H
C-CM--0.0500	0	-5.00022700	1.00336900	-0.43935600	H
H-H4-0.1500	0	-5.44085300	0.26318500	-1.07092800	H
C-CM--0.1238	0	-3.92969000	1.70974200	-0.80184200	H
C-C-0.6156	0	-3.43862400	1.68704600	-2.19023800	H
O-O--0.5700	0	-2.54189700	2.40467200	-2.56373200	H
N-N--0.8000	0	-4.11481800	0.92427500	-3.09907300	H
H-H-0.3700	0	-3.66709400	0.70276600	-3.96545700	H
H-H-0.3700	0	-4.72297000	0.18301000	-2.80280100	H
C-CT-0.1164	0	-3.25916900	2.59358000	0.22296000	H
H-HC-0.0800	0	-2.79288900	3.44557700	-0.25241100	H
H-HC-0.0800	0	-2.46148900	2.04674100	0.71751800	H
C-CM--0.2882	0	-4.29907200	3.02684100	1.22852200	H
H-HA-0.1500	0	-4.14366800	3.94853400	1.75200700	H
C-CM--0.0500	0	-5.38332500	2.31193900	1.46735200	H
H-H4-0.1500	0	-6.10833700	2.61738700	2.18939000	H
N-N3--0.8530	0	3.35482600	0.97881900	-0.30964600	H
H-H-0.4500	0	3.25257700	0.64187000	-1.25344400	H
H-H-0.4500	0	2.61200300	0.66647900	0.32404700	H
H-H-0.4500	0	4.23912400	0.68383200	0.07590400	H

C-CT-0.3170	0	3.46839000	2.44796800	-0.30285200	H
H-HP-0.0800	0	4.31352300	2.69500400	-0.92711000	H
C-CT--0.1600	0	2.21418100	3.17555700	-0.77825200	H
H-HC-0.0800	0	2.05792200	2.94971000	-1.82601100	H
H-HC-0.0800	0	2.46276300	4.22746800	-0.72570400	H
C-CT--0.1600	0	0.95306100	2.87857200	0.04362800	H
H-HC-0.0800	0	0.26732200	3.70207400	-0.08235700	H
H-HC-0.0800	0	1.22820800	2.87512400	1.09417300	H
C-CT--0.0990	0	0.24377100	1.56523000	-0.33605000	H
H-HC-0.0800	0	0.83692800	1.02499600	-1.07124000	H
H-HC-0.0800	0	-0.68881900	1.79229100	-0.82371600	H
C-C-0.4490	0	0.00235500	0.60158200	0.82466700	H
H-H-0.0600	0	-0.31198000	1.15704700	1.70488100	H
O-O--0.5700	0	1.24351400	-0.02579500	1.12133200	H
N-N3--0.9900	0	-1.02678500	-0.31339900	0.48478300	H
H-H-0.3600	0	1.00288700	-0.89790400	1.50465100	H
H-H-0.3600	0	-0.87940400	-0.69359500	-0.43347100	H
C-C-0.9060	0	3.85032200	2.79247100	1.13084600	L
O-O2--0.9000	0	3.97502600	1.90315100	2.00562900	L
O-O2--0.9000	0	4.03841000	3.98322800	1.44350300	L
C-CT--0.205900	0	-1.77754800	-1.13949300	1.41761200	H
H-H1-0.139900	0	-2.39826400	-0.47419600	2.01028000	H
C-CT-0.007100	0	-2.69889600	-1.98243600	0.53166400	H
H-HC--0.007800	0	-2.10622200	-2.63498400	-0.10032100	H
H-HC--0.007800	0	-3.23579300	-1.30077900	-0.11927100	H
C-CT-0.067500	0	-3.69482300	-2.84165500	1.29135200	L
H-HC--0.054800	0	-3.99194100	-2.31416600	2.19785600	L
H-HC--0.054800	0	-3.22045200	-3.78249400	1.57756200	L
C-C-0.818300	0	-4.95304800	-3.10165500	0.46872300	L
O-O2--0.822000	0	-5.65804600	-2.11650000	0.13865700	L
O-O2--0.822000	0	-5.34891000	-4.27416100	0.25197900	L
C-C-0.742000	0	-0.94852100	-2.01344200	2.37424400	H
O-O2--0.793000	0	-1.51150800	-2.52974700	3.33000700	H
O-O2--0.793000	0	0.24976700	-2.19084300	2.05870600	H
O-OW--0.834000	0	3.02598300	-5.94735500	-3.82375600	L
H-HW-0.417000	0	3.82202200	-6.04407000	-3.26154800	L
H-HW-0.417000	0	2.32583700	-5.76792000	-3.17632700	L
O-OW--0.834000	0	-1.76149900	-7.32818800	2.48454500	L
H-HW-0.417000	0	-2.48730100	-6.86271400	2.02069700	L
H-HW-0.417000	0	-2.12716400	-8.20776900	2.60003900	L
O-OW--0.834000	0	4.10956800	-0.60123700	2.38271400	L
H-HW-0.417000	0	3.17563700	-0.83143100	2.38037700	L
H-HW-0.417000	0	4.09111000	0.38477800	2.30483100	L
O-OW--0.834000	0	6.06272500	-1.44346100	4.05082900	L
H-HW-0.417000	0	5.75554800	-2.32772800	4.32071200	L
H-HW-0.417000	0	5.37839800	-1.17542300	3.40323300	L
O-OW--0.834000	0	-4.14933600	-6.34831100	1.48216000	L
H-HW-0.417000	0	-4.59628300	-5.67412200	0.92938100	L
H-HW-0.417000	0	-4.57972200	-6.21838000	2.33657200	L
O-OW--0.834000	0	-9.07076300	-1.32904800	-0.21645800	L
H-HW-0.417000	0	-9.91187400	-1.40202100	0.23911900	L
H-HW-0.417000	0	-8.72845300	-2.24700600	-0.18021600	L

TS5

C-CT--0.3662	-1	-12.877986	1.043612	1.886715	L

H-HC-0.1123 0 -12.378552 1.971873 1.613465 L
H-HC-0.1123 0 -13.950156 1.157456 1.735685 L
H-HC-0.1123 0 -12.494824 0.230064 1.274267 L
C-C-0.5972 0 -12.603195 0.744316 3.348048 L
O-O--0.5679 0 -11.829544 1.460448 3.973487 L
N-N--0.415700 0 -13.211563 -0.321750 3.877809 L
H-H-0.271900 0 -13.819883 -0.845838 3.270201 L
C-CT--0.025200 -1 -12.919551 -0.871769 5.214827 L
H-H1-0.069800 0 -13.669520 -1.621158 5.467280 L
H-H1-0.069800 0 -12.961009 -0.068409 5.951033 L
C-C-0.597300 0 -11.510231 -1.532054 5.284383 L
O-O--0.567900 0 -10.701956 -1.426188 4.356540 L
N-N--0.415700 0 -11.199594 -2.246960 6.377112 L
H-H-0.271900 0 -11.900208 -2.329223 7.096706 L
C-CT--0.025200 -1 -10.011164 -3.124789 6.470297 L
H-H1-0.069800 0 -9.926728 -3.521656 7.481630 L
H-H1-0.069800 0 -9.113038 -2.551288 6.236778 L
C-C-0.597300 0 -10.150150 -4.291869 5.502776 L
O-O--0.567900 0 -11.190235 -4.942527 5.449259 L
N-N--0.4157 0 -9.092201 -4.551673 4.746681 L
H-H-0.2719 0 -8.268114 -3.989361 4.889032 L
C-CT--0.0014 -1 -9.015691 -5.508750 3.642893 L
H-H1-0.0876 0 -9.858130 -5.366415 2.965145 L
C-C-0.5973 0 -8.993933 -6.993955 4.064145 L
O-O--0.5679 0 -8.363567 -7.827584 3.415938 L
C-CT--0.0152 0 -7.713588 -5.159949 2.903586 L
H-HC-0.0295 0 -7.889546 -4.197470 2.444089 L
H-HC-0.0295 0 -7.558465 -5.857530 2.078956 L
C-CA--0.0011 0 -6.413641 -5.047149 3.709695 L
C-CA--0.1906 0 -5.610720 -6.177048 3.970198 L
H-HA-0.1699 0 -5.978530 -7.168475 3.733054 L
C-CA--0.1906 0 -5.925300 -3.774520 4.069092 L
H-HA-0.1699 0 -6.537535 -2.902021 3.955256 L
C-CA--0.2341 0 -4.288630 -6.012755 4.443935 L
H-HA-0.1656 0 -3.640303 -6.863886 4.589791 L
C-CA--0.2341 0 -4.598223 -3.600947 4.497103 L
H-HA-0.1656 0 -4.193333 -2.614203 4.664893 L
C-C-0.3326 0 -3.756082 -4.717670 4.632248 L
O-OH--0.5579 0 -2.434230 -4.533631 4.881146 L
H-HO-0.3992 0 -2.112611 -3.721965 4.434956 L
N-N--0.4157 0 -9.663992 -7.342885 5.161313 L
H-H-0.2719 0 -10.258133 -6.633065 5.572388 L
C-CT--0.1490 -1 -9.595489 -8.668537 5.747028 L
H-H1-0.0976 0 -8.564305 -8.900122 6.020121 L
H-H1-0.0976 0 -10.225743 -8.719939 6.635694 L
H-H1-0.0976 0 -9.938478 -9.408887 5.021171 L
C-CT--0.3662 -1 -11.991176 -1.524109 -3.485801 L
H-HC-0.1123 0 -12.496495 -0.567282 -3.604571 L
H-HC-0.1123 0 -11.991215 -1.831714 -2.442528 L
H-HC-0.1123 0 -12.502946 -2.276206 -4.085603 L
C-C-0.5972 0 -10.564345 -1.401781 -3.978500 L
O-O--0.5679 0 -10.142507 -2.116452 -4.877303 L
N-N--0.415700 0 -9.792490 -0.517048 -3.357331 L
H-H-0.271900 0 -10.187644 -0.010827 -2.580475 L
C-CT--0.038900 -1 -8.392973 -0.176761 -3.700579 L

H-H1-0.100700	0	-8.290564	-0.116453	-4.782128	L
C-C-0.597300	0	-7.395510	-1.218138	-3.179103	L
O-O--0.567900	0	-6.255569	-0.901779	-2.849400	L
C-CT-0.365400	0	-8.074623	1.201932	-3.102000	L
H-H1-0.004300	0	-7.092498	1.510969	-3.440970	L
O-OH--0.676100	0	-8.067738	1.169156	-1.690766	L
H-HO-0.410200	0	-7.944669	2.080981	-1.403399	L
C-CT--0.243800	0	-9.101812	2.266275	-3.490513	L
H-HC-0.064200	0	-10.099488	2.012536	-3.136553	L
H-HC-0.064200	0	-9.124979	2.383750	-4.573036	L
H-HC-0.064200	0	-8.830980	3.216825	-3.048385	L
N-N--0.415700	0	-7.831053	-2.467166	-3.051692	L
H-H-0.271900	0	-8.707316	-2.685440	-3.510080	L
C-CT--0.024900	-1	-7.189394	-3.504924	-2.247122	L
H-H1-0.084300	0	-6.347030	-3.095081	-1.721663	L
C-C-0.597300	0	-6.637984	-4.637623	-3.105967	L
O-O--0.567900	0	-7.045532	-4.834941	-4.247373	L
C-CT-0.211700	0	-8.217240	-4.157133	-1.311523	L
H-H1-0.035200	0	-8.157229	-5.246573	-1.358317	L
H-H1-0.035200	0	-9.230810	-3.861657	-1.587161	L
O-OH--0.654600	0	-7.939309	-3.759847	0.001107	L
H-HO-0.427500	0	-6.997945	-4.020607	0.154603	L
N-N--0.4157	0	-5.750459	-5.437430	-2.520469	L
H-H-0.2719	0	-5.488324	-5.221158	-1.563213	L
C-CT--0.0014	0	-5.325234	-6.706686	-3.107558	L
H-H1-0.0876	0	-4.926637	-6.511204	-4.103456	L
C-C-0.5973	0	-6.462873	-7.728122	-3.246549	L
O-O--0.5679	0	-7.434022	-7.716808	-2.492188	L
C-CT--0.0152	0	-4.224647	-7.312236	-2.243605	L
H-HC-0.0295	0	-4.049812	-8.343488	-2.553337	L
H-HC-0.0295	0	-4.555480	-7.343074	-1.203791	L
C-CA--0.0011	-1	-2.913458	-6.578331	-2.341022	L
C-CA--0.1906	0	-2.004361	-6.882698	-3.377104	L
H-HA-0.1699	0	-2.281791	-7.590217	-4.145414	L
C-CA--0.1906	0	-2.524441	-5.668721	-1.339106	L
H-HA-0.1699	0	-3.205511	-5.417008	-0.546478	L
C-CA--0.2341	0	-0.721714	-6.293459	-3.398404	L
H-HA-0.1656	0	-0.020240	-6.526645	-4.184766	L
C-CA--0.2341	0	-1.237652	-5.100725	-1.335721	L
H-HA-0.1656	0	-0.943912	-4.436202	-0.536287	L
C-C-0.3226	0	-0.334818	-5.406458	-2.370872	L
O-OH--0.5579	0	0.903386	-4.845298	-2.367460	L
H-HO-0.3992	0	0.901092	-4.093790	-1.765010	L
N-N--0.4157	0	-6.277218	-8.667800	-4.180819	L
H-H-0.2719	0	-5.461613	-8.577140	-4.763379	L
C-CT--0.1490	-1	-7.129972	-9.833270	-4.358050	L
H-H1-0.0976	0	-8.144122	-9.509417	-4.602039	L
H-H1-0.0976	0	-6.750844	-10.464293	-5.162760	L
H-H1-0.0976	0	-7.165447	-10.409177	-3.430992	L
C-CT--0.3662	-1	-6.309188	-3.618040	-6.928473	L
H-HC-0.1123	0	-5.819904	-4.390488	-7.517710	L
H-HC-0.1123	0	-7.348045	-3.896479	-6.756438	L
H-HC-0.1123	0	-5.797017	-3.501706	-5.979189	L
C-C-0.5972	0	-6.240371	-2.297754	-7.679682	L
O-O--0.5679	0	-5.346368	-2.135395	-8.499310	L

N-N--0.415700 0 -7.153998 -1.359214 -7.396575 L
H-H-0.271900 0 -7.868407 -1.622170 -6.733461 L
C-CT--0.025200 -1 -7.257886 -0.024268 -8.034780 L
H-H1-0.069800 0 -8.182013 0.018558 -8.611240 L
H-H1-0.069800 0 -6.431772 0.138155 -8.725660 L
C-C-0.597300 0 -7.283249 1.119885 -7.014819 L
O-O--0.567900 0 -8.276836 1.830113 -6.921963 L
N-N--0.415700 0 -6.203444 1.286708 -6.245712 L
H-H-0.271900 0 -5.461705 0.600958 -6.339595 L
C-CT--0.025200 -1 -5.804642 2.543595 -5.598612 L
H-H1-0.069800 0 -5.622067 2.388963 -4.541316 L
H-H1-0.069800 0 -6.584252 3.297234 -5.720437 L
C-C-0.597300 0 -4.522869 3.066745 -6.256531 L
O-O--0.567900 0 -4.467305 3.201562 -7.476247 L
N-N--0.415700 0 -3.476321 3.294549 -5.459188 L
H-H-0.271900 0 -3.596749 3.135065 -4.469787 L
C-CT--0.051800 -1 -2.135936 3.673663 -5.921935 L
H-H1-0.092200 0 -2.215273 4.100708 -6.923066 L
C-C-0.597300 0 -1.222989 2.434587 -6.014989 L
O-O--0.567900 0 -0.548970 2.199607 -7.015080 L
C-CT--0.110200 0 -1.593288 4.754602 -4.966710 L
H-HC-0.045700 0 -1.572105 4.351914 -3.954527 L
H-HC-0.045700 0 -2.286439 5.597208 -4.972922 L
C-CT-0.353100 0 -0.186239 5.274999 -5.303970 L
H-HC--0.036100 0 0.523247 4.447595 -5.289308 L
C-CT--0.412100 0 -0.124093 5.960063 -6.669112 L
H-HC-0.100000 0 -0.870114 6.753814 -6.723194 L
H-HC-0.100000 0 -0.319185 5.232784 -7.457183 L
H-HC-0.100000 0 0.867668 6.381225 -6.829677 L
C-CT--0.412100 0 0.237287 6.289073 -4.239150 L
H-HC-0.100000 0 -0.465678 7.122719 -4.220386 L
H-HC-0.100000 0 1.234866 6.665798 -4.463299 L
H-HC-0.100000 0 0.252485 5.806565 -3.262996 L
N-N--0.516300 0 -1.176108 1.648398 -4.941540 L
H-H-0.293600 0 -1.717591 1.958654 -4.140673 L
C-CT-0.038100 -1 -0.284094 0.511295 -4.723452 L
H-H1-0.088000 0 -0.054142 0.011403 -5.662185 L
C-C-0.536600 0 -0.934107 -0.481441 -3.725606 L
O-O--0.581900 0 -0.713260 -0.374250 -2.519291 L
C-CT--0.030300 0 1.027167 1.110723 -4.134951 L
H-HC--0.012200 0 0.769601 1.756489 -3.295533 L
H-HC--0.012200 0 1.491474 1.740221 -4.896093 L
C-C-0.799400 -1 2.076989 0.096127 -3.658039 L
O-O2--0.801400 0 1.982850 -1.084875 -4.070648 L
O-O2--0.801400 0 3.084677 0.480676 -3.027273 L
N-N--0.254800 0 -1.751350 -1.445518 -4.185567 L
C-CT--0.026600 -1 -2.028862 -1.774288 -5.581496 L
H-H1-0.064100 0 -1.097533 -1.857981 -6.140285 L
C-C-0.589600 0 -2.942881 -0.750307 -6.277238 L
O-O--0.574800 0 -3.821728 -0.167266 -5.647389 L
C-CT--0.007000 0 -2.693894 -3.153718 -5.524411 L
H-HC-0.025300 0 -1.924111 -3.926766 -5.497875 L
H-HC-0.025300 0 -3.369364 -3.323832 -6.363300 L
C-CT-0.018900 0 -3.433113 -3.134320 -4.187415 L
H-HC-0.021300 0 -3.593771 -4.139136 -3.801191 L

H-HC-0.021300	0	-4.379369	-2.601561	-4.288976	L
C-CT-0.019200	0	-2.490259	-2.331374	-3.297398	L
H-H1-0.039100	0	-3.058370	-1.765780	-2.557539	L
H-H1-0.039100	0	-1.789340	-2.996677	-2.798357	L
N-N--0.4157	0	-2.740555	-0.541171	-7.580944	L
H-H-0.2719	0	-2.020375	-1.073969	-8.037125	L
C-CT--0.1490	-1	-3.510479	0.380883	-8.415504	L
H-H1-0.0976	0	-4.470428	0.602091	-7.964902	L
H-H1-0.0976	0	-3.687686	-0.062841	-9.396148	L
H-H1-0.0976	0	-2.962227	1.317773	-8.537626	L
C-CT--0.3662	-1	8.769061	1.808474	-5.902571	L
H-HC-0.1123	0	9.267272	2.046493	-6.841975	L
H-HC-0.1123	0	9.517377	1.599684	-5.140875	L
H-HC-0.1123	0	8.119786	0.947003	-6.045196	L
C-C-0.5972	0	7.943295	3.001135	-5.474620	L
O-O--0.5679	0	8.081393	4.072621	-6.046185	L
N-N--0.415700	0	7.085879	2.823744	-4.469281	L
H-H-0.271900	0	7.021729	1.899855	-4.065126	L
C-CT-0.021300	-1	6.094623	3.825375	-4.050912	L
H-H1-0.112400	0	6.287835	4.770322	-4.562975	L
C-C-0.597300	0	6.126670	4.088811	-2.543096	L
O-O--0.567900	0	6.390779	3.184711	-1.750076	L
C-CT--0.123100	0	4.719691	3.305410	-4.502374	L
H-H1-0.111200	0	4.502137	2.358427	-4.002436	L
H-H1-0.111200	0	4.737434	3.134739	-5.580428	L
S-SH--0.311900	0	3.411202	4.502778	-4.117990	L
H-HS-0.193300	0	2.399220	3.826564	-4.680946	L
N-N--0.415700	0	5.839249	5.325955	-2.140233	L
H-H-0.271900	0	5.583645	6.006016	-2.843377	L
C-CT--0.025200	-1	5.826244	5.754419	-0.746741	L
H-H1-0.069800	0	6.787980	6.210284	-0.512232	L
H-H1-0.069800	0	5.700823	4.899414	-0.087285	L
C-C-0.597300	0	4.741789	6.786485	-0.469999	L
O-O--0.567900	0	4.835627	7.923632	-0.923564	L
N-N--0.415700	0	3.738755	6.417361	0.322830	L
H-H-0.271900	0	3.786690	5.478343	0.715636	L
C-CT--0.025200	-1	2.689378	7.308211	0.811482	L
H-H1-0.069800	0	1.851219	6.702837	1.143254	L
H-H1-0.069800	0	2.360350	7.982325	0.019955	L
C-C-0.597300	0	3.171125	8.128002	2.006935	L
O-O--0.567900	0	2.570986	8.063463	3.077488	L
N-N--0.4157	0	4.271830	8.863959	1.818398	L
H-H-0.2719	0	4.644944	8.848465	0.874283	L
C-CT--0.1490	-1	5.011721	9.562965	2.860365	L
H-H1-0.0976	0	5.331954	8.853611	3.625405	L
H-H1-0.0976	0	5.886212	10.055607	2.432696	L
H-H1-0.0976	0	4.369420	10.313193	3.325768	L
C-CT--0.3662	-1	-1.521844	9.495339	5.158434	L
H-HC-0.1123	0	-1.741516	10.458540	4.700759	L
H-HC-0.1123	0	-0.585587	9.106973	4.760811	L
H-HC-0.1123	0	-1.448355	9.617199	6.237618	L
C-C-0.5972	0	-2.656519	8.519242	4.835390	L
O-O--0.5679	0	-3.553983	8.871450	4.080148	L
N-N--0.4157	0	-2.618840	7.293678	5.375258	L
H-H-0.2719	0	-1.861011	7.081694	6.009629	L

C-CT--0.0275	-1	-3.618214	6.204751	5.217490	L
H-H1-0.1123	0	-4.613346	6.646416	5.157406	L
C-C-0.5973	0	-3.585641	5.247625	6.438431	L
O-O--0.5679	0	-2.669570	5.317810	7.256186	L
C-CT--0.0050	0	-3.345568	5.411498	3.932441	L
H-HC-0.0339	0	-3.523314	6.058947	3.081320	L
H-HC-0.0339	0	-4.089683	4.618961	3.865734	L
C-C*--0.1415	0	-1.986141	4.782784	3.798398	L
C-CW--0.1638	0	-0.822077	5.435877	3.564317	L
H-H4-0.2062	0	-0.720872	6.506624	3.444483	L
C-CB-0.1243	0	-1.631057	3.370839	3.912024	L
N-NA--0.3418	0	0.223087	4.532998	3.529309	L
H-H-0.3412	0	1.193091	4.792413	3.401700	L
C-CN-0.1380	0	-0.218306	3.247369	3.749085	L
C-CA--0.238	0	-2.358977	2.183427	4.149085	L
H-HA-0.1700	0	-3.430630	2.233405	4.253274	L
C-CA--0.2601	0	0.442860	2.015452	3.835115	L
H-HA-0.1572	0	1.512228	1.955894	3.696349	L
C-CA--0.1972	0	-1.706098	0.939260	4.228157	L
H-HA-0.1447	0	-2.267941	0.025924	4.375035	L
C-CA--0.1134	0	-0.312606	0.855707	4.075137	L
H-HA-0.1417	0	0.165859	-0.113041	4.110026	L
N-N--0.4157	0	-4.572213	4.341197	6.563206	L
H-H-0.2719	0	-5.288561	4.361533	5.856258	L
C-CT--0.1490	-1	-4.670330	3.328274	7.628895	L
H-H1-0.0976	0	-3.854673	2.610624	7.526433	L
H-H1-0.0976	0	-4.587415	3.813175	8.604190	L
H-H1-0.0976	0	-5.625187	2.804091	7.568944	L
C-CT--0.3662	-1	-2.822847	-1.396339	7.297252	L
H-HC-0.1123	0	-3.059172	-2.429749	7.042420	L
H-HC-0.1123	0	-3.150966	-1.191108	8.313802	L
H-HC-0.1123	0	-3.321113	-0.733549	6.592715	L
C-C-0.5972	0	-1.330881	-1.202935	7.194733	L
O-O--0.5679	0	-0.615370	-2.152570	6.927215	L
N-N--0.415700	0	-0.834943	0.001767	7.472565	L
H-H-0.271900	0	-1.492312	0.753584	7.606893	L
C-CT--0.087500	-1	0.607901	0.329303	7.427472	L
H-H1-0.096900	0	0.941518	0.212392	6.396929	L
C-C-0.597300	0	1.450242	-0.623108	8.287148	L
O-O--0.567900	0	2.450226	-1.154386	7.819281	L
C-CT-0.298500	0	0.889800	1.787403	7.865342	L
H-HC--0.029700	0	0.961592	1.823500	8.953021	L
C-CT--0.319200	0	2.226365	2.262024	7.282450	L
H-HC-0.079100	0	2.174658	2.276013	6.193622	L
H-HC-0.079100	0	3.029994	1.593904	7.593407	L
H-HC-0.079100	0	2.451000	3.266399	7.641960	L
C-CT--0.319200	0	-0.199360	2.790201	7.460150	L
H-HC-0.079100	0	-0.405254	2.711127	6.396888	L
H-HC-0.079100	0	0.125016	3.807850	7.680146	L
H-HC-0.079100	0	-1.116168	2.609851	8.019394	L
N-N--0.415700	0	1.027956	-0.872908	9.534398	L
H-H-0.271900	0	0.199347	-0.388752	9.838889	L
C-CT--0.025200	-1	1.638305	-1.860944	10.439294	L
H-H1-0.069800	0	1.103315	-1.870035	11.388249	L
H-H1-0.069800	0	2.673582	-1.577370	10.632466	L

C-C-0.597300	0	1.640748	-3.301365	9.873988	L
O-O--0.567900	0	2.463591	-4.104179	10.296311	L
N-N--0.415700	0	0.735347	-3.635800	8.940476	L
H-H-0.271900	0	0.135938	-2.907562	8.580104	L
C-CT-0.033700	-1	0.639663	-4.929660	8.248906	L
H-H1-0.082300	0	1.046282	-5.698567	8.908717	L
C-C-0.597300	0	1.477283	-4.994265	6.946893	L
O-O--0.567900	0	2.121610	-6.003481	6.673903	L
C-CT--0.182500	0	-0.835584	-5.274234	8.007359	L
H-HC-0.060300	0	-1.376561	-5.273248	8.953792	L
H-HC-0.060300	0	-0.906169	-6.267070	7.561412	L
H-HC-0.060300	0	-1.292121	-4.556421	7.328990	L
N-N--0.415700	0	1.503214	-3.915041	6.157969	L
H-H-0.271900	0	0.863518	-3.167566	6.400945	L
C-CT-0.033700	-1	2.445579	-3.666419	5.054218	L
H-H1-0.082300	0	2.345857	-4.455534	4.312691	L
C-C-0.597300	0	3.917958	-3.633523	5.523985	L
O-O--0.567900	0	4.834443	-3.818016	4.720239	L
C-CT--0.182500	0	2.055809	-2.322368	4.450362	L
H-HC-0.060300	0	2.340358	-1.517540	5.127559	L
H-HC-0.060300	0	0.977527	-2.291065	4.321142	L
H-HC-0.060300	0	2.551045	-2.198870	3.487760	L
N-N--0.4157	0	4.145946	-3.340754	6.810750	L
H-H-0.2719	0	3.332828	-3.065956	7.346302	L
C-CT--0.1490	-1	5.424510	-3.400582	7.503302	L
H-H1-0.0976	0	5.939896	-4.327732	7.245159	L
H-H1-0.0976	0	5.260553	-3.372914	8.581883	L
H-H1-0.0976	0	6.044804	-2.553190	7.207334	L
C-CT--0.3662	-1	14.092649	0.904118	-0.649636	L
H-HC-0.1123	0	13.451869	1.424763	0.059137	L
H-HC-0.1123	0	15.099872	1.316099	-0.608636	L
H-HC-0.1123	0	13.695083	1.006701	-1.657189	L
C-C-0.5972	0	14.148860	-0.570849	-0.287256	L
O-O--0.5679	0	15.230112	-1.131063	-0.170069	L
N-N--0.4157	0	12.987900	-1.206041	-0.129979	L
H-H-0.2719	0	12.145624	-0.657218	-0.257002	L
C-CT--0.0014	-1	12.811352	-2.657826	0.084215	L
H-H1-0.0876	0	13.695643	-3.178784	-0.285552	L
C-C-0.5973	0	11.592489	-3.161075	-0.741582	L
O-O--0.5679	0	10.839672	-2.367255	-1.314861	L
C-CT--0.0152	0	12.662752	-2.966115	1.584812	L
H-HC-0.0295	0	13.608202	-2.729014	2.075177	L
H-HC-0.0295	0	12.500505	-4.036496	1.715976	L
C-CA--0.0011	-1	11.549483	-2.210854	2.290713	L
C-CA--0.1906	0	11.835173	-0.979475	2.910390	L
H-HA-0.1699	0	12.844891	-0.592211	2.893501	L
C-CA--0.1906	0	10.234417	-2.716900	2.314253	L
H-HA-0.1699	0	10.006219	-3.667121	1.852090	L
C-CA--0.2341	0	10.813474	-0.255805	3.551359	L
H-HA-0.1656	0	11.038002	0.675372	4.039757	L
C-CA--0.2341	0	9.207462	-1.990861	2.949863	L
H-HA-0.1656	0	8.201873	-2.383866	2.970114	L
C-C-0.3226	0	9.494592	-0.756562	3.573286	L
O-OH--0.557	0	8.512679	-0.049510	4.195002	L
H-HO-0.3992	0	7.695855	-0.588710	4.246484	L

N-N--0.415700	0	11.379242	-4.480636	-0.837572	L
H-H-0.271900	0	11.993808	-5.092581	-0.323671	L
C-CT--0.025200	-1	10.258894	-5.088783	-1.584708	L
H-H1-0.069800	0	10.475039	-6.143197	-1.755608	L
H-H1-0.069800	0	10.155716	-4.605259	-2.555397	L
C-C-0.597300	0	8.931898	-5.002634	-0.817633	L
O-O--0.567900	0	8.863632	-5.390376	0.347197	L
N-N--0.415700	0	7.863831	-4.518812	-1.457842	L
H-H-0.271900	0	7.948390	-4.397878	-2.459757	L
C-CT-0.014300	-1	6.523147	-4.491197	-0.875416	L
H-H1-0.104800	0	6.612671	-4.379163	0.208104	L
C-C-0.597300	0	5.789841	-5.806703	-1.141466	L
O-O--0.567900	0	5.078175	-5.951277	-2.140945	L
C-CT--0.204100	0	5.707963	-3.275377	-1.378326	L
H-HC-0.079700	0	5.571730	-3.349587	-2.456927	L
H-HC-0.079700	0	6.282026	-2.384318	-1.164028	L
C-C-0.713000	-1	4.326205	-3.128648	-0.717920	L
O-O--0.593100	0	3.969741	-3.827150	0.218366	L
N-N--0.919100	0	3.479621	-2.257316	-1.224750	L
H-H-0.419600	0	2.601924	-2.168604	-0.743740	L
H-H-0.419600	0	3.677118	-1.673494	-2.029059	L
N-N--0.347900	0	5.937346	-6.739561	-0.198148	L
H-H-0.274700	0	6.603387	-6.505710	0.528326	L
C-CT--0.263700	-1	5.377058	-8.096337	-0.241089	L
H-H1-0.156000	0	5.750831	-8.626640	0.635053	L
C-C-0.734100	0	5.883933	-8.909275	-1.442982	L
O-O--0.589400	0	6.893240	-8.577490	-2.058025	L
C-CT--0.000700	0	3.837740	-8.051507	-0.138546	L
H-HC-0.032700	0	3.448554	-9.069321	-0.094312	L
H-HC-0.032700	0	3.435167	-7.581931	-1.037464	L
C-CT-0.039000	0	3.305186	-7.316124	1.096866	L
H-HC-0.028500	0	3.708232	-6.306180	1.150166	L
H-HC-0.028500	0	3.574364	-7.863083	2.001562	L
C-CT-0.048600	0	1.784917	-7.252713	0.952482	L
H-H1-0.068700	0	1.393696	-8.267001	0.854729	L
H-H1-0.068700	0	1.531445	-6.705951	0.040980	L
N-N2--0.529500	-1	1.126971	-6.613570	2.094335	L
H-H-0.345600	0	0.423687	-7.153141	2.580747	L
C-CA-0.807600	0	0.911181	-5.329699	2.238433	L
N-N2--0.862700	0	-0.087891	-4.946576	2.964348	L
H-H-0.447800	0	-0.765166	-5.620317	3.291180	L
H-H-0.447800	0	-0.320690	-3.952359	3.026911	L
N-N2--0.862700	0	1.631645	-4.420047	1.660629	L
H-H-0.447800	0	2.501588	-4.589456	1.175359	L
H-H-0.447800	0	1.289275	-3.467760	1.786663	L
N-N--0.4157	0	5.201306	-10.019523	-1.729651	L
H-H-0.2719	0	4.405871	-10.218867	-1.147658	L
C-CT--0.1490	-1	5.511364	-10.927558	-2.819116	L
H-H1-0.0976	0	6.524520	-11.317156	-2.697494	L
H-H1-0.0976	0	4.806459	-11.760215	-2.832185	L
H-H1-0.0976	0	5.457154	-10.394206	-3.770359	L
C-CT--0.3662	-1	10.178387	-6.054480	-7.646214	L
H-HC-0.1123	0	9.890801	-6.754619	-8.428076	L
H-HC-0.1123	0	11.083342	-5.528680	-7.949278	L
H-HC-0.1123	0	10.365569	-6.582749	-6.712871	L

C-C-0.5972	0	9.071195	-5.043297	-7.438502	L
O-O--0.5679	0	9.302870	-3.848435	-7.587452	L
N-N--0.347900	0	7.872368	-5.491842	-7.037162	L
H-H-0.274700	0	7.786021	-6.486333	-6.890885	L
C-CT--0.263700	-1	6.890738	-4.584265	-6.409543	L
H-H1-0.156000	0	6.733697	-3.727183	-7.065400	L
C-C-0.734100	0	7.518302	-4.068719	-5.111682	L
O-O--0.589400	0	7.869816	-4.861458	-4.243971	L
C-CT--0.000700	0	5.539704	-5.263762	-6.138914	L
H-HC-0.032700	0	4.944815	-4.568675	-5.548820	L
H-HC-0.032700	0	5.682364	-6.164908	-5.538802	L
C-CT-0.039000	0	4.782198	-5.618160	-7.431895	L
H-HC-0.028500	0	5.002131	-6.656077	-7.684964	L
H-HC-0.028500	0	5.131163	-4.993304	-8.255641	L
C-CT-0.048600	0	3.258737	-5.445869	-7.303386	L
H-H1-0.068700	0	2.912834	-5.899461	-6.373920	L
H-H1-0.068700	0	2.786715	-5.988551	-8.124511	L
N-N2--0.529500	0	2.852511	-4.025371	-7.395678	L
H-H-0.345600	0	2.821202	-3.623495	-8.315831	L
C-CA-0.807600	0	2.532574	-3.197847	-6.413961	L
N-N2--0.862700	0	2.484440	-3.537086	-5.164729	L
H-H-0.447800	0	2.687228	-4.480429	-4.850225	L
H-H-0.447800	0	2.256876	-2.798565	-4.494312	L
N-N2--0.862700	0	2.254514	-1.952787	-6.653162	L
H-H-0.447800	0	2.267058	-1.525942	-7.557907	L
H-H-0.447800	0	2.098990	-1.404495	-5.803855	L
N-N--0.415700	0	7.727756	-2.759875	-5.000778	L
H-H-0.271900	0	7.423847	-2.175547	-5.762160	L
C-CT--0.025200	-1	8.631254	-2.169029	-4.009023	L
H-H1-0.069800	0	9.512180	-1.793918	-4.530686	L
H-H1-0.069800	0	8.974716	-2.919123	-3.301342	L
C-C-0.597300	0	8.037687	-1.016145	-3.220005	L
O-O--0.567900	0	6.987514	-0.472915	-3.565900	L
N-N--0.415700	0	8.722596	-0.619090	-2.150974	L
H-H-0.271900	0	9.574637	-1.112680	-1.887877	L
C-CT--0.038900	-1	8.312950	0.523126	-1.339594	L
H-H1-0.100700	0	7.682466	1.145208	-1.971062	L
C-C-0.597300	0	9.448912	1.455024	-0.944066	L
O-O--0.567900	0	10.559156	1.043085	-0.611953	L
C-CT-0.365400	0	7.418645	0.091381	-0.159781	L
H-H1-0.004300	0	7.733752	-0.892086	0.192045	L
O-OH--0.676100	0	6.095981	0.022356	-0.658871	L
H-HO-0.410200	0	6.159511	-0.169632	-1.605937	L
C-CT--0.243800	0	7.351035	1.038294	1.038860	L
H-HC-0.064200	0	6.996363	2.020804	0.728700	L
H-HC-0.064200	0	8.332888	1.125493	1.502863	L
H-HC-0.064200	0	6.660352	0.630901	1.777929	L
N-N--0.415700	0	9.123178	2.746206	-1.014978	L
H-H-0.271900	0	8.154945	2.949233	-1.242910	L
C-CT--0.025200	-1	9.963842	3.879512	-0.680716	L
H-H1-0.069800	0	9.772918	4.686333	-1.388177	L
H-H1-0.069800	0	11.014264	3.593685	-0.757887	L
C-C-0.597300	0	9.663993	4.389778	0.714705	L
O-O--0.567900	0	8.516019	4.616596	1.107599	L
N-N--0.347900	0	10.734920	4.602179	1.462983	L

H-H-0.274700	0	11.642589	4.434605	1.043994	L
C-CT--0.263700	-1	10.739053	5.205897	2.786068	L
H-H1-0.156000	0	9.830750	5.787309	2.934750	L
C-C-0.734100	0	11.908654	6.162909	2.881573	L
O-O--0.589400	0	12.859027	6.073726	2.109504	L
C-CT--0.000700	0	10.891201	4.106192	3.860525	L
H-HC-0.032700	0	11.174415	4.556898	4.813393	L
H-HC-0.032700	0	11.701191	3.434972	3.567049	L
C-CT-0.039000	0	9.626404	3.274462	4.089920	L
H-HC-0.028500	0	9.870752	2.437014	4.742080	L
H-HC-0.028500	0	9.261273	2.878844	3.141888	L
C-CT-0.048600	0	8.540066	4.100951	4.779724	L
H-H1-0.068700	0	8.375266	5.035875	4.244783	L
H-H1-0.068700	0	8.867163	4.343310	5.792572	L
N-N2--0.529500	-1	7.284127	3.350788	4.844177	L
H-H-0.345600	0	7.154016	2.707830	5.606728	L
C-CA-0.807600	0	6.300310	3.395371	3.969441	L
N-N2--0.862700	0	5.213879	2.724396	4.171655	L
H-H-0.447800	0	5.146272	2.004854	4.867443	L
H-H-0.447800	0	4.626466	2.621477	3.340841	L
N-N2--0.862700	0	6.357031	4.086251	2.872108	L
H-H-0.447800	0	7.231074	4.411294	2.483803	L
H-H-0.447800	0	5.521560	4.067620	2.285902	L
N-N--0.4157	0	11.824189	7.055222	3.858738	L
H-H-0.2719	0	11.007044	7.047295	4.445190	L
C-CT--0.1490	-1	12.888555	7.983990	4.141627	L
H-H1-0.0976	0	12.622261	8.623273	4.984923	L
H-H1-0.0976	0	13.804937	7.439759	4.382032	L
H-H1-0.0976	0	13.079509	8.609788	3.266605	L
C-CT--0.3662	-1	-2.160012	13.213940	-1.713521	L
H-HC-0.1123	0	-2.576445	13.732487	-0.852422	L
H-HC-0.1123	0	-1.072982	13.199492	-1.664783	L
H-HC-0.1123	0	-2.469620	13.725739	-2.624486	L
C-C-0.5972	0	-2.685337	11.790710	-1.756027	L
O-O--0.5679	0	-3.343532	11.409677	-2.711257	L
N-N--0.415700	0	-2.403305	11.015380	-0.705642	L
H-H-0.271900	0	-1.836829	11.441311	0.008574	L
C-CT--0.023700	-1	-2.830391	9.613056	-0.512401	L
H-H1-0.088000	0	-2.356121	9.003239	-1.282925	L
C-C-0.597300	0	-4.349407	9.432174	-0.683232	L
O-O--0.567900	0	-4.808898	8.650626	-1.516582	L
C-CT-0.034200	0	-2.306544	9.163601	0.857621	L
H-HC-0.024100	0	-2.681913	9.837420	1.629102	L
H-HC-0.024100	0	-1.218144	9.231063	0.857284	L
C-CT-0.001800	0	-2.705669	7.740586	1.232583	L
H-H1-0.044000	0	-3.775625	7.731400	1.443524	L
H-H1-0.044000	0	-2.184195	7.492828	2.153025	L
S-S--0.273700	0	-2.369779	6.439844	0.020998	L
C-CT--0.053600	0	-0.571713	6.488863	-0.102163	L
H-H1-0.068400	0	-0.246483	5.781173	-0.864441	L
H-H1-0.068400	0	-0.150231	6.205599	0.860058	L
H-H1-0.068400	0	-0.241552	7.488431	-0.382585	L
N-N--0.4157	0	-5.135867	10.168948	0.106087	L
H-H-0.2719	0	-4.680804	10.781821	0.763334	L
C-CT--0.1490	-1	-6.586074	10.190535	0.001192	L

H-H1-0.0976	0	-7.008577	10.845268	0.764705	L
H-H1-0.0976	0	-6.875963	10.556750	-0.985984	L
H-H1-0.0976	0	-6.980628	9.181587	0.134083	L
C-CT--0.3662	-1	-4.427594	8.901458	-5.259185	L
H-HC-0.1123	0	-3.994870	9.253187	-4.323329	L
H-HC-0.1123	0	-3.661149	8.889746	-6.032141	L
H-HC-0.1123	0	-5.240836	9.556012	-5.564172	L
C-C-0.5972	0	-4.963163	7.488885	-5.077090	L
O-O--0.5679	0	-4.683828	6.621169	-5.891693	L
N-N--0.415700	0	-5.741330	7.260344	-4.015576	L
H-H-0.271900	0	-5.748169	8.011423	-3.336798	L
C-CT--0.087500	-1	-6.382023	5.965399	-3.659076	L
H-H1-0.096900	0	-6.106254	5.215841	-4.400435	L
C-C-0.597300	0	-7.900835	6.088506	-3.710381	L
O-O--0.567900	0	-8.557729	5.284929	-4.360239	L
C-CT-0.298500	0	-5.871536	5.496947	-2.286871	L
H-HC--0.029700	0	-6.028424	6.284404	-1.549781	L
C-CT--0.319200	0	-6.558906	4.231439	-1.772418	L
H-HC-0.079100	0	-6.427153	3.433030	-2.498527	L
H-HC-0.079100	0	-7.615464	4.428272	-1.600454	L
H-HC-0.079100	0	-6.112313	3.931316	-0.826342	L
C-CT--0.319200	0	-4.379032	5.194449	-2.390177	L
H-HC-0.079100	0	-4.198865	4.452871	-3.162297	L
H-HC-0.079100	0	-4.030220	4.811449	-1.441043	L
H-HC-0.079100	0	-3.819739	6.100116	-2.623946	L
N-N--0.4157	0	-8.472720	7.083548	-3.024516	L
H-H-0.2719	0	-7.853083	7.713676	-2.539848	L
C-CT--0.1490	-1	-9.910522	7.333474	-3.031546	L
H-H1-0.0976	0	-10.148726	8.196883	-2.409463	L
H-H1-0.0976	0	-10.242389	7.524877	-4.054543	L
H-H1-0.0976	0	-10.440130	6.456144	-2.655751	L
C-CT-0.1200	-1	-8.874985	1.989414	3.197965	L
H-HC-0.0800	0	-8.091990	2.743137	3.246289	L
H-HC-0.0800	0	-9.713903	2.391303	2.630184	L
H-HC-0.0800	0	-9.215939	1.755880	4.207500	L
C-CT-0.2000	0	-8.389300	0.750541	2.536427	L
H-H1-0.0800	0	-9.191188	0.008248	2.488759	L
O-OS--0.5600	0	-7.945642	1.095467	1.244553	L
C-CT-0.2000	0	-7.196268	0.208408	3.309567	L
H-H1-0.0800	0	-6.492630	1.000587	3.567555	L
O-OH--0.6800	0	-7.575481	-0.521050	4.469465	L
H-HO-0.4000	0	-8.204950	-1.199677	4.181306	L
C-CT-0.2000	0	-6.632011	-0.623985	2.178372	L
H-H1-0.0800	0	-5.581777	-0.856418	2.355894	L
O-OH--0.6800	0	-7.425617	-1.804493	2.079406	L
H-HO-0.4000	0	-7.000882	-2.402547	1.432926	L
C-CT-0.5691	0	-6.810613	0.277601	0.943753	H
H-H2-0.8000	0	-6.964505	-0.374144	0.057304	H
N-N*--0.5691	0	-5.682774	1.174406	0.721137	H
C-CM--0.0500	0	-4.982939	0.999411	-0.429904	H
H-H4-0.1500	0	-5.399248	0.234062	-1.047690	H
C-CM--0.1238	0	-3.921369	1.717858	-0.799348	H
C-C-0.6156	0	-3.427889	1.688290	-2.187732	H
O-O--0.5700	0	-2.518453	2.393234	-2.554168	H
N-N--0.8000	0	-4.109630	0.933536	-3.096628	H

H-H-0.3700	0	-3.665462	0.705648	-3.963452	H
H-H-0.3700	0	-4.746200	0.214212	-2.806636	H
C-CT-0.1164	0	-3.284394	2.639806	0.213471	H
H-HC-0.0800	0	-2.809688	3.478816	-0.274030	H
H-HC-0.0800	0	-2.492109	2.123544	0.749363	H
C-CM--0.2882	0	-4.347654	3.086191	1.187627	H
H-HA-0.1500	0	-4.221641	4.028546	1.681300	H
C-CM--0.0500	0	-5.419306	2.353646	1.431515	H
H-H4-0.1500	0	-6.162154	2.665241	2.131708	H
N-N3--0.8530	0	3.249140	0.923933	-0.389035	H
H-H-0.4500	0	3.383984	0.637190	-1.345470	H
H-H-0.4500	0	2.056263	0.462099	0.872797	H
H-H-0.4500	0	4.087859	0.682841	0.119702	H
C-CT-0.3170	0	3.422660	2.378698	-0.368490	H
H-HP-0.0800	0	4.232592	2.666303	-1.031812	H
C-CT--0.1600	0	2.181856	3.184367	-0.751144	H
H-HC-0.0800	0	1.956296	2.998876	-1.795187	H
H-HC-0.0800	0	2.455012	4.229473	-0.680619	H
C-CT--0.1600	0	0.954362	2.901966	0.120569	H
H-HC-0.0800	0	0.280000	3.744949	0.085250	H
H-HC-0.0800	0	1.283596	2.817490	1.151166	H
C-CT--0.0990	0	0.203829	1.635518	-0.310509	H
H-HC-0.0800	0	0.830647	1.031439	-0.951593	H
H-HC-0.0800	0	-0.667663	1.899632	-0.892203	H
C-C-0.4490	0	-0.245033	0.755721	0.825888	H
H-H-0.0600	0	-0.518181	1.236787	1.745350	H
O-O--0.5700	0	1.311874	0.214881	1.469505	H
N-N3--0.9900	0	-0.978588	-0.303161	0.501633	H
H-H-0.3600	0	1.169454	-0.751396	1.567684	H
H-H-0.3600	0	-0.893913	-0.576035	-0.462068	H
C-C-0.9060	0	3.919753	2.705178	1.043260	L
O-O2--0.9000	0	4.122791	1.790060	1.877351	L
O-O2--0.9000	0	4.103204	3.892846	1.386552	L
C-CT--0.205900	0	-1.768647	-1.139417	1.402323	H
H-H1-0.139900	0	-2.384961	-0.475838	1.997635	H
C-CT-0.007100	0	-2.675430	-1.970886	0.495642	H
H-HC--0.007800	0	-2.077636	-2.614271	-0.139610	H
H-HC--0.007800	0	-3.222670	-1.288862	-0.146489	H
C-CT-0.067500	0	-3.660144	-2.840346	1.259579	L
H-HC--0.054800	0	-3.948684	-2.323229	2.175347	L
H-HC--0.054800	0	-3.179340	-3.782200	1.531635	L
C-C-0.818300	0	-4.930310	-3.097577	0.453483	L
O-O2--0.822000	0	-5.636865	-2.110883	0.130854	L
O-O2--0.822000	0	-5.332947	-4.269323	0.244963	L
C-C-0.742000	0	-0.893365	-2.007660	2.322525	H
O-O2--0.793000	0	-1.436973	-2.541669	3.276844	H
O-O2--0.793000	0	0.294113	-2.143157	1.963716	H
O-OW--0.834000	0	3.020353	-5.973073	-3.834383	L
H-HW-0.417000	0	3.816642	-6.063059	-3.271568	L
H-HW-0.417000	0	2.320005	-5.789055	-3.188823	L
O-OW--0.834000	0	-1.763134	-7.330817	2.481910	L
H-HW-0.417000	0	-2.491083	-6.868186	2.018285	L
H-HW-0.417000	0	-2.122965	-8.213526	2.592447	L
O-OW--0.834000	0	4.011427	0.070648	3.753648	L
H-HW-0.417000	0	3.120125	-0.272190	3.644824	L

H-HW-0.417000 0 4.097611 0.693183 2.987153 L
O-OW--0.834000 0 6.192877 -1.414280 4.357225 L
H-HW-0.417000 0 5.847552 -2.315688 4.488142 L
H-HW-0.417000 0 5.384014 -0.905464 4.149830 L
O-OW--0.834000 0 -4.148313 -6.348269 1.479682 L
H-HW-0.417000 0 -4.590581 -5.671523 0.926219 L
H-HW-0.417000 0 -4.583322 -6.220138 2.331998 L
O-OW--0.834000 0 -9.056672 -1.329651 -0.213212 L
H-HW-0.417000 0 -9.902214 -1.399044 0.234651 L
H-HW-0.417000 0 -8.716331 -2.248061 -0.170321 L

I5
C-CT--0.3662 -1 -12.877402 1.159600 1.898223 L
H-HC-0.1123 0 -12.382120 2.089420 1.622816 L
H-HC-0.1123 0 -13.949718 1.266901 1.743625 L
H-HC-0.1123 0 -12.487941 0.345284 1.290818 L
C-C-0.5972 0 -12.605387 0.867777 3.361543 L
O-O--0.5679 0 -11.835005 1.589192 3.984813 L
N-N--0.415700 0 -13.213144 -0.197028 3.894734 L
H-H-0.271900 0 -13.817154 -0.726150 3.287281 L
C-CT--0.025200 -1 -12.922385 -0.742462 5.233922 L
H-H1-0.069800 0 -13.675707 -1.486979 5.490716 L
H-H1-0.069800 0 -12.958510 0.064321 5.966650 L
C-C-0.597300 0 -11.516244 -1.409456 5.304000 L
O-O--0.567900 0 -10.707967 -1.307005 4.375992 L
N-N--0.415700 0 -11.209201 -2.126402 6.396495 L
H-H-0.271900 0 -11.909916 -2.205006 7.116291 L
C-CT--0.025200 -1 -10.027719 -3.013968 6.487787 L
H-H1-0.069800 0 -9.941176 -3.406975 7.500430 L
H-H1-0.069800 0 -9.125745 -2.449642 6.246712 L
C-C-0.597300 0 -10.182985 -4.183858 5.526180 L
O-O--0.567900 0 -11.230454 -4.822825 5.477398 L
N-N--0.4157 0 -9.129312 -4.460028 4.770903 L
H-H-0.2719 0 -8.296482 -3.910833 4.914562 L
C-CT--0.0014 -1 -9.061660 -5.416824 3.666183 L
H-H1-0.0876 0 -9.893664 -5.252899 2.980542 L
C-C-0.5973 0 -9.076557 -6.904857 4.075552 L
O-O--0.5679 0 -8.514188 -7.755010 3.385825 L
C-CT--0.0152 0 -7.747931 -5.097617 2.940178 L
H-HC-0.0295 0 -7.886216 -4.134124 2.470864 L
H-HC-0.0295 0 -7.593651 -5.802613 2.122441 L
C-CA--0.0011 0 -6.475904 -5.024895 3.785844 L
C-CA--0.1906 0 -5.704581 -6.177968 4.031550 L
H-HA-0.1699 0 -6.067279 -7.150164 3.724935 L
C-CA--0.1906 0 -5.995986 -3.775441 4.228618 L
H-HA-0.1699 0 -6.582137 -2.884057 4.107277 L
C-CA--0.2341 0 -4.411207 -6.062440 4.580354 L
H-HA-0.1656 0 -3.792261 -6.937782 4.712545 L
C-CA--0.2341 0 -4.692149 -3.649225 4.736539 L
H-HA-0.1656 0 -4.274409 -2.677384 4.952266 L
C-C-0.3326 0 -3.874627 -4.786923 4.853555 L
O-OH--0.5579 0 -2.553188 -4.640075 5.119185 L
H-HO-0.3992 0 -2.249899 -3.770111 4.769223 L
N-N--0.4157 0 -9.704026 -7.235652 5.203359 L
H-H-0.2719 0 -10.253244 -6.506855 5.641490 L

C-CT--0.1490	-1	-9.659280	-8.563649	5.784708	L
H-H1-0.0976	0	-8.626240	-8.832673	6.013132	L
H-H1-0.0976	0	-10.252095	-8.593875	6.699681	L
H-H1-0.0976	0	-10.059487	-9.289539	5.073782	L
C-CT--0.3662	-1	-12.030653	-1.436005	-3.467352	L
H-HC-0.1123	0	-12.520790	-0.473541	-3.603291	L
H-HC-0.1123	0	-12.039111	-1.726981	-2.419358	L
H-HC-0.1123	0	-12.552345	-2.189687	-4.056516	L
C-C-0.5972	0	-10.599802	-1.345098	-3.957102	L
O-O--0.5679	0	-10.178656	-2.092629	-4.829506	L
N-N--0.415700	0	-9.826209	-0.443932	-3.363639	L
H-H-0.271900	0	-10.226263	0.097133	-2.613184	L
C-CT--0.038900	-1	-8.422522	-0.118553	-3.700246	L
H-H1-0.100700	0	-8.299845	-0.089947	-4.780946	L
C-C-0.597300	0	-7.438735	-1.142099	-3.119183	L
O-O--0.567900	0	-6.341109	-0.804577	-2.681148	L
C-CT-0.365400	0	-8.118040	1.277037	-3.126574	L
H-H1-0.004300	0	-7.122761	1.571933	-3.440215	L
O-OH--0.676100	0	-8.163480	1.285326	-1.714189	L
H-HO-0.410200	0	-7.990448	2.192038	-1.440862	L
C-CT--0.243800	0	-9.124056	2.340448	-3.568759	L
H-HC-0.064200	0	-10.135820	2.095713	-3.250252	L
H-HC-0.064200	0	-9.106464	2.445250	-4.651716	L
H-HC-0.064200	0	-8.864657	3.295124	-3.127072	L
N-N--0.415700	0	-7.841987	-2.405964	-3.066304	L
H-H-0.271900	0	-8.682167	-2.633567	-3.586080	L
C-CT--0.024900	-1	-7.240534	-3.450647	-2.238104	L
H-H1-0.084300	0	-6.415758	-3.053147	-1.676724	L
C-C-0.597300	0	-6.680592	-4.587246	-3.090845	L
O-O--0.567900	0	-7.079171	-4.770352	-4.238072	L
C-CT-0.211700	0	-8.322647	-4.094395	-1.353474	L
H-H1-0.035200	0	-8.286884	-5.183463	-1.425988	L
H-H1-0.035200	0	-9.316173	-3.770817	-1.668004	L
O-OH--0.654600	0	-8.109798	-3.742260	-0.014191	L
H-HO-0.427500	0	-7.185228	-4.034556	0.178414	L
N-N--0.4157	0	-5.804133	-5.407721	-2.512923	L
H-H-0.2719	0	-5.552854	-5.223677	-1.545458	L
C-CT--0.0014	0	-5.401680	-6.668668	-3.140478	L
H-H1-0.0876	0	-5.037265	-6.453634	-4.145350	L
C-C-0.5973	0	-6.552866	-7.674530	-3.256663	L
O-O--0.5679	0	-7.499929	-7.663019	-2.471541	L
C-CT--0.0152	0	-4.282023	-7.328369	-2.341528	L
H-HC-0.0295	0	-4.073703	-8.312291	-2.763817	L
H-HC-0.0295	0	-4.620471	-7.491309	-1.320046	L
C-CA--0.0011	-1	-2.989838	-6.558767	-2.335269	L
C-CA--0.1906	0	-2.103388	-6.651700	-3.429499	L
H-HA-0.1699	0	-2.384530	-7.223181	-4.302420	L
C-CA--0.1906	0	-2.600242	-5.829486	-1.194635	L
H-HA-0.1699	0	-3.259436	-5.757177	-0.343877	L
C-CA--0.2341	0	-0.838972	-6.027807	-3.377462	L
H-HA-0.1656	0	-0.152619	-6.099993	-4.206974	L
C-CA--0.2341	0	-1.335150	-5.217427	-1.130853	L
H-HA-0.1656	0	-1.039323	-4.691906	-0.234970	L
C-C-0.3226	0	-0.452316	-5.313282	-2.223666	L
O-OH--0.5579	0	0.767350	-4.716969	-2.156591	L

H-HO-0.3992	0	0.723508	-4.020375	-1.493189	L
N-N--0.4157	0	-6.397882	-8.610433	-4.198460	L
H-H-0.2719	0	-5.599815	-8.522576	-4.805357	L
C-CT--0.1490	-1	-7.239650	-9.787409	-4.324485	L
H-H1-0.0976	0	-8.272312	-9.480147	-4.503918	L
H-H1-0.0976	0	-6.901532	-10.410608	-5.153247	L
H-H1-0.0976	0	-7.207485	-10.365805	-3.398873	L
C-CT--0.3662	-1	-6.378144	-3.589013	-6.922092	L
H-HC-0.1123	0	-5.923866	-4.371602	-7.525849	L
H-HC-0.1123	0	-7.417872	-3.843994	-6.721269	L
H-HC-0.1123	0	-5.835723	-3.485661	-5.988542	L
C-C-0.5972	0	-6.299708	-2.269961	-7.674136	L
O-O--0.5679	0	-5.406280	-2.116956	-8.495966	L
N-N--0.415700	0	-7.203359	-1.322059	-7.389444	L
H-H-0.271900	0	-7.919513	-1.577944	-6.725624	L
C-CT--0.025200	-1	-7.301872	0.007971	-8.039039	L
H-H1-0.069800	0	-8.229789	0.051499	-8.609397	L
H-H1-0.069800	0	-6.480478	0.156023	-8.738842	L
C-C-0.597300	0	-7.309673	1.166268	-7.036739	L
O-O--0.567900	0	-8.285919	1.902162	-6.968861	L
N-N--0.415700	0	-6.233974	1.318483	-6.258691	L
H-H-0.271900	0	-5.502991	0.620353	-6.344635	L
C-CT--0.025200	-1	-5.819239	2.573466	-5.618129	L
H-H1-0.069800	0	-5.630881	2.416432	-4.561460	L
H-H1-0.069800	0	-6.592097	3.334695	-5.736057	L
C-C-0.597300	0	-4.535718	3.082060	-6.284371	L
O-O--0.567900	0	-4.483321	3.211214	-7.504699	L
N-N--0.415700	0	-3.484262	3.302851	-5.491802	L
H-H-0.271900	0	-3.603924	3.149788	-4.501676	L
C-CT--0.051800	-1	-2.142747	3.672659	-5.958971	L
H-H1-0.092200	0	-2.220882	4.102715	-6.959014	L
C-C-0.597300	0	-1.237709	2.427639	-6.055126	L
O-O--0.567900	0	-0.567379	2.186919	-7.056167	L
C-CT--0.110200	0	-1.591795	4.747024	-5.000726	L
H-HC-0.045700	0	-1.586476	4.345381	-3.987764	L
H-HC-0.045700	0	-2.272254	5.599828	-5.014215	L
C-CT-0.353100	0	-0.173749	5.246025	-5.324014	L
H-HC--0.036100	0	0.523459	4.408559	-5.298270	L
C-CT--0.412100	0	-0.087008	5.924177	-6.691203	L
H-HC-0.100000	0	-0.820003	6.729169	-6.756208	L
H-HC-0.100000	0	-0.285738	5.196570	-7.478135	L
H-HC-0.100000	0	0.912740	6.329200	-6.843303	L
C-CT--0.412100	0	0.253126	6.258098	-4.258404	L
H-HC-0.100000	0	-0.431976	7.106728	-4.257706	L
H-HC-0.100000	0	1.262415	6.611301	-4.467881	L
H-HC-0.100000	0	0.240027	5.783486	-3.278068	L
N-N--0.516300	0	-1.193296	1.643527	-4.980691	L
H-H-0.293600	0	-1.732314	1.958361	-4.180203	L
C-CT-0.038100	-1	-0.312107	0.500137	-4.754763	L
H-H1-0.088000	0	-0.080052	-0.005980	-5.689595	L
C-C-0.536600	0	-0.981516	-0.477250	-3.753043	L
O-O--0.581900	0	-0.787862	-0.344432	-2.546492	L
C-CT--0.030300	0	0.995308	1.088270	-4.152031	L
H-HC--0.012200	0	0.740266	1.716258	-3.297378	L
H-HC--0.012200	0	1.457334	1.734397	-4.900703	L

C-C-0.799400	-1	2.049381	0.070084	-3.696178	L
O-O2--0.801400	0	1.881965	-1.139796	-3.979468	L
O-O2--0.801400	0	3.144280	0.481234	-3.259368	L
N-N--0.254800	0	-1.791097	-1.450208	-4.204664	L
C-CT--0.026600	-1	-2.078293	-1.774617	-5.597630	L
H-H1-0.064100	0	-1.150649	-1.865705	-6.161361	L
C-C-0.589600	0	-2.987850	-0.741797	-6.286943	L
O-O--0.574800	0	-3.855767	-0.147440	-5.650141	L
C-CT--0.007000	0	-2.755171	-3.148239	-5.537494	L
H-HC-0.025300	0	-1.992017	-3.927906	-5.518330	L
H-HC-0.025300	0	-3.437797	-3.311096	-6.371891	L
C-CT-0.018900	0	-3.484324	-3.124463	-4.194525	L
H-HC-0.021300	0	-3.655637	-4.127871	-3.809852	L
H-HC-0.021300	0	-4.424179	-2.578322	-4.286183	L
C-CT-0.019200	0	-2.523051	-2.337716	-3.310153	L
H-H1-0.039100	0	-3.075996	-1.774323	-2.556978	L
H-H1-0.039100	0	-1.822467	-3.014992	-2.824335	L
N-N--0.4157	0	-2.791333	-0.541454	-7.593381	L
H-H-0.2719	0	-2.079860	-1.086172	-8.049282	L
C-CT--0.1490	-1	-3.552723	0.381421	-8.434727	L
H-H1-0.0976	0	-4.509023	0.616986	-7.983330	L
H-H1-0.0976	0	-3.737197	-0.069061	-9.410971	L
H-H1-0.0976	0	-2.994619	1.311185	-8.566459	L
C-CT--0.3662	-1	8.746886	1.719709	-5.971278	L
H-HC-0.1123	0	9.246569	1.953722	-6.910999	L
H-HC-0.1123	0	9.494184	1.496064	-5.212764	L
H-HC-0.1123	0	8.083782	0.869294	-6.116552	L
C-C-0.5972	0	7.941276	2.924470	-5.536711	L
O-O--0.5679	0	8.096684	3.997002	-6.101521	L
N-N--0.415700	0	7.080316	2.756214	-4.532838	L
H-H-0.271900	0	7.000586	1.832071	-4.133263	L
C-CT-0.021300	-1	6.095457	3.765272	-4.117973	L
H-H1-0.112400	0	6.298387	4.710839	-4.625118	L
C-C-0.597300	0	6.120876	4.023783	-2.607679	L
O-O--0.567900	0	6.332835	3.109268	-1.808665	L
C-CT--0.123100	0	4.720341	3.256816	-4.581782	L
H-H1-0.111200	0	4.545579	2.261498	-4.170060	L
H-H1-0.111200	0	4.713309	3.185631	-5.671107	L
S-SH--0.311900	0	3.380293	4.360535	-4.048158	L
H-HS-0.193300	0	2.377034	3.689228	-4.632829	L
N-N--0.415700	0	5.880425	5.273631	-2.211216	L
H-H-0.271900	0	5.667251	5.962052	-2.920313	L
C-CT--0.025200	-1	5.854520	5.709218	-0.820427	L
H-H1-0.069800	0	6.814262	6.166488	-0.580181	L
H-H1-0.069800	0	5.723746	4.858024	-0.156924	L
C-C-0.597300	0	4.766663	6.743928	-0.554617	L
O-O--0.567900	0	4.874049	7.883907	-0.998065	L
N-N--0.415700	0	3.747864	6.376755	0.221477	L
H-H-0.271900	0	3.789202	5.442459	0.624493	L
C-CT--0.025200	-1	2.735895	7.294281	0.742909	L
H-H1-0.069800	0	1.867395	6.719336	1.052563	L
H-H1-0.069800	0	2.440337	8.015501	-0.020016	L
C-C-0.597300	0	3.268368	8.033955	1.966299	L
O-O--0.567900	0	2.768217	7.829870	3.071747	L
N-N--0.4157	0	4.306731	8.850138	1.751023	L

H-H-0.2719	0	4.614715	8.906785	0.785026	L
C-CT--0.1490	-1	5.083678	9.538169	2.774657	L
H-H1-0.0976	0	5.471196	8.814256	3.493615	L
H-H1-0.0976	0	5.915835	10.074580	2.316015	L
H-H1-0.0976	0	4.446278	10.249675	3.303165	L
C-CT--0.3662	-1	-1.441895	9.532130	5.096298	L
H-HC-0.1123	0	-1.397679	10.303037	4.328987	L
H-HC-0.1123	0	-0.505444	8.977478	5.112494	L
H-HC-0.1123	0	-1.614463	9.997798	6.064961	L
C-C-0.5972	0	-2.607490	8.585974	4.786545	L
O-O--0.5679	0	-3.440796	8.910995	3.950951	L
N-N--0.4157	0	-2.645015	7.402981	5.412005	L
H-H-0.2719	0	-1.940785	7.224486	6.115307	L
C-CT--0.0275	-1	-3.564452	6.258803	5.175703	L
H-H1-0.1123	0	-4.576123	6.629429	5.009265	L
C-C-0.5973	0	-3.569449	5.329138	6.418337	L
O-O--0.5679	0	-2.709469	5.457935	7.288786	L
C-CT--0.0050	0	-3.090662	5.462406	3.939705	L
H-HC-0.0339	0	-3.362570	6.026906	3.053994	L
H-HC-0.0339	0	-3.668257	4.540109	3.890042	L
C-C*--0.1415	0	-1.625740	5.096185	3.846353	L
C-CW--0.1638	0	-0.590604	5.961427	3.703546	L
H-H4-0.2062	0	-0.676378	7.035708	3.630462	L
C-CB-0.1243	0	-1.011030	3.767962	3.871430	L
N-NA--0.3418	0	0.609198	5.280105	3.698614	L
H-H-0.3412	0	1.512605	5.741092	3.646838	L
C-CN-0.1380	0	0.409332	3.922956	3.818502	L
C-CA--0.238	0	-1.504240	2.445744	3.944065	L
H-HA-0.1700	0	-2.569375	2.274015	3.951543	L
C-CA--0.2601	0	1.291607	2.834887	3.883082	L
H-HA-0.1572	0	2.356527	3.002254	3.845526	L
C-CA--0.1972	0	-0.627343	1.344521	3.996224	L
H-HA-0.1447	0	-1.025309	0.337748	4.040015	L
C-CA--0.1134	0	0.765243	1.534131	3.971952	L
H-HA-0.1417	0	1.417862	0.671949	4.003968	L
N-N--0.4157	0	-4.517930	4.380159	6.507372	L
H-H-0.2719	0	-5.187688	4.339815	5.756918	L
C-CT--0.1490	-1	-4.630986	3.400276	7.602073	L
H-H1-0.0976	0	-3.741756	2.768158	7.620588	L
H-H1-0.0976	0	-4.704861	3.924459	8.557476	L
H-H1-0.0976	0	-5.515745	2.776847	7.464955	L
C-CT--0.3662	-1	-2.822792	-1.340328	7.282296	L
H-HC-0.1123	0	-3.054735	-2.404283	7.233156	L
H-HC-0.1123	0	-3.182050	-0.931632	8.223839	L
H-HC-0.1123	0	-3.292987	-0.838825	6.438244	L
C-C-0.5972	0	-1.329737	-1.167010	7.176754	L
O-O--0.5679	0	-0.622250	-2.134013	6.959281	L
N-N--0.415700	0	-0.823502	0.042093	7.414491	L
H-H-0.271900	0	-1.478241	0.800304	7.518639	L
C-CT--0.087500	-1	0.622180	0.358094	7.393514	L
H-H1-0.096900	0	0.969934	0.273813	6.364896	L
C-C-0.597300	0	1.451283	-0.615055	8.243349	L
O-O--0.567900	0	2.441348	-1.160732	7.769219	L
C-CT-0.298500	0	0.900241	1.797249	7.897924	L
H-HC--0.029700	0	0.854529	1.802681	8.987576	L

C-CT--0.319200	0	2.304816	2.253297	7.482915	L
H-HC-0.079100	0	2.386753	2.265789	6.397083	L
H-HC-0.079100	0	3.057389	1.577134	7.888977	L
H-HC-0.079100	0	2.498125	3.254877	7.867518	L
C-CT--0.319200	0	-0.110827	2.845526	7.417395	L
H-HC-0.079100	0	-0.170458	2.832531	6.334964	L
H-HC-0.079100	0	0.198657	3.840620	7.738396	L
H-HC-0.079100	0	-1.097178	2.656260	7.837350	L
N-N--0.415700	0	1.036599	-0.849542	9.496389	L
H-H-0.271900	0	0.214383	-0.354927	9.801477	L
C-CT--0.025200	-1	1.645771	-1.828692	10.410171	L
H-H1-0.069800	0	1.112442	-1.826907	11.360095	L
H-H1-0.069800	0	2.682568	-1.547135	10.597674	L
C-C-0.597300	0	1.640560	-3.274184	9.857079	L
O-O--0.567900	0	2.459047	-4.079229	10.284173	L
N-N--0.415700	0	0.733333	-3.606285	8.925431	L
H-H-0.271900	0	0.138178	-2.874283	8.564597	L
C-CT-0.033700	-1	0.614580	-4.897731	8.235374	L
H-H1-0.082300	0	1.043550	-5.665422	8.882208	L
C-C-0.597300	0	1.413040	-4.972087	6.908276	L
O-O--0.567900	0	2.014735	-6.000324	6.607701	L
C-CT--0.182500	0	-0.867585	-5.244891	8.054863	L
H-HC-0.060300	0	-1.358786	-5.286060	9.027139	L
H-HC-0.060300	0	-0.956955	-6.218655	7.571730	L
H-HC-0.060300	0	-1.361165	-4.500192	7.435550	L
N-N--0.415700	0	1.468855	-3.884196	6.132355	L
H-H-0.271900	0	0.853984	-3.118850	6.385000	L
C-CT-0.033700	-1	2.419076	-3.661489	5.029341	L
H-H1-0.082300	0	2.287292	-4.435387	4.277674	L
C-C-0.597300	0	3.889226	-3.690355	5.501958	L
O-O--0.567900	0	4.800546	-3.924679	4.705390	L
C-CT--0.182500	0	2.107169	-2.283340	4.438214	L
H-HC-0.060300	0	2.527336	-1.501197	5.070541	L
H-HC-0.060300	0	1.030976	-2.131998	4.396225	L
H-HC-0.060300	0	2.529520	-2.212404	3.436987	L
N-N--0.4157	0	4.126936	-3.366335	6.779133	L
H-H-0.2719	0	3.320699	-3.054343	7.306050	L
C-CT--0.1490	-1	5.408858	-3.410178	7.466712	L
H-H1-0.0976	0	6.030037	-4.205783	7.050430	L
H-H1-0.0976	0	5.247083	-3.607505	8.528014	L
H-H1-0.0976	0	5.924346	-2.455605	7.352503	L
C-CT--0.3662	-1	14.081921	0.792848	-0.733902	L
H-HC-0.1123	0	13.471846	1.329001	-0.009920	L
H-HC-0.1123	0	15.095850	1.190230	-0.728617	L
H-HC-0.1123	0	13.653992	0.892678	-1.729278	L
C-C-0.5972	0	14.128163	-0.679547	-0.359043	L
O-O--0.5679	0	15.205299	-1.245791	-0.234508	L
N-N--0.4157	0	12.962687	-1.305811	-0.199374	L
H-H-0.2719	0	12.125221	-0.752157	-0.334834	L
C-CT--0.0014	-1	12.774652	-2.755780	0.018435	L
H-H1-0.0876	0	13.654254	-3.284942	-0.350967	L
C-C-0.5973	0	11.550672	-3.250495	-0.805016	L
O-O--0.5679	0	10.803233	-2.451273	-1.378404	L
C-CT--0.0152	0	12.620754	-3.064557	1.516941	L
H-HC-0.0295	0	13.571414	-2.847030	2.006353	L

H-HC-0.0295	0	12.439236	-4.132050	1.646444	L
C-CA--0.0011	-1	11.524381	-2.290092	2.227664	L
C-CA--0.1906	0	11.842877	-1.063327	2.840447	L
H-HA-0.1699	0	12.857416	-0.690476	2.799150	L
C-CA--0.1906	0	10.206785	-2.783229	2.285335	L
H-HA-0.1699	0	9.956653	-3.731254	1.829886	L
C-CA--0.2341	0	10.851743	-0.332480	3.517841	L
H-HA-0.1656	0	11.103428	0.589744	4.010515	L
C-CA--0.2341	0	9.210562	-2.050794	2.959414	L
H-HA-0.1656	0	8.204405	-2.437982	3.012874	L
C-C-0.3226	0	9.532278	-0.825254	3.584143	L
O-OH--0.557	0	8.584103	-0.125273	4.260928	L
H-HO-0.3992	0	7.831665	-0.722486	4.454125	L
N-N--0.415700	0	11.326671	-4.568746	-0.895995	L
H-H-0.271900	0	11.938401	-5.183183	-0.381576	L
C-CT--0.025200	-1	10.196715	-5.172535	-1.631846	L
H-H1-0.069800	0	10.400763	-6.230952	-1.792716	L
H-H1-0.069800	0	10.094477	-4.697886	-2.606921	L
C-C-0.597300	0	8.874515	-5.063983	-0.859545	L
O-O--0.567900	0	8.810037	-5.431760	0.311849	L
N-N--0.415700	0	7.805950	-4.583165	-1.501411	L
H-H-0.271900	0	7.886012	-4.478057	-2.505667	L
C-CT-0.014300	-1	6.468480	-4.542104	-0.911554	L
H-H1-0.104800	0	6.563579	-4.427549	0.171186	L
C-C-0.597300	0	5.723786	-5.852685	-1.169336	L
O-O--0.567900	0	5.004932	-5.993404	-2.164206	L
C-CT--0.204100	0	5.661827	-3.323781	-1.413523	L
H-HC-0.079700	0	5.521720	-3.400176	-2.491614	L
H-HC-0.079700	0	6.245728	-2.438867	-1.205002	L
C-C-0.713000	-1	4.283156	-3.161290	-0.751536	L
O-O--0.593100	0	3.898483	-3.862333	0.173187	L
N-N--0.919100	0	3.460366	-2.261878	-1.247856	L
H-H-0.419600	0	2.562079	-2.199468	-0.804812	L
H-H-0.419600	0	3.664883	-1.684847	-2.055323	L
N-N--0.347900	0	5.869991	-6.784257	-0.224341	L
H-H-0.274700	0	6.540573	-6.554529	0.499128	L
C-CT--0.263700	-1	5.295709	-8.135402	-0.259052	L
H-H1-0.156000	0	5.663024	-8.665399	0.620018	L
C-C-0.734100	0	5.792873	-8.958721	-1.457762	L
O-O--0.589400	0	6.804269	-8.638578	-2.075808	L
C-CT--0.000700	0	3.755626	-8.071534	-0.158112	L
H-HC-0.032700	0	3.355090	-9.084521	-0.104791	L
H-HC-0.032700	0	3.358229	-7.607257	-1.061844	L
C-CT-0.039000	0	3.227369	-7.318168	1.068931	L
H-HC-0.028500	0	3.648357	-6.315447	1.122317	L
H-HC-0.028500	0	3.481709	-7.866354	1.977215	L
C-CT-0.048600	0	1.708923	-7.221486	0.920594	L
H-H1-0.068700	0	1.298154	-8.224398	0.789891	L
H-H1-0.068700	0	1.469087	-6.641414	0.026397	L
N-N2--0.529500	-1	1.066135	-6.609372	2.085728	L
H-H-0.345600	0	0.493086	-7.197046	2.669335	L
C-CA-0.807600	0	0.820906	-5.329790	2.259394	L
N-N2--0.862700	0	-0.120923	-4.976561	3.071485	L
H-H-0.447800	0	-0.816910	-5.641100	3.369875	L
H-H-0.447800	0	-0.331229	-3.976751	3.128018	L

N-N2--0.862700 0 1.459478 -4.378447 1.642794 L
H-H-0.447800 0 2.320946 -4.506239 1.129658 L
H-H-0.447800 0 1.071912 -3.447045 1.825324 L
N-N--0.4157 0 5.098765 -10.063076 -1.739285 L
H-H-0.2719 0 4.301621 -10.252271 -1.156020 L
C-CT--0.1490 -1 5.397934 -10.977591 -2.826461 L
H-H1-0.0976 0 6.406389 -11.378894 -2.703975 L
H-H1-0.0976 0 4.683177 -11.801886 -2.836960 L
H-H1-0.0976 0 5.349529 -10.446346 -3.779163 L
C-CT--0.3662 -1 10.086594 -6.161057 -7.689204 L
H-HC-0.1123 0 9.793768 -6.868389 -8.462619 L
H-HC-0.1123 0 10.993022 -5.642626 -8.000438 L
H-HC-0.1123 0 10.273929 -6.680116 -6.750737 L
C-C-0.5972 0 8.983766 -5.142449 -7.489358 L
O-O--0.5679 0 9.221940 -3.950178 -7.649125 L
N-N--0.347900 0 7.782921 -5.579411 -7.079510 L
H-H-0.274700 0 7.690384 -6.572247 -6.925686 L
C-CT--0.263700 -1 6.815361 -4.659599 -6.446552 L
H-H1-0.156000 0 6.653586 -3.805940 -7.105637 L
C-C-0.734100 0 7.463090 -4.144015 -5.159016 L
O-O--0.589400 0 7.816839 -4.935685 -4.290861 L
C-CT--0.000700 0 5.464560 -5.326816 -6.139636 L
H-HC-0.032700 0 4.888963 -4.624473 -5.537405 L
H-HC-0.032700 0 5.614934 -6.225255 -5.537253 L
C-CT-0.039000 0 4.666397 -5.684077 -7.406101 L
H-HC-0.028500 0 4.872330 -6.724398 -7.661104 L
H-HC-0.028500 0 4.990572 -5.063597 -8.243156 L
C-CT-0.048600 0 3.149805 -5.502001 -7.220886 L
H-H1-0.068700 0 2.839302 -5.943903 -6.272916 L
H-H1-0.068700 0 2.642457 -6.049220 -8.017604 L
N-N2--0.529500 0 2.751754 -4.079024 -7.310942 L
H-H-0.345600 0 2.727609 -3.674623 -8.230188 L
C-CA-0.807600 0 2.436042 -3.250269 -6.328810 L
N-N2--0.862700 0 2.374638 -3.590184 -5.080559 L
H-H-0.447800 0 2.555168 -4.536691 -4.762307 L
H-H-0.447800 0 2.143688 -2.852982 -4.409240 L
N-N2--0.862700 0 2.174678 -2.001647 -6.566542 L
H-H-0.447800 0 2.203060 -1.570671 -7.468959 L
H-H-0.447800 0 2.022480 -1.456579 -5.715155 L
N-N--0.415700 0 7.680343 -2.836690 -5.054283 L
H-H-0.271900 0 7.375088 -2.253862 -5.816163 L
C-CT--0.025200 -1 8.583897 -2.249181 -4.061721 L
H-H1-0.069800 0 9.454181 -1.853032 -4.585700 L
H-H1-0.069800 0 8.945802 -3.004848 -3.369179 L
C-C-0.597300 0 7.980925 -1.117950 -3.248348 L
O-O--0.567900 0 6.891759 -0.622200 -3.538000 L
N-N--0.415700 0 8.700573 -0.685294 -2.218031 L
H-H-0.271900 0 9.575496 -1.154125 -1.985948 L
C-CT--0.038900 -1 8.296893 0.455789 -1.401712 L
H-H1-0.100700 0 7.635193 1.068384 -2.010809 L
C-C-0.597300 0 9.441528 1.390772 -1.043583 L
O-O--0.567900 0 10.559802 0.976925 -0.742158 L
C-CT-0.365400 0 7.471890 0.009272 -0.181245 L
H-H1-0.004300 0 7.806800 -0.976856 0.144108 L
O-OH--0.676100 0 6.134600 -0.060402 -0.628750 L

H-HO-0.410200	0	6.169229	-0.336358	-1.557426	L
C-CT--0.243800	0	7.458259	0.944399	1.028344	L
H-HC-0.064200	0	7.084490	1.928484	0.745787	L
H-HC-0.064200	0	8.461051	1.030814	1.445758	L
H-HC-0.064200	0	6.808166	0.524967	1.795772	L
N-N--0.415700	0	9.115359	2.682546	-1.096813	L
H-H-0.271900	0	8.143534	2.893481	-1.301305	L
C-CT--0.025200	-1	9.977106	3.801288	-0.761857	L
H-H1-0.069800	0	9.802958	4.609630	-1.471931	L
H-H1-0.069800	0	11.022763	3.497750	-0.836961	L
C-C-0.597300	0	9.686187	4.322821	0.630498	L
O-O--0.567900	0	8.541270	4.561072	1.024713	L
N-N--0.347900	0	10.760957	4.530963	1.374029	L
H-H-0.274700	0	11.666158	4.356818	0.952078	L
C-CT--0.263700	-1	10.775454	5.134817	2.696926	L
H-H1-0.156000	0	9.870843	5.720597	2.851975	L
C-C-0.734100	0	11.952014	6.083996	2.785213	L
O-O--0.589400	0	12.895030	5.990088	2.004751	L
C-CT--0.000700	0	10.925596	4.031772	3.769396	L
H-HC-0.032700	0	11.220960	4.478203	4.720610	L
H-HC-0.032700	0	11.727808	3.354588	3.468278	L
C-CT-0.039000	0	9.655968	3.208120	4.008142	L
H-HC-0.028500	0	9.904158	2.354342	4.636661	L
H-HC-0.028500	0	9.266938	2.836802	3.060027	L
C-CT-0.048600	0	8.593113	4.029431	4.739997	L
H-H1-0.068700	0	8.456363	4.993893	4.251209	L
H-H1-0.068700	0	8.930145	4.214698	5.761702	L
N-N2--0.529500	-1	7.313151	3.315616	4.774597	L
H-H-0.345600	0	7.161080	2.651080	5.515034	L
C-CA-0.807600	0	6.327177	3.447660	3.910926	L
N-N2--0.862700	0	5.207794	2.827349	4.088806	L
H-H-0.447800	0	5.130852	2.065653	4.741038	L
H-H-0.447800	0	4.644107	2.782910	3.239125	L
N-N2--0.862700	0	6.408764	4.172130	2.836932	L
H-H-0.447800	0	7.298866	4.447233	2.445823	L
H-H-0.447800	0	5.577571	4.181134	2.245144	L
N-N--0.4157	0	11.881120	6.975965	3.763597	L
H-H-0.2719	0	11.070225	6.972272	4.358665	L
C-CT--0.1490	-1	12.952117	7.900727	4.033930	L
H-H1-0.0976	0	12.698627	8.540282	4.880976	L
H-H1-0.0976	0	13.869499	7.353144	4.262753	L
H-H1-0.0976	0	13.134653	8.526580	3.157152	L
C-CT--0.3662	-1	-2.074878	13.229072	-1.787811	L
H-HC-0.1123	0	-2.487080	13.759208	-0.931752	L
H-HC-0.1123	0	-0.988019	13.206657	-1.739214	L
H-HC-0.1123	0	-2.380884	13.734358	-2.703641	L
C-C-0.5972	0	-2.611247	11.809593	-1.816117	L
O-O--0.5679	0	-3.269608	11.424730	-2.769740	L
N-N--0.415700	0	-2.336691	11.039565	-0.759288	L
H-H-0.271900	0	-1.771235	11.464520	-0.043853	L
C-CT--0.023700	-1	-2.769881	9.638396	-0.570240	L
H-H1-0.088000	0	-2.312841	9.035588	-1.356789	L
C-C-0.597300	0	-4.291361	9.464955	-0.725928	L
O-O--0.567900	0	-4.761253	8.679888	-1.550308	L
C-CT-0.034200	0	-2.221362	9.157775	0.778493	L

H-HC-0.024100	0	-2.560604	9.831538	1.566158	L
H-HC-0.024100	0	-1.131878	9.196742	0.748418	L
C-CT-0.001800	0	-2.646474	7.739362	1.145448	L
H-H1-0.044000	0	-3.717343	7.746638	1.351745	L
H-H1-0.044000	0	-2.135366	7.480811	2.067020	L
S-S--0.273700	0	-2.324401	6.433535	-0.064660	L
C-CT--0.053600	0	-0.526387	6.294016	-0.028582	L
H-H1-0.068400	0	-0.213398	5.531816	-0.742666	L
H-H1-0.068400	0	-0.209453	5.999044	0.970542	L
H-H1-0.068400	0	-0.077839	7.248516	-0.301598	L
N-N--0.4157	0	-5.068760	10.212922	0.061475	L
H-H-0.2719	0	-4.607308	10.826670	0.713405	L
C-CT--0.1490	-1	-6.518921	10.248122	-0.045496	L
H-H1-0.0976	0	-6.935680	10.914338	0.711209	L
H-H1-0.0976	0	-6.804182	10.607769	-1.036441	L
H-H1-0.0976	0	-6.923819	9.244558	0.096720	L
C-CT--0.3662	-1	-4.389856	8.921291	-5.308473	L
H-HC-0.1123	0	-3.950822	9.277242	-4.377179	L
H-HC-0.1123	0	-3.627454	8.900610	-6.085222	L
H-HC-0.1123	0	-5.201547	9.577602	-5.613861	L
C-C-0.5972	0	-4.931456	7.512621	-5.114026	L
O-O--0.5679	0	-4.662312	6.639075	-5.925908	L
N-N--0.415700	0	-5.703904	7.293429	-4.046143	L
H-H-0.271900	0	-5.703767	8.046527	-3.369529	L
C-CT--0.087500	-1	-6.362070	6.007311	-3.689929	L
H-H1-0.096900	0	-6.095220	5.254570	-4.431318	L
C-C-0.597300	0	-7.878759	6.150815	-3.746620	L
O-O--0.567900	0	-8.541859	5.366974	-4.413885	L
C-CT-0.298500	0	-5.862899	5.527866	-2.317255	L
H-HC--0.029700	0	-6.003210	6.318443	-1.580152	L
C-CT--0.319200	0	-6.569359	4.272564	-1.800428	L
H-HC-0.079100	0	-6.447915	3.473141	-2.526703	L
H-HC-0.079100	0	-7.624187	4.483284	-1.632768	L
H-HC-0.079100	0	-6.127475	3.963846	-0.853199	L
C-CT--0.319200	0	-4.378146	5.193510	-2.422176	L
H-HC-0.079100	0	-4.215274	4.432330	-3.179154	L
H-HC-0.079100	0	-4.037993	4.822783	-1.465955	L
H-HC-0.079100	0	-3.801032	6.082959	-2.674399	L
N-N--0.4157	0	-8.441974	7.141052	-3.046819	L
H-H-0.2719	0	-7.817783	7.755623	-2.548187	L
C-CT--0.1490	-1	-9.877163	7.406210	-3.055148	L
H-H1-0.0976	0	-10.108409	8.264039	-2.422800	L
H-H1-0.0976	0	-10.203789	7.614124	-4.076619	L
H-H1-0.0976	0	-10.417208	6.529788	-2.692269	L
C-CT-0.1200	-1	-8.862219	2.078179	3.191458	L
H-HC-0.0800	0	-8.085041	2.837381	3.257907	L
H-HC-0.0800	0	-9.685809	2.466613	2.592898	L
H-HC-0.0800	0	-9.231789	1.847402	4.191425	L
C-CT-0.2000	0	-8.335097	0.836564	2.551955	L
H-H1-0.0800	0	-9.136187	0.096804	2.465544	L
O-OS--0.5600	0	-7.815742	1.162615	1.280678	L
C-CT-0.2000	0	-7.180661	0.278933	3.373750	L
H-H1-0.0800	0	-6.467453	1.059487	3.639613	L
O-OH--0.6800	0	-7.614719	-0.416395	4.535440	L
H-HO-0.4000	0	-8.241173	-1.094803	4.239439	L

C-CT-0.2000	0	-6.599680	-0.595488	2.279199	L
H-H1-0.0800	0	-5.561677	-0.857455	2.492625	L
O-OH--0.6800	0	-7.422542	-1.756764	2.189345	L
H-HO-0.4000	0	-6.999827	-2.362494	1.547159	L
C-CT-0.5691	0	-6.713326	0.283249	1.018867	H
H-H2-0.8000	0	-6.881884	-0.380050	0.142726	H
N-N*--0.5691	0	-5.534161	1.125809	0.810156	H
C-CM--0.0500	0	-4.844089	0.931068	-0.340450	H
H-H4-0.1500	0	-5.233037	0.123717	-0.921973	H
C-CM--0.1238	0	-3.812346	1.674278	-0.761059	H
C-C-0.6156	0	-3.387115	1.660863	-2.176601	H
O-O--0.5700	0	-2.485446	2.360422	-2.566577	H
N-N--0.8000	0	-4.127183	0.938362	-3.066644	H
H-H-0.3700	0	-3.718715	0.699041	-3.948304	H
H-H-0.3700	0	-4.794595	0.255001	-2.760749	H
C-CT-0.1164	0	-3.189668	2.672569	0.190844	H
H-HC-0.0800	0	-2.790303	3.515345	-0.352312	H
H-HC-0.0800	0	-2.334345	2.246370	0.712995	H
C-CM--0.2882	0	-4.237374	3.091622	1.193734	H
H-HA-0.1500	0	-4.123451	4.041301	1.677554	H
C-CM--0.0500	0	-5.281727	2.335723	1.480445	H
H-H4-0.1500	0	-6.007906	2.639988	2.201375	H
N-N3--0.8530	0	3.478027	1.044664	-0.681032	H
H-H-0.4500	0	3.588579	0.933235	-1.675681	H
H-H-0.4500	0	3.167491	0.655827	1.798154	H
H-H-0.4500	0	4.385880	0.869843	-0.269744	H
C-CT-0.3170	0	3.340476	2.464511	-0.393389	H
H-HP-0.0800	0	3.936626	3.053296	-1.085759	H
C-CT--0.1600	0	1.903856	2.991488	-0.456099	H
H-HC-0.0800	0	1.547298	2.906808	-1.476470	H
H-HC-0.0800	0	1.955595	4.051017	-0.234349	H
C-CT--0.1600	0	0.927472	2.317932	0.518796	H
H-HC-0.0800	0	0.149129	3.016054	0.798150	H
H-HC-0.0800	0	1.471799	2.075122	1.418084	H
C-CT--0.0990	0	0.296798	1.042703	-0.051158	H
H-HC-0.0800	0	1.060171	0.490602	-0.586331	H
H-HC-0.0800	0	-0.476398	1.313268	-0.759252	H
C-C-0.4490	0	-0.221077	0.111491	0.996049	H
H-H-0.0600	0	0.432152	-0.241691	1.771121	H
O-O--0.5700	0	2.724343	-0.203681	1.663585	H
N-N3--0.9900	0	-1.412610	-0.328161	0.953167	H
H-H-0.3600	0	2.846165	-0.186047	0.712491	H
H-H-0.3600	0	-1.945089	0.022078	0.173168	H
C-C-0.9060	0	3.991829	2.718486	0.979533	L
O-O2--0.9000	0	4.347759	1.778990	1.730146	L
O-O2--0.9000	0	4.139519	3.889104	1.386418	L
C-CT--0.205900	0	-2.170852	-1.292678	1.786135	H
H-H1-0.139900	0	-2.920198	-0.704038	2.298073	H
C-CT-0.007100	0	-2.814404	-2.237118	0.769942	H
H-HC--0.007800	0	-2.041819	-2.843472	0.320016	H
H-HC--0.007800	0	-3.289530	-1.662954	-0.022906	H
C-CT-0.067500	0	-3.847663	-3.147284	1.416016	L
H-HC--0.054800	0	-4.162335	-2.705201	2.359370	L
H-HC--0.054800	0	-3.405050	-4.124345	1.622349	L
C-C-0.818300	0	-5.097092	-3.282343	0.555346	L

O-O2--0.822000	0	-5.726128	-2.235091	0.259863	L
O-O2--0.822000	0	-5.562045	-4.413897	0.273089	L
C-C-0.742000	0	-1.280866	-2.094003	2.765333	H
O-O2--0.793000	0	-1.811579	-2.466458	3.799657	H
O-O2--0.793000	0	-0.168453	-2.355181	2.310946	H
O-OW--0.834000	0	2.817577	-5.993769	-3.691328	L
H-HW-0.417000	0	3.642451	-6.092096	-3.173126	L
H-HW-0.417000	0	2.162090	-5.762047	-3.015611	L
O-OW--0.834000	0	-2.402374	-6.664156	2.409582	L
H-HW-0.417000	0	-3.102199	-6.980772	1.812185	L
H-HW-0.417000	0	-2.887867	-6.030743	2.949941	L
O-OW--0.834000	0	4.618781	0.020546	3.729286	L
H-HW-0.417000	0	3.900168	-0.553171	3.433882	L
H-HW-0.417000	0	4.729055	0.632629	2.972232	L
O-OW--0.834000	0	6.448545	-1.676122	4.745445	L
H-HW-0.417000	0	6.012773	-2.546137	4.786139	L
H-HW-0.417000	0	5.729585	-1.087476	4.447003	L
O-OW--0.834000	0	-4.924862	-6.839305	1.153694	L
H-HW-0.417000	0	-5.173266	-5.935160	0.861470	L
H-HW-0.417000	0	-5.773536	-7.285931	1.208283	L
O-OW--0.834000	0	-8.972589	-1.229584	-0.166608	L
H-HW-0.417000	0	-9.869687	-1.249980	0.171663	L
H-HW-0.417000	0	-8.709267	-2.174718	-0.132524	L

TS6

C-CT--0.3662	-1	-12.876004	1.077321	1.861295	L
H-HC-0.1123	0	-12.619305	2.113844	1.649032	L
H-HC-0.1123	0	-13.922872	0.908704	1.615974	L
H-HC-0.1123	0	-12.237924	0.418514	1.273477	L
C-C-0.5972	0	-12.641438	0.803803	3.333904	L
O-O--0.5679	0	-11.968825	1.589621	3.992632	L
N-N--0.415700	0	-13.176983	-0.313360	3.836819	L
H-H-0.271900	0	-13.687632	-0.894640	3.192440	L
C-CT--0.025200	-1	-12.921174	-0.832016	5.192832	L
H-H1-0.069800	0	-13.645026	-1.613885	5.421594	L
H-H1-0.069800	0	-13.032856	-0.023232	5.915649	L
C-C-0.597300	0	-11.482413	-1.420522	5.329067	L
O-O--0.567900	0	-10.598792	-1.173249	4.502299	L
N-N--0.415700	0	-11.224521	-2.234781	6.363998	L
H-H-0.271900	0	-11.980998	-2.435038	6.999073	L
C-CT--0.025200	-1	-10.016229	-3.086721	6.453230	L
H-H1-0.069800	0	-9.917236	-3.478356	7.465225	L
H-H1-0.069800	0	-9.128260	-2.501710	6.209499	L
C-C-0.597300	0	-10.153958	-4.257244	5.491969	L
O-O--0.567900	0	-11.186988	-4.920903	5.459494	L
N-N--0.4157	0	-9.105846	-4.504055	4.718804	L
H-H-0.2719	0	-8.295850	-3.917074	4.836089	L
C-CT--0.0014	-1	-9.023142	-5.477112	3.630420	L
H-H1-0.0876	0	-9.874056	-5.355154	2.959202	L
C-C-0.5973	0	-8.982352	-6.957148	4.071788	L
O-O--0.5679	0	-8.323416	-7.789437	3.450167	L
C-CT--0.0152	0	-7.733149	-5.134348	2.863862	L
H-HC-0.0295	0	-7.899629	-4.190363	2.357112	L
H-HC-0.0295	0	-7.585649	-5.855279	2.057998	L
C-CA--0.0011	0	-6.436126	-5.032483	3.677106	L

C-CA--0.1906	0	-5.576465	-6.142779	3.794337	L
H-HA-0.1699	0	-5.906949	-7.113211	3.441185	L
C-CA--0.1906	0	-6.024027	-3.798687	4.223807	L
H-HA-0.1699	0	-6.689046	-2.955816	4.252820	L
C-CA--0.2341	0	-4.264216	-5.976769	4.290300	L
H-HA-0.1656	0	-3.574846	-6.806225	4.334974	L
C-CA--0.2341	0	-4.703814	-3.615632	4.671568	L
H-HA-0.1656	0	-4.351658	-2.643122	4.977431	L
C-C-0.3326	0	-3.799839	-4.692699	4.646352	L
O-OH--0.5579	0	-2.484802	-4.478269	4.905550	L
H-HO-0.3992	0	-2.193637	-3.642505	4.477795	L
N-N--0.4157	0	-9.674770	-7.307530	5.153772	L
H-H-0.2719	0	-10.285588	-6.602590	5.548949	L
C-CT--0.1490	-1	-9.607866	-8.632321	5.740054	L
H-H1-0.0976	0	-8.580092	-8.856573	6.031614	L
H-H1-0.0976	0	-10.253631	-8.688026	6.617188	L
H-H1-0.0976	0	-9.932415	-9.374730	5.007905	L
C-CT--0.3662	-1	-11.991065	-1.501255	-3.506327	L
H-HC-0.1123	0	-12.523779	-0.612902	-3.841132	L
H-HC-0.1123	0	-12.000052	-1.567099	-2.419882	L
H-HC-0.1123	0	-12.470427	-2.385912	-3.924288	L
C-C-0.5972	0	-10.556761	-1.449378	-4.001522	L
O-O--0.5679	0	-10.090242	-2.313056	-4.732444	L
N-N--0.415700	0	-9.837125	-0.427080	-3.560207	L
H-H-0.271900	0	-10.328471	0.240371	-2.984022	L
C-CT--0.038900	-1	-8.390971	-0.159183	-3.722435	L
H-H1-0.100700	0	-8.084921	-0.246980	-4.762892	L
C-C-0.597300	0	-7.592279	-1.133128	-2.847780	L
O-O--0.567900	0	-6.943677	-0.755628	-1.870022	L
C-CT-0.365400	0	-8.183480	1.284630	-3.220546	L
H-H1-0.004300	0	-7.123967	1.525973	-3.255978	L
O-OH--0.676100	0	-8.666423	1.428796	-1.896715	L
H-HO-0.410200	0	-8.441345	2.324377	-1.621614	L
C-CT--0.243800	0	-8.991395	2.337372	-3.974618	L
H-HC-0.064200	0	-10.051848	2.092210	-4.006511	L
H-HC-0.064200	0	-8.627445	2.441058	-4.987578	L
H-HC-0.064200	0	-8.889746	3.296249	-3.477454	L
N-N--0.415700	0	-7.708392	-2.423719	-3.123652	L
H-H-0.271900	0	-8.302579	-2.702998	-3.899938	L
C-CT--0.024900	-1	-7.192351	-3.486358	-2.262633	L
H-H1-0.084300	0	-6.334402	-3.125180	-1.715328	L
C-C-0.597300	0	-6.684237	-4.660178	-3.089392	L
O-O--0.567900	0	-7.067331	-4.838527	-4.243346	L
C-CT-0.211700	0	-8.284917	-4.020127	-1.332211	L
H-H1-0.035200	0	-7.979761	-4.977562	-0.905042	L
H-H1-0.035200	0	-9.221715	-4.150431	-1.876582	L
O-OH--0.654600	0	-8.439197	-3.095549	-0.290755	L
H-HO-0.427500	0	-7.528976	-3.023565	0.072886	L
N-N--0.4157	0	-5.806250	-5.463043	-2.498035	L
H-H-0.2719	0	-5.579028	-5.274336	-1.522709	L
C-CT--0.0014	0	-5.348111	-6.711855	-3.093268	L
H-H1-0.0876	0	-4.958270	-6.502165	-4.089534	L
C-C-0.5973	0	-6.470908	-7.743877	-3.230763	L
O-O--0.5679	0	-7.438080	-7.753756	-2.471375	L
C-CT--0.0152	0	-4.232140	-7.301088	-2.236394	L

H-HC-0.0295	0	-4.055144	-8.333710	-2.540226	L
H-HC-0.0295	0	-4.554585	-7.329370	-1.194835	L
C-CA--0.0011	-1	-2.920566	-6.565731	-2.349708	L
C-CA--0.1906	0	-2.035593	-6.854302	-3.411336	L
H-HA-0.1699	0	-2.336436	-7.542021	-4.188732	L
C-CA--0.1906	0	-2.495116	-5.687748	-1.333724	L
H-HA-0.1699	0	-3.146443	-5.452864	-0.513167	L
C-CA--0.2341	0	-0.745189	-6.281588	-3.443557	L
H-HA-0.1656	0	-0.062021	-6.506278	-4.248176	L
C-CA--0.2341	0	-1.204120	-5.130676	-1.344087	L
H-HA-0.1656	0	-0.891153	-4.483150	-0.536464	L
C-C-0.3226	0	-0.325579	-5.425646	-2.402265	L
O-OH--0.5579	0	0.918441	-4.878215	-2.408290	L
H-HO-0.3992	0	0.939803	-4.159398	-1.767631	L
N-N--0.4157	0	-6.282241	-8.664095	-4.179841	L
H-H-0.2719	0	-5.471115	-8.564314	-4.766762	L
C-CT--0.1490	-1	-7.140887	-9.818566	-4.362163	L
H-H1-0.0976	0	-8.156425	-9.486253	-4.588707	L
H-H1-0.0976	0	-6.775333	-10.440410	-5.180116	L
H-H1-0.0976	0	-7.168191	-10.407329	-3.442838	L
C-CT--0.3662	-1	-6.310887	-3.609085	-6.943504	L
H-HC-0.1123	0	-5.850088	-4.377064	-7.561382	L
H-HC-0.1123	0	-7.352792	-3.870237	-6.762261	L
H-HC-0.1123	0	-5.779942	-3.532751	-6.000098	L
C-C-0.5972	0	-6.224143	-2.272252	-7.663674	L
O-O--0.5679	0	-5.334787	-2.113173	-8.488169	L
N-N--0.415700	0	-7.118162	-1.319506	-7.357631	L
H-H-0.271900	0	-7.837257	-1.583575	-6.701501	L
C-CT--0.025200	-1	-7.254371	-0.016025	-8.056558	L
H-H1-0.069800	0	-8.204930	-0.010267	-8.590289	L
H-H1-0.069800	0	-6.469652	0.093817	-8.804614	L
C-C-0.597300	0	-7.222164	1.217009	-7.160231	L
O-O--0.567900	0	-8.083989	2.074398	-7.301195	L
N-N--0.415700	0	-6.234332	1.307676	-6.264455	L
H-H-0.271900	0	-5.559763	0.549911	-6.257045	L
C-CT--0.025200	-1	-5.798373	2.554238	-5.624568	L
H-H1-0.069800	0	-5.608797	2.378539	-4.567960	L
H-H1-0.069800	0	-6.560315	3.327681	-5.731682	L
C-C-0.597300	0	-4.511763	3.059338	-6.288761	L
O-O--0.567900	0	-4.448200	3.175040	-7.509415	L
N-N--0.415700	0	-3.468973	3.297450	-5.490753	L
H-H-0.271900	0	-3.589199	3.154682	-4.497365	L
C-CT--0.051800	-1	-2.128041	3.678743	-5.948806	L
H-H1-0.092200	0	-2.202318	4.113719	-6.947036	L
C-C-0.597300	0	-1.209161	2.443122	-6.038199	L
O-O--0.567900	0	-0.505820	2.226296	-7.021801	L
C-CT--0.110200	0	-1.592381	4.748069	-4.976212	L
H-HC-0.045700	0	-1.579901	4.330580	-3.968943	L
H-HC-0.045700	0	-2.285468	5.590714	-4.976085	L
C-CT-0.353100	0	-0.182931	5.273573	-5.295923	L
H-HC--0.036100	0	0.526743	4.446299	-5.287209	L
C-CT--0.412100	0	-0.111534	5.976850	-6.651134	L
H-HC-0.100000	0	-0.857435	6.771159	-6.699805	L
H-HC-0.100000	0	-0.301449	5.260330	-7.450280	L
H-HC-0.100000	0	0.881090	6.400413	-6.799543	L

C-CT--0.412100	0	0.233410	6.272661	-4.214144	L
H-HC-0.100000	0	-0.466189	7.109232	-4.193501	L
H-HC-0.100000	0	1.234909	6.647586	-4.423681	L
H-HC-0.100000	0	0.235673	5.779615	-3.243300	L
N-N--0.516300	0	-1.183260	1.643513	-4.973213	L
H-H-0.293600	0	-1.777406	1.925928	-4.200431	L
C-CT-0.038100	-1	-0.280856	0.516022	-4.744079	L
H-H1-0.088000	0	-0.040899	0.016658	-5.680695	L
C-C-0.536600	0	-0.924161	-0.486061	-3.748369	L
O-O--0.581900	0	-0.703898	-0.383064	-2.542283	L
C-CT--0.030300	0	1.025199	1.115230	-4.146288	L
H-HC--0.012200	0	0.764463	1.751466	-3.300862	L
H-HC--0.012200	0	1.488201	1.754006	-4.900383	L
C-C-0.799400	-1	2.079339	0.099565	-3.677205	L
O-O2--0.801400	0	1.969452	-1.089765	-4.061032	L
O-O2--0.801400	0	3.112656	0.500285	-3.100251	L
N-N--0.254800	0	-1.732057	-1.458626	-4.205374	L
C-CT--0.026600	-1	-2.028469	-1.768726	-5.598544	L
H-H1-0.064100	0	-1.102857	-1.829143	-6.169973	L
C-C-0.589600	0	-2.971384	-0.761629	-6.281242	L
O-O--0.574800	0	-3.918481	-0.273006	-5.664179	L
C-CT--0.007000	0	-2.663176	-3.164384	-5.559022	L
H-HC-0.025300	0	-1.877419	-3.921436	-5.581513	L
H-HC-0.025300	0	-3.363067	-3.325895	-6.379449	L
C-CT-0.018900	0	-3.356488	-3.199819	-4.198457	L
H-HC-0.021300	0	-3.499315	-4.218156	-3.841699	L
H-HC-0.021300	0	-4.308550	-2.669425	-4.246325	L
C-CT-0.019200	0	-2.377561	-2.422213	-3.325165	L
H-H1-0.039100	0	-2.899706	-1.932517	-2.503576	L
H-H1-0.039100	0	-1.618757	-3.092644	-2.929498	L
N-N--0.4157	0	-2.714901	-0.483979	-7.564191	L
H-H-0.2719	0	-1.923158	-0.936388	-7.987786	L
C-CT--0.1490	-1	-3.506305	0.383354	-8.436870	L
H-H1-0.0976	0	-4.469942	0.604441	-7.991040	L
H-H1-0.0976	0	-3.678447	-0.108115	-9.395507	L
H-H1-0.0976	0	-2.978066	1.324576	-8.604674	L
C-CT--0.3662	-1	8.774406	1.798789	-5.922777	L
H-HC-0.1123	0	9.268869	2.031505	-6.865535	L
H-HC-0.1123	0	9.526086	1.594044	-5.163270	L
H-HC-0.1123	0	8.124514	0.936626	-6.058254	L
C-C-0.5972	0	7.950574	2.994433	-5.498212	L
O-O--0.5679	0	8.085415	4.063057	-6.075664	L
N-N--0.415700	0	7.097009	2.822605	-4.488815	L
H-H-0.271900	0	7.033815	1.900121	-4.081568	L
C-CT-0.021300	-1	6.102153	3.822645	-4.075561	L
H-H1-0.112400	0	6.293155	4.768345	-4.587076	L
C-C-0.597300	0	6.127142	4.084653	-2.566292	L
O-O--0.567900	0	6.358831	3.175031	-1.768117	L
C-CT--0.123100	0	4.730855	3.295128	-4.530864	L
H-H1-0.111200	0	4.547916	2.317211	-4.079730	L
H-H1-0.111200	0	4.734269	3.181350	-5.616587	L
S-SH--0.311900	0	3.391928	4.426809	-4.057524	L
H-HS-0.193300	0	2.396409	3.758130	-4.657705	L
N-N--0.415700	0	5.865486	5.329409	-2.167967	L
H-H-0.271900	0	5.638417	6.015703	-2.874848	L

C-CT--0.025200	-1	5.835399	5.757988	-0.774944	L
H-H1-0.069800	0	6.793705	6.214658	-0.527911	L
H-H1-0.069800	0	5.698720	4.902311	-0.116398	L
C-C-0.597300	0	4.744247	6.786505	-0.507290	L
O-O--0.567900	0	4.837824	7.923237	-0.961236	L
N-N--0.415700	0	3.737014	6.416114	0.281685	L
H-H-0.271900	0	3.783135	5.481181	0.683089	L
C-CT--0.025200	-1	2.700176	7.318837	0.779531	L
H-H1-0.069800	0	1.851954	6.725129	1.106188	L
H-H1-0.069800	0	2.382102	8.007517	-0.004054	L
C-C-0.597300	0	3.198110	8.115193	1.983425	L
O-O--0.567900	0	2.630074	8.006008	3.067813	L
N-N--0.4157	0	4.278937	8.877526	1.785322	L
H-H-0.2719	0	4.632497	8.889026	0.833598	L
C-CT--0.1490	-1	5.024962	9.574114	2.825066	L
H-H1-0.0976	0	5.365026	8.860658	3.577639	L
H-H1-0.0976	0	5.887186	10.082638	2.390980	L
H-H1-0.0976	0	4.380269	10.311146	3.307974	L
C-CT--0.3662	-1	-1.509382	9.519490	5.121266	L
H-HC-0.1123	0	-1.713909	10.474936	4.640795	L
H-HC-0.1123	0	-0.566382	9.119524	4.752601	L
H-HC-0.1123	0	-1.460146	9.661835	6.199370	L
C-C-0.5972	0	-2.641751	8.543299	4.791009	L
O-O--0.5679	0	-3.515574	8.882123	4.003168	L
N-N--0.4157	0	-2.623580	7.330043	5.358257	L
H-H-0.2719	0	-1.885860	7.130767	6.019961	L
C-CT--0.0275	-1	-3.610237	6.231862	5.185600	L
H-H1-0.1123	0	-4.608960	6.661532	5.101664	L
C-C-0.5973	0	-3.586211	5.281271	6.411788	L
O-O--0.5679	0	-2.671117	5.352603	7.230799	L
C-CT--0.0050	0	-3.298012	5.430855	3.915166	L
H-HC-0.0339	0	-3.461949	6.068357	3.054014	L
H-HC-0.0339	0	-4.032109	4.629885	3.834520	L
C-C*--0.1415	0	-1.925658	4.820722	3.824479	L
C-CW--0.1638	0	-0.764684	5.492385	3.632963	L
H-H4-0.2062	0	-0.677609	6.565996	3.524115	L
C-CB-0.1243	0	-1.554069	3.412250	3.930121	L
N-NA--0.3418	0	0.294767	4.605897	3.626446	L
H-H-0.3412	0	1.262703	4.889497	3.539331	L
C-CN-0.1380	0	-0.136834	3.309289	3.800902	L
C-CA--0.238	0	-2.270200	2.210678	4.126882	L
H-HA-0.1700	0	-3.342847	2.244067	4.224162	L
C-CA--0.2601	0	0.534373	2.081247	3.869111	L
H-HA-0.1572	0	1.608544	2.042626	3.785547	L
C-CA--0.1972	0	-1.608991	0.969432	4.160371	L
H-HA-0.1447	0	-2.166869	0.047580	4.267484	L
C-CA--0.1134	0	-0.213278	0.902044	4.025927	L
H-HA-0.1417	0	0.270064	-0.066580	4.032465	L
N-N--0.4157	0	-4.572162	4.374363	6.535959	L
H-H-0.2719	0	-5.291940	4.395747	5.832201	L
C-CT--0.1490	-1	-4.666985	3.361157	7.601852	L
H-H1-0.0976	0	-3.841870	2.653261	7.508028	L
H-H1-0.0976	0	-4.599423	3.847912	8.577400	L
H-H1-0.0976	0	-5.614889	2.825135	7.534024	L
C-CT--0.3662	-1	-2.825821	-1.366548	7.279263	L

H-HC-0.1123	0	-3.053315	-2.423779	7.141232	L
H-HC-0.1123	0	-3.177348	-1.045274	8.257164	L
H-HC-0.1123	0	-3.308195	-0.792942	6.490360	L
C-C-0.5972	0	-1.333672	-1.177862	7.181867	L
O-O--0.5679	0	-0.619003	-2.132496	6.934189	L
N-N--0.415700	0	-0.836204	0.028953	7.446159	L
H-H-0.271900	0	-1.494216	0.782480	7.566319	L
C-CT--0.087500	-1	0.607230	0.354665	7.407423	L
H-H1-0.096900	0	0.944705	0.246354	6.377384	L
C-C-0.597300	0	1.447605	-0.600283	8.265184	L
O-O--0.567900	0	2.446923	-1.130134	7.794114	L
C-CT-0.298500	0	0.885874	1.808486	7.863529	L
H-HC--0.029700	0	0.952277	1.832183	8.951853	L
C-CT--0.319200	0	2.225761	2.293244	7.296800	L
H-HC-0.079100	0	2.182088	2.322644	6.208771	L
H-HC-0.079100	0	3.027937	1.621959	7.604902	L
H-HC-0.079100	0	2.446117	3.292518	7.672899	L
C-CT--0.319200	0	-0.204426	2.814086	7.469304	L
H-HC-0.079100	0	-0.419070	2.736965	6.408292	L
H-HC-0.079100	0	0.121646	3.830956	7.690362	L
H-HC-0.079100	0	-1.117113	2.631744	8.034852	L
N-N--0.415700	0	1.027711	-0.844760	9.514811	L
H-H-0.271900	0	0.199118	-0.359314	9.817338	L
C-CT--0.025200	-1	1.633748	-1.831561	10.423489	L
H-H1-0.069800	0	1.084575	-1.852911	11.364107	L
H-H1-0.069800	0	2.663900	-1.540897	10.632764	L
C-C-0.597300	0	1.654354	-3.266254	9.842754	L
O-O--0.567900	0	2.517598	-4.049358	10.219118	L
N-N--0.415700	0	0.719837	-3.615869	8.943877	L
H-H-0.271900	0	0.091415	-2.899060	8.611443	L
C-CT-0.033700	-1	0.631600	-4.902852	8.238316	L
H-H1-0.082300	0	1.041934	-5.675111	8.891872	L
C-C-0.597300	0	1.470596	-4.951415	6.936917	L
O-O--0.567900	0	2.122184	-5.956148	6.663302	L
C-CT--0.182500	0	-0.841367	-5.253509	7.991905	L
H-HC-0.060300	0	-1.383644	-5.264390	8.937522	L
H-HC-0.060300	0	-0.906014	-6.242538	7.536682	L
H-HC-0.060300	0	-1.301319	-4.532302	7.319691	L
N-N--0.415700	0	1.492281	-3.871752	6.145696	L
H-H-0.271900	0	0.850752	-3.123212	6.380950	L
C-CT-0.033700	-1	2.440194	-3.647809	5.041913	L
H-H1-0.082300	0	2.353064	-4.472611	4.336261	L
C-C-0.597300	0	3.910383	-3.600845	5.515127	L
O-O--0.567900	0	4.828910	-3.790881	4.715055	L
C-CT--0.182500	0	2.046907	-2.345305	4.342232	L
H-HC-0.060300	0	2.224815	-1.498162	5.004840	L
H-HC-0.060300	0	0.989532	-2.376077	4.085237	L
H-HC-0.060300	0	2.631862	-2.231366	3.429893	L
N-N--0.4157	0	4.139942	-3.305420	6.800100	L
H-H-0.2719	0	3.328403	-3.032917	7.339735	L
C-CT--0.1490	-1	5.418744	-3.381614	7.491421	L
H-H1-0.0976	0	5.999154	-4.223716	7.109248	L
H-H1-0.0976	0	5.248382	-3.525743	8.559936	L
H-H1-0.0976	0	5.980179	-2.459264	7.336163	L
C-CT--0.3662	-1	14.095178	0.896650	-0.666608	L

H-HC-0.1123	0	13.457458	1.422031	0.041368	L
H-HC-0.1123	0	15.103376	1.306764	-0.630319	L
H-HC-0.1123	0	13.695279	0.995113	-1.673654	L
C-C-0.5972	0	14.149585	-0.576769	-0.297052	L
O-O--0.5679	0	15.230011	-1.137273	-0.174921	L
N-N--0.4157	0	12.987785	-1.210476	-0.139864	L
H-H-0.2719	0	12.146637	-0.661313	-0.271570	L
C-CT--0.0014	-1	12.808824	-2.662227	0.073253	L
H-H1-0.0876	0	13.693420	-3.184518	-0.293972	L
C-C-0.5973	0	11.591377	-3.163789	-0.755756	L
O-O--0.5679	0	10.839305	-2.368908	-1.328844	L
C-CT--0.0152	0	12.649980	-2.977299	1.570639	L
H-HC-0.0295	0	13.597365	-2.756706	2.064994	L
H-HC-0.0295	0	12.472736	-4.045996	1.695984	L
C-CA--0.0011	-1	11.546900	-2.209574	2.278562	L
C-CA--0.1906	0	11.855516	-0.976067	2.883022	L
H-HA-0.1699	0	12.868033	-0.597655	2.843091	L
C-CA--0.1906	0	10.231008	-2.708227	2.332422	L
H-HA-0.1699	0	9.987644	-3.660354	1.881728	L
C-CA--0.2341	0	10.856268	-0.242263	3.545043	L
H-HA-0.1656	0	11.099405	0.687931	4.026982	L
C-CA--0.2341	0	9.226661	-1.973348	2.992405	L
H-HA-0.1656	0	8.221169	-2.363078	3.041459	L
C-C-0.3226	0	9.538229	-0.739260	3.605662	L
O-OH--0.557	0	8.581581	-0.032303	4.263313	L
H-HO-0.3992	0	7.817288	-0.620688	4.439067	L
N-N--0.415700	0	11.377457	-4.483381	-0.851484	L
H-H-0.271900	0	11.992232	-5.094889	-0.337232	L
C-CT--0.025200	-1	10.253569	-5.092712	-1.592073	L
H-H1-0.069800	0	10.465244	-6.149219	-1.755591	L
H-H1-0.069800	0	10.150556	-4.615640	-2.565965	L
C-C-0.597300	0	8.928442	-4.995345	-0.822987	L
O-O--0.567900	0	8.862450	-5.367442	0.346970	L
N-N--0.415700	0	7.859167	-4.519407	-1.467061	L
H-H-0.271900	0	7.942484	-4.410343	-2.470511	L
C-CT-0.014300	-1	6.518423	-4.488780	-0.884991	L
H-H1-0.104800	0	6.607724	-4.375926	0.198432	L
C-C-0.597300	0	5.783219	-5.803601	-1.148872	L
O-O--0.567900	0	5.071537	-5.948262	-2.148244	L
C-CT--0.204100	0	5.705692	-3.272287	-1.388677	L
H-HC-0.079700	0	5.570559	-3.347513	-2.467407	L
H-HC-0.079700	0	6.280385	-2.381287	-1.175090	L
C-C-0.713000	-1	4.323288	-3.122970	-0.730611	L
O-O--0.593100	0	3.961919	-3.812956	0.210484	L
N-N--0.919100	0	3.479172	-2.252694	-1.242984	L
H-H-0.419600	0	2.597248	-2.165628	-0.769423	L
H-H-0.419600	0	3.676990	-1.673108	-2.050053	L
N-N--0.347900	0	5.929680	-6.735230	-0.204076	L
H-H-0.274700	0	6.594155	-6.501224	0.523630	L
C-CT--0.263700	-1	5.367249	-8.091215	-0.244533	L
H-H1-0.156000	0	5.740082	-8.620833	0.632448	L
C-C-0.734100	0	5.872249	-8.906741	-1.445342	L
O-O--0.589400	0	6.881033	-8.576084	-2.062003	L
C-CT--0.000700	0	3.828251	-8.042764	-0.141240	L
H-HC-0.032700	0	3.438103	-9.059960	-0.090560	L

H-HC-0.032700 0 3.425916 -7.578377 -1.042979 L
C-CT-0.039000 0 3.297034 -7.298087 1.089614 L
H-HC-0.028500 0 3.694455 -6.285383 1.132721 L
H-HC-0.028500 0 3.571115 -7.835795 1.998377 L
C-CT-0.048600 0 1.776657 -7.243097 0.948867 L
H-H1-0.068700 0 1.389836 -8.260055 0.861360 L
H-H1-0.068700 0 1.517781 -6.706277 0.033085 L
N-N2--0.529500 -1 1.118475 -6.598482 2.086931 L
H-H-0.345600 0 0.420955 -7.144747 2.577348 L
C-CA-0.807600 0 0.857533 -5.314760 2.194433 L
N-N2--0.862700 0 -0.159422 -4.930841 2.899087 L
H-H-0.447800 0 -0.809716 -5.599211 3.281871 L
H-H-0.447800 0 -0.408140 -3.938231 2.886515 L
N-N2--0.862700 0 1.546137 -4.391725 1.593567 L
H-H-0.447800 0 2.434966 -4.536324 1.135414 L
H-H-0.447800 0 1.122667 -3.461925 1.655742 L
N-N--0.4157 0 5.188771 -10.017024 -1.729576 L
H-H-0.2719 0 4.393613 -10.215357 -1.146705 L
C-CT--0.1490 -1 5.498487 -10.927244 -2.817427 L
H-H1-0.0976 0 6.511348 -11.317336 -2.694978 L
H-H1-0.0976 0 4.793057 -11.759510 -2.828960 L
H-H1-0.0976 0 5.444565 -10.395724 -3.769701 L
C-CT--0.3662 -1 10.173577 -6.069195 -7.651856 L
H-HC-0.1123 0 9.884784 -6.767111 -8.435288 L
H-HC-0.1123 0 11.080489 -5.545697 -7.953056 L
H-HC-0.1123 0 10.358189 -6.599795 -6.719319 L
C-C-0.5972 0 9.069345 -5.055116 -7.443643 L
O-O--0.5679 0 9.304167 -3.860533 -7.590420 L
N-N--0.347900 0 7.869132 -5.501248 -7.044212 L
H-H-0.274700 0 7.779923 -6.495853 -6.900117 L
C-CT--0.263700 -1 6.887554 -4.592297 -6.418830 L
H-H1-0.156000 0 6.731426 -3.736250 -7.076170 L
C-C-0.734100 0 7.513601 -4.074976 -5.121256 L
O-O--0.589400 0 7.858209 -4.865881 -4.249348 L
C-CT--0.000700 0 5.536435 -5.271845 -6.149706 L
H-HC-0.032700 0 4.941844 -4.577991 -5.557938 L
H-HC-0.032700 0 5.678878 -6.174394 -5.551653 L
C-CT-0.039000 0 4.779379 -5.623150 -7.443926 L
H-HC-0.028500 0 4.989363 -6.664142 -7.692808 L
H-HC-0.028500 0 5.137307 -5.004923 -8.268816 L
C-CT-0.048600 0 3.257436 -5.434665 -7.321748 L
H-H1-0.068700 0 2.901516 -5.892572 -6.398370 L
H-H1-0.068700 0 2.783707 -5.965009 -8.149937 L
N-N2--0.529500 0 2.867766 -4.008916 -7.403490 L
H-H-0.345600 0 2.849012 -3.598679 -8.320303 L
C-CA-0.807600 0 2.545112 -3.188313 -6.416818 L
N-N2--0.862700 0 2.482331 -3.538060 -5.171001 L
H-H-0.447800 0 2.683924 -4.484281 -4.863757 L
H-H-0.447800 0 2.250529 -2.805988 -4.495239 L
N-N2--0.862700 0 2.279087 -1.939034 -6.647226 L
H-H-0.447800 0 2.308226 -1.504440 -7.547945 L
H-H-0.447800 0 2.120580 -1.397670 -5.794381 L
N-N--0.415700 0 7.729314 -2.766708 -5.015411 L
H-H-0.271900 0 7.433395 -2.185848 -5.782503 L
C-CT--0.025200 -1 8.630627 -2.175116 -4.022126 L

H-H1-0.069800	0	9.510466	-1.796165	-4.542869	L
H-H1-0.069800	0	8.976497	-2.925865	-3.316230	L
C-C-0.597300	0	8.033161	-1.025883	-3.230416	L
O-O--0.567900	0	6.973936	-0.493888	-3.565572	L
N-N--0.415700	0	8.725273	-0.618882	-2.170182	L
H-H-0.271900	0	9.582931	-1.106316	-1.913524	L
C-CT--0.038900	-1	8.315175	0.522266	-1.357637	L
H-H1-0.100700	0	7.672887	1.139662	-1.982033	L
C-C-0.597300	0	9.451320	1.458536	-0.974806	L
O-O--0.567900	0	10.563589	1.048695	-0.647284	L
C-CT-0.365400	0	7.441650	0.083883	-0.164587	L
H-H1-0.004300	0	7.757072	-0.905763	0.169267	L
O-OH--0.676100	0	6.107984	0.028200	-0.636490	L
H-HO-0.410200	0	6.153138	-0.187317	-1.579716	L
C-CT--0.243800	0	7.408675	1.016108	1.047094	L
H-HC-0.064200	0	7.049202	2.003501	0.758004	L
H-HC-0.064200	0	8.402591	1.093912	1.486819	L
H-HC-0.064200	0	6.737697	0.600712	1.798928	L
N-N--0.415700	0	9.123547	2.749438	-1.044133	L
H-H-0.271900	0	8.154634	2.953677	-1.268153	L
C-CT--0.025200	-1	9.970426	3.877588	-0.704291	L
H-H1-0.069800	0	9.787341	4.686777	-1.411125	L
H-H1-0.069800	0	11.019606	3.586162	-0.778194	L
C-C-0.597300	0	9.668929	4.387744	0.690858	L
O-O--0.567900	0	8.519646	4.607622	1.085199	L
N-N--0.347900	0	10.740129	4.607022	1.436627	L
H-H-0.274700	0	11.647886	4.445273	1.015190	L
C-CT--0.263700	-1	10.746393	5.209148	2.760338	L
H-H1-0.156000	0	9.838406	5.790416	2.911145	L
C-C-0.734100	0	11.916141	6.166196	2.854180	L
O-O--0.589400	0	12.860939	6.082412	2.074764	L
C-CT--0.000700	0	10.899150	4.106839	3.832600	L
H-HC-0.032700	0	11.185458	4.555262	4.785626	L
H-HC-0.032700	0	11.707856	3.435436	3.535982	L
C-CT-0.039000	0	9.633991	3.275056	4.063987	L
H-HC-0.028500	0	9.881034	2.433814	4.710067	L
H-HC-0.028500	0	9.262917	2.885283	3.115977	L
C-CT-0.048600	0	8.553171	4.099580	4.764903	L
H-H1-0.068700	0	8.397587	5.043670	4.243478	L
H-H1-0.068700	0	8.882979	4.325501	5.780748	L
N-N2--0.529500	-1	7.288331	3.362433	4.820729	L
H-H-0.345600	0	7.151971	2.714274	5.578385	L
C-CA-0.807600	0	6.295588	3.452815	3.957548	L
N-N2--0.862700	0	5.187872	2.820208	4.162700	L
H-H-0.447800	0	5.127224	2.075844	4.835827	L
H-H-0.447800	0	4.613743	2.745311	3.323472	L
N-N2--0.862700	0	6.354229	4.147260	2.861036	L
H-H-0.447800	0	7.236132	4.442933	2.464563	L
H-H-0.447800	0	5.516149	4.141604	2.278467	L
N-N--0.4157	0	11.836624	7.054086	3.835671	L
H-H-0.2719	0	11.024786	7.041512	4.429303	L
C-CT--0.1490	-1	12.899259	7.986751	4.111555	L
H-H1-0.0976	0	12.638581	8.621368	4.960133	L
H-H1-0.0976	0	13.820612	7.445993	4.340658	L
H-H1-0.0976	0	13.078641	8.617006	3.237282	L

C-CT--0.3662	-1	-2.140427	13.226594	-1.757555	L
H-HC-0.1123	0	-2.564799	13.752748	-0.904976	L
H-HC-0.1123	0	-1.053930	13.212672	-1.698666	L
H-HC-0.1123	0	-2.441747	13.730384	-2.675764	L
C-C-0.5972	0	-2.665615	11.802882	-1.792217	L
O-O--0.5679	0	-3.312556	11.412890	-2.751523	L
N-N--0.415700	0	-2.393844	11.033949	-0.734187	L
H-H-0.271900	0	-1.838323	11.463149	-0.013376	L
C-CT--0.023700	-1	-2.816058	9.628789	-0.550168	L
H-H1-0.088000	0	-2.351530	9.030679	-1.335839	L
C-C-0.597300	0	-4.335411	9.441082	-0.707129	L
O-O--0.567900	0	-4.796416	8.638578	-1.519467	L
C-CT-0.034200	0	-2.268339	9.152307	0.801456	L
H-HC-0.024100	0	-2.624435	9.818355	1.588480	L
H-HC-0.024100	0	-1.179768	9.213753	0.779448	L
C-CT-0.001800	0	-2.666109	7.725201	1.170089	L
H-H1-0.044000	0	-3.736547	7.715252	1.378780	L
H-H1-0.044000	0	-2.146924	7.477459	2.092440	L
S-S--0.273700	0	-2.326613	6.416572	-0.035246	L
C-CT--0.053600	0	-0.524750	6.389292	-0.069549	L
H-H1-0.068400	0	-0.191480	5.648273	-0.796129	L
H-H1-0.068400	0	-0.159723	6.116029	0.918841	L
H-H1-0.068400	0	-0.144326	7.368985	-0.357101	L
N-N--0.4157	0	-5.120656	10.194352	0.067255	L
H-H-0.2719	0	-4.665709	10.823751	0.708694	L
C-CT--0.1490	-1	-6.571109	10.212290	-0.038755	L
H-H1-0.0976	0	-6.993793	10.893694	0.700971	L
H-H1-0.0976	0	-6.861181	10.543362	-1.038245	L
H-H1-0.0976	0	-6.965765	9.208790	0.130088	L
C-CT--0.3662	-1	-4.412795	8.910828	-5.296151	L
H-HC-0.1123	0	-3.979339	9.278832	-4.367024	L
H-HC-0.1123	0	-3.651488	8.900874	-6.074162	L
H-HC-0.1123	0	-5.237125	9.550475	-5.603166	L
C-C-0.5972	0	-4.928535	7.492825	-5.094681	L
O-O--0.5679	0	-4.646225	6.621829	-5.904967	L
N-N--0.415700	0	-5.695631	7.262584	-4.024951	L
H-H-0.271900	0	-5.711029	8.015016	-3.347711	L
C-CT--0.087500	-1	-6.371693	5.980304	-3.691359	L
H-H1-0.096900	0	-6.091782	5.229901	-4.430101	L
C-C-0.597300	0	-7.883363	6.149316	-3.794515	L
O-O--0.567900	0	-8.526067	5.444015	-4.561353	L
C-CT-0.298500	0	-5.914630	5.484908	-2.304882	L
H-HC--0.029700	0	-6.094135	6.261109	-1.560706	L
C-CT--0.319200	0	-6.630081	4.212869	-1.843315	L
H-HC-0.079100	0	-6.444809	3.425245	-2.567292	L
H-HC-0.079100	0	-7.698366	4.398809	-1.745679	L
H-HC-0.079100	0	-6.241701	3.900970	-0.872368	L
C-CT--0.319200	0	-4.420760	5.164583	-2.347476	L
H-HC-0.079100	0	-4.215123	4.461390	-3.148959	L
H-HC-0.079100	0	-4.109872	4.729174	-1.401363	L
H-HC-0.079100	0	-3.846727	6.074251	-2.521987	L
N-N--0.4157	0	-8.465836	7.077278	-3.028412	L
H-H-0.2719	0	-7.858597	7.636009	-2.449402	L
C-CT--0.1490	-1	-9.898519	7.354295	-3.067362	L
H-H1-0.0976	0	-10.150882	8.149313	-2.364879	L

H-H1-0.0976	0	-10.183495	7.661994	-4.076045	L
H-H1-0.0976	0	-10.457066	6.451901	-2.811874	L
C-CT-0.1200	-1	-8.872118	2.020041	3.172058	L
H-HC-0.0800	0	-8.129262	2.799369	3.336341	L
H-HC-0.0800	0	-9.632097	2.388232	2.482944	L
H-HC-0.0800	0	-9.351970	1.765806	4.117901	L
C-CT-0.2000	0	-8.235583	0.812047	2.592790	L
H-H1-0.0800	0	-9.013337	0.072029	2.383032	L
O-OS--0.5600	0	-7.530646	1.180270	1.426742	L
C-CT-0.2000	0	-7.184416	0.241495	3.534922	L
H-H1-0.0800	0	-6.479418	1.011151	3.844135	L
O-OH--0.6800	0	-7.724420	-0.403644	4.684620	L
H-HO-0.4000	0	-8.499700	-0.921184	4.405304	L
C-CT-0.2000	0	-6.537999	-0.667367	2.501595	L
H-H1-0.0800	0	-5.529860	-0.960839	2.795478	L
O-OH--0.6800	0	-7.408772	-1.785179	2.367373	L
H-HO-0.4000	0	-6.954467	-2.436732	1.790716	L
C-CT-0.5691	0	-6.548800	0.171548	1.214835	H
H-H2-0.8000	0	-6.794775	-0.502400	0.374300	H
N-N*--0.5691	0	-5.296480	0.910864	0.949882	H
C-CM--0.0500	0	-4.708777	0.689138	-0.227747	H
H-H4-0.1500	0	-5.121238	-0.127346	-0.764668	H
C-CM--0.1238	0	-3.721392	1.451610	-0.762153	H
C-C-0.6156	0	-3.457684	1.420070	-2.227895	H
O-O--0.5700	0	-2.830106	2.294976	-2.757305	H
N-N--0.8000	0	-4.107381	0.467197	-2.951244	H
H-H-0.3700	0	-3.850546	0.368017	-3.915141	H
H-H-0.3700	0	-4.365009	-0.394703	-2.523544	H
C-CT-0.1164	0	-3.112761	2.438628	0.067583	H
H-HC-0.0800	0	-2.534450	3.206730	-0.410583	H
H-HC-0.0800	0	-1.891757	1.775627	0.597500	H
C-CM--0.2882	0	-3.848027	2.760626	1.266129	H
H-HA-0.1500	0	-3.546431	3.613614	1.835761	H
C-CM--0.0500	0	-4.872346	2.001295	1.675475	H
H-H4-0.1500	0	-5.414885	2.237353	2.562647	H
N-N3--0.8530	0	3.263097	0.951294	-0.468539	H
H-H-0.4500	0	3.441725	0.755244	-1.440876	H
H-H-0.4500	0	2.844111	0.973884	2.003263	H
H-H-0.4500	0	4.119185	0.740177	0.027110	H
C-CT-0.3170	0	3.297102	2.405988	-0.336769	H
H-HP-0.0800	0	4.009362	2.833020	-1.040046	H
C-CT--0.1600	0	1.956393	3.108468	-0.575907	H
H-HC-0.0800	0	1.693533	2.972227	-1.619534	H
H-HC-0.0800	0	2.140366	4.169276	-0.446178	H
C-CT--0.1600	0	0.793391	2.676101	0.329823	H
H-HC-0.0800	0	0.077610	3.489328	0.397162	H
H-HC-0.0800	0	1.181397	2.514339	1.326033	H
C-CT--0.0990	0	0.099475	1.406983	-0.156434	H
H-HC-0.0800	0	0.833179	0.625262	-0.280487	H
H-HC-0.0800	0	-0.330627	1.602171	-1.133624	H
C-C-0.4490	0	-0.991103	0.896244	0.769817	H
H-H-0.0600	0	-0.809645	1.053524	1.825910	H
O-O--0.5700	0	2.252555	0.199993	2.064037	H
N-N3--0.9900	0	-1.522642	-0.316916	0.460001	H
H-H-0.3600	0	2.268237	-0.001541	1.133021	H

H-H-0.3600	0	-1.170850	-0.696530	-0.397792	H
C-C-0.9060	0	3.907681	2.717491	1.042591	L
O-O2--0.9000	0	4.245101	1.797995	1.825566	L
O-O2--0.9000	0	4.047768	3.898998	1.420490	L
C-CT--0.205900	0	-2.184845	-1.226693	1.391159	H
H-H1-0.139900	0	-2.860998	-0.628268	1.992412	H
C-CT-0.007100	0	-2.977864	-2.216332	0.536341	H
H-HC--0.007800	0	-2.285906	-2.836294	-0.018928	H
H-HC--0.007800	0	-3.586478	-1.680570	-0.188650	H
C-CT-0.067500	0	-3.870785	-3.126678	1.365241	L
H-HC--0.054800	0	-4.169277	-2.596905	2.268800	L
H-HC--0.054800	0	-3.319168	-4.023403	1.655292	L
C-C-0.818300	0	-5.139164	-3.485415	0.603427	L
O-O2--0.822000	0	-5.993878	-2.574967	0.445819	L
O-O2--0.822000	0	-5.319915	-4.648060	0.182926	L
C-C-0.742000	0	-1.220663	-2.046804	2.297382	H
O-O2--0.793000	0	-1.673212	-2.406477	3.385232	H
O-O2--0.793000	0	-0.167600	-2.363092	1.756700	H
O-OW--0.834000	0	3.038875	-5.988631	-3.871509	L
H-HW-0.417000	0	3.830350	-6.075677	-3.301550	L
H-HW-0.417000	0	2.329353	-5.825603	-3.230496	L
O-OW--0.834000	0	-1.648130	-7.516130	2.499098	L
H-HW-0.417000	0	-2.382227	-7.132989	1.975234	L
H-HW-0.417000	0	-1.976646	-8.396366	2.695642	L
O-OW--0.834000	0	4.457671	0.050512	3.826822	L
H-HW-0.417000	0	3.626709	-0.406980	3.647192	L
H-HW-0.417000	0	4.563795	0.622158	3.037936	L
O-OW--0.834000	0	6.421829	-1.544669	4.715644	L
H-HW-0.417000	0	6.019249	-2.430651	4.767281	L
H-HW-0.417000	0	5.667897	-0.986585	4.442093	L
O-OW--0.834000	0	-4.028403	-6.679187	1.405188	L
H-HW-0.417000	0	-4.510829	-6.085296	0.793043	L
H-HW-0.417000	0	-4.433478	-6.447701	2.249731	L
O-OW--0.834000	0	-9.273386	-0.544159	-0.030150	L
H-HW-0.417000	0	-9.183101	-0.043687	-0.856631	L
H-HW-0.417000	0	-9.238727	-1.480641	-0.299511	L

PC

C-CT--0.3662	-1	-12.886546	1.231516	1.841092	L
H-HC-0.1123	0	-12.562783	2.248194	1.624143	L
H-HC-0.1123	0	-13.944298	1.133316	1.603930	L
H-HC-0.1123	0	-12.298893	0.528754	1.252744	L
C-C-0.5972	0	-12.665172	0.948177	3.314262	L
O-O--0.5679	0	-11.991840	1.725827	3.980486	L
N-N--0.415700	0	-13.209860	-0.168285	3.808735	L
H-H-0.271900	0	-13.723886	-0.743265	3.161608	L
C-CT--0.025200	-1	-12.949964	-0.700995	5.158944	L
H-H1-0.069800	0	-13.687332	-1.469046	5.391264	L
H-H1-0.069800	0	-13.036060	0.105050	5.888365	L
C-C-0.597300	0	-11.523602	-1.320669	5.274370	L
O-O--0.567900	0	-10.656318	-1.108861	4.421177	L
N-N--0.415700	0	-11.261803	-2.122417	6.318294	L
H-H-0.271900	0	-12.006270	-2.289487	6.976459	L
C-CT--0.025200	-1	-10.069097	-2.996716	6.400421	L
H-H1-0.069800	0	-9.976353	-3.397404	7.409436	L

H-H1-0.069800 0 -9.171600 -2.424209 6.161573 L
C-C-0.597300 0 -10.223940 -4.157162 5.430280 L
O-O--0.567900 0 -11.266709 -4.804288 5.390451 L
N-N--0.4157 0 -9.175900 -4.419245 4.662693 L
H-H-0.2719 0 -8.357220 -3.845277 4.789606 L
C-CT--0.0014 -1 -9.105055 -5.377704 3.559653 L
H-H1-0.0876 0 -9.914298 -5.186070 2.854184 L
C-C-0.5973 0 -9.169468 -6.870408 3.946347 L
O-O--0.5679 0 -8.691703 -7.731356 3.207413 L
C-CT--0.0152 0 -7.764447 -5.103883 2.861142 L
H-HC-0.0295 0 -7.839065 -4.141203 2.371349 L
H-HC-0.0295 0 -7.620749 -5.815192 2.046746 L
C-CA--0.0011 0 -6.518883 -5.114257 3.750005 L
C-CA--0.1906 0 -5.792044 -6.305931 3.938817 L
H-HA-0.1699 0 -6.179157 -7.242291 3.558616 L
C-CA--0.1906 0 -6.020361 -3.916355 4.305247 L
H-HA-0.1699 0 -6.579484 -3.001023 4.258158 L
C-CA--0.2341 0 -4.514948 -6.272005 4.533162 L
H-HA-0.1656 0 -3.932872 -7.176676 4.629169 L
C-CA--0.2341 0 -4.721384 -3.864107 4.840183 L
H-HA-0.1656 0 -4.280383 -2.919520 5.125186 L
C-C-0.3326 0 -3.943555 -5.034781 4.897265 L
O-OH--0.5579 0 -2.620169 -4.955462 5.179587 L
H-HO-0.3992 0 -2.271818 -4.088120 4.871254 L
N-N--0.4157 0 -9.737876 -7.197683 5.104949 L
H-H-0.2719 0 -10.233631 -6.458797 5.586389 L
C-CT--0.1490 -1 -9.722880 -8.541254 5.647247 L
H-H1-0.0976 0 -8.692084 -8.866382 5.800387 L
H-H1-0.0976 0 -10.256176 -8.568670 6.598183 L
H-H1-0.0976 0 -10.204346 -9.225918 4.945906 L
C-CT--0.3662 -1 -12.034961 -1.318233 -3.545662 L
H-HC-0.1123 0 -12.541906 -0.390747 -3.806360 L
H-HC-0.1123 0 -12.048679 -1.473733 -2.468613 L
H-HC-0.1123 0 -12.539013 -2.151043 -4.035265 L
C-C-0.5972 0 -10.601298 -1.267257 -4.034826 L
O-O--0.5679 0 -10.155293 -2.095048 -4.817601 L
N-N--0.415700 0 -9.856800 -0.292554 -3.530809 L
H-H-0.271900 0 -10.327909 0.352380 -2.914285 L
C-CT--0.038900 -1 -8.420398 -0.014630 -3.755615 L
H-H1-0.100700 0 -8.179603 -0.065185 -4.815683 L
C-C-0.597300 0 -7.546920 -1.013613 -2.982890 L
O-O--0.567900 0 -6.670447 -0.654115 -2.196718 L
C-CT-0.365400 0 -8.182372 1.415895 -3.236443 L
H-H1-0.004300 0 -7.129577 1.663759 -3.333543 L
O-OH--0.676100 0 -8.572784 1.521428 -1.882769 L
H-HO-0.410200 0 -8.382627 2.429094 -1.619290 L
C-CT--0.243800 0 -9.034249 2.484177 -3.918340 L
H-HC-0.064200 0 -10.091426 2.224325 -3.922894 L
H-HC-0.064200 0 -8.705915 2.633361 -4.939458 L
H-HC-0.064200 0 -8.929599 3.426096 -3.389541 L
N-N--0.415700 0 -7.824157 -2.298861 -3.141262 L
H-H-0.271900 0 -8.530108 -2.558094 -3.825321 L
C-CT--0.024900 -1 -7.257467 -3.365246 -2.320771 L
H-H1-0.084300 0 -6.393996 -3.005507 -1.773042 L
C-C-0.597300 0 -6.761380 -4.519572 -3.188696 L

O-O--0.567900	0	-7.195467	-4.691250	-4.325413	L
C-CT-0.211700	0	-8.304972	-3.947483	-1.368053	L
H-H1-0.035200	0	-7.992101	-4.934654	-1.021966	L
H-H1-0.035200	0	-9.271428	-4.034396	-1.867244	L
O-OH--0.654600	0	-8.393310	-3.100154	-0.257378	L
H-HO-0.427500	0	-7.467430	-3.040252	0.066371	L
N-N--0.4157	0	-5.856465	-5.329840	-2.646950	L
H-H-0.2719	0	-5.578435	-5.139478	-1.685542	L
C-CT--0.0014	0	-5.437895	-6.586946	-3.257065	L
H-H1-0.0876	0	-5.057657	-6.380902	-4.257436	L
C-C-0.5973	0	-6.583441	-7.595193	-3.374536	L
O-O--0.5679	0	-7.518040	-7.606704	-2.574837	L
C-CT--0.0152	0	-4.334049	-7.227064	-2.417530	L
H-HC-0.0295	0	-4.142998	-8.233689	-2.792007	L
H-HC-0.0295	0	-4.686591	-7.337373	-1.395899	L
C-CA--0.0011	-1	-3.020173	-6.491070	-2.433933	L
C-CA--0.1906	0	-2.203486	-6.560908	-3.583663	L
H-HA-0.1699	0	-2.558952	-7.078906	-4.462964	L
C-CA--0.1906	0	-2.531668	-5.841396	-1.284244	L
H-HA-0.1699	0	-3.134904	-5.797743	-0.389870	L
C-CA--0.2341	0	-0.913398	-5.991820	-3.582828	L
H-HA-0.1656	0	-0.281535	-6.053643	-4.455379	L
C-CA--0.2341	0	-1.242837	-5.274506	-1.276236	L
H-HA-0.1656	0	-0.874739	-4.799042	-0.381876	L
C-C-0.3226	0	-0.430289	-5.352243	-2.423249	L
O-OH--0.5579	0	0.820553	-4.820829	-2.398037	L
H-HO-0.3992	0	0.888138	-4.224435	-1.648943	L
N-N--0.4157	0	-6.435150	-8.509901	-4.335841	L
H-H-0.2719	0	-5.647986	-8.408808	-4.954363	L
C-CT--0.1490	-1	-7.278067	-9.682448	-4.465546	L
H-H1-0.0976	0	-8.313608	-9.372222	-4.622364	L
H-H1-0.0976	0	-6.954783	-10.292922	-5.309510	L
H-H1-0.0976	0	-7.230869	-10.275239	-3.549680	L
C-CT--0.3662	-1	-6.381525	-3.464235	-7.003242	L
H-HC-0.1123	0	-5.914498	-4.231435	-7.617268	L
H-HC-0.1123	0	-7.426778	-3.722545	-6.837573	L
H-HC-0.1123	0	-5.864614	-3.392253	-6.051961	L
C-C-0.5972	0	-6.281779	-2.125670	-7.717257	L
O-O--0.5679	0	-5.383015	-1.966770	-8.531432	L
N-N--0.415700	0	-7.173721	-1.170706	-7.414271	L
H-H-0.271900	0	-7.900256	-1.435106	-6.765957	L
C-CT--0.025200	-1	-7.286083	0.146933	-8.089689	L
H-H1-0.069800	0	-8.229197	0.175603	-8.635776	L
H-H1-0.069800	0	-6.488665	0.266123	-8.822550	L
C-C-0.597300	0	-7.255004	1.352834	-7.154661	L
O-O--0.567900	0	-8.141302	2.192008	-7.240560	L
N-N--0.415700	0	-6.244205	1.439651	-6.283207	L
H-H-0.271900	0	-5.558464	0.692789	-6.310572	L
C-CT--0.025200	-1	-5.799541	2.683435	-5.640788	L
H-H1-0.069800	0	-5.627202	2.526080	-4.581759	L
H-H1-0.069800	0	-6.553852	3.462512	-5.761576	L
C-C-0.597300	0	-4.506776	3.176279	-6.301489	L
O-O--0.567900	0	-4.442346	3.292638	-7.522124	L
N-N--0.415700	0	-3.461751	3.402683	-5.503481	L
H-H-0.271900	0	-3.582795	3.268259	-4.508561	L

C-CT--0.051800 -1 -2.117249 3.769484 -5.960492 L
H-H1-0.092200 0 -2.189352 4.202834 -6.959529 L
C-C-0.597300 0 -1.209362 2.527150 -6.054049 L
O-O--0.567900 0 -0.512730 2.303920 -7.041359 L
C-CT--0.110200 0 -1.571337 4.840392 -4.995434 L
H-HC-0.045700 0 -1.559470 4.429800 -3.985550 L
H-HC-0.045700 0 -2.258280 5.688348 -4.999592 L
C-CT-0.353100 0 -0.158389 5.352860 -5.320845 L
H-HC--0.036100 0 0.545165 4.520465 -5.302408 L
C-CT--0.412100 0 -0.080755 6.039684 -6.684316 L
H-HC-0.100000 0 -0.821700 6.837967 -6.742955 L
H-HC-0.100000 0 -0.273742 5.315240 -7.475466 L
H-HC-0.100000 0 0.914515 6.455849 -6.835794 L
C-CT--0.412100 0 0.265336 6.361965 -4.251437 L
H-HC-0.100000 0 -0.424381 7.206964 -4.245236 L
H-HC-0.100000 0 1.272327 6.721736 -4.460894 L
H-HC-0.100000 0 0.256285 5.883534 -3.273032 L
N-N--0.516300 0 -1.186785 1.725384 -4.991539 L
H-H-0.293600 0 -1.774492 2.011595 -4.213881 L
C-CT-0.038100 -1 -0.304194 0.577948 -4.780163 L
H-H1-0.088000 0 -0.083275 0.075655 -5.719716 L
C-C-0.536600 0 -0.981411 -0.395724 -3.781116 L
O-O--0.581900 0 -0.798479 -0.254901 -2.568758 L
C-CT--0.030300 0 1.012101 1.154357 -4.186569 L
H-HC--0.012200 0 0.768535 1.798441 -3.340385 L
H-HC--0.012200 0 1.482403 1.783675 -4.944223 L
C-C-0.799400 -1 2.052173 0.127736 -3.718548 L
O-O2--0.801400 0 1.878371 -1.083122 -3.996243 L
O-O2--0.801400 0 3.133946 0.532177 -3.247866 L
N-N--0.254800 0 -1.782245 -1.373060 -4.247820 L
C-CT--0.026600 -1 -2.077782 -1.681123 -5.649259 L
H-H1-0.064100 0 -1.154069 -1.762069 -6.221000 L
C-C-0.589600 0 -2.999547 -0.642755 -6.316919 L
O-O--0.574800 0 -3.902791 -0.099286 -5.677277 L
C-CT--0.007000 0 -2.751007 -3.057328 -5.602983 L
H-HC-0.025300 0 -1.986109 -3.835407 -5.599501 L
H-HC-0.025300 0 -3.439007 -3.210609 -6.434914 L
C-CT-0.018900 0 -3.471996 -3.052502 -4.255207 L
H-HC-0.021300 0 -3.644931 -4.060717 -3.884391 L
H-HC-0.021300 0 -4.411866 -2.504252 -4.333302 L
C-CT-0.019200 0 -2.503139 -2.281072 -3.364520 L
H-H1-0.039100 0 -3.049145 -1.735413 -2.593301 L
H-H1-0.039100 0 -1.796355 -2.968549 -2.901381 L
N-N--0.4157 0 -2.769663 -0.396078 -7.611294 L
H-H-0.2719 0 -2.021501 -0.901253 -8.053775 L
C-CT--0.1490 -1 -3.534152 0.507462 -8.470724 L
H-H1-0.0976 0 -4.494111 0.742896 -8.024761 L
H-H1-0.0976 0 -3.713111 0.039691 -9.439882 L
H-H1-0.0976 0 -2.981956 1.438545 -8.616815 L
C-CT--0.3662 -1 8.763700 1.768680 -5.958321 L
H-HC-0.1123 0 9.268365 2.002249 -6.895639 L
H-HC-0.1123 0 9.508207 1.563565 -5.191810 L
H-HC-0.1123 0 8.114889 0.907429 -6.102956 L
C-C-0.5972 0 7.935667 2.964504 -5.542845 L
O-O--0.5679 0 8.049578 4.021980 -6.144064 L

N-N--0.415700	0	7.101211	2.803957	-4.515453	L
H-H-0.271900	0	7.050432	1.893032	-4.085484	L
C-CT-0.021300	-1	6.115675	3.808758	-4.094136	L
H-H1-0.112400	0	6.327352	4.759854	-4.587385	L
C-C-0.597300	0	6.133666	4.049399	-2.581044	L
O-O--0.567900	0	6.361533	3.126294	-1.796281	L
C-CT--0.123100	0	4.741421	3.315372	-4.575656	L
H-H1-0.111200	0	4.538225	2.332275	-4.147991	L
H-H1-0.111200	0	4.755039	3.225314	-5.663533	L
S-SH--0.311900	0	3.417045	4.460101	-4.092915	L
H-HS-0.193300	0	2.412914	3.819333	-4.708711	L
N-N--0.415700	0	5.885741	5.294299	-2.172593	L
H-H-0.271900	0	5.658108	5.984820	-2.874995	L
C-CT--0.025200	-1	5.873293	5.723270	-0.779521	L
H-H1-0.069800	0	6.833995	6.181365	-0.544974	L
H-H1-0.069800	0	5.748047	4.866851	-0.120547	L
C-C-0.597300	0	4.784182	6.749647	-0.495460	L
O-O--0.567900	0	4.898082	7.898339	-0.915490	L
N-N--0.415700	0	3.758729	6.370443	0.268928	L
H-H-0.271900	0	3.781907	5.419472	0.636960	L
C-CT--0.025200	-1	2.756934	7.307607	0.789065	L
H-H1-0.069800	0	1.874200	6.748202	1.085138	L
H-H1-0.069800	0	2.483158	8.042933	0.031352	L
C-C-0.597300	0	3.294907	8.019603	2.025460	L
O-O--0.567900	0	2.810671	7.774712	3.130084	L
N-N--0.4157	0	4.322485	8.851863	1.819787	L
H-H-0.2719	0	4.620717	8.930884	0.852167	L
C-CT--0.1490	-1	5.108378	9.522285	2.848438	L
H-H1-0.0976	0	5.500813	8.786455	3.552454	L
H-H1-0.0976	0	5.937496	10.065249	2.391974	L
H-H1-0.0976	0	4.476019	10.225804	3.393446	L
C-CT--0.3662	-1	-1.424152	9.523652	5.150437	L
H-HC-0.1123	0	-0.507889	8.946104	5.260385	L
H-HC-0.1123	0	-1.642304	10.053293	6.076026	L
H-HC-0.1123	0	-1.310275	10.242682	4.341298	L
C-C-0.5972	0	-2.597043	8.590172	4.827555	L
O-O--0.5679	0	-3.407753	8.919782	3.971876	L
N-N--0.4157	0	-2.659062	7.411584	5.459352	L
H-H-0.2719	0	-1.971430	7.231731	6.178297	L
C-CT--0.0275	-1	-3.561278	6.259134	5.193285	L
H-H1-0.1123	0	-4.573387	6.617613	5.004642	L
C-C-0.5973	0	-3.584001	5.324478	6.431815	L
O-O--0.5679	0	-2.762565	5.478724	7.335495	L
C-CT--0.0050	0	-3.044231	5.485626	3.961606	L
H-HC-0.0339	0	-3.340563	6.035315	3.074602	L
H-HC-0.0339	0	-3.573159	4.534478	3.896967	L
C-C*--0.1415	0	-1.560538	5.203677	3.883947	L
C-CW--0.1638	0	-0.574957	6.111237	3.667380	L
H-H4-0.2062	0	-0.718863	7.171924	3.525984	L
C-CB-0.1243	0	-0.875044	3.921477	4.013665	L
N-NA--0.3418	0	0.659289	5.494782	3.673230	L
H-H-0.3412	0	1.536421	6.003476	3.588058	L
C-CN-0.1380	0	0.530128	4.137801	3.869177	L
C-CA--0.238	0	-1.297661	2.595710	4.249493	L
H-HA-0.1700	0	-2.351066	2.382197	4.360551	L

C-CA--0.2601	0	1.463517	3.096427	3.953480	L
H-HA-0.1572	0	2.514173	3.310277	3.842888	L
C-CA--0.1972	0	-0.370542	1.541086	4.330892	L
H-HA-0.1447	0	-0.731681	0.535173	4.512846	L
C-CA--0.1134	0	1.006539	1.789211	4.191759	L
H-HA-0.1417	0	1.708575	0.972857	4.271262	L
N-N--0.4157	0	-4.507311	4.349053	6.486336	L
H-H-0.2719	0	-5.136162	4.276781	5.702696	L
C-CT--0.1490	-1	-4.647676	3.383098	7.589972	L
H-H1-0.0976	0	-3.698944	2.867731	7.748634	L
H-H1-0.0976	0	-4.915158	3.910348	8.507963	L
H-H1-0.0976	0	-5.421172	2.649820	7.356224	L
C-CT--0.3662	-1	-2.859335	-1.362292	7.231846	L
H-HC-0.1123	0	-3.313895	-0.940413	6.335446	L
H-HC-0.1123	0	-3.094272	-2.424930	7.276802	L
H-HC-0.1123	0	-3.232803	-0.863645	8.122744	L
C-C-0.5972	0	-1.366549	-1.199681	7.125506	L
O-O--0.5679	0	-0.662717	-2.175357	6.935010	L
N-N--0.415700	0	-0.853919	0.009039	7.351344	L
H-H-0.271900	0	-1.512324	0.768286	7.421831	L
C-CT--0.087500	-1	0.592702	0.319809	7.369021	L
H-H1-0.096900	0	0.971206	0.284415	6.351441	L
C-C-0.597300	0	1.403827	-0.683865	8.197987	L
O-O--0.567900	0	2.377062	-1.241922	7.704327	L
C-CT-0.298500	0	0.863517	1.727855	7.972053	L
H-HC--0.029700	0	0.744521	1.663677	9.054087	L
C-CT--0.319200	0	2.302193	2.182514	7.688641	L
H-HC-0.079100	0	2.464162	2.259202	6.614049	L
H-HC-0.079100	0	3.016310	1.472805	8.106339	L
H-HC-0.079100	0	2.479178	3.156393	8.145738	L
C-CT--0.319200	0	-0.073896	2.856856	7.527051	L
H-HC-0.079100	0	0.096426	3.096335	6.484469	L
H-HC-0.079100	0	0.130802	3.753627	8.112606	L
H-HC-0.079100	0	-1.118919	2.595021	7.678576	L
N-N--0.415700	0	1.000410	-0.912010	9.457405	L
H-H-0.271900	0	0.192209	-0.398858	9.769985	L
C-CT--0.025200	-1	1.597561	-1.899186	10.368406	L
H-H1-0.069800	0	1.027982	-1.940645	11.296118	L
H-H1-0.069800	0	2.621194	-1.603140	10.600264	L
C-C-0.597300	0	1.637011	-3.321207	9.755205	L
O-O--0.567900	0	2.541556	-4.087656	10.064694	L
N-N--0.415700	0	0.672814	-3.672238	8.889691	L
H-H-0.271900	0	0.019134	-2.958294	8.600810	L
C-CT-0.033700	-1	0.559501	-4.943467	8.162303	L
H-H1-0.082300	0	0.989853	-5.723865	8.792785	L
C-C-0.597300	0	1.359709	-4.994933	6.835898	L
O-O--0.567900	0	1.970822	-6.015298	6.525818	L
C-CT--0.182500	0	-0.921678	-5.290481	7.981054	L
H-HC-0.060300	0	-1.399694	-5.389122	8.955774	L
H-HC-0.060300	0	-1.013223	-6.236053	7.445098	L
H-HC-0.060300	0	-1.428087	-4.513360	7.414893	L
N-N--0.415700	0	1.418746	-3.900535	6.069487	L
H-H-0.271900	0	0.805291	-3.134951	6.328971	L
C-CT-0.033700	-1	2.379094	-3.685704	4.973219	L
H-H1-0.082300	0	2.325032	-4.532293	4.289984	L

C-C-0.597300	0	3.842625	-3.574889	5.451569	L
O-O--0.567900	0	4.757795	-3.514632	4.626724	L
C-CT--0.182500	0	1.950820	-2.413216	4.232078	L
H-HC-0.060300	0	2.047110	-1.552448	4.894523	L
H-HC-0.060300	0	0.909828	-2.501423	3.925216	L
H-HC-0.060300	0	2.575576	-2.276741	3.350203	L
N-N--0.4157	0	4.068009	-3.503782	6.766770	L
H-H-0.2719	0	3.246578	-3.460758	7.353723	L
C-CT--0.1490	-1	5.362562	-3.470069	7.421718	L
H-H1-0.0976	0	6.034061	-4.195308	6.958419	L
H-H1-0.0976	0	5.243096	-3.719898	8.477797	L
H-H1-0.0976	0	5.795256	-2.472299	7.335852	L
C-CT--0.3662	-1	14.078747	0.770042	-0.713823	L
H-HC-0.1123	0	13.469436	1.303216	0.013004	L
H-HC-0.1123	0	15.094464	1.162821	-0.702767	L
H-HC-0.1123	0	13.654049	0.879846	-1.709502	L
C-C-0.5972	0	14.117725	-0.705748	-0.350777	L
O-O--0.5679	0	15.191779	-1.279284	-0.234250	L
N-N--0.4157	0	12.949244	-1.327286	-0.194143	L
H-H-0.2719	0	12.115101	-0.766973	-0.321764	L
C-CT--0.0014	-1	12.753656	-2.779564	0.001820	L
H-H1-0.0876	0	13.634845	-3.306851	-0.366572	L
C-C-0.5973	0	11.537189	-3.259955	-0.840970	L
O-O--0.5679	0	10.803911	-2.452424	-1.420742	L
C-CT--0.0152	0	12.578383	-3.119229	1.493240	L
H-HC-0.0295	0	13.530265	-2.936768	1.994472	L
H-HC-0.0295	0	12.368034	-4.183906	1.599726	L
C-CA--0.0011	-1	11.498762	-2.328746	2.211512	L
C-CA--0.1906	0	11.847398	-1.099107	2.802078	L
H-HA-0.1699	0	12.870363	-0.750774	2.751931	L
C-CA--0.1906	0	10.167529	-2.786443	2.278185	L
H-HA-0.1699	0	9.893375	-3.734506	1.836284	L
C-CA--0.2341	0	10.874856	-0.324615	3.455771	L
H-HA-0.1656	0	11.148246	0.606680	3.919914	L
C-CA--0.2341	0	9.189001	-2.012205	2.935672	L
H-HA-0.1656	0	8.170089	-2.364850	2.997452	L
C-C-0.3226	0	9.542664	-0.778623	3.527430	L
O-OH--0.557	0	8.614096	-0.023314	4.170472	L
H-HO-0.3992	0	7.821911	-0.565610	4.369836	L
N-N--0.415700	0	11.302205	-4.576032	-0.937890	L
H-H-0.271900	0	11.904569	-5.196227	-0.419204	L
C-CT--0.025200	-1	10.170148	-5.169602	-1.678388	L
H-H1-0.069800	0	10.368699	-6.228336	-1.844108	L
H-H1-0.069800	0	10.071807	-4.689904	-2.651229	L
C-C-0.597300	0	8.848191	-5.057429	-0.905929	L
O-O--0.567900	0	8.779784	-5.430909	0.263574	L
N-N--0.415700	0	7.782581	-4.568723	-1.546475	L
H-H-0.271900	0	7.865478	-4.455706	-2.549555	L
C-CT-0.014300	-1	6.442551	-4.529386	-0.963438	L
H-H1-0.104800	0	6.537696	-4.438662	0.121544	L
C-C-0.597300	0	5.686333	-5.829415	-1.238235	L
O-O--0.567900	0	4.955558	-5.946735	-2.227167	L
C-CT--0.204100	0	5.656225	-3.291599	-1.425752	L
H-HC-0.079700	0	5.545970	-3.320999	-2.509776	L
H-HC-0.079700	0	6.243542	-2.424878	-1.160723	L

C-C-0.713000	-1	4.262830	-3.140472	-0.797204	L
O-O--0.593100	0	3.820703	-3.853722	0.093453	L
N-N--0.919100	0	3.469363	-2.230871	-1.323229	L
H-H-0.419600	0	2.531049	-2.186157	-0.979042	L
H-H-0.419600	0	3.734074	-1.658940	-2.118092	L
N-N--0.347900	0	5.834780	-6.775373	-0.306552	L
H-H-0.274700	0	6.519749	-6.561307	0.408296	L
C-CT--0.263700	-1	5.252061	-8.123334	-0.347637	L
H-H1-0.156000	0	5.615735	-8.662726	0.527291	L
C-C-0.734100	0	5.744267	-8.937185	-1.554923	L
O-O--0.589400	0	6.744728	-8.602692	-2.182972	L
C-CT--0.000700	0	3.712506	-8.049019	-0.250014	L
H-HC-0.032700	0	3.304789	-9.059641	-0.205489	L
H-HC-0.032700	0	3.321100	-7.576708	-1.152201	L
C-CT-0.039000	0	3.185716	-7.299413	0.980645	L
H-HC-0.028500	0	3.625938	-6.306183	1.054738	L
H-HC-0.028500	0	3.416816	-7.866317	1.883677	L
C-CT-0.048600	0	1.672346	-7.166980	0.814317	L
H-H1-0.068700	0	1.246035	-8.155766	0.633501	L
H-H1-0.068700	0	1.457243	-6.546401	-0.058184	L
N-N2--0.529500	-1	1.022147	-6.600328	1.998496	L
H-H-0.345600	0	0.500478	-7.229506	2.588631	L
C-CA-0.807600	0	0.689256	-5.336585	2.187901	L
N-N2--0.862700	0	-0.275652	-5.046582	2.999766	L
H-H-0.447800	0	-0.912241	-5.780077	3.273943	L
H-H-0.447800	0	-0.611538	-4.085697	3.143782	L
N-N2--0.862700	0	1.261718	-4.344553	1.572614	L
H-H-0.447800	0	2.145326	-4.434751	1.088095	L
H-H-0.447800	0	0.768498	-3.459978	1.687539	L
N-N--0.4157	0	5.056566	-10.045146	-1.837167	L
H-H-0.2719	0	4.269603	-10.250225	-1.245385	L
C-CT--0.1490	-1	5.349586	-10.942161	-2.940854	L
H-H1-0.0976	0	6.362836	-11.336625	-2.836844	L
H-H1-0.0976	0	4.641921	-11.772584	-2.953662	L
H-H1-0.0976	0	5.284466	-10.398787	-3.885718	L
C-CT--0.3662	-1	10.074018	-6.101752	-7.744915	L
H-HC-0.1123	0	9.776766	-6.784975	-8.538129	L
H-HC-0.1123	0	10.987385	-5.585474	-8.039097	L
H-HC-0.1123	0	10.252264	-6.647870	-6.820153	L
C-C-0.5972	0	8.982587	-5.077356	-7.522548	L
O-O--0.5679	0	9.232132	-3.883719	-7.652861	L
N-N--0.347900	0	7.776832	-5.513561	-7.128611	L
H-H-0.274700	0	7.672903	-6.508830	-6.999311	L
C-CT--0.263700	-1	6.805658	-4.597387	-6.498224	L
H-H1-0.156000	0	6.661312	-3.735479	-7.150562	L
C-C-0.734100	0	7.432898	-4.093963	-5.196985	L
O-O--0.589400	0	7.744221	-4.890888	-4.318757	L
C-CT--0.000700	0	5.443845	-5.258573	-6.234474	L
H-HC-0.032700	0	4.865400	-4.568184	-5.622209	L
H-HC-0.032700	0	5.573725	-6.177124	-5.658349	L
C-CT-0.039000	0	4.669558	-5.564365	-7.529921	L
H-HC-0.028500	0	4.853192	-6.604324	-7.802836	L
H-HC-0.028500	0	5.034872	-4.936764	-8.344399	L
C-CT-0.048600	0	3.153476	-5.343098	-7.389300	L
H-H1-0.068700	0	2.795558	-5.816232	-6.474271	L

H-H1-0.068700	0	2.660360	-5.841488	-8.226038	L
N-N2--0.529500	0	2.795607	-3.906833	-7.431533	L
H-H-0.345600	0	2.792572	-3.469987	-8.336397	L
C-CA-0.807600	0	2.474212	-3.110531	-6.424530	L
N-N2--0.862700	0	2.395819	-3.494684	-5.189930	L
H-H-0.447800	0	2.588090	-4.450868	-4.908483	L
H-H-0.447800	0	2.160625	-2.781684	-4.495904	L
N-N2--0.862700	0	2.223090	-1.852125	-6.618626	L
H-H-0.447800	0	2.261700	-1.391538	-7.506565	L
H-H-0.447800	0	2.059307	-1.335638	-5.752837	L
N-N--0.415700	0	7.682956	-2.791685	-5.094743	L
H-H-0.271900	0	7.423371	-2.214170	-5.877200	L
C-CT--0.025200	-1	8.577528	-2.216884	-4.085978	L
H-H1-0.069800	0	9.427896	-1.769714	-4.601558	L
H-H1-0.069800	0	8.973914	-2.991187	-3.433851	L
C-C-0.597300	0	7.950398	-1.142590	-3.213468	L
O-O--0.567900	0	6.819102	-0.707818	-3.432597	L
N-N--0.415700	0	8.694408	-0.683660	-2.211620	L
H-H-0.271900	0	9.595387	-1.118685	-2.015697	L
C-CT--0.038900	-1	8.294345	0.464692	-1.401981	L
H-H1-0.100700	0	7.650615	1.081797	-2.025969	L
C-C-0.597300	0	9.442602	1.389416	-1.029105	L
O-O--0.567900	0	10.556149	0.965504	-0.725411	L
C-CT-0.365400	0	7.445570	0.036363	-0.191430	L
H-H1-0.004300	0	7.775958	-0.942281	0.159887	L
O-OH--0.676100	0	6.122371	-0.043078	-0.674514	L
H-HO-0.410200	0	6.191996	-0.315958	-1.605487	L
C-CT--0.243800	0	7.384644	0.993584	0.999571	L
H-HC-0.064200	0	7.005681	1.967021	0.688301	L
H-HC-0.064200	0	8.375166	1.103997	1.439926	L
H-HC-0.064200	0	6.722206	0.580326	1.760158	L
N-N--0.415700	0	9.124416	2.683465	-1.075008	L
H-H-0.271900	0	8.154738	2.902775	-1.281613	L
C-CT--0.025200	-1	9.987273	3.796702	-0.726260	L
H-H1-0.069800	0	9.813823	4.614433	-1.425702	L
H-H1-0.069800	0	11.032491	3.492717	-0.805489	L
C-C-0.597300	0	9.698162	4.300562	0.671981	L
O-O--0.567900	0	8.553500	4.537553	1.064999	L
N-N--0.347900	0	10.770982	4.495384	1.421836	L
H-H-0.274700	0	11.677557	4.324039	1.001722	L
C-CT--0.263700	-1	10.780995	5.094755	2.747053	L
H-H1-0.156000	0	9.877934	5.683494	2.899561	L
C-C-0.734100	0	11.959136	6.041385	2.843624	L
O-O--0.589400	0	12.902812	5.951480	2.063430	L
C-CT--0.000700	0	10.925477	3.990459	3.818236	L
H-HC-0.032700	0	11.214143	4.435801	4.771998	L
H-HC-0.032700	0	11.730231	3.314481	3.521353	L
C-CT-0.039000	0	9.655893	3.165961	4.048945	L
H-HC-0.028500	0	9.901216	2.322516	4.692756	L
H-HC-0.028500	0	9.282121	2.781614	3.099879	L
C-CT-0.048600	0	8.579654	3.992943	4.753996	L
H-H1-0.068700	0	8.436256	4.943940	4.241457	L
H-H1-0.068700	0	8.907764	4.205718	5.773219	L
N-N2--0.529500	-1	7.304475	3.271782	4.797499	L
H-H-0.345600	0	7.138099	2.647051	5.568512	L

C-CA-0.807600	0	6.324772	3.379163	3.920463	L
N-N2--0.862700	0	5.190648	2.800426	4.127503	L
H-H-0.447800	0	5.057655	2.112263	4.849560	L
H-H-0.447800	0	4.610732	2.731789	3.290776	L
N-N2--0.862700	0	6.414587	4.040326	2.806278	L
H-H-0.447800	0	7.303597	4.329734	2.421720	L
H-H-0.447800	0	5.591280	4.026927	2.201374	L
N-N--0.4157	0	11.889602	6.924568	3.830113	L
H-H-0.2719	0	11.077339	6.918274	4.423346	L
C-CT--0.1490	-1	12.965714	7.838590	4.116022	L
H-H1-0.0976	0	12.711866	8.471476	4.967976	L
H-H1-0.0976	0	13.877756	7.282097	4.344628	L
H-H1-0.0976	0	13.157869	8.471982	3.246739	L
C-CT--0.3662	-1	-2.020082	13.286651	-1.701113	L
H-HC-0.1123	0	-2.475404	13.811791	-0.864038	L
H-HC-0.1123	0	-0.936076	13.279948	-1.606854	L
H-HC-0.1123	0	-2.295003	13.786051	-2.629927	L
C-C-0.5972	0	-2.534315	11.859500	-1.748248	L
O-O--0.5679	0	-3.146334	11.466434	-2.729015	L
N-N--0.415700	0	-2.294009	11.090431	-0.681494	L
H-H-0.271900	0	-1.765461	11.516748	0.060776	L
C-CT--0.023700	-1	-2.734509	9.688006	-0.518819	L
H-H1-0.088000	0	-2.274897	9.097403	-1.313121	L
C-C-0.597300	0	-4.256375	9.530749	-0.692400	L
O-O--0.567900	0	-4.727323	8.769488	-1.538542	L
C-CT-0.034200	0	-2.210340	9.170801	0.823032	L
H-HC-0.024100	0	-2.564715	9.820758	1.623458	L
H-HC-0.024100	0	-1.120477	9.206276	0.815397	L
C-CT-0.001800	0	-2.652916	7.742124	1.124835	L
H-H1-0.044000	0	-3.730646	7.742134	1.291664	L
H-H1-0.044000	0	-2.179975	7.456696	2.055107	L
S-S--0.273700	0	-2.280129	6.471358	-0.109827	L
C-CT--0.053600	0	-0.483027	6.355079	-0.031643	L
H-H1-0.068400	0	-0.141102	5.620252	-0.760646	L
H-H1-0.068400	0	-0.189678	6.031781	0.965557	L
H-H1-0.068400	0	-0.041372	7.323732	-0.262325	L
N-N--0.4157	0	-5.032848	10.265864	0.108034	L
H-H-0.2719	0	-4.570921	10.854632	0.782272	L
C-CT--0.1490	-1	-6.482408	10.309412	0.000338	L
H-H1-0.0976	0	-6.895811	10.977174	0.757526	L
H-H1-0.0976	0	-6.765667	10.671023	-0.990447	L
H-H1-0.0976	0	-6.892517	9.307888	0.142138	L
C-CT--0.3662	-1	-4.343271	9.021764	-5.268283	L
H-HC-0.1123	0	-3.875047	9.349549	-4.340895	L
H-HC-0.1123	0	-3.602316	9.010337	-6.065619	L
H-HC-0.1123	0	-5.154261	9.695434	-5.535204	L
C-C-0.5972	0	-4.898253	7.615534	-5.092151	L
O-O--0.5679	0	-4.663407	6.758885	-5.932591	L
N-N--0.415700	0	-5.645667	7.379707	-4.009862	L
H-H-0.271900	0	-5.619153	8.114173	-3.312403	L
C-CT--0.087500	-1	-6.333135	6.101723	-3.682600	L
H-H1-0.096900	0	-6.064612	5.354231	-4.428495	L
C-C-0.597300	0	-7.844486	6.278570	-3.771937	L
O-O--0.567900	0	-8.501592	5.552943	-4.507268	L
C-CT-0.298500	0	-5.871996	5.593762	-2.302760	L

H-HC--0.029700	0	-6.034901	6.367067	-1.551610	L
C-CT--0.319200	0	-6.595931	4.325920	-1.842998	L
H-HC-0.079100	0	-6.426941	3.540994	-2.574025	L
H-HC-0.079100	0	-7.660713	4.523906	-1.732407	L
H-HC-0.079100	0	-6.200922	4.007036	-0.877267	L
C-CT--0.319200	0	-4.382610	5.263591	-2.373090	L
H-HC-0.079100	0	-4.192495	4.608315	-3.216444	L
H-HC-0.079100	0	-4.059507	4.771801	-1.456671	L
H-HC-0.079100	0	-3.804741	6.177056	-2.509421	L
N-N--0.4157	0	-8.411581	7.230137	-3.023749	L
H-H-0.2719	0	-7.792361	7.802632	-2.471234	L
C-CT--0.1490	-1	-9.843961	7.510216	-3.045429	L
H-H1-0.0976	0	-10.080729	8.328937	-2.365040	L
H-H1-0.0976	0	-10.146599	7.785781	-4.058237	L
H-H1-0.0976	0	-10.400072	6.618771	-2.749741	L
C-CT-0.1200	0	-8.871304	2.120403	3.154730	H
H-HC-0.0800	0	-8.161487	2.912237	3.390214	H
H-HC-0.0800	0	-9.577551	2.477768	2.406108	H
H-HC-0.0800	0	-9.417193	1.843719	4.056856	H
C-CT-0.2000	0	-8.143040	0.923059	2.620504	H
H-H1-0.0800	0	-8.896063	0.186626	2.327543	H
O-OS--0.5600	0	-7.303227	1.270386	1.537285	H
C-CT-0.2000	0	-7.167808	0.308490	3.614937	H
H-H1-0.0800	0	-6.436507	1.052551	3.937706	H
O-OH--0.6800	0	-7.801475	-0.294798	4.739710	H
H-HO-0.4000	0	-8.547085	-0.829796	4.419072	H
C-CT-0.2000	0	-6.511627	-0.662083	2.636519	H
H-H1-0.0800	0	-5.546338	-1.005461	3.004415	H
O-OH--0.6800	0	-7.408442	-1.752357	2.446218	H
H-HO-0.4000	0	-6.951947	-2.416745	1.892777	H
C-CT-0.5691	0	-6.417526	0.159317	1.343612	H
H-H2-0.8000	0	-6.710302	-0.499224	0.506577	H
N-N*--0.5691	0	-5.102858	0.779851	1.038642	H
C-CM--0.0500	0	-4.985875	1.129560	-0.262335	H
H-H4-0.1500	0	-5.869157	0.994474	-0.844925	H
C-CM--0.1238	0	-3.834506	1.599092	-0.801175	H
C-C-0.6156	0	-3.649673	1.718407	-2.290306	H
O-O--0.5700	0	-2.871128	2.486730	-2.767055	H
N-N--0.8000	0	-4.433592	0.899604	-3.036530	H
H-H-0.3700	0	-4.165360	0.735766	-3.989547	H
H-H-0.3700	0	-4.870386	0.105158	-2.607372	H
C-CT-0.1164	0	-2.770578	1.854759	0.076897	H
H-HC-0.0800	0	-1.857213	2.266840	-0.307110	H
H-HC-0.0800	0	-0.169190	-0.309841	2.413175	H
C-CM--0.2882	0	-2.892830	1.489235	1.392078	H
H-HA-0.1500	0	-2.056727	1.539677	2.053811	H
C-CM--0.0500	0	-4.052428	0.876406	1.853128	H
H-H4-0.1500	0	-4.042696	0.403636	2.821136	H
N-N3--0.8530	0	3.443071	0.862319	-0.624427	H
H-H-0.4500	0	3.468206	0.830952	-1.630169	H
H-H-0.4500	0	4.303814	0.047052	1.604965	H
H-H-0.4500	0	4.409985	0.823681	-0.338934	H
C-CT-0.3170	0	3.093354	2.215102	-0.237249	H
H-HP-0.0800	0	3.480936	2.936180	-0.955152	H
C-CT--0.1600	0	1.585610	2.428081	-0.113760	H

H-HC-0.0800	0	1.144476	2.310410	-1.099046	H
H-HC-0.0800	0	1.441365	3.465000	0.173833	H
C-CT--0.1600	0	0.909560	1.502682	0.907421	H
H-HC-0.0800	0	0.105869	2.045506	1.395044	H
H-HC-0.0800	0	1.633246	1.300339	1.687412	H
C-CT--0.0990	0	0.392253	0.153899	0.399948	H
H-HC-0.0800	0	1.075793	-0.230935	-0.348483	H
H-HC-0.0800	0	-0.567896	0.283386	-0.092667	H
C-C-0.4490	0	0.280590	-0.821714	1.573837	H
H-H-0.0600	0	1.290258	-1.077056	1.876842	H
O-O--0.5700	0	4.148349	-0.906509	1.490842	H
N-N3--0.9900	0	-0.484727	-2.064686	1.323419	H
H-H-0.3600	0	3.747971	-0.838847	0.628507	H
H-H-0.3600	0	-0.487367	-2.158348	0.315963	H
C-C-0.9060	0	3.843129	2.541043	1.068942	H
O-O2--0.9000	0	4.125382	1.647882	1.908462	H
O-O2--0.9000	0	4.157522	3.726934	1.311825	H
C-CT--0.205900	0	-1.899145	-1.956134	1.707585	H
H-H1-0.139900	0	-2.333278	-1.050298	1.270297	H
C-CT-0.007100	0	-2.643871	-3.156871	1.104909	H
H-HC--0.007800	0	-2.068424	-4.044813	1.322865	H
H-HC--0.007800	0	-2.703512	-3.055028	0.020337	H
C-CT-0.067500	0	-4.052117	-3.410194	1.650564	L
H-HC--0.054800	0	-4.331774	-2.628796	2.351125	L
H-HC--0.054800	0	-4.028379	-4.351631	2.199134	L
C-C-0.818300	0	-5.155915	-3.494043	0.600598	L
O-O2--0.822000	0	-5.930042	-2.510168	0.459746	L
O-O2--0.822000	0	-5.355710	-4.573514	0.004538	L
C-C-0.742000	0	-2.100191	-1.884978	3.252405	H
O-O2--0.793000	0	-2.740153	-0.896943	3.686675	H
O-O2--0.793000	0	-1.663076	-2.837410	3.877939	H
O-OW--0.834000	0	2.909873	-5.969384	-3.937468	L
H-HW-0.417000	0	3.707430	-6.068044	-3.378048	L
H-HW-0.417000	0	2.205447	-5.832339	-3.286996	L
O-OW--0.834000	0	-2.439285	-6.834481	2.423164	L
H-HW-0.417000	0	-3.138757	-7.078483	1.788491	L
H-HW-0.417000	0	-2.938812	-6.303453	3.054829	L
O-OW--0.834000	0	4.171646	0.158485	4.143607	L
H-HW-0.417000	0	3.809440	-0.671427	3.813153	L
H-HW-0.417000	0	4.133972	0.743214	3.358304	L
O-OW--0.834000	0	6.373172	-1.335656	4.739955	L
H-HW-0.417000	0	5.978103	-2.227794	4.745356	L
H-HW-0.417000	0	5.590047	-0.762283	4.682621	L
O-OW--0.834000	0	-4.864175	-6.944316	1.075416	L
H-HW-0.417000	0	-5.060434	-6.066210	0.677625	L
H-HW-0.417000	0	-5.723808	-7.372926	1.069839	L
O-OW--0.834000	0	-8.877777	-0.484805	-0.066902	L
H-HW-0.417000	0	-8.977371	0.033342	-0.883069	L
H-HW-0.417000	0	-9.035748	-1.410267	-0.333587	L
